# Comparative efficacy and safety of pharmacological interventions for the treatment of COVID-19: A systematic review and network meta-analysis

**DOI:** 10.1371/journal.pmed.1003501

**Published:** 2020-12-30

**Authors:** Min Seo Kim, Min Ho An, Won Jun Kim, Tae-Ho Hwang

**Affiliations:** 1 Korea University, College of Medicine, Seoul, Republic of Korea; 2 Cheongsan Public Health Center, Wando, Republic of Korea; 3 Ajou University, School of Medicine, Suwon, Republic of Korea; 4 So Ahn Public Health Center, Wando, Republic of Korea; 5 Gangneung Prison Medical Department, Ministry of Justice, Republic of Korea; 6 Department of Pharmacology, Pusan National University, School of Medicine, Yangsan, Republic of Korea; 7 Gene and Cell Therapy Research Center for Vessel-associated Diseases, School of Medicine, Pusan National University, Yangsan, Republic of Korea; Universitair Medisch Centrum Utrecht, NETHERLANDS

## Abstract

**Background:**

Numerous clinical trials and observational studies have investigated various pharmacological agents as potential treatment for Coronavirus Disease 2019 (COVID-19), but the results are heterogeneous and sometimes even contradictory to one another, making it difficult for clinicians to determine which treatments are truly effective.

**Methods and findings:**

We carried out a systematic review and network meta-analysis (NMA) to systematically evaluate the comparative efficacy and safety of pharmacological interventions and the level of evidence behind each treatment regimen in different clinical settings. Both published and unpublished randomized controlled trials (RCTs) and confounding-adjusted observational studies which met our predefined eligibility criteria were collected. We included studies investigating the effect of pharmacological management of patients hospitalized for COVID-19 management. Mild patients who do not require hospitalization or have self-limiting disease courses were not eligible for our NMA. A total of 110 studies (40 RCTs and 70 observational studies) were included. PubMed, Google Scholar, MEDLINE, the Cochrane Library, medRxiv, SSRN, WHO International Clinical Trials Registry Platform, and ClinicalTrials.gov were searched from the beginning of 2020 to August 24, 2020. Studies from Asia (41 countries, 37.2%), Europe (28 countries, 25.4%), North America (24 countries, 21.8%), South America (5 countries, 4.5%), and Middle East (6 countries, 5.4%), and additional 6 multinational studies (5.4%) were included in our analyses. The outcomes of interest were mortality, progression to severe disease (severe pneumonia, admission to intensive care unit (ICU), and/or mechanical ventilation), viral clearance rate, QT prolongation, fatal cardiac complications, and noncardiac serious adverse events. Based on RCTs, the risk of progression to severe course and mortality was significantly reduced with corticosteroids (odds ratio (OR) 0.23, 95% confidence interval (CI) 0.06 to 0.86, *p* = 0.032, and OR 0.78, 95% CI 0.66 to 0.91, *p* = 0.002, respectively) and remdesivir (OR 0.29, 95% CI 0.17 to 0.50, *p* < 0.001, and OR 0.62, 95% CI 0.39 to 0.98, *p* = 0.041, respectively) compared to standard care for moderate to severe COVID-19 patients in non-ICU; corticosteroids were also shown to reduce mortality rate (OR 0.54, 95% CI 0.40 to 0.73, *p* < 0.001) for critically ill patients in ICU. In analyses including observational studies, interferon-alpha (OR 0.05, 95% CI 0.01 to 0.39, *p* = 0.004), itolizumab (OR 0.10, 95% CI 0.01 to 0.92, *p* = 0.042), sofosbuvir plus daclatasvir (OR 0.26, 95% CI 0.07 to 0.88, *p* = 0.030), anakinra (OR 0.30, 95% CI 0.11 to 0.82, *p* = 0.019), tocilizumab (OR 0.43, 95% CI 0.30 to 0.60, *p* < 0.001), and convalescent plasma (OR 0.48, 95% CI 0.24 to 0.96, *p* = 0.038) were associated with reduced mortality rate in non-ICU setting, while high-dose intravenous immunoglobulin (IVIG) (OR 0.13, 95% CI 0.03 to 0.49, *p* = 0.003), ivermectin (OR 0.15, 95% CI 0.04 to 0.57, *p* = 0.005), and tocilizumab (OR 0.62, 95% CI 0.42 to 0.90, *p* = 0.012) were associated with reduced mortality rate in critically ill patients. Convalescent plasma was the only treatment option that was associated with improved viral clearance rate at 2 weeks compared to standard care (OR 11.39, 95% CI 3.91 to 33.18, *p* < 0.001). The combination of hydroxychloroquine and azithromycin was shown to be associated with increased QT prolongation incidence (OR 2.01, 95% CI 1.26 to 3.20, *p* = 0.003) and fatal cardiac complications in cardiac-impaired populations (OR 2.23, 95% CI 1.24 to 4.00, *p* = 0.007). No drug was significantly associated with increased noncardiac serious adverse events compared to standard care. The quality of evidence of collective outcomes were estimated using the Grading of Recommendations Assessment, Development, and Evaluation (GRADE) framework. The major limitation of the present study is the overall low level of evidence that reduces the certainty of recommendations. Besides, the risk of bias (RoB) measured by RoB2 and ROBINS-I framework for individual studies was generally low to moderate. The outcomes deducted from observational studies could not infer causality and can only imply associations. The study protocol is publicly available on PROSPERO (CRD42020186527).

**Conclusions:**

In this NMA, we found that anti-inflammatory agents (corticosteroids, tocilizumab, anakinra, and IVIG), convalescent plasma, and remdesivir were associated with improved outcomes of hospitalized COVID-19 patients. Hydroxychloroquine did not provide clinical benefits while posing cardiac safety risks when combined with azithromycin, especially in the vulnerable population. Only 29% of current evidence on pharmacological management of COVID-19 is supported by moderate or high certainty and can be translated to practice and policy; the remaining 71% are of low or very low certainty and warrant further studies to establish firm conclusions.

## Abstract 2

In this meta-analysis, Min Seo Kim and colleagues synthesise results from randomized trials and observational studies on COVID-19 treatments.

## Author summary

**Why was the study done?:**

- Numerous clinical trials and observational studies have investigated various pharmacological agents as potential treatments for Coronavirus Disease 2019 (COVID-19), but systematic synthesis of this large body of information is not readily available.
- Results from these studies are heterogeneous and sometimes even contradictory to one another, making it difficult for clinicians to determine which treatments are truly effective.
- Level of evidence behind these drugs are diverse and must be classified into categories to effectively inform policy and practice.

**What did the researchers do and find?:**

- In randomized controlled trials (RCTs), remdesivir and corticosteroid were shown to reduce COVID-19 aggravation and mortality rates.
- In the whole dataset, including data from RCTs and observational studies, anti-inflammatory agents (corticosteroid, tocilizumab, anakinra, and IVIG), convalescent plasma, and remdesivir were associated with improved clinical outcomes of COVID-19.
- Hydroxychloroquine provides no benefit in mitigating COVID-19 disease course while posing safety risks, especially to vulnerable populations.

**What do these findings mean?:**

- These findings could help prioritize further research on drugs of possible benefit.
- Only 29% of current evidence on pharmacological management of COVID-19 is based on moderate/high evidence certainty and can be reflected in practice and policy; remaining 71% are of low or very low evidence certainty and warrant further studies to establish firm conclusions.

## Introduction

Numerous Coronavirus Disease 2019 (COVID-19) clinical trials and observational studies are underway, and over 47 pharmacological agents and regimens have been investigated as potential treatments of COVID-19. However, despite all such research efforts, conclusive consensus on treatment is still lacking. To the contrary, the increasing volume of information on the pharmacologic management of COVID-19 patients has led to a wider divergence in the management of COVID patients across institutions worldwide. Although studies supporting these various treatment regimens have been published, they utilize different designs, investigating different medications under various settings; as a result, the evidence on pharmacologic treatment of COVID-19 is scattered and heterogeneous. A network meta-analysis (NMA) enables a single coherent ranking of such numerous interventions, and it can thus aid decision-makers who must choose among an array of treatment options [1].

We conducted an NMA with selective predefined eligibility criteria for both published and unpublished data and investigated 47 treatment regimens for comparative efficacy and safety. We incorporated 110 studies (40 randomized controlled trials (RCTs) and 70 confounder-adjusted observational studies). The level of certainty behind the evidence for each outcome was also evaluated to assist the decision-making of clinicians and policy makers.

## Methods

### Search strategy and selection criteria

We searched PubMed, Google Scholar, MEDLINE, the Cochrane Library, medRxiv, SSRN, WHO International Clinical Trials Registry Platform, and ClinicalTrials.gov for RCTs and observational studies that evaluated treatment responses to pharmacological management in COVID-19 patients, from the beginning of 2020 to August 24, 2020. Reference lists of review articles were also reviewed to search for additional articles that may not have been retrieved by the prespecified searching strategy. We had no restriction on language, but all included studies were written in English. This study was reported as per the Preferred Reporting Items for Systematic Reviews and Meta-Analyses (PRISMA) guideline [2] (S1 PRISMA Checklist). The study protocol is publicly available on PROSPERO (CRD42020186527) and medRxiv [3]. Since this review did not involve any individual patient data, ethical approval was not required.

Participants, interventions, comparisons, outcomes, and study design (PICOS) of all included studies are described in S1 Table. We included studies investigating the effect of pharmacological management of patients hospitalized for COVID-19 management. Mild patients who do not require hospitalization or have self-limiting disease courses were not eligible for our network meta-analysis (NMA). The outcomes of interest were mortality, progression to severe disease (severe pneumonia, admission to intensive care unit (ICU), and/or mechanical ventilation), viral clearance rate, QT prolongation, fatal cardiac complications, and noncardiac serious adverse events.

We contacted principal investigators of unpublished studies identified in trial registries and regulatory submissions to obtain unpublished data. Inclusion of unpublished data in NMAs is not uncommon [4–11] and reduces risk of selection and publication bias while increasing the density of study data. This is especially beneficial in the study of COVID-19 as all data were generated relatively recently, and the bulk of the relevant data are still in the unpublished stages. Preprints have been used in meta-analysis relatively frequently for the urgent topic of COVID-19 [9–12], and American Gastroenterological Association (AGA) recently published a management guideline for the gastrointestinal manifestation of COVID-19 patients based on the result of meta-analysis incorporating preprints [11]. We contacted authors of included preprints from medRxiv and SSRN, and any change in the results was updated.

We included both RCTs and baseline-adjusted observational studies; the rationale is that inclusion of real-world data from nonrandomized studies has the potential to improve precision of findings from RCTs if appropriately integrated [13] and that the volume of information provided by these studies is necessary to assess adverse events of low to moderate incidence [14–16]. As observational studies are more vulnerable to bias, we included only the studies that accounted for relevant confounding variables by showing that baseline characteristics were similar (*p* > 0.05 for baseline characteristics) or through methods such as propensity score matching (PSM), subgroup analyses, or regression model adjustment.

Following studies were excluded: studies without a proper control group; studies of children or adolescents (<18 years) as they may have a different disease course [17–19]; observational studies with significant differences in baseline characteristics between groups and did not perform adequate adjustments; and studies investigating the effect of medication initiated prior to the diagnosis of COVID-19 (e.g., ACEi/ARB for hypertensive patients).

### Data extraction and quality assessment

The study search and data extraction were independently conducted by 3 authors (MS Kim, MH An, and WJ Kim). Manuscript and supplementary materials of the included studies were reviewed for relevant information which was extracted according to a prespecified protocol. Any discrepancy or ambiguity in this process was resolved by discussion. Authors of certain included studies were contacted in case of missing or unclear information. Nonrandomized studies were qualitatively assessed using the Newcastle-Ottawa Scale (NOS) [20], and RCTs were assessed with the Jadad scale [21]. All studies were assessed for risk of bias (RoB) using the Risk of Bias 2 (RoB2) tool for randomized studies and Risk of Bias in Nonrandomized Studies of Interventions (ROBINS-I) tool for nonrandomized studies [22]. The quality of evidence of collective outcomes were estimated using the Grading of Recommendations Assessment, Development, and Evaluation (GRADE) framework [23]. A comparison-adjusted funnel plot with Egger test was constructed to assess for publication bias [24].

Control groups consisted of patients who received standard care or placebo. Patients who received hydroxychloroquine or corticosteroids were subdivided according to the dosage they received. For hydroxychloroquine, most studies reported 400 mg hydroxychloroquine daily for maintenance, and this was considered the standard prescription; patients who received daily maintenance dosage of over 600 mg (>600 mg/day) hydroxychloroquine were classified into a separate high-dose hydroxychloroquine group. For corticosteroids, average daily dosage of 40 mg methylprednisolone (or equivalent) was regarded as the standard dosage, while 1 to 2 mg/kg/day methylprednisolone (or equivalent) was regarded as high dose. A total of 1 mg methylprednisolone was considered equivalent to 0.1875 mg dexamethasone and 5 mg hydrocortisone.

A critically ill patient was defined as a patient who received invasive mechanical ventilation or needed intensive care in the ICU before or soon after beginning the treatment of interest, while moderate-severe patients were defined as patients hospitalized in a non-ICU setting at admission. The mortality rate of patients included in our mortality analyses were 13.1% for moderate-severe (non-ICU) patients and 40.5% for critically ill (ICU) patients on average.

### Data synthesis and statistical analysis

We conducted a random-effects NMA within a frequentist framework using STATA (Stata Corp, College Station, Texas, United States of America, version 15.0) and R (version 3.6.0) software [25]. Direct and indirect (and mixed) comparison were accomplished through the self-programmed routines of STATA [24,26] and the netmeta package of R [27]. Further details of the methodology for NMA are described elsewhere [28,29]. The effect estimation was in odds ratios (OR) for dichotomous variables and mean difference (MD) for continuous variables, both with 95% confidential intervals (CI). When median (interquartile range) was presented for continuous variables of interest, it was converted to mean (standard deviation) by calculation [30,31]. A 2-sided *p*-value of less than 0.05 was regarded as statistically significant.

Statistical heterogeneity was estimated using restricted maximum likelihood method [32] and expressed with Higgins I^2^ statistics and the Cochran Q test [33]. The net heat plot was constructed to visualize the inconsistency matrix and detect specific comparisons which introduced large inconsistencies [34]. The rank of effect estimation for each treatment was investigated using the surface under the cumulative rank curve (SUCRA) of P rank score of R [35].

Prespecified subgroup and sensitivity analyses were performed to determine whether the results were affected by the patient severity, treatment protocol, and study design. The primary outcomes were separately analyzed for moderate to severe patients (non-ICU at admission) and critically ill patients (ICU) as these patients may respond differently to treatments. Sensitivity analyses were conducted by restricting the analyses to only RCTs, only published studies, and excluding studies with high/serious RoB.

## Results

**Fig 1:**
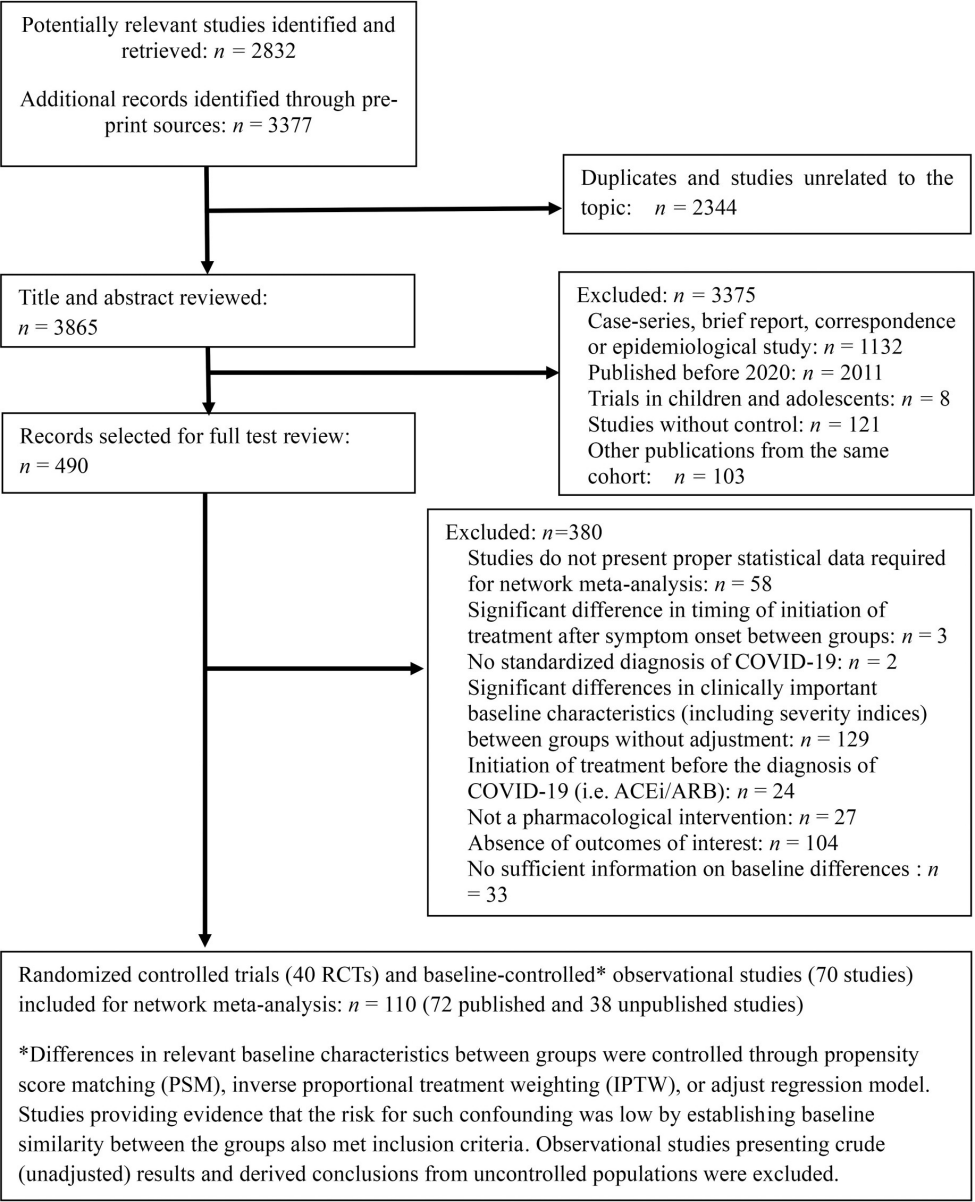
PRISMA diagram showing selection of articles for pairwise and network meta-analysis.

The initial search identified 6,209 articles. These studies were assessed for inclusion using the prespecified inclusion and exclusion criteria described in methods. Title and abstract of 3,865 articles were assessed, and 490 studies were found suitable for full-text review. After excluding 380 studies, 40 RCTs and 70 observational studies were finally included in our NMA (Fig 1). A total of 49,569 COVID-19 patients were included. Background characteristics and reference list of included studies are presented in S1 Table. The RoB in included studies were generally low to moderate (S2 Table).

**Fig 2:**
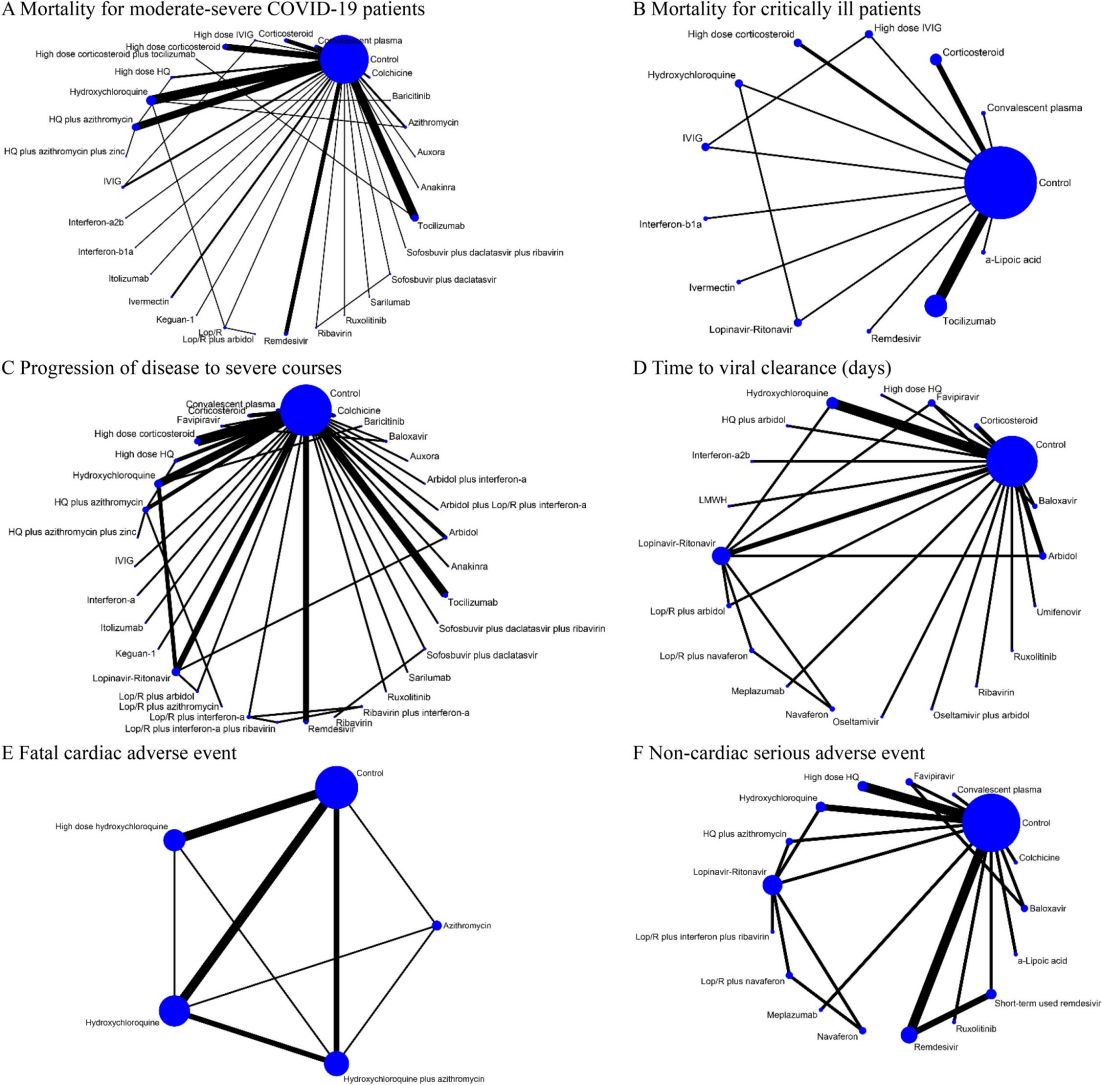
Network of eligible comparisons for primary outcomes (A) Mortality for moderate-severe COVID-19 patients (non-ICU at admission). (B) Mortality for critically ill patients (ICU). (C) Progression of disease to severe courses (i.e., progression to severe pneumonia, admission to ICU, and/or mechanical ventilation). (D) Time to viral clearance (days). (E) Fatal cardiac adverse events (torsades de pointes, cardiac arrest, and severe ventricular arrhythmia). (F) Noncardiac serious adverse events. Lines indicate direct comparison of agents, and the thickness of line corresponds to the number of trials in the comparison. Size of node corresponds to the number of studies that involve the intervention. HQ, hydroxychloroquine; ICU, intensive care unit; Lop/R, lopinavir-ritonavir.

For both pairwise meta-analysis and NMA, the primary outcomes presented no evidence of heterogeneity (S3 Table), except for mortality rate of critically ill patients (I^2^ = 62.0%) and viral clearance rate (I^2^ = 83.8%). Inconsistency, which represents discordance of direct and indirect comparisons, was also evaluated for outcomes, but none were subject to global inconsistency. We visualized the network of comparisons as shown in Fig 2. In the network, each regimen is represented by a unique node, meaning different nodes were designated for different dosages of the same drug. Lines indicate direct head-to-head comparison of agents, and the thickness of line corresponds to the number of trials in the comparison. Size of the node corresponds to the number of studies behind the intervention. Detailed information of studies included in the analysis for cardiac adverse events are presented in Table 1, and the certainty of evidence (GRADE) for each outcome is summarized in Table 2.

**Table 1:** Studies included for analysis of QT prolongation and fatal cardiac complications after taking hydroxychloroquine alone or hydroxychloroquine with azithromycin.

Study	Baseline characteristics	Assessed fatal cardiac complications	Incidence (%)
Studies including relatively high portion of patients with poor cardiac function (>10% of patients have CAD/CHD*)			
Rosenberg et al. [65]	Proportion of patients with cardiovascular comorbidities was high at baseline (approximately 30%), and proportions of cardiovascular comorbidities were significantly different between groups in crude analysis. However, adjustments were made for sex, age category (<65 vs ≥65 years), diabetes, any chronic lung disease, cardiovascular disease, abnormal chest imaging, respiration rate >22/min, O2 saturation <90%, elevated creatinine, and AST >40 U/L. Adjusted odds ratios for prolonged QT interval were presented. Obesity was significantly higher in pharmacologic treatment groups (hydroxychloroquine, hydroxychloroquine, alone, and azithromycin alone) compared to control (neither drug). Obesity was not adjusted for. Median age 63 Coronary heart disease (12.7%)	Cardiac arrest	Hydroxychloroquine+Azithromycin: 15.5% Hydroxychloroquine alone: 13.6% Azithromycin alone: 6.1% Neither drugs: 7.1%
Mercuro et al. [66]	Baseline QTc interval was longer in the hydroxychloroquine group compared to hydroxychloroquine plus azithromycin group. Therefore, we used the change in QTc intervals (ΔQTc) in each group for analysis. Mean age 60.1 Coronary heart disease (11.1%) and atrial fibrillation (13.3%)	Torsades de pointes	Hydroxychloroquine+Azithromycin: .8% Hydroxychloroquine: 0%
Ramireddy et al. [67]	Baseline QTc intervals were significantly different between groups. Therefore, we used change in QTc intervals (posttreatment QTc-baseline QTc, ΔQTc) of each group for analysis. Mean age 62.3, mean BMI 27.8 Heart failure (20%)	Syncope, torades de pointes, or other lethal arrhythmias	Azithromycin alone: 0% Hydroxychloroquine+Azithromycin: 0%
Saleh et al. [68]	Sex, structural heart disease, cirrhosis, other medications known to cause QT prolongation were comparable between hydroxychloroquine and hydroxychloroquine plus azithromycin groups. Mean age 58.5, mean BMI 28.2 Mean ejection fraction (61.9%), coronary artery disease (11.4%)	Monomorphic ventricular arrhythmia	Hydroxychloroquine: 2.4% Hydroxychloroquine+Azithromycin: 5.0%
Bessiere et al. [69]	ICU setting. Median age 68, median BMI 28 Structural heart disease (20%)	Severe ventricular arrhythmia including torades de pointes	Hydroxychloroquine: 0% Hydroxychloroquine+Azithromycin: 0%
Borba et al. [70]	Randomized controlled trial. Generally, baseline characteristics were well controlled between groups, but preexisting heart disease was more frequent in the high dosage group (*p*-value not provided). Mean age 51.1 Heart disease (17.9%) in high-dose hydroxychloroquine group	Ventricular tachycardia	High-dose hydroxychloroquine: 4.8% Standard-dose hydroxychloroquine: 0%
Horby et al. [71]	Randomized controlled trial Mean age 65.3 Heart disease (26% but perhaps including hypertension)	Ventricular tachycardia or fibrillation	High-dose hydroxychloroquine: 0.9% No hydroxychloroquine: 0.7%
Studies including relatively low portion of patients with poor cardiac function (<10% of patients have CAD/CHD*)			
Mahevas et al. [72]	Baseline characteristics were generally balanced and controlled (i.e., COPD/asthma, HF, CVD, DM, CKD, LC, immunosuppression). Median age 60 Chronic heart failure (4%)	Severe arrhythmia	Hydroxychloroquine: 1.1% (first-degree atrioventricular block) No hydroxychloroquine: 1.0% (left bundle branch block)
Tang et al. [73]	Randomized controlled trial. Mild to moderate patients with low rate of cardiovascular comorbidity presence. Mean age 46.1, BMI 23.5 Hypertension was presented in 6% of patients, and the prevalence of structural heart disease is expected to be less than 6%	Severe arrhythmia	Hydroxychloroquine: 0% No hydroxychloroquine: 0%
An et al. [74]	Patients in the conservative treatment group had milder baseline features than patients in the hydroxychloroquine plus azithromycin group. Mean age 42.2 and mean BMI 23 Very low prevalence of cardiovascular comorbidities, perhaps due to young age.	Cardiac arrest	Hydroxychloroquine+Azithromycin: 0% No hydroxychloroquine: 0%
Boulware et al. [75]	Randomized controlled trial. Mild patient group. Only the adverse event-related data from this study were used in this network meta-analysis, as this study investigates the effect of hydroxychloroquine as a prophylactic measure which is not the focus of our efficacy meta-analysis. Median age 41 Cardiovascular disease (0.7%, not include hypertension)	Cardiac arrhythmia	High-dose hydroxychloroquine: 0% No hydroxychloroquine: 0%
Skipper et al. [76]	Randomized controlled trial. Mild patient group. Only the adverse event-related data from this study were used in this network meta-analysis, as this study investigates the effect of early hydroxychloroquine intake in nonhospitalized COVID-19 or probable COVID-19 patients with high-risk exposure which is not the focus of our efficacy analysis. Median age 40 Cardiovascular disease (1.2%, not include hypertension)	Cardiac arrhythmia	High-dose hydroxychloroquine: 0% No hydroxychloroquine (placebo): 0%
Mitja et al. [77]	Randomized controlled trial. Mild patient group. Only the adverse event-related data from this study were used in this network meta-analysis, as this study investigates the effect of early hydroxychloroquine intake in nonhospitalized COVID-19 which is not the focus of our efficacy analysis. Median age 41.6 Cardiovascular disease (9.6% in control group and 14.7% in HQ group; however, note that this prevalence includes hypertension and therefore rate of structural/functional cardiac comorbidities such as CAD and CHD may be lower)	Cardiac arrhythmia	Hydroxychloroquine: 0% No hydroxychloroquine: 0%
Cavalcanti et al. [78]	Randomized controlled trial. Mild-to-moderate patient group. Median age 50.3 Heart failure (1.5%)	Cardiac arrhythmia/ventricular tachycardia	Hydroxychloroquine plus azithromycin: 1.3% High-dose hydroxychloroquine: 1.5% Azithromycin alone: 0% Control (neither medication): 0.6%
Mitja et al. [79]	Randomized controlled trial. Mild patient group (postexposure group). Only the adverse event-related data from this study were used in this network meta-analysis, as this study investigates the effect of prophylactic hydroxychloroquine intake in postexposure patients which is not the focus of our efficacy analysis. Median age 48.6 Cardiovascular disease (14.9% in control group and 11.6% in HQ group; however, note that the prevalence includes hypertension and therefore the prevalence of structural/functional cardiac comorbidities such as CAD and CHD may be lower)	Cardiac arrhythmia	Hydroxychloroquine: 0% No hydroxychloroquine: 0%
Lofgren et al. [80]	Randomized controlled trial. Mild patient group (post- and pre-exposure groups). Only the adverse event-related data from this study were used in this network meta-analysis, as this study investigates the effect of prophylactic hydroxychloroquine intake which is not the focus of our efficacy analysis. Median age 40 Mostly healthworkers	Ventricular tachycardia	Hydroxychloroquine (daily intake): 0% Placebo: 0%

*CAD/CHD, coronary artery disease/congestive heart disease.

CKD, chronic kidney disease; COPD, chronic obstructive pulmonary disease; COVID-19, Coronavirus Disease 2019; CVD, cardiovascular disease; DM, diabetes mellitus; HF, heart failure; ICU, intensive care unit; LC, liver cirrhosis.

**Table 2:** Certainty of evidence evaluated with GRADE framework.

Comparisons (vs. Control)	Study No. ^†^	Effect size (95% CI), *p*-value	Study design	Risk of bias	Inconsistency	Indirectness	Imprecision	Publication bias	GRADE
Mortality in moderate to severe patients (non-ICU at admission), odds ratio									
High-dose corticosteroid plus tocilizumab	1	0.04 (0.01, 0.17), *p* < 0.001	Observational study	Downgrade	Downgrade*	Downgrade	Downgrade	No downgrade	Very Low
Interferon-a2b	1	0.05 (0.01, 0.39), *p* = 0.004	Observational study	No downgrade	Downgrade*	No downgrade	Downgrade	No downgrade	Very Low
Baricitinib	1	0.05 (0.00, 1.04), *p* = 0.053	Observational study	No downgrade	Downgrade*	Downgrade	Downgrade	No downgrade	Very Low
Itolizumab	1	0.10 (0.01, 0.92), *p* = 0.042	Observational study	Downgrade	Downgrade*	No downgrade	Downgrade	No downgrade	Very Low
Sofosbuvir plus daclatasvir plus ribavirin	1	0.13 (0.01, 2.64), *p* = 0.184	RCT	Downgrade	Downgrade*	No downgrade	Downgrade	No downgrade	Very Low
Ruxolitinib	1	0.13 (0.01, 2.74), *p* = 0.190	RCT	No downgrade	Downgrade*	No downgrade	Downgrade	No downgrade	Low
Interferon-b1a	1	0.13 (0.01, 2.96), *p* = 0.201	RCT	No downgrade	Downgrade*	No downgrade	Downgrade	No downgrade	Low
Sofosbuvir plus daclatasvir	3	0.26 (0.07, 0.88), *p* = 0.030	RCT	No downgrade	No downgrade	No downgrade	No downgrade	No downgrade	High
Anakinra	1	0.30 (0.11, 0.82), *p* = 0.019	Observational study	No downgrade	Downgrade*	No downgrade	No downgrade	No downgrade	Very Low
High-dose corticosteroid	7	0.38 (0.24, 0.63), *p* < 0.001	Observational study	No downgrade	No downgrade	No downgrade	No downgrade	No downgrade	Moderate^‡^
Tocilizumab	9	0.43 (0.30, 0.60), *p* < 0.001	Observational study	No downgrade	No downgrade	No downgrade	No downgrade	No downgrade	Low
Sarilumab	1	0.35 (0.06, 2.10), *p* = 0.251	Observational study	No downgrade	Downgrade*	No downgrade	Downgrade	No downgrade	Very Low
Keguan-1	1	0.31 (0.01, 8.11), *p* = 0.482	RCT	Downgrade	Downgrade*	No downgrade	Downgrade	No downgrade	Very Low
Colchicine	2	0.39 (0.07, 2.33), *p* = 0.302	RCT	No downgrade	No downgrade	No downgrade	Downgrade	No downgrade	Moderate
High-dose IVIG	1	0.37 (0.02, 7.17), *p* = 0.511	Observational study	No downgrade	Downgrade*	No downgrade	Downgrade	No downgrade	Low^‡^
Convalescent plasma	3	0.48 (0.24, 0.96), *p* = 0.038	Observational study	No downgrade	No downgrade	No downgrade	No downgrade	No downgrade	Low
Remdesivir	4	0.52 (0.34, 0.80), *p* = 0.003	RCT	No downgrade	No downgrade	No downgrade	No downgrade	No downgrade	High
Auxora	1	0.47 (0.05, 4.19), *p* = 0.499	RCT	Downgrade	Downgrade*	No downgrade	Downgrade	No downgrade	Very Low
HQ plus azithromycin plus zinc	1	0.56 (0.28, 1.10), *p* = 0.092	Observational study	No downgrade	Downgrade*	Downgrade	No downgrade	No downgrade	Very Low
LOP/R plus arbidol	1	0.58 (0.03, 10.79), *p* = 0.715	Observational study	Downgrade	Downgrade*	No downgrade	Downgrade	No downgrade	Very Low
LOP/R	3	0.66 (0.33, 1.35), *p* = 0.255	Observational study	No downgrade	No downgrade	No downgrade	No downgrade	No downgrade	Low
Azithromycin	2	0.70 (0.46, 1.09), *p* = 0.114	Observational study	No downgrade	No downgrade	No downgrade	No downgrade	No downgrade	Low
Ivermectin	2	0.76 (0.25, 2.33), *p* = 0.631	Observational study	No downgrade	No downgrade	No downgrade	No downgrade	No downgrade	Low
Ribavirin	2	0.86 (0.33, 2.24), *p* = 0.758	Observational study	No downgrade	No downgrade	No downgrade	No downgrade	No downgrade	Low
IVIG	2	1.00 (0.25, 3.96), *p* = 1.000	RCT	No downgrade	No downgrade	No downgrade	Downgrade	No downgrade	High^‡^
HQ	11	0.93 (0.77, 1.13), *p* = 0.465	Observational study	No downgrade	No downgrade	No downgrade	No downgrade	No downgrade	Moderate^‡^
Corticosteroid	4	1.00 (0.74, 1.33), *p* = 1.000	Observational study	No downgrade	No downgrade	No downgrade	No downgrade	No downgrade	Moderate^‡^
High-dose HQ	2	1.15 (0.77, 1.71), *p* = 0.490	RCT	No downgrade	No downgrade	No downgrade	No downgrade	No downgrade	High^‡^
HQ plus azithromycin	8	1.13 (0.89, 1.44), *p* = 0.323	Observational study	No downgrade	No downgrade	No downgrade	No downgrade	No downgrade	Moderate^‡^
Mortality in critically ill patients (ICU), odds ratio									
High-dose IVIG	1	0.13 (0.03, 0.49), *p* = 0.003	Observational study	No downgrade	Downgrade*	No downgrade	Downgrade	No downgrade	Low^‡^
Ivermectin	1	0.15 (0.04, 0.57), *p* = 0.005	Observational study	No downgrade	Downgrade*	No downgrade	Downgrade	No downgrade	Very Low
a-Lipoic acid	1	0.17 (0.02, 1.61), *p* = 0.122	RCT	No downgrade	Downgrade*	No downgrade	Downgrade	No downgrade	Low
HQ	1	0.45 (0.12, 1.70), *p* = 0.239	Observational study	No downgrade	Downgrade*	No downgrade	Downgrade	No downgrade	Low^‡^
Interferon-b1a	1	0.47 (0.11, 1.94), *p* = 0.297	RCT	No downgrade	Downgrade*	No downgrade	Downgrade	No downgrade	Low
Tocilizumab	6	0.62 (0.42, 0.90), *p* = 0.012	Observational study	No downgrade	Downgrade	No downgrade	No downgrade	No downgrade	Very Low
Convalescent plasma	1	0.72 (0.19, 2.72), *p* = 0.628	RCT	No downgrade	Downgrade*	No downgrade	Downgrade	No downgrade	Low
IVIG	1	0.73 (0.22, 2.45), *p* = 0.610	Observational study	No downgrade	Downgrade*	No downgrade	Downgrade	No downgrade	Low^‡^
High-dose corticosteroid	2	0.74 (0.38, 1.46), *p* = 0.385	RCT	No downgrade	Downgrade	No downgrade	No downgrade	No downgrade	High^‡^
LOP/R	1	0.78 (0.19, 3.27), *p* = 0.734	Observational study	No downgrade	Downgrade*	No downgrade	Downgrade	No downgrade	Very Low
Remdesivir	1	0.92 (0.38, 2.25), *p* = 0.855	RCT	No downgrade	Downgrade*	No downgrade	No downgrade	No downgrade	Moderate
Corticosteroid	3	0.91 (0.50, 1.68), *p* = 0.763	Observational study	No downgrade	Downgrade	No downgrade	No downgrade	No downgrade	Low^‡^
Progression to severe course (progress to severe pneumonia or admission to ICU), odds ratio									
Baricitinib	1	0.02 (0.00, 0.32), *p* = 0.006	Observational study	No downgrade	Downgrade*	Downgrade	Downgrade	No downgrade	Very Low
High-dose corticosteroid	5	0.11 (0.06, 0.19), *p* < 0.001	Observational study	No downgrade	No downgrade	No downgrade	No downgrade	No downgrade	Moderate^‡^
Sofosbuvir plus daclatasvir plus ribavirin	1	0.09 (0.00, 1.85), *p* = 0.119	RCT	Downgrade	Downgrade*	No downgrade	Downgrade	No downgrade	Very Low
Ruxolitinib	1	0.09 (0.00, 1.91), *p* = 0.122	RCT	No downgrade	Downgrade*	No downgrade	Downgrade	No downgrade	Low
Auxora	1	0.17 (0.03, 1.07), *p* = 0.059	RCT	Downgrade	Downgrade*	No downgrade	No downgrade	No downgrade	Low
Anakinra	1	0.22 (0.09, 0.56), *p* = 0.002	Observational study	No downgrade	Downgrade*	No downgrade	No downgrade	No downgrade	Very Low
IVIG	1	0.20 (0.03, 1.22), *p* = 0.081	RCT	No downgrade	Downgrade*	No downgrade	No downgrade	No downgrade	High^‡^
Keguan-1	1	0.18 (0.01, 3.90), *p* = 0.275	RCT	Downgrade	Downgrade*	No downgrade	Downgrade	No downgrade	Very Low
Remdesivir	3	0.28 (0.16, 0.50), *p* < 0.001	RCT	No downgrade	No downgrade	No downgrade	No downgrade	No downgrade	High
Itolizumab	1	0.26 (0.07, 1.03), *p* = 0.055	Observational study	Downgrade	Downgrade*	No downgrade	No downgrade	No downgrade	Very Low
Arbidol plus interferon-a	1	0.23 (0.01, 5.34), *p* = 0.360	Observational study	No downgrade	Downgrade*	No downgrade	Downgrade	No downgrade	Very Low
Colchicine	2	0.33 (0.06, 1.89), *p* = 0.213	RCT	No downgrade	No downgrade	No downgrade	No downgrade	No downgrade	High
Tocilizumab	4	0.39 (0.24, 0.62), *p* = 0.001	Observational study	No downgrade	No downgrade	No downgrade	No downgrade	No downgrade	Low
Sofosbuvir plus daclatasvir	3	0.37 (0.09, 1.62), *p* = 0.187	RCT	No downgrade	No downgrade	No downgrade	No downgrade	No downgrade	High
Ribavirin plus interferon-a	1	0.33 (0.02, 6.41), *p* = 0.464	RCT	No downgrade	Downgrade*	Downgrade	Downgrade	No downgrade	Very Low
Arbidol	2	0.49 (0.15, 1.64), *p* = 0.247	RCT	No downgrade	No downgrade	No downgrade	No downgrade	No downgrade	High
Corticosteroid	2	0.51 (0.35, 0.76), *p* < 0.001	Observational study	No downgrade	No downgrade	No downgrade	No downgrade	No downgrade	Moderate^‡^
Convalescent plasma	2	0.53 (0.28, 0.98), *p* = 0.043	Observational study	No downgrade	No downgrade	No downgrade	No downgrade	No downgrade	Low
LOP/R plus interferon-a	2	0.62 (0.12, 3.26), *p* = 0.572	RCT	No downgrade	No downgrade	No downgrade	No downgrade	No downgrade	High
LOP/R plus interferon-a plus ribavirin	1	0.70 (0.05, 9.71), *p* = 0.790	RCT	No downgrade	Downgrade*	Downgrade	Downgrade	No downgrade	Very Low
Sarilumab	1	0.82 (0.23, 2.90), *p* = 0.758	Observational study	No downgrade	Downgrade*	No downgrade	No downgrade	No downgrade	Very Low
Arbidol plus LOP/R plus interferon-a	1	0.85 (0.16, 4.59), *p* = 0.850	Observational study	No downgrade	Downgrade*	No downgrade	Downgrade	No downgrade	Very Low
HQ	6	0.92 (0.56, 1.51), *p* = 0.742	Observational study	No downgrade	No downgrade	No downgrade	No downgrade	No downgrade	Moderate^‡^
HQ plus azithromycin plus zinc	1	0.96 (0.42, 2.18), *p* = 0.922	Observational study	No downgrade	Downgrade*	Downgrade	No downgrade	No downgrade	Very Low
Interferon-a	1	1.33 (0.15, 11.95), *p* = 0.799	Observational study	No downgrade	Downgrade*	No downgrade	Downgrade	No downgrade	Very Low
LOP/R	5	1.08 (0.53, 2.21), *p* = 0.833	Observational study	No downgrade	No downgrade	No downgrade	No downgrade	No downgrade	Low
High-dose HQ	2	1.12 (0.82, 1.53), *p* = 0.476	RCT	No downgrade	No downgrade	No downgrade	No downgrade	No downgrade	High^‡^
LOP/R plus arbidol	2	1.60 (0.28, 9.11), *p* = 0.596	Observational study	No downgrade	No downgrade	No downgrade	No downgrade	No downgrade	Low
Ribavirin	1	1.67 (0.25, 11.01), *p* = 0.594	Observational study	No downgrade	Downgrade*	Downgrade	No downgrade	No downgrade	Very Low
Baloxavir	1	3.31 (0.12, 91.51), *p* = 0.480	RCT	No downgrade	Downgrade*	No downgrade	Downgrade	No downgrade	Low
HQ plus azithromycin	3	1.76 (0.90, 3.44), *p* = 0.098	Observational study	No downgrade	No downgrade	No downgrade	No downgrade	No downgrade	Moderate^‡^
LOP/R plus azithromycin	1	4.78 (0.45, 51.11), *p* = 0.196	Observational study	No downgrade	Downgrade*	Downgrade	Downgrade	No downgrade	Very Low
Favipiravir	1	6.97 (0.29, 168.34), *p* = 0.232	RCT	No downgrade	Downgrade*	No downgrade	Downgrade	No downgrade	Low
Viral clearance rate, odds ratio									
LOP/R plus arbidol	1	15.83 (0.60, 417.8), *p* = 0.098	Observational study	Downgrade	Downgrade*	Downgrade	Downgrade	No downgrade	Very Low
Convalescent plasma	1	11.39 (0.89, 145.7), *p* = 0.061	RCT	No downgrade	Downgrade*	No downgrade	Downgrade	No downgrade	Low
Meplazumab	1	8.67 (0.48, 156.43), *p* = 0.143	Observational study	No downgrade	Downgrade*	No downgrade	Downgrade	No downgrade	Very Low
LOP/R plus navaferon	1	6.27 (0.28, 138.55), *p* = 0.245	RCT	No downgrade	Downgrade*	Downgrade	Downgrade	No downgrade	Very Low
Navaferon	1	3.51 (0.16, 76.55), *p* = 0.425	RCT	No downgrade	Downgrade*	Downgrade	Downgrade	No downgrade	Very Low
LOP/R	5	2.88 (0.50, 16.69), *p* = 0.238	Observational study	No downgrade	Downgrade	No downgrade	No downgrade	No downgrade	Very Low
Baloxavir	1	2.11 (0.14, 32.18), *p* = 0.591	RCT	No downgrade	Downgrade*	No downgrade	Downgrade	No downgrade	Low
Arbidol	1	1.69 (0.16, 18.46), *p* = 0.667	RCT	No downgrade	Downgrade*	No downgrade	Downgrade	No downgrade	Low
Favipiravir	2	1.61 (0.21, 12.32), *p* = 0.647	RCT	No downgrade	Downgrade	No downgrade	No downgrade	No downgrade	Moderate
HQ	5	1.34 (0.36, 5.08), *p* = 0.667	RCT	No downgrade	Downgrade	No downgrade	No downgrade	No downgrade	High^‡^
Umifenovir	1	0.79 (0.06, 9.88), *p* = 0.855	Observational study	No downgrade	Downgrade*	No downgrade	Downgrade	No downgrade	Very Low
Darunavir plus cobicistat	1	0.71 (0.05, 10.84), *p* = 0.806	RCT	Downgrade	Downgrade*	No downgrade	Downgrade	No downgrade	Very Low
Corticosteroid plus IVIG	1	0.63 (0.05, 7.67), *p* = 0.717	Observational study	Downgrade	Downgrade*	No downgrade	Downgrade	No downgrade	Low^‡^
High-dose HQ	1	0.58 (0.05, 6.46), *p* = 0.658	RCT	No downgrade	Downgrade*	No downgrade	Downgrade	No downgrade	Moderate^‡^
Time to viral clearance (days), mean difference									
Meplazumab	1	−10.00 (−16.79, −3.21), *p* = 0.045	Observational study	No downgrade	Downgrade*	No downgrade	Downgrade	No downgrade	Very Low
HQ	5	−4.01 (−5.28, −2.73), *p* = 0.011	Observational study	No downgrade	No downgrade	No downgrade	No downgrade	No downgrade	Moderate^‡^
Favipiravir	2	−3.83 (−6.77, −0.89), *p* = 0.009	RCT	No downgrade	No downgrade	No downgrade	No downgrade	No downgrade	High
Interferon-a2b	1	−3.00 (−8.17, 2.17), *p* = 0.615	Observational study	No downgrade	Downgrade*	No downgrade	No downgrade	No downgrade	Very Low
Corticosteroid	2	−2.12 (−5.19, 0.95), *p* = 0.464	Observational study	No downgrade	No downgrade	No downgrade	No downgrade	No downgrade	Moderate^‡^
Ribavirin	1	−1.30 (−3.41, 0.81), *p* = 0.521	Observational study	No downgrade	Downgrade*	No downgrade	No downgrade	No downgrade	Very Low
LOP/R plus navaferon	1	−0.59 (−2.82, 1.64), *p* = 0.453	RCT	No downgrade	Downgrade*	Downgrade	No downgrade	No downgrade	Low
Oseltamivir	1	0.15 (−2.21, 2.51), *p* = 0.948	Observational study	No downgrade	Downgrade*	No downgrade	No downgrade	No downgrade	Very Low
LMWH	1	0.28 (−8.99, 9.56), *p* = 0.974	Observational study	No downgrade	Downgrade*	No downgrade	Downgrade	No downgrade	Very Low
Navaferon	1	0.31 (−1.90, 2.52), *p* = 0.927	RCT	No downgrade	Downgrade*	Downgrade	No downgrade	No downgrade	Low
Ruxolitinib	1	0.72 (−5.34, 6.77), *p* = 0.911	RCT	No downgrade	Downgrade*	No downgrade	Downgrade	No downgrade	Low
HQ plus arbidol	1	1.26 (−9.46, 11.98), *p* = 0.687	Observational study	No downgrade	Downgrade*	No downgrade	Downgrade	No downgrade	Very Low
Umifenovir	1	0.93 (−2.64, 4.50), *p* = 0.817	Observational study	No downgrade	Downgrade*	No downgrade	No downgrade	No downgrade	Very Low
High-dose HQ	1	1.00 (−2.28, 4.28), *p* = 0.839	RCT	No downgrade	Downgrade*	No downgrade	No downgrade	No downgrade	High^‡^
Arbidol	2	0.96 (−0.85, 2.76), *p* = 0.489	RCT	No downgrade	No downgrade	No downgrade	No downgrade	No downgrade	High
LOP/R	6	1.31 (−0.27, 2.89), *p* = 0.137	Observational study	No downgrade	No downgrade	No downgrade	No downgrade	No downgrade	Low
Baloxavir	1	7.32 (−10.20, 24.84), *p* = 0.735	RCT	No downgrade	Downgrade*	No downgrade	Downgrade	No downgrade	Low
Oseltamivir plus arbidol	1	4.57 (−0.21, 9.36), *p* = 0.079	Observational study	No downgrade	Downgrade*	No downgrade	No downgrade	No downgrade	Very Low
LOP/R plus arbidol	2	4.12 (0.79, 7.45), *p* = 0.032	Observational study	No downgrade	No downgrade	No downgrade	No downgrade	No downgrade	Low
ΔQTc interval from baseline (msec) of HQ plus AZ and AZ alone compared to ΔQTc of control (HQ alone), mean difference									
HQ plus azithromycin (vs. HQ)		20.97 (12.60, 28.98), *p* < 0.001	Observational study	No downgrade	No downgrade	No downgrade	No downgrade	No downgrade	Low
Azithromycin (vs. HQ)		4.09 (−15.76, 23.94), *p* = 0.522	Observational study	Downgrade	Downgrade*	Downgrade	Downgrade	No downgrade	Very Low
Proportion of patients experiencing QTc prolongation (>500 ms or delta >60 ms), odds ratio									
High-dose HQ	3	2.24 (1.04, 4.82), *p* = 0.039	RCT	No downgrade	No downgrade	No downgrade	Downgrade	No downgrade	Moderate
HQ plus azithromycin	7	2.01 (1.26, 3.20), *p* = 0.003	Observational study	No downgrade	No downgrade	No downgrade	No downgrade	No downgrade	Low
HQ	7	1.39 (0.90, 2.17), *p* = 0.147	Observational study	No downgrade	No downgrade	No downgrade	No downgrade	No downgrade	Low
Azithromycin	3	1.09 (0.63, 1.87), *p* = 0.754	Observational study	No downgrade	No downgrade	No downgrade	No downgrade	No downgrade	Low
Fatal cardiac complication after HQ (TdP, cardiac arrest, and severe ventricular arrhythmia)—overall study, odds ratio									
HQ plus azithromycin	7	2.10 (1.23, 3.60), *p* = 0.007	Observational study	No downgrade	No downgrade	No downgrade	No downgrade	No downgrade	Low
HQ	9	1.53 (0.88, 2.66), *p* = 0.132	RCT	No downgrade	No downgrade	No downgrade	No downgrade	No downgrade	High
High-dose HQ	6	1.52 (0.73, 3.17), *p* = 0.264	RCT	No downgrade	No downgrade	No downgrade	Downgrade	No downgrade	Moderate
Azithromycin	2	0.55 (0.26, 1.18), *p* = 0.125	Observational study	No downgrade	No downgrade	No downgrade	Downgrade	No downgrade	Very Low
Fatal cardiac complication after HQ (TdP, cardiac arrest, and severe ventricular arrhythmia)—studies with CAD/CHD <10% at baseline									
High-dose HQ	4	1.34 (0.38, 4.71), *p* = 0.648	RCT	No downgrade	No downgrade	No downgrade	Downgrade	No downgrade	Moderate
HQ plus azithromycin	3	1.25 (0.27, 5.74), *p* = 0.774	RCT	No downgrade	No downgrade	No downgrade	Downgrade	No downgrade	Moderate
HQ	4	1.05 (0.26, 4.25), *p* = 0.946	RCT	No downgrade	No downgrade	No downgrade	Downgrade	No downgrade	Moderate
Fatal cardiac complication after HQ (TdP, cardiac arrest, and severe ventricular arrhythmia)—studies with CAD/CHD >10% at baseline									
HQ plus azithromycin	5	2.23 (1.24, 4.00), *p* = 0.007	Observational study	No downgrade	No downgrade	No downgrade	No downgrade	No downgrade	Low
HQ	5	1.65 (0.90, 3.02), *p* = 0.104	Observational study	No downgrade	No downgrade	No downgrade	No downgrade	No downgrade	Low
High-dose HQ	2	1.42 (0.54, 3.73), *p* = 0.477	RCT	No downgrade	No downgrade	No downgrade	Downgrade	No downgrade	Moderate
Azithromycin	2	0.59 (0.27, 1.30), *p* = 0.191	Observational study	No downgrade	No downgrade	No downgrade	No downgrade	No downgrade	Low
Noncardiac serious adverse events, odds ratio									
Ruxolitinib	1	0.09 (0.00, 1.89), *p* = 0.121	RCT	No downgrade	Downgrade*	No downgrade	Downgrade	No downgrade	Low
LOP/R plus interferon plus ribavirin	1	0.09 (0.00, 2.43), *p* = 0.152	RCT	No downgrade	Downgrade*	Downgrade	Downgrade	No downgrade	Very Low
Short-term used remdesivir	2	0.40 (0.26, 0.62), *p* < 0.001	RCT	No downgrade	No downgrade	No downgrade	No downgrade	No downgrade	High
LOP/R	5	0.58 (0.31, 1.10), *p* = 0.095	RCT	No downgrade	No downgrade	No downgrade	No downgrade	No downgrade	High
HQ	3	0.61 (0.26, 1.44), *p* = 0.259	Observational study	No downgrade	No downgrade	No downgrade	No downgrade	No downgrade	Low
Navaferon	1	0.58 (0.03, 10.51), *p* = 0.713	RCT	No downgrade	Downgrade*	Downgrade	Downgrade	No downgrade	Very Low
LOP/R plus navaferon	1	0.58 (0.03, 10.51), *p* = 0.713	RCT	No downgrade	Downgrade*	Downgrade	Downgrade	No downgrade	Very Low
Meplazumab	1	0.63 (0.04, 11.16), *p* = 0.753	Observational study	No downgrade	Downgrade*	No downgrade	Downgrade	No downgrade	Very Low
Remdesivir	4	0.71 (0.55, 0.90), *p* = 0.005	RCT	No downgrade	No downgrade	No downgrade	No downgrade	No downgrade	High
Baloxavir	1	1.00 (0.06, 16.76), *p* = 1.000	RCT	No downgrade	Downgrade*	No downgrade	Downgrade	No downgrade	Low
Favipiravir	1	1.00 (0.06, 16.76), *p* = 1.000	RCT	No downgrade	Downgrade*	No downgrade	Downgrade	No downgrade	Low
a-Lipoic acid	1	1.00 (0.06, 16.76), *p* = 1.000	RCT	No downgrade	Downgrade*	No downgrade	Downgrade	No downgrade	Low
HQ plus azithromycin	1	1.05 (0.14, 7.93), *p* = 0.962	Observational study	Downgrade	Downgrade*	No downgrade	Downgrade	No downgrade	Very Low
High-dose HQ	3	2.15 (0.98, 4.72), *p* = 0.056	RCT	No downgrade	No downgrade	No downgrade	No downgrade	No downgrade	High
Colchicine	1	4.72 (0.22, 100.72), *p* = 0.320	RCT	No downgrade	Downgrade*	No downgrade	Downgrade	No downgrade	Low
Convalescent plasma	1	5.10 (0.24, 108.86), *p* = 0.297	RCT	No downgrade	Downgrade*	No downgrade	Downgrade	No downgrade	Low

CAD, coronary artery disease; CHD, congestive heart disease; CI, confidence interval; GRADE, Grading of Recommendations Assessment, Development, and Evaluation; HQ, hydroxychloroquine; ICU, intensive care unit; IVIG, intravenous immunoglobulin; LMWH, low molecular weight heparin; LOP/R, lopinavir-ritonavir; RCT, randomized controlled trial.

* Downgraded by one when unable to evaluate inconsistency/heterogeneity due to lack of sufficient data (a single study).

^‡^ Upgrade by one for dose-response gradient.

^**†**^ Number of studies investigated on each intervention.

Rationale

Study design: If randomized trials form the evidence base, the quality rating starts at “high.” If observational studies form the evidence, base the quality rating starts at “low.”

Risk of bias: Downgraded for failure to conceal random allocation or blind participants in randomized controlled trials or failure to adequately control for confounding in observational studies.

Inconsistency: Downgraded if heterogeneity represented by I^2^ statistics or global inconsistency (Q statistic to assess consistency under the assumption of a full design-by-treatment interaction random effects model) was high.

Indirectness. Downgraded when assumption of transitivity is challenged, or the result is solely derived from indirect comparisons.

Imprecision: Downgraded when confidential interval (CI) is relatively too large compared to other active drugs.

Publication bias: Downgraded when substantial asymmetry is observed in funnel plot or *p* < 0.05 in Egger test.

GRADE Definition (suggested by Puhan et al. in “A GRADE Working Group approach for rating the quality of treatment effect estimates from network meta-analysis”)

High quality: We are very confident that the true effect lies close to that of the estimate of the effect.

Moderate quality: We are moderately confident in the effect estimate, i.e., the true effect is likely to be close to the estimate of the effect, but there is a possibility that it is substantially different.

Low quality: Our confidence in the effect estimate is limited, i.e., the true effect may be substantially different from the estimate of the effect.

Very low quality: We have very little confidence in the effect estimate, i.e., the true effect is likely to be substantially different from the estimate of effect.

### Mortality in non-ICU settings

**Fig 3:**
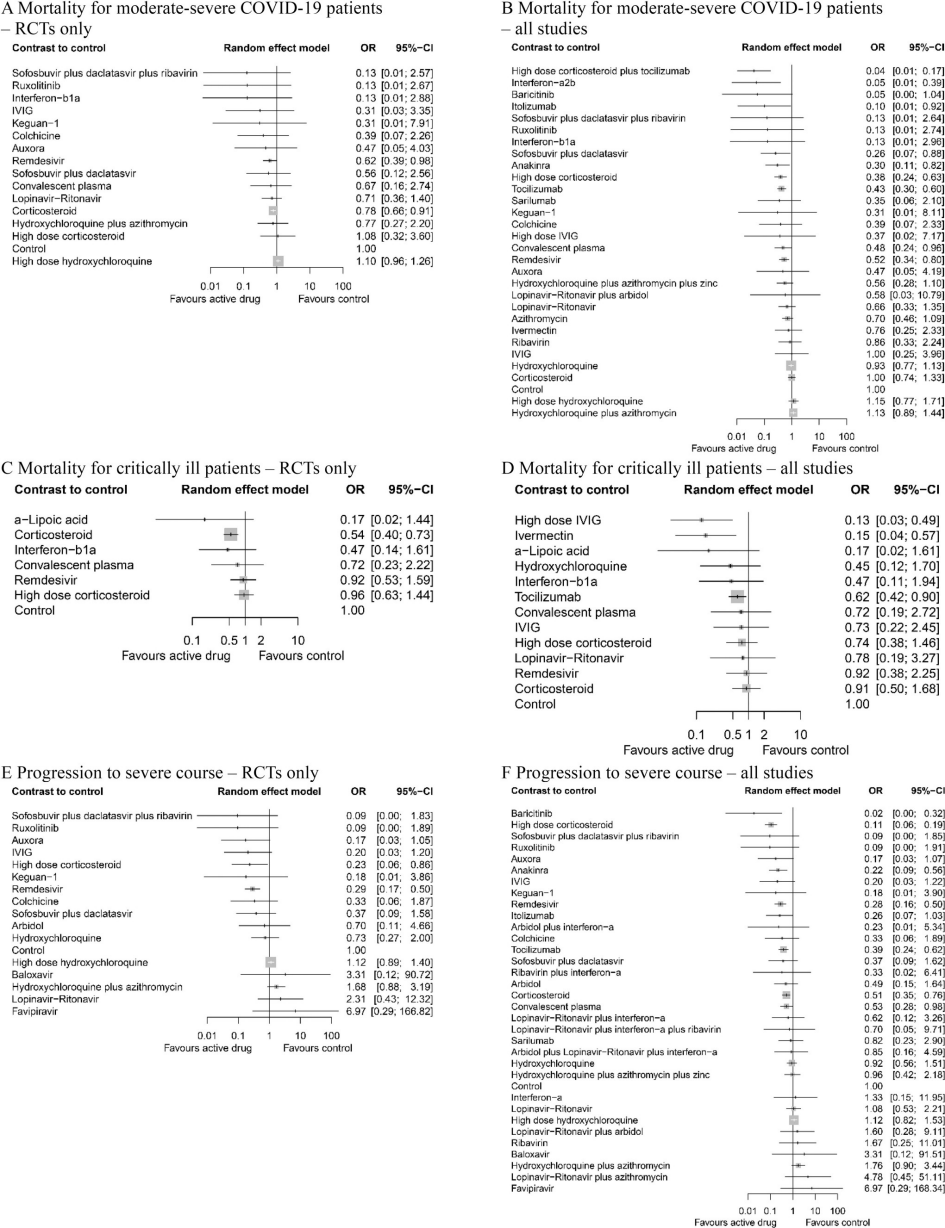
Network meta-analysis of pharmacological interventions compared with control (standard care) for efficacy outcomes. Mortality for moderate-severe patients (non-ICU at admission) from (A) RCTs and (B) all studies. Mortality for critically ill patients (ICU) from (C) RCTs and (D) all studies. Progression to severe course (i.e., progression to severe pneumonia, admission to ICU, and/or mechanical ventilation) from (E) RCTs and (F) all studies. Effect estimates are presented in OR with 95% CI. Pharmacological agents are ranked by SUCRA value. CI, confidence interval; ICU, intensive care unit; OR, odds ratio; RCT, randomized controlled trial; SUCRA, surface under the cumulative ranking curve.

Remdesivir (OR 0.62, 95% CI 0.39 to 0.98, *p* = 0.041) and corticosteroids (OR 0.78, 95% CI 0.66 to 0.91, *p* = 0.002) significantly reduced the mortality in moderate-to-severe patients in analysis of RCTs (Fig 3A). High-dose corticosteroid plus tocilizumab (OR 0.04, 95% CI 0.01 to 0.17, *p* < 0.001, very low (GRADE)), interferon-a2b (OR 0.05, 95% CI 0.01 to 0.39, *p* = 0.004, very low), itolizumab (OR 0.10, 95% CI 0.01 to 0.92, *p* = 0.042, very low), sofosbuvir plus daclatasvir (OR 0.26, 95% CI 0.07 to 0.88, *p* = 0.030, high), anakinra (OR 0.30, 95% CI 0.11 to 0.82, *p* = 0.019, very low), high-dose corticosteroid (OR 0.38, 95% CI 0.24 to 0.63, *p* < 0.001, moderate), tocilizumab (OR 0.43, 95% CI 0.30 to 0.60, *p* < 0.001, low), convalescent plasma (OR 0.48, 95% CI 0.24 to 0.96, *p* = 0.038, low), and remdesivir (OR 0.52, 95% CI 0.34 to 0.80, *p* = 0.003, high) were associated with reduced mortality in moderate-to-severe patients hospitalized in a non-ICU setting compared to the control group (Fig 3B).

### Mortality in ICU settings

In critically ill patients hospitalized in the ICU, corticosteroid (OR 0.54, 95% CI 0.40 to 0.73, *p* < 0.001) was the only agent showing effect in analysis with RCTs (Fig 3C). High-dose IVIG (OR 0.13, 95% CI 0.03 to 0.49, *p* = 0.003, low), ivermectin (OR 0.15, 95% CI 0.04 to 0.57, *p* = 0.005, very low), and tocilizumab (OR 0.62, 95% CI 0.42 to 0.90, *p* = 0.012, very low) were associated with lower mortality (Fig 3D).

### Progression to severe disease

High-dose corticosteroid (OR 0.23, 95% CI 0.06 to 0.86, *p* = 0.032) and remdesivir (OR 0.29, 95% CI 0.17 to 0.50, *p* < 0.001) were shown to be effective in the sensitivity analysis using only RCTs (Fig 3E). Baricitinib (OR 0.02, 95% CI 0.00 to 0.32, *p* = 0.006, very low), high-dose corticosteroid (OR 0.11, 95% CI 0.06 to 0.19, *p* < 0.001, moderate), anakinra (OR 0.22, 95% CI 0.09 to 0.56, *p* = 0.002, very low), remdesivir (OR 0.28, 95% CI 0.16 to 0.50, *p* < 0.001, high), tocilizumab (OR 0.39, 95% CI 0.24 to 0.62, *p* < 0.001, low), corticosteroid (OR 0.51, 95% CI 0.35 to 0.76, *p* < 0.001, moderate), and convalescent plasma (OR 0.53, 95% CI 0.28 to 0.98, *p* = 0.043, low) was associated with lower rate of progression to severe disease (Fig 3F).

### Viral clearance rate (negative conversion rate within 14 days)

**Fig 4:**
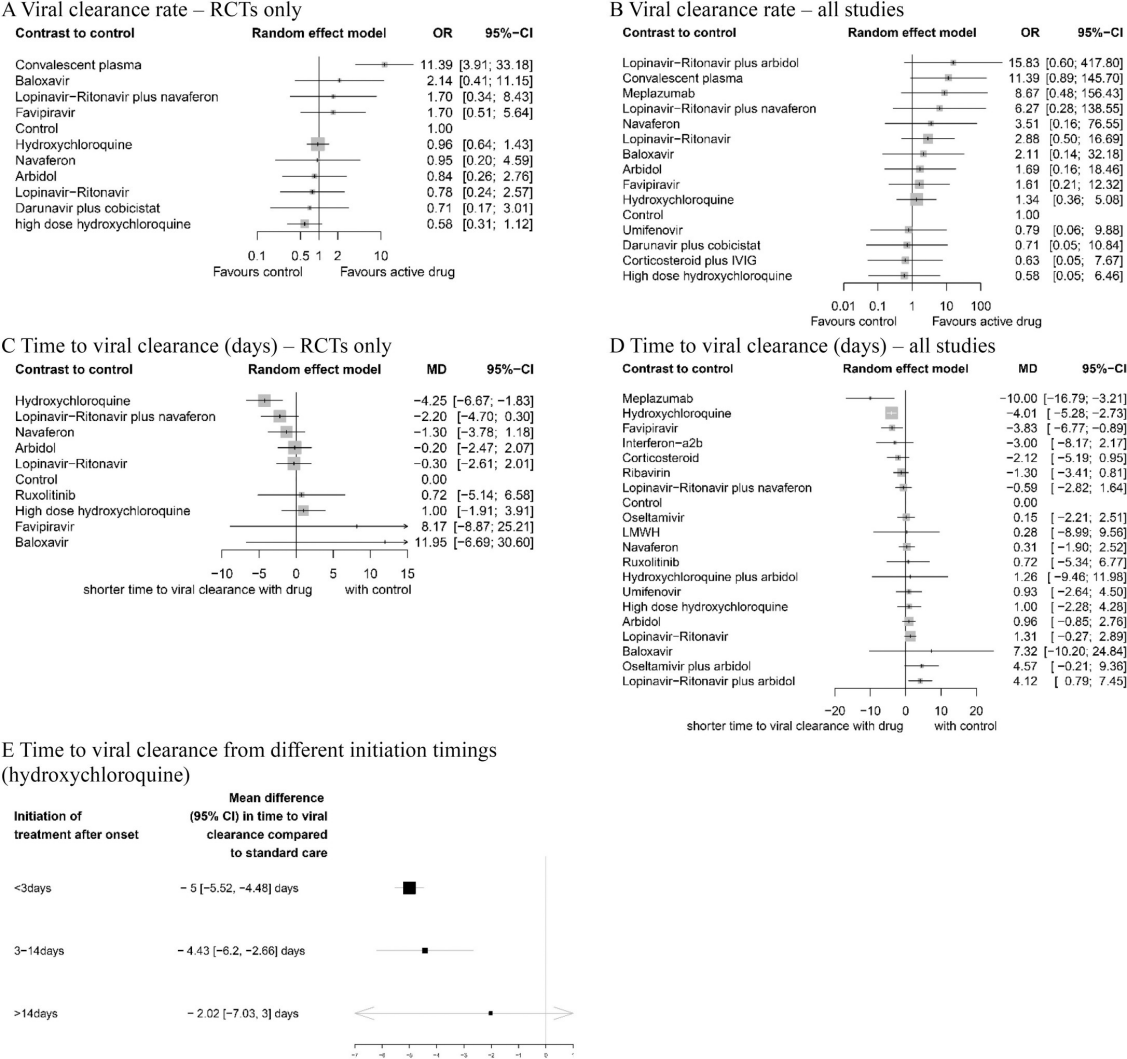
Network meta-analysis of pharmacological interventions compared with control (standard care) for viral clearance. Viral clearance rate (proportion of patients converted to PCR-negative status) from (A) RCTs and (B) all studies. Time to viral clearance (days) from (C) RCTs and (D) all studies. (E) Time to viral clearance from different hydroxychloroquine treatment initiation timings after symptom onset. Effect estimates are presented in OR for viral clearance rate and MD for time to viral clearance, with 95% CI. Pharmacological agents are ranked by SUCRA value. CI, confidence interval; MD, mean difference; OR, odds ratio; RCT, randomized controlled trial; SUCRA, surface under the cumulative ranking curve.

In RCTs, the use of convalescent plasma (OR 11.39, 95% CI 3.91 to 33.18, *p* < 0.001, low) showed significantly higher viral clearance rate compared to standard supportive therapy (Fig 4A). No active drug was associated with improved viral clearance rate within 14 days in mixed studies (Fig 4B).

### Time to viral clearance (days)

In RCTs, hydroxychloroquine was associated with the reduced time to viral clearance (Fig 4C). Meplazumab (MD −10.00, 95% CI −16.79 to −3.21, *p* = 0.045, very low), hydroxychloroquine (MD −4.01, 95% CI −5.28 to −2.73, *p* = 0.011, moderate), and favipiravir (MD −3.83, 95% CI −6.77 to −0.89, *p* = 0.009, high) were associated with shorter time to viral shedding compared with standard care (Fig 4D).

### Time to treatment initiation from symptom onset

The effect of the timing of hydroxychloroquine treatment initiation after the symptom onset (Fig 4E) was assessed. Treatment initiated after 14 days (MD −2.02, 95% CI −7.03 to 3.00) from symptom onset did not reduce the time to viral clearance compared to standard care.

### QTc prolongation

**Fig 5:**
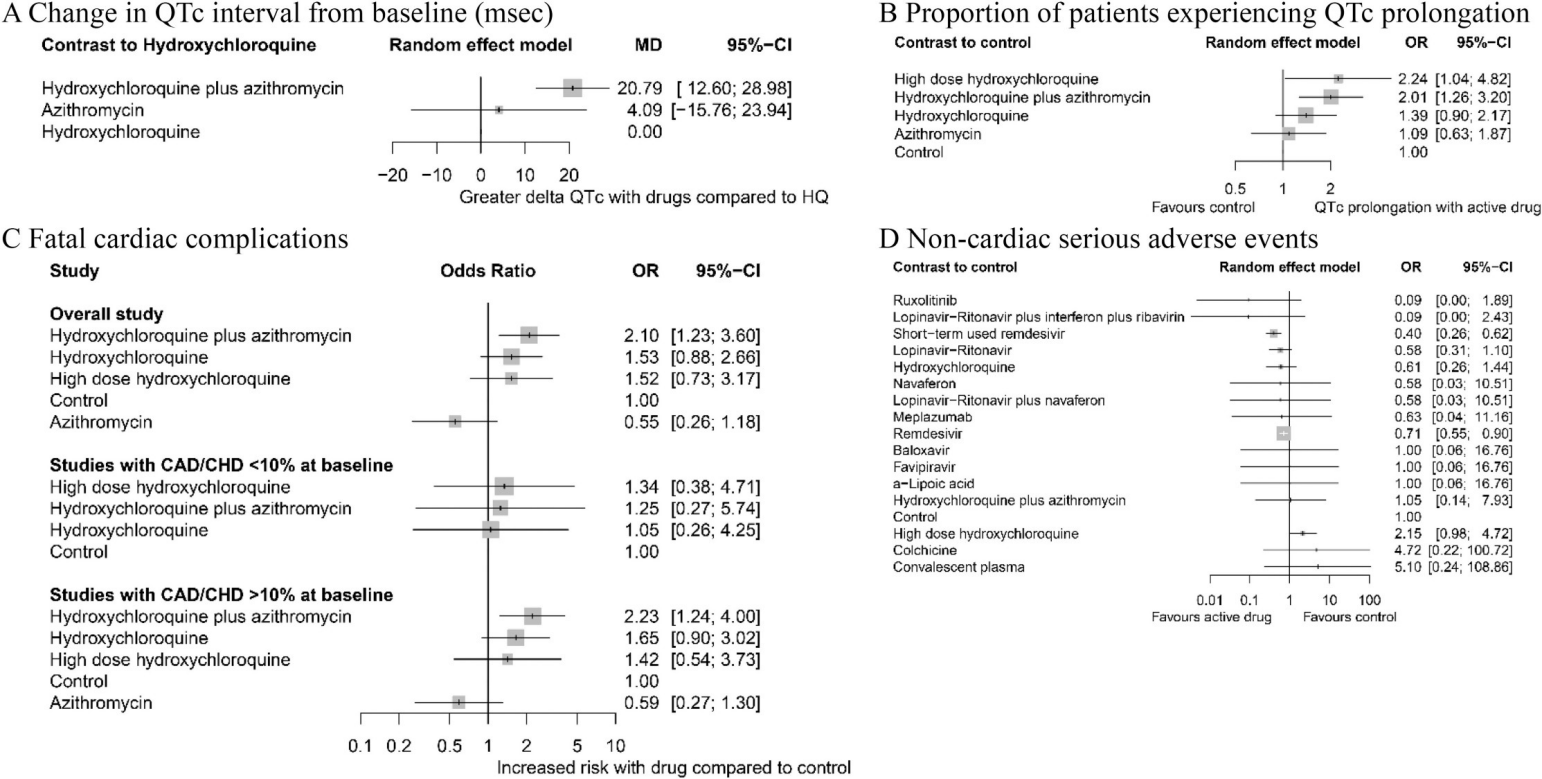
Network meta-analysis of safety of different pharmacological interventions. (A) Change in QTc interval (ΔQTc) from baseline (msec). (B) Proportion of patients experiencing QTc prolongation (>500 ms or ΔQTc >60 ms). (C) Fatal cardiac complication after hydroxychloroquine administration (torsades de pointes, cardiac arrest, and severe ventricular arrhythmia). (D) Noncardiac serious adverse events. Effect estimates are presented in OR and MD with 95% CI. Pharmacological agents are ranked by SUCRA value. CAD, coronary artery disease; CHD, congestive heart disease; CI, confidence interval; HQ, hydroxychloroquine; MD, mean difference; OR, odds ratio; SUCRA, surface under the cumulative ranking curve.

Compared to hydroxychloroquine monotherapy, the prolongation of QTc interval after treatment initiation was statistically significantly longer in the hydroxychloroquine plus azithromycin group (MD 20.79 ms, 95% CI 12.60 to 28.98, low) (Fig 5A). The proportion of patients experiencing QTc prolongation (defined by QTc interval >500 ms or ΔQTc >60 ms) was also significantly higher in the hydroxychloroquine plus azithromycin group and high-dose hydroxychloroquine group compared to the control group (OR 2.01, 95% CI 1.26 to 3.20, *p* = 0.003, low and OR 2.24, 95% CI 1.04 to 4.82, *p* = 0.039, moderate, respectively) (Fig 5B).

### Fatal cardiac complications (torsades de pointes, cardiac arrest, or severe ventricular arrhythmia)

The associations between fatal cardiac complications and hydroxychloroquine, azithromycin, or hydroxychloroquine plus azithromycin therapy were analyzed (Fig 5C). Overall, treatment with hydroxychloroquine plus azithromycin showed a significant association (OR 2.10, 95% CI 1.23 to 3.60, *p* = 0.007, low) while others did not. We further subdivided the included studies based on prevalence of coronary artery disease (CAD) and congestive heart disease (CHD) at baseline. In studies in which >10% of the baseline population had CAD/CHD, the risk of fatal cardiac complication was statistically significantly higher in patients receiving hydroxychloroquine plus azithromycin. In studies in which <10% of the baseline population had CAD/CHD, no notable difference in incidence of fatal heart complication was observed in any treatment group.

### Noncardiac serious adverse events

No agent or regimen was associated with noncardiac severe adverse events (Fig 5D). To the contrary, there was a protective effect, i.e., decreased rate of adverse events with both short-term (5 days regimen) and standard (10 days regimen) remdesivir compared to standard care. High-dose hydroxychloroquine (>600 mg/day) was more prone to risk of severe adverse events (i.e., nausea, vomiting, and diarrhea that required discontinuation of the treatment) compared to standard care, but this difference was not statistically significant.

### Subgroup and sensitivity analysis

The results of our subgroup and sensitivity analysis are reported in S4 Table. The assessments of other specific complications such as nausea/vomiting, diarrhea, hypoalbuminemia, anemia, leukopenia, lymphopenia, elevated AST/ALT, elevated CK, and increase total bilirubin are also presented in S4 Table.

## Discussion

To our knowledge, this is the first comprehensive NMA of pharmacological treatment for COVID-19. Our conclusions provide possible insights on the use of individualized treatment strategies based on clinical setting and severity. For moderate and severe patients hospitalized in non-ICU settings, corticosteroids, tocilizumab, anakinra, remdesivir, and convalescent plasma were associated with reduced risk of progression to severe pneumonia, admission to ICU, and/or mechanical ventilation. Among these agents, corticosteroids and remdesivir further showed survival benefit compared to standard care. For ICU-based critically ill patients, corticosteroids reduced mortality from RCT evidence; high-dose IVIG, ivermectin, and tocilizumab may be associated with reduced mortality when including observational data. Hydroxychloroquine, a topic of much debate, was not shown to reduce mortality rate or prevent progression to severe disease in our analysis.

We analyzed 47 active pharmacologic agents and their combinations in a large-scale analysis incorporating 49,569 COVID-19 patients. Our study included unpublished data to integrate recent investigations and avoid selection and publication bias, as done in previous studies [4–7]. We did not limit our inclusions to RCTs and incorporated observational studies as we deemed that, in this analysis, the inclusion of real-world evidence from nonrandomized studies has the potential to add validity to certain findings [13], provide additional information regarding low-to-moderate incidence adverse events [14–16], and improve the density of the network [14]. Many previous NMAs included observational studies with this rationale [14–16,36,37], but inclusion of observational studies to an NMA requires careful integration to avoid biases from these observational studies pervading the meta-analysis [38]; as such, we exclusively included cohort studies that adjusted for confounders through methods such as PSM, subgroup analyses, and/or regression modeling or established similarity in the baseline characteristics (*p* > 0.05) of the groups being compared so that such adjustments are not necessary or irrelevant.

Hydroxychloroquine was not shown to reduce mortality rate or progression to severe disease. Such accumulated empirical results on hydroxychloroquine is supported by a recent in vitro study that revealed chloroquine does not prevent SARS Coronavirus 2 (SARS-CoV-2) entry into human lung cells and the subsequent spread through pulmonary tissue [39]

The potential cardiotoxicity of hydroxychloroquine and azithromycin is a widely shared concern in treating COVID-19 with these medications. According to our quantitative synthesis, incidence of QT prolongation was significantly higher in the patients who received hydroxychloroquine plus azithromycin compared to those who received standard care (Fig 5B). In addition, this combination of hydroxychloroquine and azithromycin was also associated with increased rate of fatal cardiac complications such as torsades de pointes, cardiac arrest, and severe ventricular arrhythmia in the cardiac-impaired population with a pooled incidence of 12.27%; in comparison, the pooled fatal cardiac complications rate in healthy populations with preserved cardiac function was about 0.01%. It should also be noted that noncardiac serious adverse events (nausea, vomiting, and/or diarrhea requiring discontinuation of the medication) were more frequent in high-dose (>600 mg/day) hydroxychloroquine monotherapy compared to standard care (Fig 5D), but the difference was not statistically significant. Strict monitoring should be implemented in all patients receiving hydroxychloroquine with or without azithromycin to maintain a tolerable safety margin.

Corticosteroids, tocilizumab (monoclonal IL-6 receptor antibody), anakinra (IL-1 receptor antagonist), and IVIG were associated with significantly reduced mortality in COVID-19 patients (Fig 3A–3D). Corticosteroids have been widely used in managing inflammation [40–42]. Other 3 drugs are also known anti-inflammatory agents that have been conventionally used in hyperimmune or autoimmune conditions; tocilizumab and anakinra have been used for the management of severe rheumatoid arthritis [43,44] and juvenile idiopathic arthritis [45–47]; IVIG was used for management of Kawasaki disease [18,48], inflammatory muscle diseases [49,50], and sepsis [51]. As there is accumulating evidence for an hyperimmune response characterized by the release of pro-inflammatory cytokines in severe and deceased COVID-19 patients [52–56], suppression of the inflammatory response and potential cytokine storm with immune-modulatory therapies was proposed as a potential therapeutic target; the results of this NMA support the efficacy of these treatments. Effectiveness of anti-inflammatory agents (corticosteroids, tocilizumab, anakinra, and IVIG) and ineffectiveness of antiviral agents, except for remdesivir, in hospitalized COVID-19 patients suggest that the management of hyperreactivity of the host immune response is advantageous over targeting viral replication itself.

Although corticosteroids and tocilizumab were highlighted as potential therapeutic agents against COVID-19 here, there are safety concerns regarding superinfection and other side effects related to these agents. Somers and colleagues reported that COVID-19 patients who received tocilizumab experienced significantly more superinfection compared to standard care, but they also stated that increase in superinfection did not significantly impact fatality [57]. This may indicate that the vulnerability to infection induced by tocilizumab may be manageable, but caution is still warranted to avoid bacterial infection [58]. Long-term use of corticosteroids may also entail side effects as reported in the past [59]; however, this safety concern is not expected to be of huge concern as most regimens for COVID-19 do not last more than 10 days [60–63] and short-term use was sufficient. Indeed, the recent RECOVERY trial showed that oral or intravenous dexamethasone (at a dose of 6 mg once daily) for up to 10 days reduces 28-day mortality [63]. These results regarding corticosteroids may have the farthest-reaching effect during this global pandemic, as they are available worldwide at relatively low cost.

### Limitations

Our study has several limitations. First, some of the results were derived from a single study (i.e., Ruxolitinib) or studies with high RoB. To account for such weakness in evidence, we assessed the certainty of evidence for each outcome using the GRADE framework as summarized in Table 2. Second, for certain treatment agents, many articles have been published among which only one or few have been included in our analysis (e.g., convalescent plasma). This is because we prospectively collected studies that adhered to predefined inclusion criteria, and studies that did not adequately account for confounding or those prone to significant bias were filtered out. The excluded studies are listed and described in S5 Table with reasons for exclusion. Third, we included observational studies and unpublished data. While such inclusions may introduce biases into the final analysis, we judged the benefits overweigh the risks for reasons we mentioned in methods. Furthermore, we attempted to minimize biases by exclusively including observational studies that accounted for potential confounders and further conducted sensitivity analyses in which the same analysis was performed using only RCTs. Lastly, some of the results derived from this NMA lacks the support of pairwise meta-analysis. However, the methodological power of NMA is credible as empirical evidence supported that NMAs were 20% more likely to provide stronger evidence against the null hypothesis than conventional pairwise meta-analyses [64]. Accordingly, our NMA can offer meaningful implications for guiding management of COVID-19 until future studies build up stronger evidence.

The gradient of evidence levels analyzed in this review may assist the decision-making of clinicians and policymakers. Although numerous studies reported consistent results on beneficial effect of anti-inflammatory agents, the certainty of evidence for these agents are either low or very low because conclusions on tocilizumab, anakinra, and IVIG are based on observational studies. RCTs on these anti-inflammatory agents are required to confirm these findings and increase the level of evidence.

## Conclusions

Corticosteroids and remdesivir may effectively improve clinical outcomes of COVID-19 as shown in RCTs; there is evidence of associations for other agents from the observational data that tocilizumab, anakinra, itolizumab, IVIG, and convalescent plasma may also provide clinical benefits. Hydroxychloroquine was not associated with improved clinical outcomes for COVID-19, while posing both cardiac and noncardiac safety risks and warrant appropriate patient selection. Only 29% of current evidence on pharmacological management of COVID-19 is on moderate/high evidence certainty and can therefore be confidently incorporated into practice and policy.

## Supporting information

S1 PRISMA Checklist PRISMA NMA checklist of items to include when reporting a systematic review involving a network meta-analysis. (DOCX)

S1 Table PICOS (participants, interventions, comparisons, outcomes, study design) and study characteristics of include studies. (DOCX)

S2 Table Risk of Bias, NOS, and Jadad evaluations of individual studies. (DOCX)

S3 Table Bias analyses for outcomes (heterogeneity, inconsistency, publication bias, etc.). (DOCX)

S4 Table Subgroup and sensitivity analyses. (DOCX)

S5 Table Excluded studies. (DOCX)

S1 Text Search strategy. (DOCX)

S2 Text Appendix method descriptions. (DOCX)

## Abbreviations

AGA: American Gastroenterological Association
CAD: coronary artery disease
CHD: congestive heart disease
CI: confidence interval
COVID-19: Coronavirus Disease 2019
GRADE: Grading of Recommendations Assessment, Development, and Evaluation
ICU: intensive care unit
IVIG: intravenous immunoglobulin
MD: mean difference
NMA: network meta-analysis
NOS: Newcastle-Ottawa Scale
OR: odds ratio
PICOS: participants, interventions, comparisons, outcomes, and study design
PRISMA: Preferred Reporting Items for Systematic Reviews and Meta-Analyses
PSM: propensity score matching
RCT: randomized controlled trial
RoB: risk of bias
RoB2: Risk of Bias 2
ROBINS-I: Risk of Bias in Nonrandomized Studies of Interventions
SARS-CoV-2: SARS Coronavirus 2
SUCRA: surface under the cumulative ranking curve
