# Supplementary material for: Comparative efficacy and safety of pharmacological interventions for the treatment of COVID-19: A systematic review and network meta-analysis
Source: PLoS Med. 2020 Dec 30;17(12):e1003501. doi: 10.1371/journal.pmed.1003501 (PMC7794037; doi:10.1371/journal.pmed.1003501)
Supplement: S1 Table — (DOCX) [file pmed.1003501.s002.docx]

S1 Table. PICOS (participants, interventions, comparisons, outcomes, study design) and study characteristics of include studies

**Study characteristics of include studies in treatment analysis**

| **#** | **Author (year)** | **study design** | **number of participants in each arm** | **population (age, sex, and other distinctive characteristics)** | **Severities** | **dosage/regimen** | **timing of initiation of admission/ treatment/**  **randomization from symptom onset**  **(or closest date)** | **duration of medication use**  **(median or mean)** | **follow-up duration** | **primary outcome** | **conclusion** |
| --- | --- | --- | --- | --- | --- | --- | --- | --- | --- | --- | --- |
| 1 | Rosenberg et al.^1^  (2020) | RS | Total: 1438 -HQ plus AZ: 735 -HQ alone: 271 -AZ alone: 211 -Control (not received any): 211 | -median age 63 years -male 59.7% | - Patients with whole spectrum of severities are included for analysis.   - Severities were adjusted when primary outcome was analyzed. | -HQ: 400mg for 90.3% of participants/70.4% twice a day  -AZ: 500mg for 92% of participants/IV (50.9%)/once a day (75.4%) | HQ was initiated at a median of 1 day (Q1-Q3, 1-2) following admission and AZ was given at a median of 0 days (Q1-Q3, 0-1) | N/A | - 14 days of sampling period  - last f/u April 24, 2020 | in-hospital mortality | No benefit |
| 2 | Borba et al.^2^  (2020) | RCT | Total: 81 -high-dosage CQ: 41 -low-dosage CQ: 40 | -mean age 51.1 years -male 75.3% | Critical | -high-dosage CQ: 600mg CQ twice daily for 10 days (PO) -low-dosage CQ: 450mg twice daily on day 1 and once daily for 4 days(PO) | N/A | -high-dose CQ: 10 days -low-dose CQ: 9 days | - 28 days | lethality and safety by day 28 | No benefit |
| 3 | Geleris et al.^3^  (2020) | RS | Total: 1376 -HQ: 811 -no-HQ: 565 | HQ:  -age>60 63.4%  - 41.6% female  no HQ group -age>60 56.2% -45.7% Female | Moderate to severe | -HQ: 600 mg twice on day 1, then 400 mg daily for a median of 5 days (PO) -AZ: dose of 500 mg on day 1 and then 250 mg daily for 4 more days(Route of administration for AZ not specified) | N/A | 5 days | - median follow-up of 22.5 days | Decompensation (intubation or death) | No benefit |
| 4 | Bin Cao et al.^4^  (2020) | RCT | Total: 194  - LPV/R: 95  - standard: 99 | -median age 58  - 60.3% male | Severe | LPV/R (400 mg and 100 mg, orally; twice daily, plus standard care for 14 days. | The median interval time between symptom onset and randomization was 13 days (IQR, 11 to 16 days) | 14 days | - Protocol in Chinese, - Follow up duration not specified in the manuscriptbut presumably 28 days (RCT).- 28 days outcome were seen in protocol | Time to clinical improvement, defined as the time from randomization to an improvement of two points (from the status at randomization) on a seven-category ordinal scale or live discharge from the hospital, whichever came first. | No benefit |
| 5 | Ivan Fan-Ngai Hung et al.^5^  (2020) | RCT | Total: 127 - LPV/R + RBV  + IFN-1b : 86 - LPV/R : 41(Control) | - median age 52 - 54% male | Disease severity upon presentation was mild based on NEWS2 and SOFA scores | - 14-day combination of lopinavir 400 mg and ritonavir 100 mg every 12 h, RBV 400 mg every 12 h, and three doses of 8 million international units of interferon beta-1b on alternate days (combination group) or   - to 14 days of lopinavir 400 mg and ritonavir 100 mg every 12 h (control group).  *IFN-1b: subcutaenous Injection, *LPV/R, RBV: PO | 5 days | 14 days | - follow-up till discharge   - at least 30 days post treatment | time to negative conversion | Benefit in Early triple antiviral therapy |
| 6 | Yeming Wang et al.^6^  (2020) | RCT | Total: 237 - Remdesivir: 158  (3 did not start study  treatment,  5 received <5 days) - Placebo: 78 | remdesivir group - median age 66 - 56% male  Placebo group - median 64 - 51% men | Most patients were in category 3 of the six-point ordinal scale of clinical status at baseline. (hospital admission, requiring supplemental oxygen) | - Received either intravenous remdesivir (200 mg on day 1 followed by 100 mg on days 2–10 in single daily infusions) or  - The same volume of placebo infusions for a total of 10 days | 10 days | 10 days | 28 days | - Time to clinical improvement within 28 days after randomization.  - Clinical improvement: a two-point reduction in patients’ admission status on a six-point ordinal scale, or live discharge from the hospital, whichever came first. | No benefit but may help time to clinical improvement in early treated group |
| 7 | Qingxian Cai et al.^7^  (2020) | RCT) | Total: 80  - FPV + IFN-a1b: 35  - LPV/R + IFN-a1b: 45 | - median age 47  - 43.8% male- | non-severe | - FPV: 1600 mg twice daily on Day 1 and 600 mg twice daily on Days 2–14. (PO)- LPV/R: The dose was LPV 400 mg/R 100 mg twice daily. Both FPV and LPV/R were continued until the viral clearance was confirmed or until 14 d had passed (PO)- all participants received IFN-a1b 60 mg (Beijing Tri-Prime Gene Pharmaceutical Co., 30 lg per ampule) twice daily by aerosol inhalation. | 7 days | 14days | follow up for 14 days after treatment | - the time of viral clearance- the improvement rate of chest computed tomography (CT) scans on Day 14 after treatment | Benefit in FPV over LPV/R |
| 8 | D.R. Boulware et al^8^  (2020) | RCT | Total: 821  - HQ prophylaxis:414  - placebo:407 | HQ group  - median age 41  - 52.7% female  Placebo  - median age 40  - 50.6% female  - not hospitalized" | N/A | The dosing regimen for hydroxychloroquine was 800 mg (tablets) once, then 600 mg (3 tablets) 6 to 8 hours later, then 600 mg (3 tablets) daily for 4 more days for a total course of 5 days (19 tablets total). If participants had gastrointestinal upset, they were advised to divide the daily dose into two or three doses. | within 4 days after exposure | 5 days | primary outcome up to 14 days  surveys up to 4-6 weeks | incidence of either laboratory-  confirmed Covid-19 or illness compatible with Covid-19 within 14 days | No benefit |
| 9 | Francis Bessière et al.^9^  (2020) | RS | Total: 40  - HQ + AZ: 18  - HQ alone: 22 | - median age 68  - 80% male | presumably severe  (admitted ICU) | HQ (200mg, twice a day, for 10 days) with or without AZ (250mg, daily, for 5 days) | N/A | HQ: 10days  AZ: 5days | N/A | - increase in QTc intervals of more than 60 milliseconds (ΔQTc >60 milliseconds) compared with baseline or as a QTc of 500 milliseconds or greater- corrected QT (QTc) intervals greater than 460 milliseconds | may have harmful effect in terms of QT prolongation in AZ+ HQ combination |
| 10 | Nicholas J. Mercuro et al.^10^  (2020) | RS | Total: 90 - HQ alone: 3  7 -HQ + AZ : 5­­­3 | - mean age 60.1  -48.9% female | Supplemental oxygen required 71.1% Radiographic findings of pneumonia: 92.2% Intensive care at time of testing: 33.3% Mechanically ventilated at time of testing: 25.6% | The standard regimen was 400mg of HQ twice on day 1, then 400 mg daily on days 2 through 5. | Ninety patients were diagnosed with COVID-19 at a median (IQR) of 8 (5-12) days from the time of symptom onset. | 5 days | Median follow-up of 9 days | changes in QTc (ΔQTc) in the cohort and between groups receiving HQ and HQ plus AZ,   development of prolonged QTc interval to 500 milliseconds or more,   documented adverse effects | HQ with or without AZ may have harmful effect in terms of QT prolongation |
| 11 | Yueping Li et al.^11^  (2020) | RCT | Total:  - 86- control: 17  - LPV/R : 34  - Arbidol : 35 | - mean age 49.4  - 46.5% male- | non-severe (mild to moderate) | Group A (LPV/r group), 34 patients were administered lopinavir (200mg) boosted by ritonavir (50mg) (orally administer, twice daily, 500 mg, each time for 7-14 days).In group B (arbidol group), 35 patients were given arbidol (100mg) (orally administer, 200mg three times daily for 7 14 days). In group C(control group), 17 patients were not given any antiviral therapy | LPV/R:  3.5 days    Arbiodol:  6 days  control: 5 days | LPV/R  7-14 days  arbidol:  7-14 days | All three groups were followed for up to 21 days | rate of positive-to-negative conversion ofSARS-CoV-2 nucleic acid from the initiation of treatment to day 21, | LPV/R or arbidol monotherapy present little benefit for improvingthe clinical outcome |
| 12 | Wei Tang et al.^12^  (2020) | RCT | Total: 150  - SOC:75 - SOC+HQ : 75 | - mean age 46  - 55% male | Almost all (148; 99%) patients had mild to moderate covid-19, and only 2 (1%) patients had severe disease on screening. | Patients in the treatment group were given HQ within 24 hours after randomization, with a loading dose of 1200 mg daily for three days followed by a maintenance dose of 800 mg daily for the remaining days (total treatment duration was two weeks for patients with mild to moderate disease and three weeks for those with severe disease) | 16.6 days, | 14 days | 11 to 29 February 2020. | - negative conversion of SARS-CoV-2 by 28 days  - whether patients with severe covid-19 had clinical improvement by 28 days (as the trial was stopped early and only two patients with severe disease were enrolled, results on clinical improvement are not presented) | No benefit, adverse events higher in HQ group |
| 13 | Matthieu Mahévas et al.^13^  (2020) | RS | Total: 181  - HQ within 48 hours  of admission: 84  - HQ more than 48  hours after admission: 8  - No HQ (control): 89 | - median age: 60  - 72% male | severe(pneumonia who required oxygen but not intensive care) --> equivalent to WHO progression score 5 | HQ at a dose of 600 mg/day  (treatment group) and no HQ treatment  (control group) | Patients in the treatment group could start treatment within a grace period of 48 hours after admission.  The median interval between symptom onset and admission to hospital was 7 days | N/A | Median follow-up for surviving patients was 32.5 days; | Survival without transfer to the intensive care unit at day 21. | No benefit |
| 14 | Xiaofan Lu et al.^14^  (2020) | RS | Cohort Study (total: 244) - steroid: 151 - non- steroid: 93  Case-control study(PSM)  (total: 62)  - steroid (31) - non-steroid (31) | - median age 62  - 52% male | Critical | they converted all preparations to hydrocortisone equivalent doses (MP 1:5, dexamethasone 1:25)  (median hydrocortisone equivalent dosage 200 [range 100–800] mg/day) = daily average of 40mg MP (or equivalent) | N/A | N/A | presumably 28 days | 28-day mortality after admission | No benefit, higher dosage associated with elevated mortality risk |
| 15 | Xudan Chen et al.^15^  (2020) | RS | Baseline total: 284  Model 1: total treatment  regimen in all patients  (total: 280 )  - No treatment: 121  - Chloroquine only: 17  - Oseltamivir only: 13  - Arbidol only: 37  - LPV/R only : 60  - LPV/R+arbidol : 16  - Chloroquine + arbidol : 5  - oseltamivir + arbidol : 11  Model 2: antiviral  regimens in patients  with moderate  illness (total : 197)  No treatment:82  Chloroquine only: 13  Oseltamivir only :10  Arbidol only :27  LPV/R only :42  LPV/R  +Arbidol :10  Chloroquine+Arbidol :5  Oseltamivir+Arbidol :8 | - median age 48  - 46.1% male | mild (n= 22)  moderate  (n=199)  serious(n=40)  critical illness(n=6)  mostly moderate | N/A | 4 days | N/A | N/A | - Time to clearance since illness onset- Clearance rate 14 days after illness onset- Time to clearance since admission- Hospital stay | No benefit in antiviral agent |
| 16 | Ziyun Shao et al.^16^  (2020) | RS | Total: 325  (in final analysis)  - IVIG :174  - non-IVIG: 151 | - mean age 58  - 58% male- | Severe and Critically ill | Whether and when to use IVIG, dosageand course were decided by the doctors in charge.Mean dosage and range not specified in the paperbut can be divided into IVIG >15g/d, and <15g/d | N/A but time from admission to the beginning of IVIG treatment (>7 days and ≤7 days admission) were compared | N/A | The data collection period was from December2019 to March 2020, and the data cutoff date was April 3, 2020. | the 28-day and 60-day hospital mortality | Benefit in early administration of IVIG |
| 17 | Lisi Deng et al.^17^  (2020) | RS | Total: 33 - arbidol LPV/R: 16 - LPV/R alone: 17 | - mean age 44.56  - 52% male | - non-severe  -None corresponded to a lobe score of 0, minimal to a lobe score of 1, mild to a lobe score of 2, moderate to a lobe score of 3, and severe to a lobe score of 4. 9, 10 5 (31%) of 16 patients reached a lobe score of 4 in the combination group and 6(35%) of 17 in the monotherapy group in baseline characteristics, and there is no statistically significant differences between two groups | arbidol was given at a dose of 200 mg every 8 h and lopinavir (400 mg)/ritonavir (100 mg) orally every 12 h | N/A | 5–21 days | N/A | - negative conversion rate from the date of COVID-19 diagnosis (day7, day14)  - assessed whether the pneumonia was progressing or improving by chest CT (day7) | Benefit with ardidol and LPV/R combination over LPV/R monotherapy |
| 18 | Yin Wang et al.^18^ (2020) | RS | Total: 46- MP: 26- No- MP: 20 | - median age 54  - 57% male | Severe | - 1-2mg/kg/day MP (or equivalent)- while 26 of them received extra low-dose MP treatment with the dosage of 1–2 mg/kg/day for 5–7 days via intravenous injection. The specific dosage and duration of MP for the patients were determined according to the clinical manifestations, leukocyte count, lymphocyte count, inflammatory index, and lesion range.- Human immunoglobulin was usually used in the critical patients with a dosage of 10–20 g/day for 7–10 days as the pulse therapy. | N/A | MP: 5-7days | presumably 14 days (not specified in the script) | -SpO2,  -length of ICU hospitalization  -length of hospitalization  -images of chest CT scan on day 1, 7, and 14 after hospitalization | Benefit in early low-dose and short-term application ofMP was associated in terms of clinical outcomes |
| 19 | Moussa Saleh et al.^19^  (2020) | pro-  spective  observa  -tional  study | Total: 201 - Chloroquine: 10 - HQ 191 --> among them, HQ + AZ: 119 | - mean age 58.5  - 57.2% male | Severity not specified but refer to the criteria for the use of HQ may indicate severity of this cohort. | The treatment regimens for these medications were as follows: chloroquine 500 mg by mouth twice daily for one day followed by 500 mg by mouth once daily for four days, HQ 400 mg by mouth twice daily for one day followed by 200 mg by mouth twice daily for four days, and AZ 500 mg by mouth or intravenous daily for five days. | N/A | CQ: 4 days  HQ: 4days  AZ: five days | N/A | - QT prolongation resulting in TdP. | May prolong QT interval but no serious adverse events including Tdp and arrhythmogenic death observed |
| 20 | Ling Li et al^20^  (2020) | RCT | Total: 103  - convalescent plasma: 52  - control: 51 | Convalescent plasma  - age: 70  - 51.9% male  Control  - age:69  - 64.7% male | Severe or life threatening | The transfusion dose of COVID-19 convalescent plasma was  approximately 4 to 13 mL/kg of recipient body weight.  Convalescent plasma transfusion  was administered at approximately 10 mL for the first 15 minutes,  which was then increased to approximately 100 mL  per hour with close monitoring. Adjustments in the infusion  rates were allowed based on the patient’s risk for volume  overload and tolerance, at the discretion of the treating physicians. | 30 days | 1 day | final follow-up April 28, 2020. | clinical improvement within a 28-day period | No benefit |
| 21 | J.H. Beigel et al.^21^  (2020) | RCT | Total:1063  - Remdesivir: 538 -Placebo: 521 | - mean age 58.9  - male: 64.3% | Ordinal scale 4~8, majority on 5 | Remdesivir was administered intravenously as a 200-mg loading dose on day 1, followed by a 100-mg maintenance dose administered daily on days 2 through 10 or until hospital discharge or death. | 9 days | 10days or  until hospital discharge  or death. | 29 days | The primary outcome measure was the time to recovery, defined as the first day, during the 28 days after enrollment, on which a patient satisfied categories 1, 2, or 3 on the eight-category ordinal scale. | Benefit in terms of time to recovery |
| 22 | Jason D. Goldman et al.^22^  (2020) | RCT | Total: 397  - 5-day course: 200  - 10-day course: 197 | 5-day course:  - median age 61  -60% male  10-day course:  -median age 62  - 68% male | severe | - Received 200 mg of remdesivir on day 1, followed by 100 mg of remdesivir once daily for the subsequent 4 or 9 days. | 5-day group  - 8 days  10days group  - 8days | 5days and 10days | 28days in protocol, but majority of outcomes were measured within 14 days | clinical status assessed on day 14 on a 7-point ordinal scale | No significant difference |
| 23 | Fang Zheng et al.^23^  (2020) | RCT | Total: 89  - NVF: 30  - LPV/R + NVF: 30  - LPV/R :29 | NVF:  - mean age 50.1  -56.7% male  LPV/R + NVF:  - mean age 48.8  - 43% male-  LPV/R  - mean age 41.1  - 41.4% male- | moderate to severe | - The approved dosage of NVF: daily injection of 10 μg of protein in 1.0ml volume per vial. LPV/R (Kaletra) was manufactured by AbbVie Inc. and each tablet contained 200mg of Lopinavir and 50mg of Ritonavir.- The total daily doses (40 μg) of Novaferon were administered to patients twice per day by the oxygen-driven atomized inhalation for 15 minutes of 20 μg of Novaferon (2 x l ml vials) diluted with saline. For patients receiving LPV/R (Kaletra), 2 tablets were orally taken twice per day. | N/A | presumably 10 days or less, not specified in the script | N/A | viral clearance rates on day 3,6,9 after drug administration | Benefit in  NVF over  LPV/R |
| 24 | Ming Zhong et al.^24^  (2020) | RCT | Total: 17 - placebo: 9 -α-Lipoic acid: 8 | - median age 63  - 76.5%) male | Critical | ALA (1200 mg/d, intravenous infusion) once daily plus for 7 days on top of standard medical care.   Patients in the control group were treated with equal volume saline infusion on top of standard medical care for 7 days. | N/A | 7 days | up to day 30 after therapy | SOFA score | Benefit |
| 25 | Yan Lou et al.^25^  (2020) | RCT | Total: 29  - baloxavir: 10  - FPV :9  - control: 10 | - mean age 52.5  - 72.4% male | all spectrum | - Baloxavir marboxil group: baloxavir marboxil is used in combination with the existing antiviral treatment. The dose was 80 mg once a day orally on Day 1 and Day 4; for patients who are still positive in virologic test, they can be given again on Day 7, no more than three additional doses.  Favipiravir group: favipiravir was used in combination with the existing antiviral treatment. The first dose was 1600 mg or 2200mg orally, followed by 600 mg each time, three times a day, and the duration of administration was not more than 14 days; (3) Control group: Continue the existing antiviral treatment. - The existing antiviral treatment included LPV/R (400mg/100mg, bid, po.) or darunavir/cobicistat (800mg/150mg, qd, po.) and arbidol (200mg, tid, po.). - All of them were used in combination with interferon-α inhalation (100,000 iu, tid or qid). | 11.7 days | 14 days or less | N/A | - Percentage of subjects with viral negative by Day 14- the time from randomization to clinical improvement (seven-category ordinal scale or live discharge from the hospital, whichever came first.) | No benefit |
| 26 | Archana Ramireddy et al.^26^  (2020) | RS | Total: 98  - AZ only: 27  - AZ+HQ: 61 | - mean age 62.3  -61% male | all spectrum  but  62% were high risk (Tisdale Score)  The Elixhauser comorbidity index: 15.2 | - Though a majority (81.6%) of patients received at least a 5-day course of each medication, there were many patients who received a higher dose on day 1 compared to the dosage on days 2-5.- AZ was either given as PO or IV, and the dosage was either 500mg daily or 500mg on day 1 followed by 250mg daily on days 2-5. - For HQ, most patients (87.0%) received 400mg PO twice daily prior to receiving a dosage of 200mg PO twice daily on days 2-5. | N/A | A majority (81.6%) of patients  received  at least  a 5-day  course  of  each  medication | N/A | - maximum post-medication QTc ≥500 ms (if QRS <120 ms) or QTc ≥550 (if QRS ≥120 ms) - mean increase from baseline QTc to maximum post-medication QTc of ≥60 ms | may have harmful effect in combination (critical QTC interval prolongation, QT prolongation) |
| 27 | Daniel E. Freedberg et al.^27^  (2020) | RS | total 504(after PSM)  - famotidine:84 - no famotidine: 420 | - median age 65   - 44% female | Non-severe  (not specified in the script, but based on inclusion and exclusion criteria and oxygen requirement)  Famotidine and No famotidine group mostly on nasal cannula (45% vs 44%) | Famotidine users received a median 5.8 days of drug for a total median dose of 136 mg (63 – 233 mg). include oral, IV at any dose or duration | within 72 hours after PCR diagnosis | 5.8 days | 30 days of follow-up, or the close of the study on April 20, 2020 | Death or endotracheal intubation within 30 days of hospital admission (intubation-free survival). | Benefit |
| 28 | Joseph Magagnoli et al.^28^  (2020) | RS | total :368  -HQ:97  - HQ+AZ:113  - no HQ : 158 | -median age 70  -100% male | precise severity- Not available but presumably non-severe  HQ:  - SPO2: median 96%  HQ+AZ:  - SPO2: median 95%  No HQ  - SPO2: median 96% | N/A | N/A | N/A | Patients were followed from index until hospital discharge or death. | - discharge or death - Ventilation requirement- Result of hospitalization among patients requiring ventilation | No benefit, HQ alone rather increased overall mortality |
| 29 | Zhaowei Chen et al.^29^  (2020) | RCT | Total: 62 - control: 31 - HQ: 31 | -mean age 44.7  - 46.8% male | non-severe | - All received the standard treatment (oxygen therapy, antiviral agents, antibacterial agents, and immunoglobulin, with or without CSs), patients in the HQ treatment group received additional oral HQ (HQ sulfate tablets, Shanghai Pharma) 400 mg/d (200 mg/bid) between days 1 and 5  - Patients in the control group with the standard treatment only. | N/A | 5 days | N/A | - 5 days after enrollment or severe adverse reactions were observation end point. - Observed measured are changes in time to clinical recovery (TTCR) and clinical characteristics of patients | Benefit |
| 30 | Philip M. Carlucci et al.^30^  (2020) | RS | Total: 932- Zinc: 411- No zinc: 521 | Zinc  - mean age 63.19  - 35.7% female  No Zinc- mean age 61.83- 38.6% female- | Zinc- SPO2: 94%- RR: 20(19-24)No Zinc- SPO2 : 94%- RR: 20(18-24) | Patients were categorized based on their exposure to HQ (400 mg load followed by 200 mg twice daily for five days) and AZ (500 mg once daily) alone or with zinc sulfate (220 mg capsule containing 50 mg elemental zinc twice daily for five days) as treatment in addition to standard supportive care. | N/A | 5 days | N/A | - Duration of hospital stay, - Duration of mechanical ventilation, - Maximum oxygen flow rate,- Average oxygen flow rate, - Average FiO2, maximum FiO2, - Admission to the intensive care unit(ICU), - Duration of ICU stay, death/hospice, need for intubation, and dischargedestination | Benefit with HQ |
| 31 | Xiu Lan et al.^31^  (2020) | RS | Total: 73 - LPV/R : 34 - LPV/R + arbidol : 39 | LPV/R alone - mean age 59.5 - 32.4% men  LPV/R + arbidol - mean age 52.3 - 66.7% men | LPV + arbidol - ordinary 71.8% - Heavy 29.2%  LPV/R - Ordinary 61.8% - Heavy 38.2% | - LPV/R alone: LPV/R (400 mg and 100mg, orally orally) twice daily   - LPV/R + arbidol : lopinavir LPV/R (400 mg and 100mg, orally) twice daily combined with arbidol (200 mg, orally) three times a day. | LPV/R alone : 10.6 days  LPV + arbidol: 10.0 days | at least three days | N/A | The rate of cure(discharge) | No benefit compared to LPV/R alone |
| 32 | Mingxing Huang et al.^32^  (2020) | pro-  spective  observa-  tional  study | total: 373 - CQ: 197 - non-CQ: 176 | CQ group - the median age 42 - 51% female  non-CQ group - the median age 47.5 - 55% female | Majority: moderate (CQ:93% vs no-CQ: 89%) | - Chloroquine phosphate 500mg, orally, once (half dose) or twice (full dose) daily. | Guangdong - CQ: 7days - non-CQ 4 days  Hubei - CQ: 19days - non-CQ: 11 days | no more  than  10 days | 30 days | the time to undetectable viral RNA | Benefit |
| 33 | Jianfeng Wu et al.^33^  (2020) | RS | total: 1514  - no CS: 983  - CS: 531 | - median age 61  - 52.2% female- | severe or critical | - CS use definition: intravenous systemic CSs, including hydrocortisone, MP, and dexamethasone.- The dose of CSs was converted to MP-equivalent doses (1mg MP = 0.1875mg dexamethasone = 5mg hydrocortisone).- Daily average of 40mg IQR(40-60) MP (or equivalent) | Severe cases- 67.6% started CS use within 24 hours after being diagnosed as severe disease- The median initial time of CS use since being diagnosed as severe cases was 2.2 hourscritical case- 79.9% started CS use within 24 hours after being diagnosed as critical cases- The median initial time of CS use since being diagnosed as critical cases was 0.1 hours | severe cases: average 6 dayscritical cases: average 5 days | the data collected from Dec 26 to March 15, 2020  The final follow-up date was March 19th, 2020. | In-hospital mortality of severe/critical cases. | No Benefit |
| 34 | Min Seo kim et al.^34^  (2020) | RS | Moderate total :97  - HQ plus antibiotics: 22 - LPV/R plus antibiotics: 35 - Conservative: 40 | - mean age 37.9   - 64.4%female | moderate | Patients in the HQ group received 200mg HQ tablets twice daily, and patients in the LPV/R group received lopinavir 200mg/ritonavir 50mg tablets twice daily.   AZ, when indicated, was used for 3 days in each patient and given as 500mg tablets once daily. Cefixime, when indicated, was used until remission of pneumonia and was administered as 100mg tablets twice daily | LPV/R plus anti: 5.5 days  HQ plus anti: 6.1days  conservative: 4.6 days | LPV/R: 8.3 days + anti: AZ(4.2days), Cefixime(8.9days)  HQ: 8.9days + anti-AZ(3.4days), Cefixime(8.7days) | Until April 28, 2020 (n/a?)  beginning on Feb 28 | Time from treatment initiation to  - complete viral clearance (i.e. two consecutive negatives on PCR signified by Ct value ≥ 40) - probable viral clearance (Ct value ≥ 35),  - discharge - symptom resolution | Benefit in HQ + antibiotics |
| 35 | Chen Shi et a.^35^  (2020) | RS | Total: 42  - LMWH: 21  - control: 21 | LMWH group  - Median age 69.0  -62% male  Control group  -Median age 69.0  - 67% male | Severe | N/A | LMWH group- time from hospitalization to virus shedding after the onset of the COVID-19: 20 days  control group- time from hospitalization to virus shedding after the onset of the COVID-19 : 19 days | N/A | N/A | - Duration of negative conversion- length of hospital stay | No benefit in clinical 564improvement but may have benefit in reducing inflammatory response |
| 36 | Zhichao Feng et al.^36^  (2020) | RS | total: 564 No antiviral therapy: 31 Arbidol : 43 LPV/RTV : 71 IFN-a : 18 Arbidol + LPV/R : 34 LPV/R + IFN-a: 184 Arbidol + LPV/R + IFN-a: 137 | - the median age 47   - 50.4% male | severe:69  non-severe: 495 | Specific regimen N/A except the information that only patients in Changsha  received chloroquine therapy (500mg, bid).  protocol and medication dose were varied in different hospitals | N/A | N/A | Monitored up to March 15, 2020. | progression to severe COVID-19 pneumonia | no benefit in antivirals or Chloroquine |
| 37 | Huijie Bian et al.^37^  (2020) | RCT | Total : 28 -meplazumab :17  - control : 11 | Meplazumab group  - Median age 51  - 64.7% male   Control group - mean age 64 - 45.5% male | Treated group - common 4(23.5%) - severe 6(35.3%) - Critical 7 (41.2%)  Control group - common: 4(36.4) - severe 4(36.4%) - critical 3(27.3%) | 10mg meplazumab was administered on day 1, day 2 and day 5 by intravenous infusion within 60–90 min. Efficacy and safety were assessed at baseline, every day after day 1 to day 14, and every week thereafter up to day 28 or discharge. | N/A | 5 days in script | presumably until day 28 or discharge | The primary study endpoint was the virological clearance (i.e. negative conservation rate and time to negative) | Benefit |
| 38 | Shailendra 28  Singh et al.^38^  (2020) | RS | Total: 3372  HQ (Treatment group:1125)  non-HQ group (control group:2247)  after PSM, both group :910 | Treatment group  - mean age 62.17  - 53.96% male  control group  - mean age  62.66  - 52.94% male | N/A | N/A | N/A | N/A | N/A | 30-day mortality | No benefit |
| 39 | Andrew Ip et al.^39^  (2020) | RS | total 2512  HQ: 1914  - HQ+AZ: 1473  -HQ alone: 441  no HQ: 598  - no HQ and AZ:342  - AZ alone: 256  ICU patients:611  - TCZ 134(53 missing, 11 TCZ before ICU- exclusion)  -no TCZ: 413 | HQ vs No HQ- median age 64  - 62% male.  Use of TCZ (ICU admission)  - median age 67  - 72% male | moderate to critical | HQ: at least one dose, and at any point during hospitalization. Dosing and duration were at prescribers’ discretion. The majority of patients received 800 mg on day 1, and 400 mg on day 2-5 (80%, n=1533), followed by 200 mg TID (4%, n=71) and other (15%, n =299), and missing dosing information (1%, n=11). TCZ was administered as a single dose in 104 (78%), with the majority receiving 400 mg (96%), followed by 800 mg (1%), 8 mg/kg (1%), 4 mg/kg (1%), and missing dosing (1%) | self-reported onset of symptoms to hospitalization was 5 days | HQ:  Median 5 days.  TCZ: N/A | hospitalized from March 1 to April 22,2020  Follow-up until final study cut-off date of May 5, 2020 | death with follow-up through May 5, 2020 | Benefit in TCZ, not in HQ with or without AZ |
| 40 | Corrado Campochiaro et al^40^  (2020) | RS | Total: 65 - TCZ: 32 - SOC: 33 | TCZ: - median age 64 - 91% male  SOC: - median age 60 - 82% male | Severe | TCZ was administrated intravenously at a dose of 400 mg. A second dose of 400 mg of TCZ was given after 24 hours in case of respiratory worsening (defined as need to increase FiO2, to start Noninvasive Ventilation (NIV), or to start mechanical ventilation) after the first TCZ infusion. | N/A | 2 days or less | N/A | clinical status with a six-category ordinal scale, | No benefit |
| 41 | Yang Cao et al^41^  (2020) | RCT | Total: 41 - Control: 21  - Ruxolitinib: 20 | -median age 63 -41.5% female | Severe | the treatment group (group B), which received oral intake of ruxolitinib 5mg twice a day plus standard-of-care (SoC); the control group (group A), which was treated with placebo (100mg vitamin C) twice a day with SOC. | 20 days | N/A | Presumably 28 days | the time to clinical improvement, | No benefit associated with primary outcome but improvement in CT and lymphopenia |
| 42 | Thomas Huet et al^42^  (2020) | RS | Total: 96 - anakinra group: 52 - historical group: 44 | Anakinra group: - mean age 71 - 69% male  Historical group - mean age 71.1 - male 57% | Severe | Patients in the anakinra group received subcutaneous anakinra (Swedish Orphan Biovitrum, Stockholm, Sweden) at a dose of 100 mg twice daily for 72 h, followed by 100 mg daily for 7 days, in addition to the standard treatment.   The dose of anakinra was adapted to renal function and reduced to one daily injection of 100 mg for 72 h, followed by an injection every other day for the next 7 days, in patients under dialysis or with a glomerular filtration rate of less than 30 mL/min  Standard treatments in Groupe Hospitalier Paris Saint-Joseph at the time included oral hydroxychloroquine 600 mg/day for 10 days, oral azithromycin 250 mg/day for 5 days, and parenteral β-lactam antibiotics (intravenous ceftriaxone 1 g per day or intravenous amoxicillin 3 g per day) for 7 days, in the absence of their respective contraindications. All patients received thromboembolic prophylaxis. No oral CSs or vasopressors were used, but some patients received an intravenous bolus of MP (500 mg). Supportive care included low-flow oxygen therapy (≤6 L/min through low-flow nasal cannula) or high-flow oxygen therapy (>6 L/min with high-flow nasal cannula or face mask). None of the patients had invasive or non-invasive mechanical ventilation at baseline. | Anakinra:  8.4days Historical: 6.2 days | 10days | N/A | the composite of either need for admission to the ICU with invasive mechanical ventilation or death | Benefit |
| 43 | Ruggero Capra et al^43^  (2020) | RS | Total: 85 - TCZ: 62 - Control: 23 | - median age 65 - 75% male | Severe | All patients received hydroxychloroquine 400 mg daily and lopinavir 800 mg daily plus ritonavir 200 mg daily as standard care and were subsequently assisted with non-invasive or invasive oxygen therapy (from low flow nasal cannula to mechanical ventilation), according to their needs.  availability of the drug, 33 (53%) received 400 mg i.v once, whereas 27 (43.5%) received subcutaneous 324 mg once, after analyzing the pharmacodynamic aspects. The first two patients (3.5%) received 800 mg iv. | Included patients treated with TCZ within 4 days from hospital admission and control patients who were admitted to the hospital earlier than 4 days before TCZ availability. | presumably 1 day | from Feb 26 to April 2, 2020 | the survival rate in patients treated with TCZ and controls. | may have benefit if used early |
| 44 | Mingli Yuan et al^44^  (2020) | RS | Total:70(for matched) CS: 35 non-CS: 35 | CS group: - median age 48.1 - 49.6% male  non-CS - median age 47.7 - 40,0% male | non-severe | With matched population - maximum dosage of 50.6mg/day - initial dosage 43.5 mg/day | CS was initiated within a median of 8.3 days (IQR, 5.0–10 d) of the onset of illness, and they were initiated within a median of 1.9 days (IQR, 0–2.25 d) of hospital admission. | - median time to initiation from onset of illness 9.7 day - median time to initiation from admission 1.9 days - median duration of therapy 10.7 days | N/A | clinical and radiographic outcomes (progressing to severe cases, secondary infection, time for fever, hospital stay, duration of viral shedding after illness onset) | may have negative effect on lung injury recovery |
| 45 | Ana Fernández Cruz et al^45^  (2020) | RS | Total: 463 -Steroids: 396 -control: 67 | Steroid cohort - mean age 65.4 - 69.7% male  control cohort - mean age 68.1 - 61.2% male | Critical | 1-2mg/kg/day MP (or equivalent) | Steroid - Days from symptoms to therapy: 7.4 days - Days from onset of symptoms to steroids: 10.7 days  Control - Days from symptoms to therapy: 7.1 days | N/A | N/A | in-hospital mortality | may benefit in survival |
| 46 | Estela Moreno-García et al^46^  (2020) | RS | Total: 171 -TCZ: 77 -No TCZ: 94 | TCZ group: - mean age 61.5 - 68.8%male  no TCZ group: - mean age 61.4 - 62.7% male | severe | The dose was 400 mg/24h iv for patients with ≤75 kg and 600 mg/24h iv for those with >75 kg with the possibility to repeat the dose every 12h up to 3 doses in case of only partial response. However, due to the lack of evidence to support its efficacy, the ultimate decision about using TCZ was left to the judgement of the attending physician. | The mean (SD) time from symptoms onset to hospital admission in TCZ group was 6.5 (3.3) days while it was 5 (6.5) days in the control group. | presumably 3 days or less | Admitted from Feb 19 to April 16, 2020  until April 26,2020 | composite of the need of ICU admission or death whichever came first | Benefit in reducing ICU admissions and mortality |
| 47 | Emily C Somers^47^  (2020) | RS | A: Total: 154 - TCZ: 78 - no TCZ: 76  B: (TCZ: 49 vs no-TCZ:67)  C: (TCZ:78 vs no-TCZ 76) | mean age 56 34% female | Critically ill | The standard TCZ dose was 8 mg/kg (maximum 800 mg) x 1; additional doses were discouraged. | 47% are treated within 24 hours of intubation | presumably 1day | Median follow-up was 47 days | survival probability after intubation | Benefit in decreasing likelihood of death |
| 48 | Effat Davoudi-Monfared,et al^48^  (2020) | RCT | Total:81 -IFN group: 42 -control:39 | IFN group: -mean age 56.09 -52.38% male  Control group: -mean age 59.53 - 53.84% male | severe | Patients in the IFN group received IFN β-1a in addition to the standard of care. Each 44 micrograms/ml (12 million IU/ml) of interferon β-1a (ReciGen®, CinnaGen Co., Iran) was subcutaneously injected three times weekly for two consecutive weeks. The control group received only the standard of care. The standard of care (the hospital protocol) consisted of hydroxychloroquine (400 mg BD in first day and then 200 mg BD) plus LPV/R (400/100 mg BD) or atazanavir/ritonavir (300/100 mg daily) for 7-10 days. | IFN:  11.70 days  Control  9.31 days | 14 days | presumably until day 28 or discharge | time to reach clinical response. Clinical response was defined according to the six-category ordinal scale | May have benefit in discharge rate on day 14 and decreased 28-day mortality (no benefit in clinical response) |
| 49 | Benjamin Rossi et al^49^  (2020) | RS | Matched total: 168 - TCZ: 84 - no TCZ: 84 | matched TCZ: - mean age64.8 - 34.5% female  matched control -mean age:64.4 - 41.7% female | severe | single IV injection 400mg | Delay between first symptoms and admission: matched control(5.2days) vs matched TCZ(6.5days) | 1 day  (single dose) | Maximum follow up 28 days | a composite of all-cause mortality and invasive mechanical  ventilation (i.e. requiring tracheal intubation) | Benefit in improving survival without mechanical ventilation |
| 50 | Caleb P. Skipper et al^50^  (2020) | RCT | Total: 423  - HQ:212  - Placebo: 211 | HQ:  -median age 41  -58.0% female  placebo:  - median age:39  - 54.5% female | Mean symptom severity score (0-10):  - HQ: 4.1  - placebo: 4.2 | Hydroxychloroquine was prescribed at 800 mg (4 tablets) once, then 60mg (3 tablets) 6 to 8 hours later, then 600 mg (3 tablets) once daily for 4 more days (5 days in total) | Duration of antecedent symptoms:  - HQ: 40.6%  < 1d  - Placebo: 39.3% <1d | 5 days | 14 days | ordinal outcome by day 14 of not hospitalized, hospitalized, or  intensive care unit stay or death | No benefit |
| 51 | Joseph Miller et al^51^  (2020) | RCT | Total: 34  **Arm A** (those who have severe COVID-19 pneumonia):  - Auxora: 17  - SOC: 17  Total : 4  **Arm B** (who have critical COVID-19 pneumonia)  -Auxora:3  -SOC:1 | **Arm A:**  - Auxora:  mean age 59  41% male  -SOC:  mean age 61  56% male  **Arm B:**  - Auxora:  mean age 64  33% male  - SOC:  mean age 36  100% male | Severe and critical | Auxora was administered on three consecutive days as a 4-hours continuous IV infusion. The initial dose was 2.0 mg/kg (max 250 mg), and subsequent doses were 1.6 mg/kg (max 200 mg) at 24 and 48 hours. All patients received local standard of care, including antiviral agents, but investigational therapies and immunosuppressive medications were not permitted | **Arm A:**  - Auxora:  median 9 days  -SOC:  median 7 days  **Arm B:**  - Auxora:  median 11 days  - SOC:  median 13 days | 3 days | 30 days | Mortality at day 30 was evaluated as a safety outcome.  Efficacy outcome measures included recovery rate defined as the first day the patient satisfied criterion 6, 7, or 8 of the 8-point ordinal scale | Benefit |
| 52 | Marie Lecronier et al^52^  (2020) | RS | Total: 80  - SOC: 22  - LPV/R: 20  - HQ: 38 | SOC:  - median age 63  - 82% male  LPV/R  - median age 55  - 75% male  HQ:  - median age 59  - 82% male | critical | During the first period, each new patient admitted in our ICU was receiving, in addition to standard of care, LPV/R (400 mg twice daily, oral route down the nasogastric tube in syrup form) for 5 days as per our local disease control policies. During the second period, due to a LPV/R shortage, the local policy was changed to hydroxychloroquine (200 mg, twice a day, oral route) for each new patient admitted in our ICU. During the last period, no specific treatment was given besides the standard of care. | - SOC: 6 days  - LPV/R 5 days  - HQ: 3 days | refer to regimen | 28 days | treatment escalation occurring after day 1 after ICU admission until day 28 | No benefit |
| 53 | Andrey A. Ivashchenko et al^53^  (2020) | RCT | Total: 60  - AFV: 40  - SOC: 20 | AFV:  - mean age 51.8  - 52.5% male  SOC:  - mean age 48.6  - 45% female | moderate | receive either AFV 1600 mg BID on Day 1 followed by 600 mg BID on Days 2-14 (1600/600 mg), or AFV 1800 mg BID on Day 1  followed by 800 mg BID on Days 2-14 (1800/800 mg), or SOC | - AFV: ≤ 7 days, 52.5%  - SOC: ≤ 7 days, 65% | 14 days | 29 days | the elimination of SARS-CoV-2 by Day 10 | Benefit |
| 54 | Brian C. Nelson et al^54^  (2020) | RS | Propensity matched cohort total: 84  - Control: 42  - MP:42 | Control:  - median age 62  - 71% male  MP:  - median age 60  - 67% male | Critical | MP one mg/kg/day with a max dose of 80mg/day, with recommended duration of five days, although the course could be extended at the discretion of the treating physician. | control: 6 days  MP: 6 days | depending on the patients, refer to regimen | 60 days | ventilator-free days at hospital day 28 | Benefit |
| 55 | Oriol Mitjà et al^55^  (2020) | RCT | Total: 293  - Control: 157  - HQ : 136 | Control:  - mean age 41.7  - 65.6% female  HQ:  - mean age 41.6  - 72.1% female | mild | HQ 800mg on day 1, followed by 400 mg once daily for six days) | median 3 days | 6 days | 28 days | reduction of viral RNA load in nasopharyngeal swabs at days 3,7 after treatment start | No benefit |
| 56 | Christiane Maria Prado Jeronimo et al^56^  (2020) | RCT | Total: 393  - placebo:199  MP: 194 | Placebo:  - mean age:57  -35.7% female  MP:  - mean age 54  - 35.1% female | moderate to critical | IV sodium succinate MP(0.5mg/kg), twice daily for 5 days, or placebo (saline solution). | Placebo:  median 13 days  MP:  median 13 days | 5 days | 28 days | 28-day mortality | No benefit |
| 57 | Susan A. Olende et al^57^  (2020) | RS  +  RCT | inverse probability weighting total: 1130  Remdesivir-cohort: 312  Non-remdesivir cohort: 818 | Remdesivir  - 40~64 years: 50%  - 59% male  Non-remdesivir  - 40~ 64 years: 50%  - 59% male | severe | SOC plus Remdesivir 200mg on day 1, followed by remdesivir 100mg daily on days 2-5 OR  SOC plus remdesivir 200mg on day 1, followed by remdesivir 100mg daily on days 2-10 | Duration of symptoms before baseline:  - Remdesivir:  median 8 days  - median 7 days | can be up to 10 days | 28 days | clinical status assessed by a 7-point ordinal scale on day 14 and all cause mortality at day 28 | Benefit |
| 58 | Samia Arshad et al^58^  (2020) | RS | Total: 2541  - neither medication: 409  - HQ alone:1202  - AZ alone: 147  - HQ + AZ: 783 | Total:  - mean age 64  - 51.1% male | all spectrum | Hydroxychloroquine was dosed as 400 mg twice daily for 2 doses on day 1, followed by 200 mg twice daily on  days 2–5. Azithromycin was dosed as 500 mg once daily on day 1  followed by 250 mg once daily for the next 4 days. The combination  of hydroxychloroquine + azithromycin was reserved for selected patients with severe COVID-19 and with minimal cardiac risk  factors | N/A | up to 5 days | up to 53 days  (median 28.5 days) | in-patient hospital mortality | Benefit |
| 59 | Noa Biran et al^59^  (2020) | RS | Matched population total: 630  - No TCZ: 420  - TCZ: 210 | No TCZ:  - median age 65  - 33% female  TCZ  - median age 62  - 26% female | Critical | Exposure to TCZ was defined as receipt of the drug as found in the electronic health record  The 400 mg IV dose of TCZ was chosen. A second dose of  TCZ was permitted at the point of worsening oxygenation (eg, increased oxygen [O2] requirement, high-flow O2) and before mechanical ventilation, with administration at the treating clinician’s discretion. | N/A | refer to regimen | Followed up until May 22 from March 1, 2020 | hospital-related  mortality | Benefit |
| 60 | the RECOVERY collaborative group^60^  (2020) | RCT | Total: 6425  Dexamethasone: 2104  Usual Care: 4321 | Dexamethasone:  - mean age 66.9  - 64% male  Usual care  - mean age 65.8  - 64% male | all spectrum | Received either the usual standard of care alone or  the usual standard of care plus oral or IV dexamethasone (at a dose of 6 mg once daily) for up to 10 days (or until hospital discharge if sooner) or to receive one of the other suitable and available treatments that were being evaluated in the trial. | -Dexamethasone: 8 days  - Usual care:  9 days | up to 10 days | 28 days | all-cause mortality  within 28 days after randomization | Benefit in population receiving invasive mechanical ventilation or oxygen alone |
| 61 | A.B. Cavalcanti et al^61^  (2020) | RCT | Total: 665  - HQ+ AZ: 217  - HQ: 221  - control:227 | HQ+AZ:  - mean age 49.6  - 56.7% male  HQ:  - mean age 51.3  - 64.3% male | mild to moderate | Patients were randomly assigned in a 1:1:1 ratio to receive standard care (control group), standard care plus hydroxychloroquine at a dose of  400 mg twice daily for 7 days (hydroxychloroquine-alone group), or standard care plus hydroxychloroquine at a dose of 400 mg twice daily plus  azithromycin at a dose of 500 mg once a day for 7 days. | median 7 days | up to 7 days | 15 days | clinical status at 15  days, evaluated with the use of a seven-level ordinal scale | No benefit |
| 62 | Q.MA et al^62^  (2020) | RS | Total:72  - CS: 47  - No CS: 25 | - mean age 60  - 53% male | severe or critical | The patients in the CS group receiving low-dose MP therapy for 3 days, in which 42 patients were given a dose of 40 mg/d, and 5 patients with shock were given 80 mg/d. The non-CS group patients did  not receive CS therapy. | mean 4.2 days | 3 days | N/A | hospital mortality,  hospital length of stay, and time of viral clearance | May have benefit in improving clinical symptoms |
| 63 | Zhiliang Hu et al^63^  (2020) | RS | Total: 72  CS: 28  - No CS: 44 | - median age 53  - 55.6% male | severe | The principle of this therapy is short course (within 1 week) and low dose (MP,  40 mg per day intravenously) application of CSs. Intravenous immunoglobulin (20 g per day for 3–5 days) was co-administered with CSs. The decision on initiation of  CS treatment and duration of this medicine was made  by the treating physicians. | median 5 days | up to 7 days | N/A | progression to severe illness. | may have benefit |
| 64 | Hassan Abolghasem et al^64^  (2020) | RS | Total: 189  Plasma: 115  Control: 74 | Plasma:  - mean age 54.41  - 58.3% male  Control  - mean age:56.83  - 50% male | Severe | The first 500 cc (one unit) plasma  was infused within four hours and if the patient did not show any improvement after 24 h, based on the decision of responsible physician,  another unit of plasma was administrated. | less than 7 days | refer to regimen | N/A | the patient survival and length of  hospital stay | Benefit |
| 65 | Jun Chen et al^65^  (2020) | RCT | Total: 30  - DRV/c:15  -Control:15 | DRV/c  - mean age: 51.5  - 60% male  Control group  - mean 42.9  - 60% male | all spectrum | Participants in experiment  group received 1 pill of DRV/c (a single-tablet regimen containing 800 mg of darunavir and 150 mg of cobicistat) per day  for 5 days, while participants in the control group did not receive oral antiviral drug | N/A | 5 days | 14 days | viral clearance rate at day 7 after  randomization | No benefit |
| 66 | Fabrizio Cantini et al^66^  (2020) | RS | Total: 191  - Baricitinib combined with LPV/R: 113  - HQ combined with LPV/R(control):78 | Baricitinib group  - median age 68  - 64.6% male  Control group  - median age 63  - 59% male | moderate | Baricitinib 4 mg/day was provided orally associated with  LPV/R tablets 250 mg/bid for 2 weeks | N/A | 14 days | 14 days | 2-week case  fatality rate | Benefit |
| 67 | WANG Jia-bo et al^67^  (2020) | RCT | Total:47  - CM: 24  - Control: 23 | CM:  - mean age 46.8  - 58.3% male  Control:  - mean age 51.4  - 51.4% male | all spectrum | Control:  antiviral treatment (alpha interferon  inhalation, 50 μg twice daily; and LPV/R,  400 mg and 100 mg twice daily, respectively) and the  other supportive treatments  Treatment:  each patient was given  the same treatments as in the control therapy plus Keguan-1 19.4 g twice daily. | Treatment group:  6.5 days  Control group:  8.0 days | 14 days | 28 days | the occurrence of  ARDS | Benefit |
| 68 | Sofia Ramiro et al^68^  (2020) | RS | Total: 172  Treated (glucocorticoid with or without TCZ): 86  control: 86 | Treated:  - mean age 67  - 79% male  Control:  - mean age 67  - 79% male | severe | The treatment protocol included two steps: (1) immediate treatment with MP (MP) 250mg intravenously on  day 1, followed by MP 80mg intravenously on days 2–5, and an  option for a 2-day extension if considered necessary and safe; (2)  escalation of immunosuppressive treatment with a monoclonal  antibody directed against the interleukin-6 receptor, TCZ  (TCZ), between day 2 and day 5 (single-dose TCZ, 8mg/kg body  weight intravenous, max 800mg). Criteria for escalation with  TCZ were lack of clinical improvement or worsening in respiratory status (assessed on the WHO scale). Criteria for a 2-day  extension of MP at day 5 were clear clinical improvement in respiratory status (≥1 stage improvement on the WHO scale)  but a partial decrease of biomarkers (CRP reduction less than  50%). | N/A | up to 7 days | N/A | discharge from the hospital or  improvement of at least two stages (compared with baseline;  whatever came first) on a WHO-endorsed 7-point ordinal scale | Benefit |
| 69 | Mario Karolyi et al^69^  (2020) | RS | Total:156  - No Treatment: 89  - HQ: 20  - LPV/R:47 | - mean age 72  - 41% female | severe | The HCQ was administered with a loading dose of  400mg twice daily on the first day, followed by 200mg  twice daily. Contraindications for HCQ were prolonged QT interval (defined as QTc >440ms), known  retinopathies, psoriasis and known glucose-6-phosphate dehydrogenase deficiency. The LPV/RTV dose  of 400mg/100mg was administered twice daily and  had the following contraindications: known human  immunodeficiency virus (HIV) infection, severe liver  disease and essential background medication with  potentially relevant interactions.  Duration of treatment was 5–10 days depending on disease severity and clinical progression | 7 days | 5-10 days | from 1 March  to 26 April 2020 | in-hospital mortality and ICU admission, length of stay  (LOS), viral clearance and side effects of treatment. | lower hospital mortality rate in LPV/R |
| 70 | N. Lian et al^70^  (2020) | RS | Total: 81  - UFV: 45  - Control: 36 | - mean age 60  - 45% male | moderate to severe | Patients in the UFV  group received UFV at 0.2 g three times a day. The levels of  ALT (alanine transaminase), AST (aspartate aminotransferase) and  creatinine after treatment were collected on the tenth day after  hospitalization. | N/A | N/A | at least 7 days | the rate of negative pharyngeal swab  tests for SARS-CoV-2 within 1 week after admission and the time  for virus to turn negative | No benefit |
| 71 | Shao-rui HAO et al^71^  (2020) | RS | Matched Total:  64  - IFN: 32  - Control: 32 | Interferon:  - mean age: 55.0  - 31.3% female  Control:  - mean age 61.5  - 31.3% female | all spectrum | In the IFN group, patients  received recombinant human IFN-α2b spray at a dosage of 100000 U, four times a day, for 7 days | median 6 days | 7 days | patients hospitalized from January 19 to February 19, 2020; the  final follow-up was March 3, 2020. | duration of SARS-CoV-2 virus shedding time  from the respiratory tract | No benefit |
| 72 | Lorenzo M. Canziani et al^72^  (2020) | RS | Total: 128  - TCZ: 64  - Controls: 64 | - mean age 64  - 73% male | presumably severe to critical | All admitted patients were treated with subcutaneous enoxaparin at prophylactic dosage, (ii)direct antivirals including first lopinavir 400 mg + ritonavir 100 mg  twice daily and subsequently, when this became unavailable, darunavir  800 mg + cobicistat 150 mg once a day were used in patients without  contraindications, (iii)hydroxychloroquine 200 mg twice a day was used in all patients who could take oral treatments and had no contraindications (i.e. prolonged QT interval, retinopathies, advanced  renal failure, known hypersensitivity). The same committee provided  the criteria that candidates had to fulfill to be eligible for TCZ:  (i) clinical worsening in the previous 24 h with increasing need for  oxygen or ventilatory support, (ii) absence of clinical or biochemical  signs of an active bacterial infection, (iii) elevated C reactive protein,  (iv) a higher risk for mortality at blood tests, based on the odds ratios  reported elsewhere and including lymphocyte count, ferritin,  creatine kinase, alanine aminotransferase, and D-dimer. Late intubation (over 24 h) was considered an exclusion criterion. The process of  eligibility evaluation lasted a maximum of 2 days  Patients in the TCZ group received one intravenous infusion of 8 mg/kg TCZ, followed by a second dose 24 h later if  no clinical worsening had occurred between infusions; 61/64 (95%)  patients in the TCZ group received 2 infusions. | mean 11 days | 2 days | 30 days | compare mortality rates  between the TCZ and control groups | No Benefit |
| 73 | Xiaowei Fang et al ^73^  (2020) | RS | Total: 78  General group total: 55  - CS:9  - No CS:46  Severe group total: 23  CS:16  No CS: 7 | General Group:  CS  - mean age 40.2  - 55.6% male  No CS  - mean age 39.9  - 47.8% male  Severe Group:  CS  - mean age 60.6  - 75% male  No CS  - mean age 54.3  - 71.4% male | non-severe/severe | Oral MP [median  hydrocortisone-equivalent dose, 237.5 mg/day (IQR, 206.3-300.0  mg/day)] was administered to 9 patients in the general group for  a median duration of 7 days (IQR, 5.5-8.0 days), while intravenous MP [median hydrocortisone-equivalent dose, 250.0  mg/day (IQR, 250.0-250.0 mg/day)] was administered to 16 patients in the severe group for a median duration of 4.5 days (IQR,3.5-5.8 days) | General Group(median):  CS: 7 days  No CS: 5 days  Severe group  CS: 6.39 days  No CS: 8.3 days | refer to regimen | N/A | SARS-CoV-2 viral clearance time | No delay in viral clearance |
| 74 | Ji-Won Kim et al^74^  (2020) | RS | Total: 65  - LPV/R: 31  - HQ: 34 | - mean age 64.3  - 38.5% male | mild to moderate | Lopinavir-ritonavir (400 and 100 mg, respectively) was  administered twice daily and hydroxychloroquine 400  mg was administered once daily. | median duration of symptoms before therapy:  median 7 days | more than 7 days | 6 weeks  (42 days) | time to negative conversion  of viral RNA in nasopharyngeal and oropharyngeal  swabs since the start of lopinavir-ritonavir or hydroxychloroquine therapy | Benefit in LPV/R compare to HQ |
| 75 | Emanuel Della-Torre et al^75^  (2020) | RS | Total: 56  - sarilumab: 28  - comparison group: 28 | - mean age 56  - 78% male | severe | Eligible patients received a single intravenous infusion of sarilumab 400mg in addition to local standard of care. Specifically, two single-dose prefilled syringes, each containing 200mg sarilumab, were added to 100mL 0.9% sodium chloride and  infused intravenously over 1hour. Treatment with sarilumab  was initiated on a compassionate indication within 24hours  from the fulfilment of inclusion criteria. All patients received  oral therapy with LPV/R, hydroxychloroquine and  a course of azithromycin as per local institutional standard of  care at the time of admission | median 7 days | refer to regimen | 28 days | Data on hemoglobin saturation, oxygen-support requirements,  PaO2  /FiO2  ratio, fever, laboratory values and adverse events  were recorded daily from enrolment through day 28 of hospitalization, intensive care unit (ICU) admission, discharge, or death,  whichever came first. T | No benefit |
| 76 | Francisco Sanz Herrero et al^76^  (2020) | RS | Total: 72  - No MP: 16  - MP 56 | - median age 67.5  - 62.5% male | severe | The dosage regimen was MP 250 mg administered intravenously daily on the first day followed by 40mg every 12 hours for 4 more days and stopped without tapering | median 6.8 days | 5 days | N/A | in-hospital all-cause mortality | Benefit |
| 77 | Yin-Qiu Huang et al^77^  (2020) | RCT | Total: 101  - RBV(RBV) + IIFN-a:33  - LPV/R + IFN-a: 36  - RBV+LPV/R+IFN-a:32 | - mean age:42.5  - 46% male | mild to moderate | RBV was given by intravenous injection at a loading dose of 2 g,  followed by oral doses of 400–600 mg every 8 h depending on  patients’ body weight, for 14 d.  . LPV/r was given orally at a dose of 400 mg/100 mg per  dose twice per day for 14 d. IFN-a was given by atomizing inhalation at a dose of 5 million U or 50 mg per dose twice a day for 14 d. All enrolled patients were hospitalized, and  medication was dispensed and administered by nurses punctually  and in the correct doses, and administered under camera  surveillance during hospitalization, so that patient compliance  was assured by visual confirmation. Missed doses were  administered within 2 h of the prescribed timing. All patients in  each cohort could additionally receive nasal cannula oxygen  therapy, non-steroidal anti-inflammatory drugs (NSAIDs), oral  or intravenous rehydration, electrolyte correction, anti-pyretics,  analgesics, and anti-emetic drugs as required by their clinical  conditions, as supportive treatment | median 4.0 days | refer to regimen | 28 days | the difference in the interval from  baseline (initiation of antiviral treatment) to SARS-CoV-2  nucleic acid negativity by nasopharyngeal swab among the  three antiviral treatment groups, with each of these two tests at  least 24 h apart | No benefit |
| 78 | Nan Wang et al^78^  (2020) | RS | Total: 381  IFN+LPV/R:83  LPV/R: 122  IFN + UFV: 94  UFV: 82 | - median age 50  - female 47.1% | all spectrum – but mostly moderate to critical | N/A | 6 days | N/A | January 15 through March 31  to  final follow-up was May 22,2020 | In-hospital mortality | early treatment with IFN-a2b was associated with reduced in-hospital mortality, whereas no significant  benefit was associated with IFN-a2b use in moderately ill  patients. |
| 79 | Song Tong et al^79^  (2020) | RS | Total: 115  - Control group:71  - RBV group: 44 | Control group:  - mean age 55.1  - 60.6% male  RBV group:  - mean age 54.6  - 43.2% male | severe | The experimental group comprised patients who received intravenous RBV 500 mg every 12 hours (Zhengzhou Cheuk-fung pharmaceutical Co., Ltd., Xinzheng City, Zhengzhou, China), whereas  those who did not receive RBV therapy formed the control  group. The control group received no other potential antiviral  treatments. All patients received appropriate antimicrobial therapy  and supportive care. | In the treatment  group, RBV was initiated within a median of 4 days (range 1–  12 days) of the SARS-CoV-2 diagnosis and within a median of 8  days (range 1–18 days) from symptom onset. | N/A | N/A | (i) the negative conversion  time for SARS-CoV-2 RT-PCR test and (ii) mortality rate in the two  groups. | No Benefit |
| 80 | Roberto Rossott et al^80^  (2020) | RS | Total: 222  - TCZ: 74  - SOC: 148 | TCZ:  - median age 59  - 82.4% male  SOC:  - median age 59  - 81.1% male | severe and critical | TCZ dose was 8 mg/kg infused  over 60 min (maximum dose of 800 mg); a second dose would be  administered after 12 h in case of fever persistence | TCZ:  median 7 days  SOC:  median 6 days | refer to regimen | up to 40 days | survival and hospital discharge | May have benefit |
| 81 | Christoph D. Spinner et al^81^  (2020) | RS | Total: 584  - 10-day remdesivir: 193  - 5-day remdesivir: 191  - SOC: 200 | 10-day remdesivir:  - median age:56  - 61% male  5-day remdesivir:  - median age 58  - 60% male  SOC:  - median age 57  - 63% male | moderate | All patients randomized to a remdesivir group received  200 mg of remdesivir intravenously on day 1, followed  by 100 mg of remdesivir once daily for the subsequent days,  infused over 30 to 60 minutes. Remdesivir treatment was to  be discontinued in any patient experiencing severe elevations in liver enzymes or decreases in estimated creatinine  clearance to less than 30 mL/min ( | Duration of symptoms before first dose of remdesivir:  - 10days Remdesivir:  - 5 days Remdesivir:  - SOC | 5 days, 10 days | The date of final follow-up was May 20, 2020. | the distribution of clinical status assessed on the 7-point ordinal scale on study day  11 | 5-day course remdesivir may have benefit |
| 82 | Giacomo De Luca et al^82^  (2020) | pro-  spective cohort study | Total: 39  - mavrilimumab group: 13  - Control group: 26 | Mavrilimumab group:  - mean age 57  - 92% male  Control group:  - mean age 60  - 65%male | severe | All patients who were  admitted to hospital with COVID-19 pneumonia received  on admission treatment with oral hydroxychloroquine  (200 mg twice a day), intravenous azithromycin (500 mg  once daily until patient tested negative for urine antigen for  Legionella pneumophila), oral lopinavir–ritonavir (400 mg  and 100 mg, respectively, twice a day), and respiratory  support with supplemental oxygen or non-invasive ventilation with continuous positive airway pressure (with a  positive end expiratory pressure of 10 cm of water). Mavrilimumab (provided by Kiniksa Pharmaceuticals, Lexington, MA, USA) was administered intravenously as a single dose of 6 mg/kg. | Duration of hospital stay before enrollment (median):  - Mavrilimumab group: 2 days  - Control: 1 day | refer to regimen | 28 days | time to clinical improvement (defined as  improvement of two or more points on the seven-point ordinal scale of clinical status). | Benefit |
| 83 | Giovanni Guaraldi et al^83^  (2020) | RS | Total: 544  - TCZ + SOC: 179  - SOC: 365 | TCZ + SOC:  - median age 64  - 71% male | severe | All patients were treated with the  standard of care (ie, supplemental oxygen, hydroxychloroquine, azithromycin, antiretrovirals, and low molecular weight heparin), and a non-randomly selected subset of patients also received TCZ. TCZ was given  either intravenously at 8 mg/kg bodyweight (up to a maximum of 800 mg) in two infusions, 12 h apart, or  subcutaneously at 162 mg administered in two simultaneous doses, one in each thigh (ie, 324 mg in total), when the  intravenous formulation was unavailable. | (median):  TCZ+SOC: 7 days  SOC: 5 days | at least 1 day | maximum 18 days | a composite of invasive mechanical ventilation  or death | Benefit |
| 84 | Anahita Sadeghi et al^84^  (2020) | RCT | Total:66  - sofosbuvir/daclatasvir. (SOF/DCV):33  - Control: 33 | - median age 58 – 52% male | moderate to severe | All patients received standard care according to the national Iranian  COVID-19 treatment guidelines which at the time of the study was  hydroxychloroquine 200 mg twice daily with or without LPV/R  200 mg/50 mg twice daily. The treatment arm received a single daily oral tablet containing 400 mg sofosbuvir and 60 mg daclatasvir (Sovodak, Rojan Pharma, Tehran, Iran) in addition to standard care for 14 days. | 8 day or less | 14 days | 14 days | s clinical recovery within 14 days of enrollment. Clinical recovery was defined as normalization of fever (37.2C),  respiratory rate (24/min) and oxygen saturation (94%) without supplementary oxygen therapy sustained for at least 24 h. | Benefit |
| 85 | Hamideh Abbaspour Kasgari et al^85^  (2020) | RCT | Total: 48  - SOF/DCV/RBV: 24  - control: 24 | SOF/DCV/RBV  - median age 45  - 46% male  Control  - median age 60  - 29% male | moderate | he intervention group received the combined single pill once-daily regimen of sofosbuvir/daclatasvir at a dose of 400/60 mg  (Sovodak, Fanavaran Rojan Mohaghegh Daru Co, Tehran, Iran) and RBV  600 mg twice daily. The control group received hydroxychloroquine  (400 mg single dose) and LPV/R (400/100 mg twice daily), with  or without RBV (600 mg twice daily), according to the national recommendation at the time of the study. | SOF/DCV/RBV:  median 5 days  control:  median 5 days | daily, duration not specified | 14 days | length of hospital stays. | May have benefit |
| 86 | Spyridon G. Deftereos et al^86^  (2020) | RCT | Total: 105  - control: 50  - Colchicine: 55 | - median age 64  - 58.1% male | non-critical | Patients who were  randomized to the intervention arm received a loading dose of colchicine followed by maintenance  daily dosage. The loading dose consisted of 1.5 mg of colchicine followed by 0.5 mg of colchicine 60  minutes later if no adverse gastrointestinal effects were observed. In the case of azithromycin  coadministration, a single 1.0-mg loading dose of colchicine was administered. The maintenance  dosage was 0.5 mg colchicine twice daily (reduced to once daily among patients with body weight  <60 kg) until hospital discharge or a maximum of 21 days | control:  median 5 days  Colchicine:  median 3 days | refer to regimen | 21 days | the difference in  maximal high-sensitivity cardiac troponin (hs cTn) levels between the 2 groups and the time for  C-reactive protein to reach levels greater than 3 times the upper reference limit.  the time from baseline to clinical deterioration, defined as a 2-grade  increase on an ordinal clinical scale, based on the World Health Organization R&D Blueprint Ordinal  Clinical Scale | Benefit |
| 87 | Gholamali Eslami et al^87^  (2020) | Open-  label  parallel  trial | Total: 62  - SOF/DCV: 35  - RBV: 27 | SOF/DCV  - median age 62  - 49% male  RBV  - median age 60  - 52% male | severe | One arm received a single daily pill  containing 400 mg sofosbuvir and 60 mg daclatasvir (Sovodak, Fanavaran  Rojan Mohaghegh Daru Co, Tehran, Iran) and the other received 600 mg RBV (Bakhtar Biochemistry Co, Kermanshah, Iran) every 12 h. Treatment was administered during admission for a maximum of 14 days. In addition, both arms received the national standard treatment protocol, which was at the time LPV/R 200/50 mg, two tablets every 12 h during admission for a maximum of 5 days, and hydroxychloroquine 400 mg single  dose on admission | N/A | up to 14 days | 21 days | the time from starting the trial  medications until discharge from hospital | Benefit |
| 88 | Eric Salazar et al^88^  (2020) | RS | Total: 243  - control:158  - Case: 85 | - mean age  50~50:27.8%  (majority)  - 55.7% male | severe to critical | Patients were transfused with one or two units of COVID-19 convalescent plasma. Criteria for a second transfusion included, but were not limited to: worsening imaging findings (X-ray/CT) thought to be due to COVID-19; increasing oxygenation requirement thought to be due to COVID-19; worsening  PaO2/FiO2 ratio thought to be due to COVID-19; worsening hemodynamic status thought be due to  COVID-19; or new end-organ failure thought to be due to COVID-19; or BMI >30 kg/m2  . As plasma  inventory was limited early in the study; a second unit was not always available. | N/A | refer to regimen | 28 days | mortality within 28 days | Benefit in early phase |
| 89 | Timothee Klopfenstein et al^89^  (2020) | RS | Total: 206  - TCZ: 30  - control: 176 | TCZ:  - mean age 75.6  - 70% male  control:  - mean age: 74.3  - 59.1% male | severe to critical | TCZ (8mg/kg per dose, 1 or 2 doses) | TCZ:  median 11.7 days  Control:  median 6.5 days | refer to regimen | N/A | mortality and/or IMV requirement | Benefit |
| 90 | Yang Li et al^90^  (2020) | RS | Total: 68  - Early-start group (CS): 47  - Control group: 21 | Early Start group:  - mean 57.6  63.8% male  Control group  - mean 59.0  - 57.1% male | non-critical | Therapeutic treatment usually  consisted of 40–80 mg/d (0.75–1.5 mg/kg/d) of MP for 3 days, then was tapered to 20 mg/d,  with a total treatment period of less than 7 days. | radiographic progression with a median time of 7 days from the onset of illness | maximum 7 days | last follow up:  26 March 2020 | the proportion of patients  requiring invasive mechanical ventilation | Benefit |
| 91 | Oriol Mitjà et al^91^  (2020) | RCT | Total: 2314  - Control arm: 1198  - Intervention arm:1116 | Control arm:  - mean age 48.7  - 73% female  Intervention arm:  - mean age 48.6  - 72.8% female | No severity/prevention trial | the intervention arm received HCQ  (Dolquine® 127 ) 800 mg on day 1, followed by 400 mg once daily for six days. | control:  - median 4 days  intervention:  - median 4 days | 6 days | 28 days | onset of a confirmed Covid-19 episode, defined as symptomatic illness (at  150 least one of the following symptoms: fever, cough, difficulty breathing, myalgia, headache, sore throat,  151 new olfactory and taste disorder(s), or diarrhea) and a positive SARS-CoV-2 RT-PCR test | No benefit |
| 92 | Maria IF Lopes et al^92^  (2020) | RCT | Total: 35  - placebo:18  - colchicine: 17 | Placebo group  - mean age 53.5  - 27.8% male  Colchicine group  - mean age 52.9  - 52.9% male | moderate to severe | Patients of the intervention arm received colchicine 0.5 mg thrice daily for 5 days, then  0.5 mg twice daily for 5 days; if body weight ≥ 80 kg, the first dose was 1.0 mg. Whether a  patient had chronic kidney disease, with glomerular filtration rate under 30 mL/min/1.73 m2  ,  colchicine dose was reduced to 0.25 mg thrice daily for 5 days, then 0.25 mg twice daily for 5  days, no matter the body weight.  All participants received the institutional treatment for COVID-19 with azithromycin  500 mg once daily for up to 7 days, hydroxychloroquine 400 mg twice daily for 2 days, then  400 mg once daily for up to 8 days and unfractionated heparin 5000 UI thrice daily until the end  of hospitalization. MP 0.5 mg/kg/day for 5 days could be added if the need for supplemental oxygen was 6 L/min or more. | Placebo:  - time of symptoms to baseline: median 7 days  Colchicine group:  - symptoms to baseline:  median 9 days | 10 days | 26 days | clinical parameters, such as the time of need for  supplemental oxygen; time of hospitalization; need for admission and length of stay in ICU; and  death rate and causes of mortality | Benefit |
| 93 | Cheng-Pin Chen^93^  (2020) | RCT | Total: 33  - HQ: 21  - SOC: 12 | - mean age 32.9  - 57.6% male | mild to moderate | HCQ administration plan was 400 mg b.i.d. on day 1 and 200 mg b.i.d. for 6 days on days 2–7. Both study group and comparison group received standard of care comprising supportive treatment for subjects with mild clinical COVID-19 symptoms and antimicrobial therapy for subjects presenting with moderate clinical COVID-19 symptoms. The treatment consisted of: (1) ceftriaxone 2 g daily for 7 days ± azithromycin 500 mg on day 1 and 250 mg on days 2–5; or (2) levofloxacin 750 mg daily for 5 d; or (3) levofloxacin 500 mg daily; or (4) moxifloxacin 400 mg daily for 7–14 days for subjects allergic to ceftriaxone or azithromycin or according to physician discretion. | within 4 days of diagnosis | 6 days | 14 days | time to negative  rRT-PCR assessments from randomization up to 14 days. | No Benefit |
| 94 | Monil Majmundar et al^94^  (2020) | RS | Total: 205  - CS: 60  - No CS: 145 | - mean age 57.6  - 74.63% male | non-critical | Patients in the CS cohort received systemic CSs in the form of  MP (n=29, 48.33%), prednisone (n=10, 16.67%), hydrocortisone (n=1, 1.67%),  and dexamethasone (n=20, 33.33%). CS was commenced at a median of 2 days (IQR,  1-5) following admission, on a median dose of 80 mg of MP or its equivalent of  other systemic CSs per day (IQR, 60-107) for a median duration of 5 days (IQR, 4-7) | N/A | refer to regimen | final follow-up on 10th May, 2020 | the composite outcome of intensive care unit (ICU) transfer,  intubation, or death | Benefit |
| 95 | Luis Corral-Gudino et al^95^  (2020) | partially RCT | Total: 85  - preference MP arm: 22  - Control arm: 29  - MP arm: 34 | - mean age 69  - 58% male | moderate to severe | SOC plus intravenous MP [40mg/12h 3 days, then 20mg/12h 3  days]. | N/A | refer to regimen | 14 days | composite of death, admission to the intensive care unit (ICU) or  requirement of non-invasive ventilation (NIV) | Benefit |
| 96 | Emilie Sbidian et al^96^  (2020) | RS | Total: 4642  - HQ alone: 623  - HQ + AZ: 227  - Neither drug: 3792 | HQ alone:  - median age 63  - 66.3% male  HQ + AZ:  - median age 61  - 69.6% male  Neither drug:  - median age 69  - 57.1% male | moderate to severe | The suggested HCQ regimen was a loading  dose of 600 mg on day 1, followed by 400 mg daily for 9 additional days. AZI at a dose of 500 mg  on day 1 and then 250 mg daily for 4 more days in combination with HCQ was an additional suggested therapeutic option | N/A | refer to regimen | 28 days | all-cause 28-day mortality as a time-to-event endpoint under a  competing risks survival analysis framework | No benefit |
| 97 | Juliana Cepelowicz Rajter et al  (2020)^97^ | RS | Total:280  - Usual Care: 107  - IVM: 173 | - mean age 59.6  - 45.4% female | all spectrum | Patients in the Ivermectin group  received at least one oral dose of ivermectin at 200 micrograms/kilogram in addition to  usual clinical care. | N/A | refer to regimen | Follow up data for all outcomes was May 19, 2020 | all-cause in-hospital mortality | Benefit |
| 98 | Faiq I Gorial et al^98^  (2020) | Pilot  clinical  trial | Total: 87  - IVM: 16  - Control: 71 | - mean age 45.17  - 72% male | mild to moderate | Patients received IVM 200 Mcg single dose at the admission day as add on therapy to Iraqi  Ministry of Health protocol for treatment of mild to moderate COVID-19 [ HCQ 400mg BID for  the first day then 200mg BID for 5 days plus AZT 500mg single dose in the first day then 250mg  for 5 days. | less than 3 days from enrollment | refer to regimen | 23 days | percentage of the cured patients within 23 days | Benefit |
| 99 | Ooi Say Tat et al^99^  (2020) | RS | Total: 92  - Group A: 57  (HQ+LPV/R)  - Group B: 35  (HQ+LPV/R+CS) | Group A:  - median age 50  - 21% female  Group B  - median age 48  - 11% female | all spectrum | Treatment regimen included LPV/R 400/100 mg twice a day, hydroxychloroquine 400mg twice on day 1, followed by 200mg three times a day on days 2-5 and prednisolone. Dose of prednisolone varied from 30mg once a day to 40mg twice a day at the physician's discretion depending on severity of illness and patient's body weight. CSs use of 3 days or more for any medical indications was considered as adjunctive CSs. | group A: median 4 days  group B: median 5 days | refer to regimen | 14 days | composite of clinical progression or death | No benefit |
| 100 | Sultan Mehmood Kamran et al^100^  (2020) | RCT | Total: 500  Intervention (HQ): 349  Control: 151 | - mean age: 35.96  - 93.2% males  . | mild | Patients assigned to standard dose of HCQ  (400mg 12 hourly day 1 then 200mg 12 hourly for next 4 days) plus SOC were 349 while 151  patients received SOC comprising of Vit C, Vit D, and Zinc only (control group) | N/A | refer to regimen | 14 days | progression of disease |  |
| 101 | Arvind Gharbharan et al^101^  (2020) | RCT | Total:86  - SOC: 43  - Convalescent plasma (CP): 43 | SOC:  - median age 63  - 77% male  CP:  - median age: 61  - 67% male | non-critical | Patients were randomly assigned via a web-based system at a 1:1 ratio to the current standard  of care at each hospital with or without the addition of 300ml of CP, the standard volume  of one plasma unit produced by Sanquin Blood Supply, was administered intravenously on the  day of inclusion. Patients without a clinical response and a persistently positive RT-PCR could  receive a second plasma unit after five days. Off-label use of EMA-approved drugs (e.g.  chloroquine, azithromycin, LPV/R, TCZ, anakinra) as a treatment for  COVID-19 was allowed in hospitals were this was part of the standard of care. | SOC:  - median 11 days  CP:  - median 9 days | refer to regimen | maximum 60 days | overall mortality until discharge from the hospital or a maximum of 60 days after admission whichever came first. | may have benefit in recent symptom onset |
| 102 | Filippo Albani et al^102^  (2020) | RS | Total:1403  - No CS: 844  - CS: 559 | No CS: 844  - mean age 68.5  - 33.9% female  CS: 559  - mean age 68.7  34.5% female | all spectrum | Median equivalent doses of dexamethasone dose (cumulative dose/days of therapy) was  10 mg (1st-3rd quartile 4.5-20). Patients who were never admitted to ICU received  equivalent doses of dexamethasone of 8 mg (4-16.1). Duration of CS therapy  was longer in survivors than in patients who died: 6 (4-10) days vs 4 (1-7), respectively (p  <0.001). | N/A | refer to regimen | N/A | In-hospital mortality | No benefit |
| 103 | RECOVERTY Collaborative Group^103^  (2020) | RCT | Total: 4716  - HQ: 1561  - Usual Care:3155 | HQ:  - mean age 65.2  - 62% male  Usual Care:  - mean age 65.4  - 63% male | all spectrum | Eligible and consenting patients were assigned in a ratio of 2:1 to either usual standard of care or usual standard of care plus hydroxychloroquine or one of the other available treatment arms  using web-based simple (unstratified) randomization with allocation concealment. Patients allocated to hydroxychloroquine sulfate (200mg tablet containing 155mg base equivalent) received a loading dose of 4 tablets (800 mg) at zero and 6 hours, followed by 2 tablets (400 mg) starting at 12 hours after the initial dose and then every 12 hours for the next 9 days or until discharge (whichever occurred earlier). | median days since symptom onset: 9 days for both | refer to regimen | 28 days | 28 day mortality | No benefit |
| 104 | Javier Martínez-Sanz et al^104^  (2020) | RS | Total: 1229  - Control:  969  - TCZ:  260 | Control:  - median age 68  - 59 % male  TCZ:  - median age 65  - 73% male | all spectrum | Of the 1,229  patients, 260 (21%) received a median total dose of 600 mg (IQR 600–800 mg) of  TCZ. The first dose was administered at a median time of 4 (IQR 3 – 5) days from inpatient admission. | median 6 days | refer to regimen | N/A | the time from study baseline to death | Benefit |
| 105 | Lan Chen et al^105^  (2020) | RCT | total: 48  - chloroquine: 18  - Hydroxychloroquine: 18  - Control: 12 | Chloroquine:  - mean age: 45.22  - 61.11% female  Hydroxychloroquine:  - mean age: 45.67  - 55.56% female  control:  - mean age: 51.33  - 41.67% female | moderate | In the chloroquine group, patients received standard treatment and chloroquine  phosphate (Guangdong Zhongsheng Pharmaceutical Co., Ltd., Guangzhou, China)  orally at 1000 mg QD for the first day, then 500 mg QD for additional 9 days. In the  hydroxychloroquine group, patients received standard treatment plus oral  hydroxychloroquine sulphate (Shanghai Pharmaceutical Co., Ltd., Shanghai, China) at 200 mg BID for 10 days. | N/A | refer to regimen | 28 days | time to clinical recovery  (TTCR), defined as the number of days from randomization to clinical recovery. | may have benefit |
| 106 | Andrew Tsai et al^106^  (2020) | RS | Matched Total: 132  - TCZ: 66  - No TCZ: 66 | TCZ:  - mean age: 62.38  - 30.3% female  No TCZ:  - mean age 61.35  - 24.2% female | severe | 10 patients (15.1%) received 800 mg of TCZ, 3 patients (4.5%) received  600mg of TCZ, and 53 patients (80.3%) received 400 mg. F | N/A | refer to regimen | N/A | all-cause in-hospital death. | No benefit |
| 107 | George Sakoulas et al^107^  (2020) | RCT | Total: 33  - IVIG: 16  - SOC: 17 | IVIG:  - mean age 54  - 63% male  SOC:  - mean age 54  - 59% male | moderate to severe | The IVIG  treatment arm consisted of the subject receiving IVIG (Octagam 10% provided by  Octapharma USA, Inc) 0.5 g/kg daily for 3 days beginning on the day of enrollment in addition to SOC. For subjects not already receiving glucocorticoid therapy, enrolled  treatment arm subjects received MP 40 mg IV once 30 minutes before  IVIG to mitigate headache commonly experienced after IVIG therapy | N/A | refer to regimen | 30 days | endpoint of i)  respiratory failure requiring receipt of mechanical ventilation (a composite of either  receiving ventilation or the subject status changed to a do not resuscitate/do not  intubate resulting in progressive respiratory failure and death) or ii) death from nonpreparatory causes prior to receipt of mechanical ventilation | Benefit |
| 108 | Francesco Salton et al^108^  (2020) | RS | Total: 173  - MP: 83  - Control: 90 | MP:  - mean age 64.4  - 65.1% male  Control:  - mean age 67.1  - 73.3% male | severe | Exposure to MP (non-patented drug, ATC code H02AB04) complied with the  following protocol: a loading dose of 80 mg iv at study entry (baseline), followed by an infusion of 80  mg/day in 240 mL normal saline at 10 mL/h for at least eight days, until achieving either a PaO2:FiO2  > 350 mmHg or a CRP < 20 mg/L. After which, oral administration at 16 mg or 20 mg iv twice daily  until CRP reached < 20% of normal range or a PaO2:FiO2 > 400 (alternative SatHbO2 ≥ 95% on room  air). | N/A | refer to regimen | between February 27th and April 24th, 2020. Follow-up continued through  May 21st, 2020 | admission to ICU, need for invasive MV,  or all-cause death by day 28 | Benefit |
| 109 | Sarah M Lofgren et al^109^  (2020) | RCT | Total: 1312  - HQ: 658  - Placebo:654 | - median age 40  - 51.4% women | Not specified | The first two trials evaluated: 1) post-exposure prophylaxis (PEP); 2) preemptive early treatment (PET), (Clinicaltrials.gov Identifier: NCT04308668). Trial enrollment began on March 17, 2020, concluded on May 6, and follow up was completed on May 20, 2020. The third trial assessed pre-exposure prophylaxis (PREP) for COVID-19 (ClinicalTrials.gov Identifier: NCT04328467). Enrollment for this third trial began April 6 and ended May 26, 2020, with follow-up concluding on July 13, 2020.  Hydroxychloroquine dosing for both the PEP and PET trials was 800mg load dosing, followed by 600mg in 6-8 hours, and then 600mg daily for five days in total. Participants were instructed to split their follow-up dosing in the event of gastrointestinal upset. In designing the trials, investigators chose doses within the existing FDA-approved dosing range that were modeled to achieve therapeutic concentrations from day 1 through 10. Hydroxychloroquine dosing for PREP was dosed at 400 mg orally once, followed by 400mg 6 to 8 hours later, thereafter 400mg weekly or twice weekly for the duration of follow-up, up to 12 weeks. The placebo was dosed similarly. | N/A | refer to regimen | 14 days | Incidence of COVID19 Disease among those who are asymptomatic at baseline, adverse reaction | mild side effects |
| 110 | Yayquier Díaz et al^110^  (2020) | case series | Total: 38(presumably)  - itolizumab:19  - control :19  (paper declares matched control subjects were used, but no demographic information is provided) | 12/19 female,  age N/A | moderate | patients received standard treatments (LPV/R (kaletra), chloroquine, prophylactic antibiotics, interferon alpha (α) 2B and low-molecular-weight heparin (LMWH)) included in the institutional protocol for COVID-19.  In addition, patients received a first intravenous dose of 200 mg of a humanized Mab, itolizumab (8 vials). Some patients received a second dose (200 mg), considering their clinical evolution and the physician criteria. Itolizumab-associated adverse events (AE) were reported. | 1 day | refer to regimen | maximum 40 days | serum IL6-level, admission to ICU, mortality | Benefit |

**Table continued; numbers noted here are comparable to the previous table.**

| **#** | **Country/region** | **included date** | **KEY inclusion or**  **Exclusion criteria** | **criteria for severity assessment** | **other comments on protocol** | **Adjustment/propensity matching/controlled baseline for primary/secondary outcomes** |
| --- | --- | --- | --- | --- | --- | --- |
| 1 | U.S/NY | Admitted from March 15 to 28, 2020 | Inclusion - random sampling (~30%) was applied to the patient ≥ 45 with lab-confirmed COVID-19  Exclusion  - discharge within 24 hours - chart too incomplete for review | -abnormal chest imaging -respiratory rate>22/min -systolic BP<90 mmHg or -diastolic BP<60mmHG -O2 saturation -fever >38°C -elevated creatinine, AST/ALT | N/A | Models adjusted for sex, age category (<65 vs65 years), diabetes, any  chronic lung disease, cardiovascular disease, abnormal chest imaging,  respiration rate >22/min, O2 saturation <90%, elevated creatinine, and AST  >40 U/L as fixed effects and repeated measures for hospital. |
| 2 | Brazil/Manaus | Study conducted from March 23 to April 5, 2020 | Inclusion - 18-80 years with lab-confirmed and suspected COVID-19(virological and clinical and epidemiological) - hospitalized - RR> 24 AND/OR HR >125 bpm (in the absence of fever) AND/OR O2 sat <90% in ambient air AND/OR shock | - respiratory rate higher than 24 rpm and/or heart rate higher than 125 bpm (in the absence of fever)  - and/or peripheral oxygen saturation lower than 90% in ambient air   - and/or shock (ie, arterial pressure lower than 65mmHg, with the need for vasopressor medicines, oliguria, or a lower level of consciousness) | According to hospital protocol, all patients meeting the same criteria of the study (ie, acute respiratory distress syndrome) received intravenous ceftriaxone (1 g twice daily for 7 days) plus azithromycin (500mg once daily for 5 days), systematically, starting on day 0. | RCT |
| 3 | U.S/NY | Admitted from March 7 to April 8, 2020 | Inclusion - lab-confirmed COVID-19 - moderate to severe respiratory illness  Exclusion - intubated - transferred to another facility within 24 hours after presentation to the emergency department | resting oxygen saturation of less than 94% while they were breathing ambient air | 59.9% of patients in HQ group and 37.2% of patients in no HQ group prescribed azithromycin, and 74.5% of patients in HQ group and 66.8% of patients in no HQ group prescribed any antibiotics. | PSM |
| 4 | China/  Wuhan | patients enrolled from January 18, 2020 to February 3, 2020 | Inclusion - lab-confirmed COVID-19 - pneumonia firmed by chest imaging - O2 sat 94% or less while they were breathing ambient air or a ratio of the partial pressure of oxygen (Pao2) to the fraction of inspired oxygen (Fio2) (Pao2:Fio2) at or below 300 mg Hg  Exclusion - Physician decision that involvement in the trial was not in the patient's best interest - presence of any condition that would not allow the protocol to be followed safely, known allergy or hypersentivity to LPV/R, known severe liver disease - pregnancy or breast feeding or known HIV infection | - had pneumonia confirmed by chest imaging - oxygen saturation (Sao2) of 94% or less while they were breathing ambient air or a ratio of the partial pressure of oxygen - (Pao2) to the fraction of inspired oxygen (Fio2) (Pao2:Fio2) at or below 300 mg Hg | N/A | RCT |
| 5 | Hong Kong/ Queen Mary Hospital, Pamela Youde Nethersole Hospital, Ruttonjee Hospital, United Christian Hospital, Queen Elizabeth Hospital, and Tuen Mun Hospital | admitted to  hospital from Feb 10 to March 20, 2020 | Inclusion - at least 18 - lab-confirmed COVID-19 - National early warning score 2(NEWS2) of at least 1 - symptom duration of 14 days or less upon recruitment - Auditory temperature ≥38°C or other symptoms including cough, sputum, production, sore-throat, nasal discharge, myalgia, headache, fatigue or diarrhea upon admission  - Symptom duration ≤14 days  Exclusion - Patients with known prolonged QTc syndrome, ventricular cardiac arrhythmias, including torsade de pointes, second or third degree heart block, QTc interval ≥480ms | NEWS2 and SOFA score | For those recruited and treated between days 7 and 14, interferon beta-1b injection was omitted to avoid its pro-inflammatory effects. | RCT |
| 6 | China/Hubei | Patients enrolled from February 6 to March 12, 2020 | Inclusion - admitted to hospital with severe COVID-19. - lab-confirmed diagnosis with pneumonia confirmed by chest imaging - had O2 saturation of 94% or lower or a ratio of arterial oxygen partial pressure to fractional inspired oxygen of  300 mm Hg or less, and were within 12 days of symptom onset.   Exclusion - pregnancy or breast feeding; hepatic cirrhosis; severe renal impairment,   - enrolment into an investigational treatment study for COVID-19 in the 30 days before screening | National Early Warning Score 2 level (NEWS2)    six-point ordinal scale of clinical status | N/A | RCT |
| 7 | China/Shenzhen | Patients screened from January 30 to February 14, 2020 | Inclusion - aged 16–75 years old - lab-confirmed - patients who had initially been treated with antiviral therapy with LPV/R - duration of disease onset to enrollment was less than 7 days   Exclusion - RR > 30/min - O2 Saturation below 93%, Oxygenation index(OD) < 300 mmHg (1 mmHg = 133.3 Pa), respiratory failure, shock, and/or combined failure of other organs that required ICU monitoring and treatment); chronic liver and kidney disease and reaching end stage; pregnancy | Severe clinical condition (meeting one of the following criteria:   - a resting respiratory rate greater than 30 per minute, oxygen saturation below 93%, - oxygenation index (OI) < 300 mmHg (1 mmHg = 133.3 Pa),   - respiratory failure, shock,   - and/or combined failure of other organs that required ICU monitoring and treatment);   - chronic liver and kidney disease and reaching end stage;   are excluded | N/A | Open label controlled trial. Comparable baseline characteristics (including severities) between groups |
| 8 | U.S and Canada | Enrollment began on March 17, 2020 | Inclusion: - participants who had household or occupational exposure to a person with confirmed Covid-19 at a distance of less than 6 ft for more than 10 minutes while wearing neither a face mask nor an eye shield (high-risk exposure) or while wearing a face mask but no eye shield (moderate-risk exposure).  - with the eligibility window extended to within 4 days after exposure.  Exclusion - age younger than 18 - hospitalized - Persons with symptoms of Covid-19 or with PCR-proven SARS-CoV-2 infection were excluded | N/A | N/A | RCT |
| 9 | France | Included from March 15 and March 29, 2020 | Inclusion:  Lab-confirmed COVID-19 admitted to ICU | ICU admission | - Other treatment favoring QT prolongation in ICU: 20(50%) | N.A for adverse effect |
| 10 | U.S/Boston | admitted between March 1 and April 7,2020, | Inclusion - admitted patients - received at least 1 day of HQ - lab-confirmed COVID-19 | Clinical status, refer to severities column | N/A | N.A for adverse. Baseline QTc was different between groups and thereby we utilized delta QTc for our analysis |
| 11 | China/  Guangzhou | Screened from February 1 to March 28, 2020 | Inclusion - age between 18 and 80 years - lab-confirmed COVID-19 diagnosis - mild clinical status, defined as having mild clinical symptoms but no signs of pneumonia on imaging - moderate clinical status, defined as having fever, respiratory symptoms and pneumonia on imaging - No poor lab findings   Exclusion - severe underlying disease including mental illness affecting treatment compliance - deemed otherwise unsuitable for the study by researchers. | - mild to moderate specified in inclusion criteria  - The severe status: experiencing respiratory distress, RR≥30 times/minute; oxygen saturation ≤93% in the resting state; arterial blood oxygen partial pressure (PaO2)/oxygen concentration (FiO2) ≤300 mmHg (1mmHg =0.133kPa).   - The critical status: development of respiratory failure requiring mechanical ventilation; occurrence of shock; requirement for ICU monitoring and treatment because of complications with other organ failures. | Antiviral treatment was discontinued for patients who 1) had been treated for more than 7 days and tested negative for SARS-CoV-2 nucleic acid in two consecutive tests separated by more than 24 hours, or 2) were discharged from hospital, or 3) had intolerable side effects. | RCT |
| 12 | China/Hubei, Henan, Anhui | Admitted from  February 11 to February 29, 2020 | Inclusion - age 18 or older - lab-confirmed COVID-19 diagnosis  Exclusion - severe conditions including malignancies, heart, liver, or kidney disease or poorly controlled metabolic diseases; unsuitability for oral administration; pregnancy or lactation - inability to cooperate with investigators due to cognitive impairments or poor mental status | fifth version of the Chinese guideline for the management of covid-19 | 90 (60%) patients received concomitant drug treatment before randomization. Among these, 52 (35%) patients received antiviral treatment.  The dose of HQ was adjusted when adverse events were related to HQ, as judged by investigators. Details of dose adjustment are provided in the study protocol available in the online supplement. | RCT |
| 13 | France | Admitted between  March 12 and March 31 ,2020 | Inclusion - aged 18-80 years - lab-confirmed COVID-19 diagnosis - required oxygen by mask or nasal prongs (corresponding to a WHO progression score of 5).  Exclusion criteria  - HQ treatment started before admission to hospital; treatment with another experimental drug for covid-19 (TCZ, LPV/R, or remdesivir) within 48 hours after admission; - organ failure that required immediate admission to the intensive care unit or continuous care unit; acute respiratory distress syndrome at admission (defined by the need for non-invasive ventilation with provision of continuous positive airway pressure or invasive mechanical ventilation);  - discharge from the intensive care unit to standard care;  - decision to limit and stop active treatments prescribed at admission; | WHO progression score | Azithromycin was administered to 18% of the participants in the treatment group versus 29% in the control group; amoxicillin and clavulanic acid was given to 52% versus 28%, respectively  No patients received antiviral or anti-inflammatory treatments, including steroids or non-steroidal anti-inflammatory drugs  (non-steroidal anti-inflammatory drugs are not used as antipyretics in adults in France and are considered to be contraindicated for covid-19) before transfer to the intensive care unit. | mortality |
| 14 | China/  Wuhan | Admitted from January 25 to February 25, 2020 | Inclusion - had complete records and were critically ill and treated with antiviral agents | Critically ill patients were defined as those with ARDS (PaO2/FiO2 ≤ 300 mmHg; when PaO2 is not available, SpO2/FiO2 ≤ 315 suggests ARDS) or sepsis with acute organ dysfunction. (WHO interim guidance (March 13,2020)) | Research Letter  All patients were given antiviral therapy (e.g., oseltamivir, arbidol, LPV/R, ganciclovir, interferon-α), and 151 (62%) were given adjuvant CS treatment (median hydrocortisone equivalent dosage 200 [range 100–800] mg/day). | adjusted via PSM |
| 15 | China/  Guangzhou | Admitted from January 20 to March 15, 2020 | Inclusion - lab confirmed COVID-19 diagnosis admitted in Guangzhou Eighth People’s Hospital  Exclusion not specified | Chinese COVID-19 guideline: Guidelines for the Prevention, Diagnosis, and Treatment of Novel Coronavirus-induced Pneumonia, The 7th ed | N/A | They presented sensitivity analysis for moderate illness patients where severity indices were controlled. They further adjusted for age, disease severity, time lag from illness onset to hospital admission, body temperature, and corticosteroid using a regression model. |
| 16 | China/Wuhan, GuangZhou, and Shenzhen | Enrolled from December 23, 2019 to March 31, 2020 | Inclusion Criteria:  - Lab-confirmed COVID-19; or - conformed plasma positive of specific antibody (IgM or/and IgG) against SARS-COV-2; - In-hospital treatment ≥72 hours;  - Meet any one of the following criteria for severe type(a-c) or criteria for critical type(d-f): (a) Respiratory rate >=30/min; or (b) Rest SPO2<=90%; or (c) PaO2/FiO2<=300 mmHg; or (d) Respiratory failure and needs mechanical ventilation; or (e) Shock occurs; or (f) Multiple organ failure and needs ICU monitoring;  Exclusion Criteria:  - Exist of other evidences that can explain pneumonia including but not limited to influenza A virus, influenza B virus, bacterial pneumonia, fungal pneumonia, noninfectious causes, etc.;  - Women who are pregnant or breast-feeding;  - Researchers consider unsuitable. | Classified following Chinese Recommendations for Diagnosis and Treatment of Novel Coronavirus (SARSCoV2) infection (Trial 7th version) published by National Health Commission of China. | N/A | They presented sensitivity analyses for severities of illness and timings of initiation of treatment. |
| 17 | China/  Guangzhou | Included from January 17 to February 13, 2020 | Inclusion - with pneumonia without Invasive or non-invasive ventilation.  - No other exclusion criteria were applied at this stage. | Pneumonia without Invasive or non-invasive ventilation.  An overall lung “total severity score “was reached by summing the five lobe scores. Each of the five lung lobes was assessed for degree of involvement and classified as none (0%), minimal (1 - 25%), mild (26 - 50%), moderate (51 - 75%), or severe (76 - 100%). | N/A | comparable baseline characteristics between groups (controlled) |
| 18 | China/Wuhan | admitted from January 20 to February 25, 2020 | Inclusion - severe patients with COVID-19 pneumonia | Coronavirus pneumonia diagnosis and treatment plan (trial version 5) developed by the National Health Committee of the People’s Republic of China. Severe case was defined when any of the following criteria was met: (1) respiratory distress, respiratory rate per min ≥ 30; (2) in the resting state, means oxygen saturation ≤ 93%; (3) arterial blood oxygen partial pressure/oxygen concentration ≤ 300 mmHg. | Oxygen therapy, antiviral therapy (a-interferon, Kaletra [LPV/R]), immunoenhancement therapy (thymosin), prevention of bacterial infection, relieving cough eliminating phlegm, and nutritional support were commonly used for all of the 46 patients; | comparable baseline characteristics between groups (controlled) |
| 19 | U.S/NY | admitted patients from March 1 to March 23,2020 | Inclusion - lab-confirmed COVID-19 diagnosis - Treated with chloroquine/HQ ± azithromycin  Exclusion - Patients chronically on HQ for autoimmune diseases such as lupus | Criteria for the use of chloroquine/HQ +/- azithromycin therapy placed on March 1st were as follows: confirmed Covid-19 PCR testing or high suspicion of Covid-19 with test pending; ARDS or severe illness characterized by SIRS criteria; or clinician’s judgement that the patient is likely to progress to ARDS or severe illness in the next 6 hours. | N/A | Basline QTc interval was comparable between groups |
| 20 | China/multi-center  . | recruited from February 14 to April 1 2020 | Inclusion - signed informed consent; - aged at least 18 years;  - lab-confirmed COVID-19 diagnosis;  - 0 positive PCR result within 72 hours prior to randomization;  - pneumonia confirmed by chest imaging; - clinical symptoms meeting the definitions of severe or life-threatening COVID-19;  - hospital admission;  Exclusion - life expectancy less than 24 hours; - disseminated intravascular coagulation;  - severe septic shock;  - PaO2/FIO2 of less than 100;  - severe congestive heart failure; | Severe COVID-19 was defined as respiratory distress (≥30 breaths/min; in resting state, oxygen saturation of 93% or less on room air; or arterial partial pressure of oxygen (PaO2)/fraction of inspired oxygen (FIO2) of 300 or less. Lifet hreatening COVID- 19 was defined as respiratory failure requiring mechanical ventilation; shock; or other organ failure (apart from lung) requiring intensive care unit (ICU) monitoring. | N/A | RCT |
| 21 | multinational | Enrolled from Feb 21 to April 19, 2020 | Inclusion - 18 years of age or older who were hospitalized with symptoms suggestive of COVID-19 with a laboratory-confirmed COVID-19 diagnosis - Participants had to meet one of the following criteria suggestive of lower respiratory tract infection at the time of enrollment: radiographic infiltrates by imaging study, peripheral oxygen saturation (SpO2) ≤94% on room air, or requiring supplemental oxygen, mechanical ventilation, or extracorporeal membrane oxygenation (ECMO).  - No limit to the duration of symptoms prior to enrollment.   Exclusion  - pregnancy or breast-feeding; and anticipated discharge from the hospital or transfer to another hospital within 72 hours of enrollment. - poor lab finding | Eight-category ordinal scale and the National Early Warning Score(NEWS) | N/A | RCT |
| 22 | multinational | Enrolled from March 6 and March 26, 2020 | Inclusion: - Aged ≥ 12 years - lab-confirmed VOID-19 diagnosis ≤ 4 days before randomization - Currently hospitalized with fever defined as temperature ≥ 37.2 °C oral, ≥ 37.8 °C rectal SpO2 ≤ 94% on room air at screening - Radiographic evidence of pulmonary infiltrates  Exclusion: - Concurrent treatment with other agents with actual or possible direct acting antiviral activity against SARS-CoV-2 is prohibited < 24 hours prior to study drug dosing - pregnancy, breastfeeding | Clinical status on the 7-point ordinal scale | N/A | RCT |
| 23 | China/Hunan | Enrolled from February 1 to February 20, 2020 | Inclusion: - at the age over 18 years - lab-confirmed COVID-19 diagnosis - clinically classified as moderate or severe - without comorbidity of severe heart, lung, brain diseases | - Moderate patients were defined as “patients with fever, symptoms of respiratory system and pneumonia changes in CT images  - severe patients were defined as “patients with any of the following:  - Respiratory distress, respiratory frequency ≥30/minute; - Under rest status, arterial oxygen saturation (SaO2)≤ 93%; - Arterial partial pressure of oxygen(PaO2)/fraction of inspired oxygen (FiO2)≤ 300mmHg. | N/A | RCT |
| 24 | China/Wuhan | Enrolled from February 2020 to March 2020 | Inclusion: - Patients diagnosed with critically ill COVID COVID-19.  - complies with the COVID COVID-19 Critical and Critical Diagnostic Standards("Pneumonitis Diagnosis and Treatment Scheme for Novel Coronavirus Infection (Trial Version 5))    Exclusion: - Pregnant or breastfeeding women;  - other life life-threatening diseases such as cancer - Expected survival time <24 hours;  - A history of immune system diseases or diseases closely related to the immune system | COVID-19 Critical and Critical Diagnostic Standards, namely "Pneumonitis Diagnosis and Treatment Scheme for Novel Coronavirus Infection (Trial Version 5); | N/A | RCT |
| 25 | China/Zhejiang | Trial initiated on February 3, 2020  End date in protocol:  May 31, 2020 (exact inclusion date not specified in the script) | Inclusion: - Adults 18-85 years of age - lab-Confirmed COVID-19 diagnosis  Exclusion: - Weight < 40 kg - Critical illness meeting one of the following conditions: respiratory failure and mechanical ventilation; shock; other organ failure requiring ICU monitoring and treatment;  - severe comorbidities -poor lab finding detected within 24 hours before screening(according to local laboratory reference range) - Base on the researcher's judgment | NEWS2 | N/A | RCT |
| 26 | U.S/LA | admitted from February 1, to April 4, 2020 | Inclusion: - lab-confirmed COVID-19 diagnosis - as well as patients under investigation(PUI) - received either AZ and/or HQ as part of medical treatment  Exclusion: - patients with paced ventricular rhythms, atrial fibrillation, atrial flutter, supraventricular tachycardia, or ECGs otherwise unsuitable for accurate QT interval measurement - patients without ECGs performed on day 2 of medication administration or later | Tisdale Score The Elixhauser comorbidity index | N/A | baseline QTc interval was different between groups, thereby we used delta QT (>60ms) for analysis |
| 27 | U/S/NY | Admitted from February 25 to April 13, 2020 | Inclusion: - aged 18 or more - lab-confirmed COVID-19 diagnosis at presentation or within no more than 72 hours following admission  Exclusion: - survived less than 48 hours following hospital admission or  - if they required urgent or semi-urgent intubation within 48 hours of hospital admission | initial oxygen requirement and refer to exclusion criteria | N/A | PSM and adjusting for baseline patient characteristics |
| 28 | U.S/Veterans Health Administration medical centers | Index date from March 9 to April  11, 2020 | Inclusion - lab-confirmed COVID-19 diagnosis - patient with complete record: BMI, vital signs during an encounter, discharge disposition status available for the hospitalization - male | Clinical Status - Ventilatory status - metabolic and hematologic parameter -SpO2 |  | PSM and adjusting for baseline patient characteristics |
| 29 | China/Wuhan | admitted from February 4, 2020, to February 28, 2020 | Inclusion: - Age ≥ 18 years; - Lab-confirmed COVID-19 diagnosis - Chest CT with pneumonia - SaO2/SPO2 ratio > 93% or PaO2/FIO2 ratio > 300 mmHg under the condition in the hospital room (mild illness)  Exclusion - Severe and critical illness patients  - doctor’s discretion - Retinopathy and other retinal diseases - Conduction block and other arrhythmias - severe comorbidities - Pregnant or breastfeeding  - Possibility of being transferred to another hospital within 72 h - Received any trial treatment for COVID-19 within 30 days before this research. | Criteria of the China National Health Commission | N/A | RCT |
| 30 | U.S/NY | admitted from March 2 to April 5, 2020 | Inclusion: - at least one positive test for COVID-19(lab-confirmed COVID-19 diagnosis) - received HQ and azithromycin  - had either been discharged from the hospital, transitioned to hospice, or expired.   Exclusion: - never admitted to the hospital or  - if there was an order for other investigational therapies for COVID-19, including TCZ, nitazoxanide, rituximab, anakinra, remdesivir, or LPV/R during the course of their hospitalization to avoid potential confounding effects of these medications. | Clinical Status including SPO2, RR and laboratory indices | N/A | comparable baseline characteristics between groups (controlled) |
| 31 | China/Wuhan | Collected from February 21 to March 18, 2020 | Inclusion:  - hospitalized patients with lab-confirmed COVID-19 diagnosis  - treated with lopinavir LPV/R alone or combined with arbidol for antiviral therapy.   Exclusion criteria - hospitalized patients with pneumonia caused by other pathogens - hospitalized patients with COVID-19 haven’t received LPV/R for antiviral therapy. | Diagnosis and treatment protocol for novel coronavirus pneumonia issued by General Office of National Health Commission (version 7) | N/A | comparable baseline severity indices between groups (controlled) |
| 32 | China/Guangdong and Hubei | Conducted from February 7 to March 8, 2020 | Inclusion: - 16 years or older - lab-confirmed COVID-19 diagnosis  Exclusion - Pregnant women - Severe comorbidities - Arrhythmia or second/third degree heart block, known retinopathy, hypoacusis or hearing loss, mental disease, glucose-6-phosphate dehydrogenase (G6PD) deficiency - Received digitalis drugs within the 7 days preceding enrollment - Critical case | China’s Novel Coronavirus Pneumonia Diagnosis  and Treatment Plan (4th Edition) | N/A | They presented subgroup analysis for time from onset to treatment initiation and sensitivity analysis for moderate illness patients where severity indices were controlled. |
| 33 | China/Wuhan | Collected from December 26 to March 15, 2020 | Exclusion - Non severe or critical cases - Not being diagnosed as severe cases within 24 hours since admission - The time of being diagnosed of severe/critical cases were missing. | - Severe cases were defined as those who required oxygen therapy during hospital stay.   - Critical cases were those who met any of the following conditions during the whole hospital stay: 1. mechanical ventilation was required; 2. required treatment in intensive care unit (ICU); 3. Shock occurred in hospital.  Disease severity further classified as in-hospital stay, progression to critical case, death | N/A | PSM |
| 34 | Korea/Daegu | Admitted from February 28 to April 28, 2020 | Inclusion:  - lab-confirmed COVID-19 diagnosis - moderate illness according to NIH guideline on COVID-19  Exclusion: - severe patients and patients referred to tertiary hospitals | NIH | N/A | comparable baseline characteristics between HQ plus antibiotics and Lop/R with antibiotics groups (controlled) |
| 35 | China/Wuhan | Admitted from February 1 to March 15, 2020 | Inclusion: - met the diagnostic standards of novel coronavirus pneumonia (7th edition) formulated by the National Health Commission of China - experienced any of the following: shortness of breath, respiration rate(RR) ≥ 30 breaths/minute; resting oxygen saturation ≤ 93%; PaO2/FiO2 ≤ 30 mmHg; lung imaging showing significant lesion progression > 50% within 24-48h, and a severe clinical classification; - age ≥ 18 years - no previous history of bronchiectasis, bronchial asthma, or respiratory diseases; - no immunosuppressant or glucocorticoid use during treatment.  Exclusion: - severe comorbidities - patients who had been treated with LMWH in the previous three month. - critically ill or admitted in the ICU | diagnostic standards of novel coronavirus pneumonia (7th edition) formulated by the National Health Commission of China | N/A | comparable baseline characteristics between treatment groups (controlled) |
| 36 | China/Hubei | Admitted from January 17 to February 28, 2020 | Inclusion: - adult patients - lab-confirmed COVID-19 diagnosis  Exclusion - Patients without available clinical or imaging data | The severity degree of COVID-19 at the time of admission is defined as follows: (1) mild type, with fever or respiratory tract symptoms but without radiological pneumonia. (2) Moderate type, with fever, respiratory tract symptoms, and radiological evidence of pneumonia. (3) Severe type, with one of the following: a) respiratory distress (respiratory rate ≥ 30 beats/min); b) hypoxia (oxygen saturation ≤ 93% in the resting state); c) hypoxemia (arterial blood oxygen partial pressure/oxygen concentration ≤ 300mmHg). (4) Critical type, with one of the following: a) respiratory failure requiring mechanical ventilation; b) shock; c) intensive care unit (ICU) admission is required for combined other organs failure. In this study, severe COVID-19 pneumonia broadly included severe and critical types. | N/A | Adjusted OR was calculated by adjusting age, gender, smoking history, hypertension, diabetes, cardiovascular disease,  chronic obstructive pulmonary disease, and lung CT score. |
| 37 | China/Xi'an | Enrolled from February 3 to February 10, 2020 | Inclusion: - aged 18 to 78 years - patients with common, severe, or critical COVID-19 pneumonia were laboratory and clinical diagnosed according to Diagnosis and Treatment for 2019 Novel Coronavirus Diseases released by National Health Commission of China | Case severity of COVID-19 is categorized as common, severe, and critical by the vital signs, oxygenation index (PaO2/FiO2), chest radiographic, and vital organ function based on the Diagnosis and Treatment for 2019 Novel Coronavirus Disease. (China NHCo. Diagnosis and Treatment for 2019 Novel Coronavirus Diseases (4th Edition).) | N/A | controlled clinical trial |
| 38 | U. S | hospitalized from January 20 to May 1, 2020 | Inclusion: - 18 years or more - lab-confirmed COVID-19 diagnosis (WHO and CDC guidelines)  Exclusion: - received potential COVID-19 specific therapeutic agents (N=246) other than HQ (Azithromycin and CSs were not considered COVID-19 specific) | N/A | N/A | PSM |
| 39 | U.S/NJ | hospitalized from March 1 to April 22,2020 | Inclusion: - lab-confirmed COVID-19 diagnosis - Non-pregnant - Did not die during first day of hospitalization - Were not discharged to home within 24 hours. | Oxygenation <94% Ferritin >1500 ng/ml D-dimer >1 mcg/ml ICU admission | N/A | PSM |
| 40 | Italy/Milan | admitted from March 13th to March 19th, 2020 | Inclusion - lab-confirmed COVID-19 diagnosis - (CRP, ≥ 100 mg/L, normal values <6 mg/L) or ferritin (≥ 900 ng/mL,,normal value <400 ng/mL), in the presence of increased lactate dehydrogenase(LDH, > 220 U/L); - severe respiratory involvement defined by typical radiological findings at chest X-ray and/or computed tomography (CT) scan, in the presence of an oxygen saturation (SaO2) ≤92% while breathing ambient air or a ratio of the partial pressure of oxygen (PaO2) to the fraction of inspired oxygen (FiO2) (PaO2:FiO2) ≤300 mmHg  Exclusion - evidence of concomitant bacterial infection, history of diverticular disease, neutropenia < 1500 10^9 cells/L, concomitant use of other immunosuppressive biologic drugs, baseline elevation of alanine aminotransferase (ALT) or aspartate aminotransferase (AST) levels > 5-fold the upper limit of the normal range. No concomitant CS therapy was allowed. | refer to inclusion criteria | All patients received the same background treatment, following an Institutional protocol for standard of care: hydroxychloroquine 400 mg daily, LPV/R 400/100 mg twice daily, ceftriaxone 2 gr for 6 days, azithromycin 500 mg daily until a negative report of urine antigen for L. pneumophila, anti-coagulation prophylaxis with enoxaparin 4000 UI subcutaneously once a day. Due to a shortage of TCZ in Italy, only patients who were admitted between March 13th and March 19th, 2020 were treated with TCZ. Patients admitted to hospital before or after the time period of TCZ availability who retrospectively fulfilled eligibility criteria for TCZ treatment were used as a comparison group. | comparable baseline characteristics between groups (controlled) |
| 41 | China/multi-center | screening between February 9, and February 28, 2020 | Inclusion - met diagnostic criteria for COVID-19 - age:18~75 - severe cased according to Chinese management guideline for COVID-19  Exclusion - patients with concomitant malignant tumor - severe cardiovascular and metabolic disease, not medically controlled - need for invasive mechanic ventilation | Chinese management guideline for COVID-19(5th ed) | N/A | RCT |
| 42 | France/Paris | From March 24 to April 6, 2020 | Inclusion - aged older than 18 years - severe COVID-19-related bilateral pneumonia - lab-confirmed COVID-19 diagnosis or a typical aspect on CT scan of the lungs (multiple ground-glass abnormalities with crazy paving, absence of lymphadenopathy, and pulmonary nodules); had bilateral lung infiltrates on a lung CT scan or chest x-ray - had critical pulmonary function defined by oxygen saturation of 93% or less under 6 L/min of oxygen or more or oxygen saturation of less than 93% on 3 L/min with a saturation on ambient air decreasing by 3% in the previous 24 h.  Exclusion - bedridden and near the end of life, patients with respiratory failure explained by an alternative etiology, and patients already admitted to the ICU | Clinical status: Chest CT, O2 saturation, Oxygen therapy | N/A | adjusted multivariate analysis |
| 43 | Italy | Patients admitted from Feb 26 to April 2, 2020 | Inclusion: - Lab-confirmed COVID-19  - at east one of the following conditions: 1) respiratory rate ≥ 30 breaths/ min, 2) peripheral capillary oxygen saturation (SpO2) ≤ 93% while breathing room air, 3) PaO2/FiO2 <=300 mmHg.   Exclusion Patients with critical respiratory syndrome, needing mechanical ventilation at onset - Chest x-ray showed in all patients bilateral pulmonary opacities on chest imaging that were not fully explained by congestive heart failure or other forms of volume overload. - Transaminase 5 times the upper limit of the normal value and/or neutrophils <500 / mmc or Platelets <50.000 / mmc | Clinical Status: RR, SpO2, PaO2/FIO2 | N/A | multivariate HR was adjusted for baseline variables (age, gender, comorbidities as diabetes, hypertension and heart disease) and serum PCR at admission |
| 44 | China/Wuhan | hospitalized from Jan 20 to Feb 25 2020 | Inclusion - diagnosed as non-severe COVID-19 pneumonia and discharged with recovered symptoms or developed to severe cases in the hospitalization - lab-confirmed COVID-19 diagnosis - non-severe cases | the coronavirus pneumonia diagnosis and treatment plan (trial version 7) developed by the National Health Committee of the People's Republic of China | N/A | PSM |
| 45 | Spain/Hospital Puerta de Hierro-Majadahonda | Admitted from March 4to April  7, 2020 | Inclusion - COVID-19 diagnosis according to WHO interim guidance - complicated with ARDS and/or an hyperinflammatory syndrome | ARDS and/or an hyperinflammatory syndrome | Among patients treated with steroids, 310 (78.3 %) patients were initially treated with 1 mg/kg/day MP or equivalent (22.5% of them  received steroid pulses later-on) and 86 (21.7%) received pulses from the beginning. | PSM |
| 46 | Spain/Barcelona | Admitted from Feb 19 to April 16, 2020 | Inclusion - lab-confirmed COVID-19 or clinical criteria - patients that did not qualify as requiring transfer to the ICU during the first - 24h after admission to a conventional hospital ward, | refer to inclusion criteria | The standard protocol included antiviral treatment that consisted of LPV/R 400/100 mg BID for 7-14 days plus hydroxychloroquine 400 mg/12h on the first day, followed by 200 mg/12h for the next 4 days. From the 18th of March onwards,azithromycin 500 mg the first day and 250 mg/24h for 4 additional days was added to the regimen. All patients with risk factors for thrombosis received prophylactic doses of low molecular weight heparin. Intravenous MP was recommended for patients with disease progression to ARDS. | multivariate adjustment |
| 47 | U.S/Michigan | admitted from March 9 to April 20,2020 | Inclusion - lab-confirmed COVID-19 diagnosis - severe COVID-19 pneumonia - requires invasive mechanical ventilation  Exclusion - patients younger than 16 years - intubated for conditions unrelated to COVID-19, or were enrolled into a RCT for sarilumab | refer to inclusion criteria, and 6 level ordinal scale of illness severity | N/A | multivariable cox regression with propensity score inverse probability weighting (IPTW) |
| 48 | Iran/Tehran | Admitted from February 29 to April 3, 2020 | Inclusion: - aged ≥ 18 years old) - lab-confirmed diagnosis - Patients with the severe disease: hypoxemia (need for noninvasive or invasive respiratory support to provide capillary oxygen saturation above 90%) (2) Hypotension (systolic blood pressure less than 90 mmHg or vasopressor requirement) (3) renal failure secondary to COVID-19 (according to KDIGO definition) (4) neurologic disorder secondary to COVID-19 (decrease of 2 or more scores in Glasgow Coma Scale)(5) thrombocytopenia secondary to COVID-19 (platelet count less than 150000 /mm3) (6) severe gastrointestinal symptoms secondary to COVID-19 (vomiting/diarrhea that caused at least mild dehydration). | refer to inclusion criteria | N/A | RCT |
| 49 | France/Aulnay-sous-bois | Screening began on March 14, 2020. TCZ available on March 23,2020 | Inclusion: - lab-confirmed COVID-19 diagnosis or CT scan with typical lesions  - severe COVID-19 pneumonia - (SpO2) ≤96% despite 79 O2 flow 6 L/minute delivered by high concentration oxygen mask, for >6 hours  Exclusion: - patients with invasive mechanical ventilation - patient in critical care medicine department | refer to inclusion criteria | N/A | PSM, multivariate cox regression, and inverse probability treatment weighting |
| 50 | U.S /Canada | from 22 March through 20 May 2020 | Inclusion:  - non-hospitalized adults who were required to have 4 or fewer days of symptoms and either  PCR-confirmed SARS-CoV-2 infection or compatible  symptoms after a high-risk exposure to a person with  PCR-confirmed COVID-19 within the past 14 days.  Exclusion:  - younger than 18 years, were  hospitalized, received certain medications, or met other  safety exclusion criteria | Questionnaire based symptom severity scale 0-10, 10 being most severe | N/A | RCT |
| 51 | U.S | Enrolment  took place from April 8, 2020, to May 13, 2020 | Inclusion:  - with a diagnosis of COVID-19 determined by reverse transcription polymerase chain reaction and pneumonia documented by chest imaging. In  addition, patients were required to have ≥ 1 symptom consistent with COVID-19, such as fever, cough, sore throat, malaise, headache, muscle pain, dyspnea, confusion, or respiratory distress, and ≥ 1 clinical sign suggesting respiratory compromise, such as respiratory rate ≥ 30 breaths per minute, heart rate ≥ 125 bpm, SpO2 < 93% on room air or requiring > 2 L oxygen by nasal cannula to maintain SpO2 ≥ 93%, or PaO2/FiO2 < 300, imputed from pulse oximetry or determined by arterial blood gas. | patients receiving low-flow supplemental oxygen at screening – severe  patients receiving high-flow supplemental oxygen through a high-flow nasal cannula at screening - Critical | N/A | RCT |
| 52 | France | From March 4, 2020, to April 6, 2020 | (1) acute respiratory failure as defined by severe hypoxemia requiring either a high level of oxygen via facemask (> 6 L/min  to achieve SpO2 > 90%), high flow oxygen therapy (with  a minimum of 30 L/min and 50% FiO2 to achieve SpO2  > 90%), or invasive mechanical ventilation and (2)  proven infection by SARS-CoV-2 defined by positive reverse transcriptase polymerase chain reaction (RT-PCR)  assay targeting the E (envelope) gene of SARS-CoV-2,  obtained from nasopharyngeal swab or lower respiratory  tracts | Sequential Organ Failure  Assessment (SOFA), Simplified Acute  Physiology Score (SAPS) 2 | N/A | comparable baseline characteristics between groups (controlled) except for respiratory rate (RR). However, severity index such as SOFA was comparable between groups and thus we judged RR is unlikely to impact the treatment outcomes substantially |
| 53 | Russia | From April 2020 to May 2020 | Inclusion:  hospitalized men and non-pregnant women of 18 years or  older who signed the informed consent form, had moderate PCR-confirmed COVID-19 (positive test at screening), were able to administrate the drug orally and willing to use adequate contraception  during the study and 3 months after its completion. | moderate COVID-19 pneumonia based on WHO R &D Blueprint recommendations | N/A | RCT |
| 54 | U.S/NY | Admitted from March 1, 2020 to April 12,2020 | Inclusion:  - laboratory confirmed diagnosis within 48 hours after admission and subsequently required mechanical ventilation.  Exclusion:  - died less than five days after hospital admission, weighed more than 200kg or if patients received steroids other than MP for greater than 24 hours. | Ventilated Status | N/A | PSM |
| 55 | Spain | Conducted from March 17, 2020 to May 26, 2020 | Inclusion:  -COVID-19 with mild symptoms(fever, acute cough, shortness of breath, sudden olfactory or gustatory loss, or influenza-like illness) for less than five days before enrollment, were non-nospitalized.  Exclusion  - patients who have moderate to severe covid-19 disease (hospitalization requirement) and other severe comorbidities | symptom based and hospitalization requirement | N/A | RCT |
| 56 | Brazil | From April 18 to June 16, 2020 | Inclusion:  clinical AND/OR radiological suspicion of COVID-19, SpO2 ≤ 94% at room air OR in use of supplementary oxygen OR under IMV.  Patients were enrolled before laboratory confirmation of COVID-190 to avoid treatment delays | refer to inclusion criteria | N/A | RCT |
| 57 | Multinational | From Feb 6 to April 10, 2020 | Inclusion:  SpO2 ≤94% on room air or required supplemental oxygen, and all had radiographic evidence of pulmonary infiltrates  Exclusion:  mechanically ventilated | refer to inclusion criteria | N/A | Inverse probability weighting and PSM |
| 58 | U. S | From March 10, 2020 to May 2, 2020 | Inclusion  hospitalization during  which the patient had a positive SARS-CoV-2 test. Diagnosis with  SARS-CoV-2 was confirmed by a positive reverse-transcriptase polymerase-chain-reaction (RT-PCR) assay from a nasopharyngeal  sample. All patients evaluated were 18 years of age and older and were treated as inpatients for at least 48 h unless they died within  the time period | refer to inclusion criteria | N/A | propensity matched cox regression model |
| 59 | U. S | From March 1 and April 22, 2020 | Inclusion:  - adult patients (aged  ≥18 years) with a positive SARS-CoV-2 diagnosis by  RT-PCR who were hospitalized at one of Hackensack  Meridian Health’s 13 hospitals during the study period  and required ICU support.  Exclusion:  - Patients receiving TCZ  for chronic rheumatological conditions | refer to inclusion criteria | N/A | propensity score matched multivariable cox model |
| 60 | Multinational | Data cut off on July 6, 2020 | Inclusion:  - Hospitalized patients were eligible for the  trial if they had clinically suspected or laboratory-confirmed SARS-CoV-2 infection and no medical  history that might, in the opinion of the attending clinician, put patients at substantial risk if  they were to participate in the trial. Initially, recruitment was limited to patients who were at  least 18 years of age, but the age limit was removed starting on May 9, 2020. Pregnant or breastfeeding women were eligible. | respiratory status (oxygen support, mechanically ventilated), but severity not specified in the script | N/A | RCT |
| 61 | Brazil | Randomization from March 29, 2020 to May 17,2020 | Inclusion:  - consecutive patients who were  18 years of age or older and who had been hospitalized with suspected or confirmed Covid-19  with 14 or fewer days since symptom onset.  Exclusion:  - use of supplemental oxygen at a rate of more than 4 liters per minute as administered by a nasal cannula or at a level of at least 40% as administered by a Venturi mask; the use of supplemental oxygen administered by a high-flow  nasal cannula or invasive or noninvasive ventilation | refer to inclusion and exclusion criteria | N/A | RCT |
| 62 | China | Between January 2020 and  March 2020 | Inclusion:  - laboratory confirm diagnosis with severe and critical level of severity according to Chinese management guideline for COVID-19 | Chinese management guideline for COVID-19 | N/A | comparable baseline characteristics between groups (controlled) |
| 63 | China | admitted from January 20, 2020 to February 16,  2020 | Inclusion:  - received CS  treatment because of clinical progression of COVID-19; and  with non-severe COVID-19 pneumonia both at the time of  admission and at the time of starting CS treatment  For the purpose of comparing the effect of CSs on viral clearance, all 44 CS-untreated symptomatic patients, with non-severe COVID-19 pneumonia, who were at least 19 years old, and admitted during the same period were included in this study (referred to as non- CS group). | refer to inclusion criteria | N/A | adjusting for age of more than 60 years, sex, any underlying disease, and baseline parameters (LDH of more than 245 IU/L  , lymphocytes of <0.8 × 109 cells/L, CRP of more than 10 mg/L,,viral load [Ct value] and number of lung lobe involvement |
| 64 | Iran | study was conducted between March  to April 2020 | Inclusion:  - Confirmed COVID-19 infection through laboratory (RT-qPCR) and/or lung involvement confirmed with chest imaging (CT scan). Presence of some or all of disease clinical symptoms such as shortness of breath (dyspnea), respiratory frequency ≥ 20/min, fever and cough. Hospitalized with a blood oxygen saturation (SPO2) ≤93 % at rest on room air.  ≤7 days since illness onset  Exclusion  - mechanical ventilation  - Other critical patients (ex. Severe liver or kidney disease, septic shock etc) | refer to inclusion criteria | N/A | comparable baseline characteristics between groups (nonRCT) |
| 65 | China | enrolled from January 30, 2020, to February 6, 2020 | Inclusion:  all patients with laboratory -confirmed SARS-CoV-2 infection  Exclusion:  hypersensitivity to darunavir, cobicistat,  or any excipients; patients with severe liver injury (Child-Pugh  Class C); patients receiving concomitant medications that are  highly dependent on cytochrome P450 3A clearance, and for  which the elevated plasma concentrations are associated with serious or life-threatening events; subjects considered to be  unable to complete the study (eg, severely and critically ill patients) or not suitable for the study by researchers | Patients who met any of the following criteria were classified as severe cases: respiratory rate ≥30 times/min, pulse oxygen saturation ≤93% at resting, or ratio between partial pressure of oxygen in arterial blood and fraction of inspired oxygen (PaO2/FiO2) ≤300 mmHg Critical illness was defined as respiratory  failure that needed mechanical ventilation or shock or exacerbation of any comorbidity that required transfer to the intensive  care unit. | N/A | RCT |
| 66 | Italy | treated arm: admitted between March 15 to May 5,2020  control arm: February 20 to March 15, 2020 | Inclusion:  - SARS-CoV-2 naso-pharingeal swab-positive with a moderate pneumonia characterized by typical symptoms,  radiological findings of pneumonia, SpO2 >92% on room air, and PaO2/FiO2 100–300 mmHg  - all consecutive-hospitalized patients from  February 20th-March 15th, 2020 with moderate COVID-19 pneumonia, 18 years-older, treated with hydroxychloroquine (HCLR)  and LPV/R | refer to inclusion criteria | N/A | comparable baseline characteristics between groups (controlled) |
| 67 | China | enrolled from January 22 to February 25,2020 | Inclusion:  - patients who were newly tested  positive for SARS-CoV-2. The "suspected COVID-19  patients" were defined as individuals who had  contact history with SARS-CoV-2 positive patient(s)  and exhibited at least one of the early symptoms of  COVID-19, i.e. fever, cough, aspiration, or abnormal  findings in chest X-ray radiography or computer tomography (CT) analyses | refer to inclusion criteria | N/A | RCT |
| 68 | Netherland | Treated group From April 1, 2020  Control group admitted between March 7 and March 31, 2020 | Inclusion:  A diagnosis of COVID-19  involved the presence of clinical signs and symptoms suggestive  of COVID-19 in combination with either a positive PCR test for severe acute respiratory syndrome coronavirus 2 (SARS-CoV-2) or a chest CT result of COVID-19 CT Classification (CO-RADS) 4 or 5  patients had to have an oxygen saturation at rest ≤94% (ambient air) or tachypnoea (>30/min). In addition, they had to meet at least  two out of the following three biomarker criteria: high CRP. (>100mg/L), high serum ferritin (>900µg/L at one occasion,  or a twofold increase of the level at admission within 48hours)  and high D-dimer level (>1500µg/L). | refer to inclusion criteria | All patients received ceftriaxone (2g every  24 hours for 7 days) and up to 11 May 2020 in the presence of  oxygen saturation <90% chloroquine 300mg every 12 hours  following a loading dose of 600mg unless the corrected QT  interval on an ECG was prolonged (>500ms). Informed consent  was obtained for this off-label therapy. | multivariate adjustment in regression models |
| 69 | Austria | from 1 March  to 26 April 2020 | Inclusion:  - molecular proven SARS-CoV-2 infections were eligible for the study. need for hospitalization plus respiratory insufficiency (SpO2≤ 93% at room air or need for oxygen insufflation) or bilateral consolidations as demonstrated on chest X-ray and at least two comorbidities associated with poor COVID-  19 prognoses (e.g. age >60 years, diabetes and hypertension). | refer to inclusion criteria | N/A | comparable baseline characteristics between groups (although comorbidity profiles are minorly different, all other clinically important characteristics such as severity, symptoms, X-ray consolidations are comparable between groups) |
| 70 | China | from February 2 2020 to 20 March 2020 | Inclusion  - laboratory confirmed COVID-19  - with complete clinical data. Exclusion criterion was patients using other medications with potential antiviral activity | Novel coronavirus pneumonia prevention and control program (7th ed.) | N/A | comparable baseline severity indices between groups (controlled) |
| 71 | China | hospitalized from January 19 to February 19, 2020 | Inclusion:  - laboratory confirmed COVID-19 who are hospitalized in the First Affiliated Hospital from January 19 to February 19,2020 | A mild case was defined as slight  clinical symptoms without pneumonia or with only mild pneumonia. A severe case was diagnosed by the presence of dyspnea (respiratory rate of ≥30 times/min), resting peripheral oxygen saturation of ≤93%, arterial PaO2/FiO2 of ≤300 mmHg (1 mmHg=133.3 Pa), and/ or lung infiltrates of >50% within a 24- to 48-h period. A critical case was determined by any respiratory failure requiring mechanical ventilation, septic shock, and/or intensive care unit (ICU) admission for multiple organ dysfunction or failure. | N/A | PSM |
| 72 | Italy | admitted between February 23, and May 9, 2020 | Inclusion not specified but regimen part may help | not specified | N/A | multivariable adjusted model |
| 73 | China | admitted between January 22, 2020 and March 1, 202 | confirmed COVID-19 who were admitted to the Infectious Diseases Branch of Anhui Provincial Hospital between January 22,  2020 and March 1, 2020 | general and severe but criteria not specified but presumably by SOFA | N/A | comparable baseline characteristics between groups (controlled) |
| 74 | South Korea | patients treated from February 17 to March 31, 2020 | Inclusion:  all patients with laboratory confirmed COVID-19 diagnosis  Exclusion:  tients who were treated with  lopinavir-ritonavir and hydroxychloroquine concur rently and those who received lopinavir-ritonavir or  hydroxychloroquine for less than 7 days | Not specified but presumably respiratory status, or using seven category scale | N/A | multivariable adjusted model |
| 75 | Italy | Conducted  from  March 14 2020, through April 2 2020 | Patients enrolled were prospectively followed up with  daily data collection into an electronic case report form. Patients eligible  to sarilumab were required to have (1) confirmed SARS-CoV-2  infection by reverse-transcriptase PCR on nasal-pharyngeal  swab, (2) radiologically documented bilateral pneumonia and (3)  severe hyper inflamed COVID-19 as defined in online supplementary material. Contemporary patients fulfilling the inclusion  criteria and matched for age, sex, comorbidities, inflammatory  markers, respiratory parameters and radiological findings on  lung CT scan were identified and used as a comparison group. | severe  COVID-19 as defined by either ≤ 92% of oxygen saturation while breathing ambient air or by a  partial pressure of arterial oxygen/fraction of inspired oxygen (PaO2/FiO2) ratio ≤ 300 mmHg on  supplemental oxygen, and a hyper-inflamed phenotype as defined by an elevation of lactate  dehydrogenase (LDH) above the upper limit of normal (ULN), and by at least one of the  following: C-reactive protein (CRP) ≥ 100 mg/L; IL-6 ≥ 40 pg/ml; or ferritin (≥ 900 ng/ml) | N/A | comparable baseline characteristics between groups (contemporary matched patients) |
| 76 | Spain | Starting on March 27, end date not available | Inclusion:  - PaO2/FiO2<300, SpO2<92 (room air), tachypnea, and high  ferritin levels. | refer to inclusion criteria | N/A | multivariable adjusted model |
| 77 | China | enrolled between January 29,  2020 and February 25, 2020 | Inclusion:  (1) diagnosed as mild to  moderate COVID-19; and (2) willing to sign informed consent.  Exclusion  (1) were pregnant or breastfeeding  women; (2) had aspartate aminotransferase (AST) or alanine  aminotransferase (ALT) >5× upper normal limit, creatinine  clearance <50 ml/min (Lu and Dong, 2019); (3) were allergic or  intolerant to therapeutic drugs; (4) were HIV-positive patients; (5) had severe heart disease, brain disease, lung disease, kidney  disease, neoplastic disease, or other systemic diseases, which  may have had the potential to influence patients’ adherence to  the prescribed antiviral regimens; and (6) withheld  informed consent. | refer to inclusion criteria | N/A | RCT |
| 78 | China | January 15 through March 31, 2020 | admitted patients with  confirmed COVID-19 diagnosis who underwent anti-viral therapies in two regional medical centers of adjacent cities in Hubei,China and contains complete record | Chinese management guideline for COVID-19 version 7 | N/A | multivariable adjusted model |
| 79 | China | From January to February | Inclusion  Laboratory confirmed COVID-19, with severe level of severity  exclusion:  - Patients were excluded if they met the following exclusion criteria: (i) age <18 years or >80 years; and (ii) transferred to another hospital (after the first consultation, some patients with special medical insurance types were transferred to the corresponding  hospitals for treatment). | Patients were considered severe COVID-19 patients if they met any of the following condition: (i) oxygen saturation ≤93% at resting state; (ii) respiratory rate ≥30 breaths/min;  and (iii) arterial oxygen tension (PaO2)/inspiratory oxygen fraction  (FiO2) ≤300 mmHg | N/A | comparable baseline characteristics between groups (controlled) |
| 80 | Italy | started TCZ treatment from March 13 to April 03, 2020. | Inclusion:  - included all the patients consecutively admitted to our Hospital in Milan, in the Lombardy Region, with a diagnosis of severe or critical COVID-19 and  who started TCZ treatment from March 13th to April 03rd,  CT scan findings  of severe, bilateral interstitial pneumonia; presence of an active  inflammatory status alternatively defined by abnormal C reactive  protein (CRP) levels (>1 mg/dL), IL-6 >40 pg/mL, d-dimer >1.5  mcg/mL, or ferritin >500 ng/mL. Only individuals with severe or  critical clinical picture according to the Chinese Guidelines for the  management of COVID-19 were eligible | A “severe” case was de-  fined as the presence of respiratory distress (respiratory rate ≥30  per min), oxygen saturation on room air at rest ≤93% or P/F (or  Horowitz Index, partial pressure of oxygen in arterial blood / fraction of inspired oxygen) ≤300 mmHg; a “critical” case was de-  fined as the presence of respiratory failure with need of ventilation (either invasive or not), septic shock or any other organ dysfunction requiring ICU monitoring and treatment. | N/A | contemporary matched patient and further adjusted for severity by stratified analysis for mortality |
| 81 | Multinational | between March 15, and April 18, 2020 | Inclusion:  - Hospitalized patients with SARS-CoV-2 infection confirmed  by polymerase chain reaction assay within 4 days of randomization and moderate COVID-19 pneumonia (defined as any radiographic evidence of pulmonary infiltrates and  oxygen saturation >94% on room air) | refer to inclusion criteria | N/A | RCT |
| 82 | Italy | Between March 17 and April 15, 2020 | Inclusion:  - non-mechanically ventilated  patients for treatment with mavrilimumab who fulfilled  the following criteria: patients who were aged 18 years  or older and diagnosed with COVID-19 pneumonia by  detection of viral sequences at quantitative RT-PCR testing  (nasopharyngeal swab) and radiological findings at chest  x-ray or CT scan; had acute lung injury, defined as a ratio of  the partial pressure of oxygen to the fraction of inspired  oxygen (PaO2:FiO2) of 300 mm Hg or less, in the presence  of bilateral pulmonary infiltrates by chest radiograph or CT  scan, and no clinical evidence of left atrial hypertension; and had hyperinflammation, defined as elevation of  serum inflammation markers C-reactive protein (CRP) to  100 mg/L or more (normal range <6 mg/L) or ferritin  to 900 μg/L or more (normal range 30–400 μg/L), in the  presence of any increase in lactate dehydrogenase (LDH;  normal range 125–220 U/L).  Exclusion:  - management (including mechanical ventilation) in the intensive care unit (ICU); evidence of bacterial  infection; and concomitant administration of other immunosuppressive biological agents or CSs. | refer to inclusion criteria | N/A | comparable baseline severity indices between groups (although patients who received mavrilimumab had a longer fever duration before enrollment than had control patients, any potential difference in disease stage/severity would be small, especially considering the comparable number of days of hospital stay between groups) |
| 83 | Italy | between February 21 and March 24, 2020,  between February 21 and April 30, 2020 | Inclusion  - laboratory confirmed COVID-19 patients who were admitted to the centres in Bologna and  Reggio Emilia between Feb 21 and March 24, 2020, and to  the centre in Modena between Feb 21 and April 30, 2020  (after reviewers requested a follow-up extension, which was  only possible at the Modena centre). Eligible patients had  severe pneumonia, defined as at least one of the following:  presence of a respiratory rate of 30 or more breaths per  minute, peripheral blood oxygen saturation (SaO2) of less  than 93% in room air, a ratio of arterial oxygen partial  pressure (PaO2) to fractional inspired oxygen (FiO2) of  less than 300 mm Hg in room air, and lung infiltrates  of more than 50% within 24–48 h, according to Chinese  management guidelines for COVID-19 (version 6.0) | refer to inclusion criteria | N/A | multivariable adjusted model |
| 84 | Iran | Between March 26 and April 26 2020. | Participants were enrolled into  the study if they had both positive qualitative RT–PCR on nasopharyngeal  swab and chest CT scan compatible with moderate or severe COVID-19 infection. In addition, participants were required to have signs of severity of  disease defined as fever (oral temperature 37.8C at any one time prior to  enrolment) and at least one of respiratory rate >24/min, O2 saturation  <94% or PaO2/FiO2 ratio <300 mgHg. Only participants whose onset of  symptoms was 8 days or less were included. | refer to inclusion criteria | N/A | RCT |
| 85 | Iran | Between March 20 and April 8 2020 | Inclusion:  - laboratory confirmed COVID-19 patients. Only patients with moderate disease. on admission were included, which was defined as respiratory rate of  <24/min, arterial O2 saturation of >94% and symptom onset 8 days prior  to admission, together with compatible findings in a chest CT scan. | refer to inclusion criteria | N/A | RCT |
| 86 | Greece | recruitment started on April 3, 2020, and was terminated on April 27, 2020 | Inclusion:  - laboratory confirmed COVID-19, eligible if they had a body temperature of  37.5 °C or greater and 2 or more of the following: sustained coughing, sustained sore throat, anosmia  and/or ageusia, fatigue and/or tiredness, and arterial oxygen partial pressure lower than 95 mm Hg  on room air.  Exclusion:  - clinical  assessment indicating that ventilatory support would be inevitable in the following 24 hours because  of rapidly declining respiratory status | clinical status score, respiratory status (paO2 etc.) | N/A | RCT |
| 87 | Iran | Enrolled between March 18 and April 16, 2020 | Inclusion:  - lab-confirmed COVID-19, or bilateral multi-lobar  ground-glass opacity on their chest CT and signs of severe COVID-19,  defined as oxygen saturation less than 94% or respiratory rate above 24 or  decreased level of consciousness. The RT–PCR test used was qualitative, not  quantitative. | refer to inclusion criteria | N/A | comparable baselines (controlled) |
| 88 | U. S | from March  28 through July 6, 2020 | Inclusion:  - laboratory confirmed COVID-19. Patients were eligible for this study if they had severe and/or life-threatening COVID-  19 disease | Severe disease was defined as one or more of the following: shortness  of breath (dyspnea), respiratory rate ≥ 30/min, blood oxygen saturation ≤ 93% (on room air), partial  pressure of arterial oxygen to fraction of inspired oxygen ratio < 300, and/or pulmonary infiltrates > 50%  within 24 to 48 h (of screening assessment). Life-threatening disease was defined as one or more of  the following: respiratory failure, septic shock, and/or multiple organ dysfunction or failure | N/A | PSM |
| 89 | France | Between  April 1 and May 11, 2020 | The “TCZ group” (TCZ group) included all patients (except patients already in intensive care  unit with IMV) whom received standard treatment and TCZ (8mg/kg per dose, 1 or 2 doses). Between  April 1st and May 11  th, 2020, we enrolled all adult patients who received TCZ for confirmed COVID-19  by RT-PCR SARS-CoV-2 RNA.  The standard treatment group (ST group) included patients receiving standard treatment but without TCZ.  Exclusion:  patients with moderate disease in control group was excluded. | moderate disease (i.e. those  hospitalized for less than 48 hours and/or patients without any COVID-19 symptoms) and patients  who were less than 50-year-old (as none of the patients in the TCZ group was younger than 50 years old) | N/A | comparable baselines, except for LDH and D-dimer which are of minor gaps and thus less likely to reflect clinically significant differences in severities between groups. |
| 90 | China | Between January 20 2020 and February 13  2020 | Inclusion:  - admitted to Shanghai public health clinical center, Patients were considered eligible for CSs once chest  radiology examinations suggested they were at risk of  progression to ARDS | This study used the following brief definition: mild patients were defined as those with uncomplicated illness  or mild pneumonia; severe patients were defined as those with severe pneumonia; critically ill patients were defined as those with ARDS, sepsis and septic shock.  a rapid progression of pneumonia  defined by size increasing by more than 50% with involvement of one-third of the lung fields within 48 h or the presence of extensive ground-glass opacity involving more than half of the lung fields. | N/A | comparable baseline severity indices between groups |
| 91 | Spain | from March 17 to April 28, 2020 | Inclusion:  - study clusters (called rings) of healthy individuals (contacts) epidemiologically linked to a PCR-positive Covid-19 case (index case). | No severity | N/A | RCT |
| 92 | Brazil | From April 11 to July 6, 2020 | Inclusion:  individuals hospitalized with moderate or severe forms of  COVID-19 diagnosed by RT-PCR in nasopharyngeal swab specimens and lung computed  tomography scan involvement compatible with COVID-19 pneumonia; older than 18 years;  body weight > 50 kg; normal levels of serum Ca2+ and K+  ; QT interval < 450 ms at 12  derivations electrocardiogram (according to the Bazett formula) and negative serum or urinary  β-HCG if woman under 50  Exclusion:  mild form of COVID-19 or  in need for ICU admission; d | refer to inclusion criteria | N/A | RCT |
| 93 | Taiwan | between April 1 and May 31, 2020 | Inclusion:  - confirmed positive for SARS-CoV-2 infection by real-time reverse transcription polymerase chain reaction (rRT-PCR).  Exclusion:  - Participants presenting with severe illness | (1) mild illness without evidence of infiltration according to chest roentgenography  (2) moderate illness with evidence of infiltration according to chest roentgenography but neither respiratory distress nor supplemental oxygen requirement; and (3) severe illness with  respiratory distress, oxygen supplementation, and evidence of infiltration according to chest roentgenography | N/A | RCT |
| 94 | U.S | from the March 16 to April 30, 2020 | Inclusion:  - laboratory-confirmed COVID-19 pneumonia were screened for inclusion. 205  patients who developed AHRF (SpO2/FiO2 ≤ 440 or PaO2/FiO2 ≤ 300) were only included in the  final study.  Exclusion:  - Direct admission to the Intensive care unit (ICU), patients developing composite  primary outcome within 24 hours of admission, and patients who never became hypoxic during  their stay in the hospital | refer to inclusion criteria | N/A | multivariable adjusted model |
| 95 | Spain | April-May 2020. | Inclusion:  - a laboratory confirmed diagnosis of  SARS-CoV2 infection. Additional inclusion criteria were all the following:    1) Symptom duration of at least 7 days  2) Radiological evidence of lung disease in chest X-ray or CT-scan  3) Moderate-to-severe disease with abnormal gas exchange: PaFi (PaO2/FiO2) < 300, or SAFI  (SAO2/FiO2) < 400, or at least 2 criteria of the BRESCIA-COVID Respiratory Severity Scale (BCRSS).  4) Laboratory parameters suggesting a hyper-inflammatory state: serum C-Reactive Protein (CRP)  >15 mg/dl, D-dimer > 800 mg/dl, ferritin > 1000 mg/dl or IL-6 levels > 20 pg/ml.  Exclusion:  intubated or mechanically ventilated, were hospitalized in the ICU, | refer to inclusion criteria | N/A | partial randomized partial controlled trial; comparable baseline between groups |
| 96 | France | From February 1st to April 6, 2020 | at least one polymerase chain reaction-documented  SARS-CoV-2 RNA from a nasopharyngeal sample between February 1st, 2020 and April 6th, 2020  were eligible for the present analysis. The date of inclusion in the study cohort (index date) was  defined as the date of admission. We restricted the study population to previously  hydroxychloroquine- and azithromycin-naive inpatients, defined as those who had not received a  prescription before the index date | not specified but hydroxychloroquine to physicians as a therapeutic option for patients with moderate to-severe COVID-19 infection is mentioned in this script | N/A | IPTW adjusted analysis |
| 97 | U. S | Enrollment dates were March 15,  2020 through May 11, 2020 | Inclusion:  - laboratory-confirmed infection with SARS-CoV-2  during their admission were reviewed in this study | Patients were considered to have  severe pulmonary involvement if they required an FiO2 of 50% or greater, high-flow nasal oxygen, noninvasive ventilation, or intubation and mechanical ventilation. The  non-severe pulmonary criteria encompassed patients who required no supplemental  oxygen, or “low FIO2” (ie: Venturi mask 40% or less, or any amount of low flow nasal  cannula), independent of radiographic or laboratory findings. | N/A | comparable baseline severity indices between groups (controlled); they further presented subgroup analysis for severe and non-severe cases. |
| 98 | U. S | April 1 to the May 1, 2020 | Inclusion:  2) mild to  moderate COVID-19 diagnosed by positive polymerase chain reaction (PCR) testing <=3 days  from enrollment 3) Patient acceptance and willingness to comply with planned study procedures  and to complete the follow up. 4) hospital admission 5) no participation in other clinical trials,  such as antiviral trials, during the study period  Exclusion:  1) severe COVID-19 defined as respiratory distress  (≥30 breaths/min; in resting state, oxygen saturation of 93% or less on room air; or arterial partial  pressure of oxygen (PaO2)/fraction of inspired oxygen (FIO2) of 300 or less. 2) Life threatening  COVID-19 was defined as respiratory failure requiring mechanical ventilation; shock; or other  organ failure (apart from lung) requiring intensive care unit (ICU) monitoring | Mild and moderate COVID-19 were defined according to World Health Organization (WHO)  interim guidance. Mild COVID-19 was defined as symptomatic patients meeting the case. definition for COVID-19 without evidence of viral pneumonia or hypoxia. The symptoms  included: fever, cough, fatigue, anorexia, shortness of breath, myalgias. Other non-specific  symptoms such as sore throat, nasal congestion, headache, diarrhea nausea, vomiting, loss of smell,  loss of taste, Older people and immunosuppressed patients in particular may present with atypical  symptoms such as fatigue, reduced alertness, reduced mobility, diarrhea, loss of appetite, delirium,  and absence of fever. Moderate COVID-19: included adolescent or adult with clinical signs of  pneumonia (fever, cough, dyspnea, fast breathing) but no signs of severe pneumonia, including  SpO2 ≥ 90% on room air. | N/A | comparable baseline severity indices between groups (controlled) |
| 99 | Singapore | from February 9, 2020 to May 31, 2020 | Inclusion:  - confirmed by PCR on nasopharyngeal swab who were admitted to our center and received treatment for COVID-19.  Exclusion:  - Those who were on supportive care only, on invasive mechanical ventilation prior to treatment or those admissions not related to respiratory illness | Clinical conditions of the patients with COVID-19 included at initiation of treatment were categorized into 3 stages: COVID-19 without pneumonia (Stage 1), COVID-19 related pneumonia not requiring supplemental oxygen (Stage 2) and COVID-19 related pneumonia requiring supplemental oxygen (Stage 3). Chest radiograph was the primary modality used in  95 the assessment for pneumonia | N/A | covariate adjustment in regression models |
| 100 | Pakistan | from 10  April 2020 to 31 May 2020. | Inclusion:  - admitted patients from both genders with Mild confirmed COVID-19 in Pakistan Emirates military Hospital (PEMH) | Severity of  disease was defined as per criteria designed by WHO  Mild disease meant Patients with  uncomplicated upper respiratory tract viral infection having non-specific symptoms such as low-grade fever (fever < 100F for < 3 days), fatigue, body aches, cough (with or without sputum  production), anorexia, muscle pain, sore throat, nasal congestion, anosmia, headache and rarely  diarrhea, nausea, and vomiting | N/A | RCT |
| 101 | Netherlands | Enrollment began on April 8 2020 | Inclusion:  - admitted to a study  site for COVID-19 and had clinical COVID-19 disease proven by a positive SARS-CoV-2 reverse  transcriptase polymerase chain reaction (RT-PCR) test (laboratory confirmed) in the previous 96 hours  Exclusion:  - Patients with  a documented IgA deficiency or on mechanical ventilation for >96 hours | ordinal 8-point WHO COVID-19 disease severity scale | N/A | RCT |
| 102 | Italy | between February 20th and May 10th | Inclusion:  - Laboratory confirmed COVID-19, admitted in hospital located in Brescia, Italy | not specified in script but presumably respiratory status | N/A | PSM and multivariate adjustment |
| 103 | Multinational | March 25 to  June 5, 2020 | Inclusion:  patients were eligible for the study if they had clinically suspected or laboratory confirmed SARS-CoV-2 infection and no medical history that might, in the opinion of the attending clinicians, put the patient at significant risk if they were to participate in the trial | not specified but presumably respiratory status | N/A | RCT |
| 104 | Spain | between January 31 and April 23, 2020 | Inclusion:  - subjects included in the HM Hospitals cohort—a  multicenter cohort of patients admitted to any of the 17 hospitals in the HM Group  in Madrid and diagnosed with COVID-19 from January 31st to April 23rd, with laboratory confirmed COVID-19 | clinical severity (blood pressure, heart rate, total lymphocyte and neutrophil count, lactate dehydrogenase, alanine aminotransferase, urea, D-dimers, and CRP) | N/A | IPTW adjusted analysis |
| 105 | China | From February 18 to March 30, 2020 | Inclusion:  - inhabitants of the Wuhan area, and  diagnosed with mild or moderate types of COVID-19 based on the Chinese Diagnosis  and Treatment Protocol for Novel Coronavirus Pneumonia (5th -7th Editions), and laboratory confirmed COVID-19 or lung changes characteristic of COVID-19 on  computerized tomography (CT) scan of the chest. At admission, all patients had SaO2  (oxygen saturation) > 93% at room temperature. | moderate: cases meeting the diagnostic criteria for  COVID-19; 2. pneumonia confirmed by chest CT scan; 3. absence of severe hypoxia  or dyspnea (SaO2>93%, PaO2/FiO2>300 mmHg, respiratory rate <30/min) | N/A | RCT |
| 106 | U. S | between March 1, and May 5, 2020 | Inclusion:  - including all consecutive COVID-19 patients, admitted to the  medical center who were either discharged from the medical center or expired between March 1, 2020, and May 5, 2020,  was performed. In addition, patients were required to exhibit severe disease at time of administration. | Severe disease was  defined as an SpO2 ≤ 94% on room air, requiring supplemental oxygen, or requiring invasive or non-invasive mechanical  ventilation | N/A | PSM |
| 107 | U. S | between May 1 and June  16, 2020, | Inclusion:  - laboratory confirmed COVID-19, moderate to severe hypoxia (sPO2 <96% on > 4 liters O2 by nasal cannula). This corresponds to FiO2 of 37% to maintain  a PaO2 of 90 mm Hg (alveolar-arterial [A-a] gradient of 120 mm Hg or PaO2/FiO2 243)  Exclusion:  - mechanical ventilation | refer to inclusion criteria | N/A | RCT |
| 108 | Italy | between February 27 and April 24, 2020 | inclusion criteria  1)laboratory confirmed COVID-19 diagnosis; 2) PaO2:FiO2 <250 mmHg; 3) bilateral infiltrates; 4) CRP >100 mg/L; and/or 5) diagnosis of acute respiratory distress syndrome (ARDS)  Exclusion:  - on long-term  oxygen or home mechanical ventilation | refer to inclusion criteria | N/A | multivariable adjusted model |
| 109 | U.S/Canada | depending on the sub-trial | Inclusion:  Exposure to a COVID19 case within 4 days as either a household contact or occupational exposure, OR  Symptomatic COVID19 case with confirmed diagnosis within 4 days of symptom onset OR symptomatic high-risk exposure with known COVID19 contact and within 4 days of symptom onset | refer to inclusion criteria | N/A | RCT |
| 110 | Cuba | enrollment began  March 28,  2020 | Any gender and skin color  SARS-CoV2 infection diagnosed by PCR-RT or rapid test to the COVID 19 positive • Confirmed Multifocal interstitial pneumonia • Need for oxygen therapy to maintain saturation of O2>93% • Worsening of lung involvement, defined as one of the following criteria: o Worsening oxygen saturation >3 percentage points or decrease in PaO2 >10%, with FiO2 stable in the last 24 hours. o Need to increase FiO2 in order to maintain a stable SO2 or new need for mechanical installation in the last 24 hrs. o Increase in the number and/or extent of consolidation lung areas • Express willfulness of the patient, family member or impartial witness Alternatively, itolizumab may be used in patients suspecting macrophage activation syndrome, according to the following criteria: Need for oxygen therapy not less than 6L/min plus one of the following conditions: • Wheezing or irregular speech (cannot quickly count to 20 after deep inspiration) • Respiratory frequency greater than 22 breaths per minute with oxygen therapy at 6L/min • PO2: Partial arterial oxygen pressure <65 mm Hg • Worsening of the radiological image • Fever ≥ 38oC • Reduction of initial values of hemoglobin, platelets or neutrophils or Hb< 90 g/L, platelets <100 x109/L, neutrophils <1 x109/L or leukocytes < 4 x109/L. • Decreased erythrocyte sedimentation in mismatch with PCR (low erythrocyte sedimentation and PCR increases or does not change) • Increased initial value of triglycerides or triglycerides greater than 3 mmol/L. • Increased initial ferritin value from 500 ng/ml or absolute ferritin value ≥ 2000 ng/ml. • Transaminase aspartate-aminotransferase ≥30 IU/L • Increase in dimer D • Fibrinogen < 2.5 g/L • Onset of neurological manifestations | refer to inclusion criteria | N/A | contemporary matched patient |

Abbreviations: HQ: Hydroxychlroquine, AZ: Azithromycin, CQ: Chloroquine, LPV/R: lopinavir-ritonavir, IQR: interquartile range, RCT: randomized controlled trial, NEWS2: National Early Warning Score 2, SOFA: Sequential Organ Failure Assessment, IFN: interferon, PO: Per os, IV: Intravenous, f/u: follow-up, QTc: QT interval corrected for heart rate. RR: respiratory rate, SPO2: Oxygen saturation, ICU: Intensive Care Unit, HR: heart rate, IVIG: Intravenous immunoglobulin, ECMO: Extracorporeal membrane oxygenation, LMWH: low molecular weight heparin, Toci: Tocilizumb, U.S: United States, NY: New York, NY: New Jersey. WHO: World Health Organization; SOC: Standard of Care; Methylprednisolone; PSM: Propensity score matching; RS: Retrospective study; IPTW: Inverse probability treatment weighting; DRV/c: Darunavir/Cobicistat; CM: Chinese medicine; RBV: Ribavirin; IFN: Interferon; IFN: Interferon-alpha(IFN-a); UFV: umifenovir; SOF/DCV: Sofosbuvir/daclatasvir; IVM: Ivermectin; CP: Convalescent plasma; FPV:Favipiravir; NVF:Novaferon

References:

1. Rosenberg ES, Dufort EM, Udo T, et al. Association of Treatment With Hydroxychloroquine or Azithromycin With In-Hospital Mortality in Patients With COVID-19 in New York State. *JAMA* 2020.

2. Borba MGS, Val FFA, Sampaio VS, et al. Effect of High vs Low Doses of Chloroquine Diphosphate as Adjunctive Therapy for Patients Hospitalized With Severe Acute Respiratory Syndrome Coronavirus 2 (SARS-CoV-2) Infection: A Randomized Clinical Trial. *JAMA Network Open* 2020; **3**(4): e208857-e.

3. Geleris J, Sun Y, Platt J, et al. Observational Study of Hydroxychloroquine in Hospitalized Patients with Covid-19. *New England Journal of Medicine* 2020.

4. Cao B, Wang Y, Wen D, et al. A Trial of Lopinavir–Ritonavir in Adults Hospitalized with Severe Covid-19. *New England Journal of Medicine* 2020; **382**(19): 1787-99.

5. Hung IF-N, Lung K-C, Tso EY-K, et al. Triple combination of interferon beta-1b, lopinavir&#x2013;ritonavir, and ribavirin in the treatment of patients admitted to hospital with COVID-19: an open-label, randomised, phase 2 trial. *The Lancet* 2020; **395**(10238): 1695-704.

6. Wang Y, Zhang D, Du G, et al. Remdesivir in adults with severe COVID-19: a randomised, double-blind, placebo-controlled, multicentre trial. *The Lancet* 2020; **395**(10236): 1569-78.

7. Cai Q, Yang M, Liu D, et al. Experimental Treatment with Favipiravir for COVID-19: An Open-Label Control Study. *Engineering* 2020.

8. Boulware DR, Pullen MF, Bangdiwala AS, et al. A Randomized Trial of Hydroxychloroquine as Postexposure Prophylaxis for Covid-19. *New England Journal of Medicine* 2020.

9. Bessière F, Roccia H, Delinière A, et al. Assessment of QT Intervals in a Case Series of Patients With Coronavirus Disease 2019 (COVID-19) Infection Treated With Hydroxychloroquine Alone or in Combination With Azithromycin in an Intensive Care Unit. *JAMA Cardiology* 2020.

10. Mercuro NJ, Yen CF, Shim DJ, et al. Risk of QT Interval Prolongation Associated With Use of Hydroxychloroquine With or Without Concomitant Azithromycin Among Hospitalized Patients Testing Positive for Coronavirus Disease 2019 (COVID-19). *JAMA Cardiology* 2020.

11. Li Y, Xie Z, Lin W, et al. Efficacy and safety of lopinavir/ritonavir or arbidol in adult patients with mild/moderate COVID-19: an exploratory randomized controlled trial. *Med* 2020.

12. Tang W, Cao Z, Han M, et al. Hydroxychloroquine in patients with mainly mild to moderate coronavirus disease 2019: open label, randomised controlled trial. *BMJ* 2020; **369**: m1849.

13. Mahévas M, Tran V-T, Roumier M, et al. Clinical efficacy of hydroxychloroquine in patients with covid-19 pneumonia who require oxygen: observational comparative study using routine care data. *BMJ* 2020; **369**: m1844.

14. Lu X, Chen T, Wang Y, Wang J, Yan F. Adjuvant corticosteroid therapy for critically ill patients with COVID-19. *Critical Care* 2020; **24**(1): 241.

15. Chen X, Zhang Y, Zhu B, et al. Associations of clinical characteristics and antiviral drugs with viral RNA clearance in patients with COVID-19 in Guangzhou, China: a retrospective cohort study. *medRxiv* 2020: 2020.04.09.20058941.

16. Shao Z, Feng Y, Zhong L, et al. Clinical efficacy of intravenous immunoglobulin therapy in critical patients with COVID-19: A multicenter retrospective cohort study. *medRxiv* 2020: 2020.04.11.20061739.

17. Deng L, Li C, Zeng Q, et al. Arbidol combined with LPV/r versus LPV/r alone against Corona Virus Disease 2019: A retrospective cohort study. *J Infect* 2020.

18. Wang Y, Jiang W, He Q, et al. A retrospective cohort study of methylprednisolone therapy in severe patients with COVID-19 pneumonia. *Signal Transduction and Targeted Therapy* 2020; **5**(1): 57.

19. Saleh M, Gabriels J, Chang D, et al. The Effect of Chloroquine, Hydroxychloroquine and Azithromycin on the Corrected QT Interval in Patients with SARS-CoV-2 Infection. *Circulation: Arrhythmia and Electrophysiology*; **0**(0).

20. Li L, Zhang W, Hu Y, et al. Effect of Convalescent Plasma Therapy on Time to Clinical Improvement in Patients With Severe and Life-threatening COVID-19: A Randomized Clinical Trial. *JAMA* 2020.

21. Beigel JH, Tomashek KM, Dodd LE, et al. Remdesivir for the Treatment of Covid-19 — Preliminary Report. *New England Journal of Medicine* 2020.

22. Goldman JD, Lye DCB, Hui DS, et al. Remdesivir for 5 or 10 Days in Patients with Severe Covid-19. *New England Journal of Medicine* 2020.

23. Zheng F, Zhou Y, Zhou Z, et al. A Novel Protein Drug, Novaferon, as the Potential Antiviral Drug for COVID-19. *medRxiv* 2020: 2020.04.24.20077735.

24. Zhong M, Sun A, Xiao T, et al. A Randomized, Single-blind, Group sequential, Active-controlled Study to evaluate the clinical efficacy and safety of α-Lipoic acid for critically ill patients with coronavirus disease 2019（COVID-19）. *medRxiv* 2020: 2020.04.15.20066266.

25. Lou Y, Liu L, Qiu Y. Clinical Outcomes and Plasma Concentrations of Baloxavir Marboxil and Favipiravir in COVID-19 Patients: an Exploratory Randomized, Controlled Trial. *medRxiv* 2020: 2020.04.29.20085761.

26. Ramireddy A, Chugh HS, Reinier K, et al. Experience with Hydroxychloroquine and Azithromycin in the COVID-19 Pandemic: Implications for QT Interval Monitoring. *medRxiv* 2020: 2020.04.22.20075671.

27. Freedberg DE, Conigliaro J, Wang TC, et al. Famotidine Use is Associated with Improved Clinical Outcomes in Hospitalized COVID-19 Patients: A Propensity Score Matched Retrospective Cohort Study. *Gastroenterology*.

28. Magagnoli J, Narendran S, Pereira F, et al. Outcomes of hydroxychloroquine usage in United States veterans hospitalized with Covid-19. *medRxiv* 2020: 2020.04.16.20065920.

29. Chen Z, Hu J, Zhang Z, et al. Efficacy of hydroxychloroquine in patients with COVID-19: results of a randomized clinical trial. *medRxiv* 2020: 2020.03.22.20040758.

30. Carlucci P, Ahuja T, Petrilli CM, Rajagopalan H, Jones S, Rahimian J. Hydroxychloroquine and azithromycin plus zinc vs hydroxychloroquine and azithromycin alone: outcomes in hospitalized COVID-19 patients. *medRxiv* 2020: 2020.05.02.20080036.

31. Lan X, Shao C, Zeng X, Wu Z, Xu Y. Lopinavir-ritonavir alone or combined with arbidol in the treatment of 73 hospitalized patients with COVID-19: a pilot retrospective study. *medRxiv* 2020: 2020.04.25.20079079.

32. Huang M, Li M, Xiao F, et al. Preliminary evidence from a multicenter prospective observational study of the safety and efficacy of chloroquine for the treatment of COVID-19. *medRxiv* 2020: 2020.04.26.20081059.

33. Wu J, Huang J, Zhu G, et al. Systemic corticosteroids show no benefit in severe and critical COVID-19 patients in Wuhan, China: A retrospective cohort study. *medRxiv* 2020: 2020.05.11.20097709.

34. Kim MS, Jang S-W, Park Y-K, et al. Treatment Response to Hydroxychloroquine, Lopinavir/Ritonavir, and Antibiotics for Moderate COVID 19: A First Report on the Pharmacological Outcomes from South Korea. *medRxiv* 2020: 2020.05.13.20094193.

35. Shi C, Wang C, Wang H, et al. The potential of low molecular weight heparin to mitigate cytokine storm in severe COVID-19 patients: a retrospective clinical study. *medRxiv* 2020: 2020.03.28.20046144.

36. Feng Z, Li J, Yao S, et al. The Use of Adjuvant Therapy in Preventing Progression to Severe Pneumonia in Patients with Coronavirus Disease 2019: A Multicenter Data Analysis. *medRxiv* 2020: 2020.04.08.20057539.

37. Bian H, Zheng Z-H, Wei D, et al. Meplazumab treats COVID-19 pneumonia: an open-labelled, concurrent controlled add-on clinical trial. *medRxiv* 2020: 2020.03.21.20040691.

38. Singh S, Khan A, Chowdhry M, Chatterjee A. Outcomes of Hydroxychloroquine Treatment Among Hospitalized COVID-19 Patients in the United States- Real-World Evidence From a Federated Electronic Medical Record Network. *medRxiv* 2020: 2020.05.12.20099028.

39. Ip A, Berry DA, Hansen E, et al. Hydroxychloroquine and tocilizumab therapy in COVID-19 patients—An observational study. *PLOS ONE* 2020; **15**(8): e0237693.

40. Campochiaro C, Della-Torre E, Cavalli G, et al. Efficacy and safety of tocilizumab in severe COVID-19 patients: a single-centre retrospective cohort study. *European Journal of Internal Medicine* 2020; **76**: 43-9.

41. Cao Y, Wei J, Zou L, et al. Ruxolitinib in treatment of severe coronavirus disease 2019 (COVID-19): A multicenter, single-blind, randomized controlled trial. *Journal of Allergy and Clinical Immunology* 2020.

42. Huet T, Beaussier H, Voisin O, et al. Anakinra for severe forms of COVID-19: a cohort study. *The Lancet Rheumatology*.

43. Capra R, De Rossi N, Mattioli F, et al. Impact of low dose tocilizumab on mortality rate in patients with COVID-19 related pneumonia. *European Journal of Internal Medicine* 2020; **76**: 31-5.

44. Yuan M, Xu X, Xia D, et al. Effects of Corticosteroid Treatment for Non-Severe COVID-19 Pneumonia: A Propensity Score-Based Analysis. *Shock* 9000; **Publish Ahead of Print**.

45. Fernandez-Cruz A, Ruiz-Antoran B, Munoz-Gomez A, et al. IMPACT OF GLUCOCORTICOID TREATMENT IN SARS-COV-2 INFECTION MORTALITY: A RETROSPECTIVE CONTROLLED COHORT STUDY. *medRxiv* 2020: 2020.05.22.20110544.

46. Moreno Garcia E, Rico Caballero V, Albiach L, et al. Tocilizumab is associated with reduction of the risk of ICU admission and mortality in patients with SARS-CoV-2 infection. *medRxiv* 2020: 2020.06.05.20113738.

47. Somers EC, Eschenauer GA, Troost JP, et al. Tocilizumab for treatment of mechanically ventilated patients with COVID-19. *medRxiv* 2020: 2020.05.29.20117358.

48. Davoudi-Monfared E, Rahmani H, Khalili H, et al. Efficacy and safety of interferon beta-1a in treatment of severe COVID-19: A randomized clinical trial. *medRxiv* 2020: 2020.05.28.20116467.

49. Rossi B, Nguyen LS, Zimmermann P, et al. Effect of tocilizumab in hospitalized patients with severe pneumonia COVID-19: a cohort study. *medRxiv* 2020: 2020.06.06.20122341.

50. Skipper CP, Pastick KA, Engen NW, et al. Hydroxychloroquine in Nonhospitalized Adults With Early COVID-19: A Randomized Trial. *Ann Intern Med* 2020.

51. Miller J, Bruen C, Schnaus M, et al. Auxora versus standard of care for the treatment of severe or critical COVID-19 pneumonia: results from a randomized controlled trial. *Critical Care* 2020; **24**(1): 502.

52. Lecronier M, Beurton A, Burrel S, et al. Comparison of hydroxychloroquine, lopinavir/ritonavir, and standard of care in critically ill patients with SARS-CoV-2 pneumonia: an opportunistic retrospective analysis. *Critical Care* 2020; **24**(1): 418.

53. Ivashchenko AA, Dmitriev KA, Vostokova NV, et al. AVIFAVIR for Treatment of Patients with Moderate COVID-19: Interim Results of a Phase II/III Multicenter Randomized Clinical Trial. *Clinical Infectious Diseases* 2020.

54. Nelson BC, Laracy J, Shoucri S, et al. Clinical Outcomes Associated with Methylprednisolone in Mechanically Ventilated Patients with COVID-19. *Clinical Infectious Diseases* 2020.

55. Mitjà O, Corbacho-Monné M, Ubals M, et al. Hydroxychloroquine for Early Treatment of Adults with Mild Covid-19: A Randomized-Controlled Trial. *Clinical Infectious Diseases* 2020.

56. Jeronimo CMP, Farias MEL, Val FFA, et al. Methylprednisolone as Adjunctive Therapy for Patients Hospitalized With COVID-19 (Metcovid): A Randomised, Double-Blind, Phase IIb, Placebo-Controlled Trial. *Clinical Infectious Diseases* 2020.

57. Olender SA, Perez KK, Go AS, et al. Remdesivir for Severe COVID-19 versus a Cohort Receiving Standard of Care. *Clinical Infectious Diseases* 2020.

58. Arshad S, Kilgore P, Chaudhry ZS, et al. Treatment with hydroxychloroquine, azithromycin, and combination in patients hospitalized with COVID-19. *International Journal of Infectious Diseases* 2020; **97**: 396-403.

59. Biran N, Ip A, Ahn J, et al. Tocilizumab among patients with COVID-19 in the intensive care unit: a multicentre observational study. *The Lancet Rheumatology*.

60. Dexamethasone in Hospitalized Patients with Covid-19 — Preliminary Report. *New England Journal of Medicine* 2020.

61. Cavalcanti AB, Zampieri FG, Rosa RG, et al. Hydroxychloroquine with or without Azithromycin in Mild-to-Moderate Covid-19. *New England Journal of Medicine* 2020.

62. Ma Q, Qi D, Deng XY, et al. Corticosteroid therapy for patients with severe novel Coronavirus disease 2019. *Eur Rev Med Pharmacol Sci* 2020; **24**(15): 8194-201.

63. Hu Z, Lv Y, Xu C, et al. Clinical Use of Short-Course and Low-Dose Corticosteroids in Patients With Non-severe COVID-19 During Pneumonia Progression. *Frontiers in Public Health* 2020; **8**(355).

64. Abolghasemi H, Eshghi P, Cheraghali AM, et al. Clinical efficacy of convalescent plasma for treatment of COVID-19 infections: Results of a multicenter clinical study. *Transfus Apher Sci* 2020: 102875-.

65. Chen J, Xia L, Liu L, et al. Antiviral Activity and Safety of Darunavir/Cobicistat for the Treatment of COVID-19. *Open Forum Infect Dis* 2020; **7**(7): ofaa241-ofaa.

66. Cantini F, Niccoli L, Nannini C, et al. Beneficial impact of Baricitinib in COVID-19 moderate pneumonia; multicentre study. *J Infect* 2020: S0163-4453(20)30433-3.

67. Wang J-B, Wang Z-X, Jing J, et al. Exploring an Integrative Therapy for Treating COVID-19: A Randomized Controlled Trial. *Chin J Integr Med* 2020; **26**(9): 648-55.

68. Ramiro S, Mostard RLM, Magro-Checa C, et al. Historically controlled comparison of glucocorticoids with or without tocilizumab versus supportive care only in patients with COVID-19-associated cytokine storm syndrome: results of the CHIC study. *Annals of the Rheumatic Diseases* 2020; **79**(9): 1143.

69. Karolyi M, Pawelka E, Mader T, et al. Hydroxychloroquine versus lopinavir/ritonavir in severe COVID-19 patients : Results from a real-life patient cohort. *Wien Klin Wochenschr* 2020: 1-8.

70. Lian N, Xie H, Lin S, Huang J, Zhao J, Lin Q. Umifenovir treatment is not associated with improved outcomes in patients with coronavirus disease 2019: a retrospective study. *Clin Microbiol Infect* 2020; **26**(7): 917-21.

71. Hao SR, Yan R, Zhang SY, et al. Interferon-α2b spray inhalation did not shorten virus shedding time of SARS-CoV-2 in hospitalized patients: a preliminary matched case-control study. *J Zhejiang Univ Sci B* 2020; **21**(8): 628-36.

72. Canziani LM, Trovati S, Brunetta E, et al. Interleukin-6 receptor blocking with intravenous tocilizumab in COVID-19 severe acute respiratory distress syndrome: A retrospective case-control survival analysis of 128 patients. *Journal of Autoimmunity* 2020: 102511.

73. Fang X, Mei Q, Yang T, et al. Low-dose corticosteroid therapy does not delay viral clearance in patients with COVID-19. *J Infect* 2020; **81**(1): 147-78.

74. Lopinavir-ritonavir versus hydroxychloroquine for viral clearance and clinical improvement in patients with mild to moderate coronavirus disease 2019 FAU - Kim, Ji-Won FAU - Kim, Eun Jin FAU - Kwon, Hyun Hee FAU - Jung, Chi Young FAU - Kim, Kyung Chan FAU - Choe, Jung-Yoon FAU - Hong, Hyo-Lim. *Korean J Intern Med* 2020; **0**(0): 0-.

75. Della-Torre E, Campochiaro C, Cavalli G, et al. Interleukin-6 blockade with sarilumab in severe COVID-19 pneumonia with systemic hyperinflammation: an open-label cohort study. *Annals of the Rheumatic Diseases* 2020: annrheumdis-2020-218122.

76. Sanz Herrero F, Puchades Gimeno F, Ortega García P, Ferrer Gómez C, Ocete Mochón MD, García Deltoro M. Methylprednisolone added to tocilizumab reduces mortality in SARS-CoV-2 pneumonia: An observational study. *Journal of Internal Medicine*; **n/a**(n/a).

77. Huang Y-Q, Tang S-Q, Xu X-L, et al. No Statistically Apparent Difference in Antiviral Effectiveness Observed Among Ribavirin Plus Interferon-Alpha, Lopinavir/Ritonavir Plus Interferon-Alpha, and Ribavirin Plus Lopinavir/Ritonavir Plus Interferon-Alpha in Patients With Mild to Moderate Coronavirus Disease 2019: Results of a Randomized, Open-Labeled Prospective Study. *Frontiers in Pharmacology* 2020; **11**(1071).

78. Wang N, Zhan Y, Zhu L, et al. Retrospective Multicenter Cohort Study Shows Early Interferon Therapy Is Associated with Favorable Clinical Responses in COVID-19 Patients. *Cell Host & Microbe* 2020.

79. Tong S, Su Y, Yu Y, et al. Ribavirin therapy for severe COVID-19: a retrospective cohort study. *International journal of antimicrobial agents* 2020; **56**(3): 106114-.

80. Rossotti R, Travi G, Ughi N, et al. Safety and efficacy of anti-il6-receptor tocilizumab use in severe and critical patients affected by coronavirus disease 2019: A comparative analysis. *Journal of Infection* 2020.

81. Spinner CD, Gottlieb RL, Criner GJ, et al. Effect of Remdesivir vs Standard Care on Clinical Status at 11 Days in Patients With Moderate COVID-19: A Randomized Clinical Trial. *JAMA* 2020.

82. De Luca G, Cavalli G, Campochiaro C, et al. GM-CSF blockade with mavrilimumab in severe COVID-19 pneumonia and systemic hyperinflammation: a single-centre, prospective cohort study. *The Lancet Rheumatology* 2020; **2**(8): e465-e73.

83. Guaraldi G, Meschiari M, Cozzi-Lepri A, et al. Tocilizumab in patients with severe COVID-19: a retrospective cohort study. *The Lancet Rheumatology* 2020; **2**(8): e474-e84.

84. Sadeghi A, Ali Asgari A, Norouzi A, et al. Sofosbuvir and daclatasvir compared with standard of care in the treatment of patients admitted to hospital with moderate or severe coronavirus infection (COVID-19): a randomized controlled trial. *Journal of Antimicrobial Chemotherapy* 2020.

85. Abbaspour Kasgari H, Moradi S, Shabani AM, et al. Evaluation of the efficacy of sofosbuvir plus daclatasvir in combination with ribavirin for hospitalized COVID-19 patients with moderate disease compared with standard care: a single-centre, randomized controlled trial. *Journal of Antimicrobial Chemotherapy* 2020.

86. Deftereos SG, Giannopoulos G, Vrachatis DA, et al. Effect of Colchicine vs Standard Care on Cardiac and Inflammatory Biomarkers and Clinical Outcomes in Patients Hospitalized With Coronavirus Disease 2019: The GRECCO-19 Randomized Clinical Trial. *JAMA Network Open* 2020; **3**(6): e2013136-e.

87. Eslami G, Mousaviasl S, Radmanesh E, et al. The impact of sofosbuvir/daclatasvir or ribavirin in patients with severe COVID-19. *Journal of Antimicrobial Chemotherapy* 2020.

88. Salazar E, Christensen PA, Graviss EA, et al. Treatment of COVID-19 Patients with Convalescent Plasma Reveals a Signal of Significantly Decreased Mortality. *The American Journal of Pathology*.

89. Klopfenstein T, Zayet S, Lohse A, et al. Impact of Tocilizumab on mortality and/or invasive mechanical ventilation requirement in a cohort of 206 COVID-19 patients. *International Journal of Infectious Diseases* 2020.

90. Li Y, Zhou X, Li T, et al. Corticosteroid prevents COVID-19 progression within its therapeutic window: a multicentre, proof-of-concept, observational study. *Emerging Microbes & Infections* 2020; **9**(1): 1869-77.

91. Mitja O, Ubals M, Corbacho M, et al. A Cluster-Randomized Trial of Hydroxychloroquine as Prevention of Covid-19 Transmission and Disease. *medRxiv* 2020: 2020.07.20.20157651.

92. Lopes MIF, Bonjorno LP, Giannini MC, et al. Beneficial effects of colchicine for moderate to severe COVID-19: an interim analysis of a randomized, double-blinded, placebo controlled clinical trial. *medRxiv* 2020: 2020.08.06.20169573.

93. Chen C-P, Lin Y-C, Chen T-C, et al. A Multicenter, randomized, open-label, controlled trial to evaluate the efficacy and tolerability of hydroxychloroquine and a retrospective study in adult patients with mild to moderate Coronavirus disease 2019 (COVID-19). *medRxiv* 2020: 2020.07.08.20148841.

94. Majmundar M, Kansara T, Lenik JM, et al. Efficacy of Corticosteroids in Non-Intensive Care Unit Patients with COVID-19 Pneumonia from the New York Metropolitan region. *medRxiv* 2020: 2020.07.02.20145565.

95. Corral L, Bahamonde A, Arnaiz delas Revillas F, et al. GLUCOCOVID: A controlled trial of methylprednisolone in adults hospitalized with COVID-19 pneumonia. *medRxiv* 2020: 2020.06.17.20133579.

96. Sbidian E, Josse J, Lemaitre G, et al. Hydroxychloroquine with or without azithromycin and in-hospital mortality or discharge in patients hospitalized for COVID-19 infection: a cohort study of 4,642 in-patients in France. *medRxiv* 2020: 2020.06.16.20132597.

97. Rajter JC, Sherman M, Fatteh N, Vogel F, Sacks J, Rajter J-J. ICON (Ivermectin in COvid Nineteen) study: Use of Ivermectin is Associated with Lower Mortality in Hospitalized Patients with COVID19. *medRxiv* 2020: 2020.06.06.20124461.

98. Gorial FI, Mashhadani S, Sayaly HM, et al. Effectiveness of Ivermectin as add-on Therapy in COVID-19 Management (Pilot Trial). *medRxiv* 2020: 2020.07.07.20145979.

99. Ooi ST, Parthasarathy P, Lin Y, et al. Adjunctive Corticosteroids for COVID-19: A Retrospective Cohort Study. *medRxiv* 2020: 2020.07.18.20157008.

100. kamran sm, Mirza ZeH, Naseem A, et al. Clearing the fog: Is HCQ effective in reducing COVID-19 progression: A randomized controlled trial. *medRxiv* 2020: 2020.07.30.20165365.

101. Gharbharan A, Jordans CCE, GeurtsvanKessel C, et al. Convalescent Plasma for COVID-19. A randomized clinical trial. *medRxiv* 2020: 2020.07.01.20139857.

102. Albani F, Fusina F, Granato E, et al. Effect of corticosteroid treatment on 1376 hospitalized COVID-19 patients. A cohort study. *medRxiv* 2020: 2020.07.17.20155994.

103. Horby P, Mafham M, Linsell L, et al. Effect of Hydroxychloroquine in Hospitalized Patients with COVID-19: Preliminary results from a multi-centre, randomized, controlled trial. *medRxiv* 2020: 2020.07.15.20151852.

104. Martinez-Sanz J, Muriel A, Ron R, et al. Effects of Tocilizumab on Mortality in Hospitalized Patients with COVID-19: A Multicenter Cohort Study. *medRxiv* 2020: 2020.06.08.20125245.

105. Chen L, Zhang Z-y, Fu J-g, et al. Efficacy and safety of chloroquine or hydroxychloroquine in moderate type of COVID-19: a prospective open-label randomized controlled study. *medRxiv* 2020: 2020.06.19.20136093.

106. Tsai A, Diawara O, Nahass RG, Brunetti L. Impact of tocilizumab administration on mortality in severe COVID-19. *medRxiv* 2020: 2020.07.30.20114959.

107. Sakoulas G, Geriak M, Kullar R, et al. Intravenous Immunoglobulin (IVIG) Significantly Reduces Respiratory Morbidity in COVID-19 Pneumonia: A Prospective Randomized Trial. *medRxiv* 2020: 2020.07.20.20157891.

108. Salton F, Confalonieri P, Santus P, et al. Prolonged low-dose methylprednisolone in patients with severe COVID-19 pneumonia. *medRxiv* 2020: 2020.06.17.20134031.

109. Lofgren SMM, Nicol MR, Bangdiwala AS, et al. Safety of Hydroxychloroquine among Outpatient Clinical Trial Participants for COVID-19. *medRxiv* 2020: 2020.07.16.20155531.

110. Ramos-Suzarte M, Diaz Y, Martin Y, et al. Use of a humanized anti-CD6 monoclonal antibody (itolizumab) in elderly patients with moderate COVID-19. *medRxiv* 2020: 2020.07.24.20153833.
