# Supplementary material for: Comparative efficacy and safety of pharmacological interventions for the treatment of COVID-19: A systematic review and network meta-analysis
Source: PLoS Med. 2020 Dec 30;17(12):e1003501. doi: 10.1371/journal.pmed.1003501 (PMC7794037; doi:10.1371/journal.pmed.1003501)
Supplement: S2 Table — (DOCX) [file pmed.1003501.s003.docx]

**S2 Table. Risk of Bias, NOS, and Jadad evaluations of individual studies**

- 1. **Randomized controlled trials**


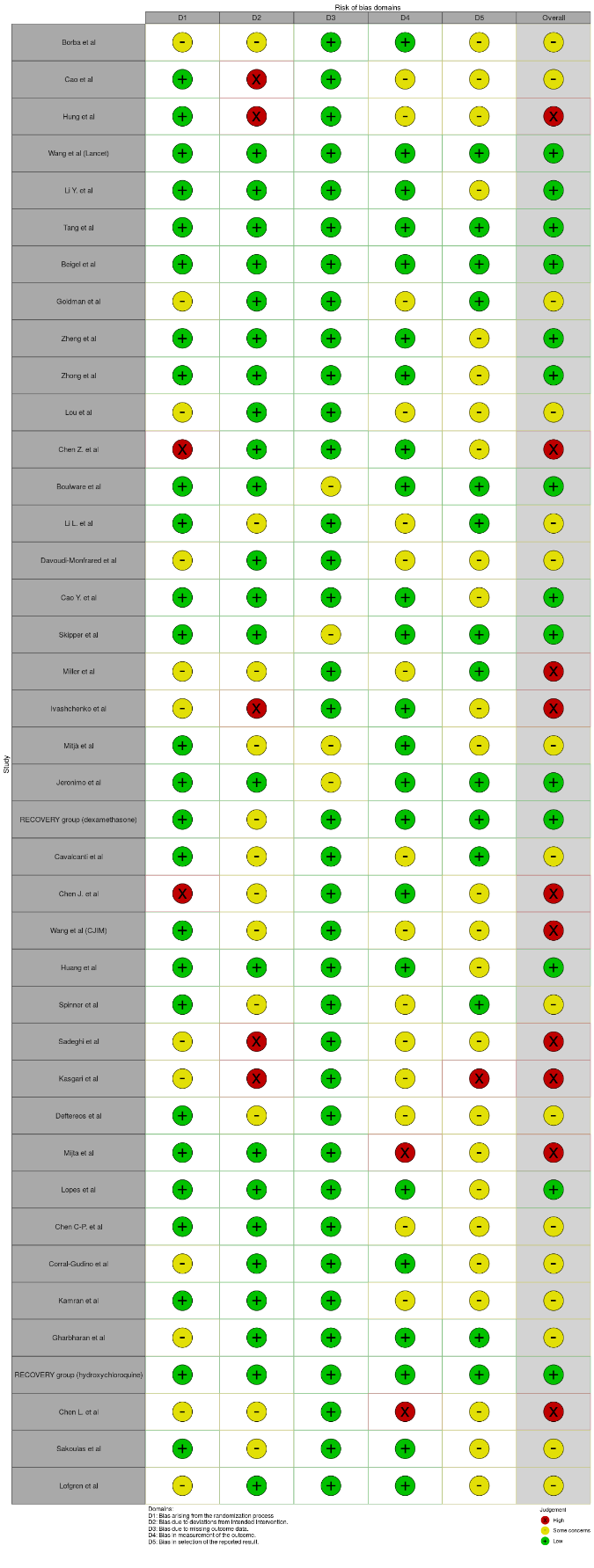

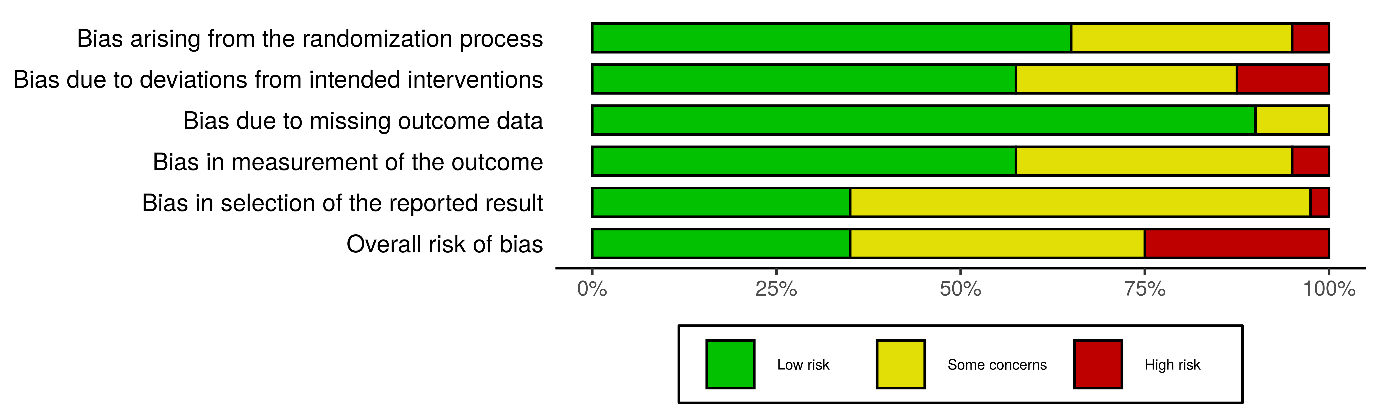


- 1. **Non-randomized observational studies**


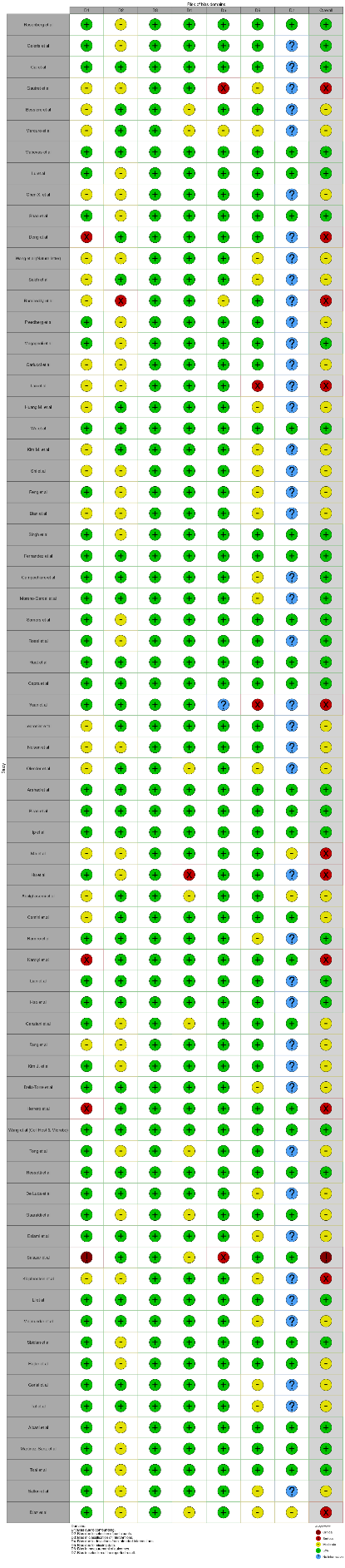

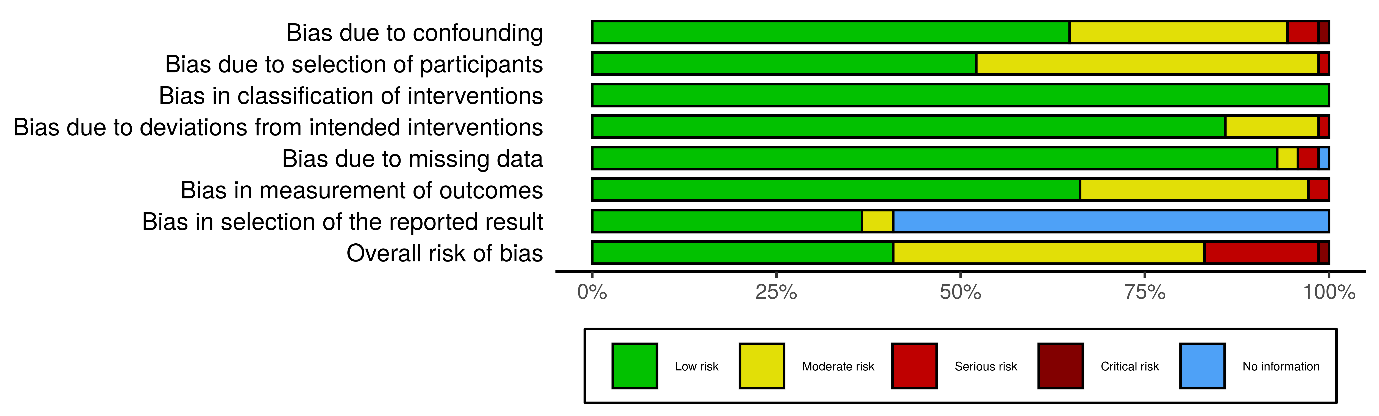


1. **Risk of bias tables for included studies**

Risk of bias (RoB) was assessed using the Risk of Bias 2 (RoB2) tool for randomized studies and the Risk of Bias In Nonrandomized Studies (ROBINS-I) tool for nonrandomized studies. Although the RoB2 and ROBINS-I template provided by the authors of the tools were adhered to for the most part, certain deviations from their guidelines were made in the context of this specific network meta-analysis. Specifically, in assessing confounding bias in non-randomized studies, the ROBINS-I template attributes low risk of bias to studies that have no risk for confounding and, if applied directly here, puts most studies in the moderate risk of bias category. In order to generate a RoB gradient with higher resolution, we used our own standards: for confounding bias in nonrandomized studies, “low risk” was attributed to studies that performed sufficient statistical adjustment for potential confounders with methods such as regression modeling or propensity score matching. “Moderate risk” was attributed to studies that only showed baseline characteristics between treatment arms were similar but did not perform adjustments. Studies with weaker designs were judged at “serious” or “critical risk” of bias. Other such deviations were made when the authors deemed necessary, and the support for each judgment is elaborated in the tables below. Readers should keep in mind that while these judgments are partly subjective, they were applied consistently here and justifications are provided.

A sensitivity analysis was performed excluding the studies that were judged to be at overall serious/critical risk of bias.

Rosenberg et al. “Association of treatment with hydroxychloroquine or azithromycin with in-hospital mortality in patients with COVID-19 in New York state”.

ROBINS-I (risk of bias assessment for nonrandomized studies)

| Bias | Author’s judgment | Support for judgment |
| --- | --- | --- |
| **Pre-intervention domains** | | |
| Bias due to confounding  (Confounding) | Low risk | Retrospective cohort study. Cox proportional hazards model was fit for time to death, controlling for treatment group and potential confounders (exhaustive list provided along with justification) |
| Bias in selection of participants into the study  (Selection bias) | Moderate risk | Collected a random sample of inpatients with laboratory confirmed COVID-19 admitted to NYC metropolitan hospitals from an extensive dataset representing 88.2% of the region’s hospitalized patients. Exposure to medication was determined by administration at any point during the data collection period, but enough time was allowed to pass before analysis of results for outcome measures to manifest. |
| **At-intervention domain** | | |
| Bias in classification of interventions  (Information bias) | Low risk | Patients were categorized into 4 treatment groups based on the medication they received during hospitalization. |
| **Post-intervention domains** | | |
| Bias due to deviations from intended interventions  (Confounding) | Low risk | Interventions consist of medication administration, data for which was collected retrospectively. Deviations should be insignificant. |
| Bias due to missing data  (Selection bias) | Low risk | Data were considered complete. |
| Bias in measurement of the outcome  (Information bias) | Low risk | Main outcomes are in-hospital mortality and cardiac arrest. Assessment of outcome is unlikely to have been affected by knowledge of intervention received. |
| Bias in selection of the reported result  (Reporting bias) | Low risk | Mortality is the most important and objective endpoint in severe COVID-19 research, and this is reported as the main outcome measure. |

Borba et al. “Effect of high vs low doses of chloroquine diphosphate as adjuvant therapy for patients hospitalized with severe acute respiratory syndrome coronavirus-2 (SARS-CoV-2) infection: a randomized clinical trial”.

RoB 2 tool

| Bias | Author’s judgement | Support for judgement |
| --- | --- | --- |
| Bias arising from the randomization process | Some concerns | Allocation sequence was subverted and random, but baseline imbalances include older age and higher rate of underlying heart disease in one group (however, it must be noted that an adjusted analysis was conducted). |
| Bias due to deviations from intended interventions | Some concerns | Participants were aware that they were in a trial, and no information is available about deviations from the intended intervention (per protocol analysis could not be conducted). However, there is low probability that participants were analyzed in the wrong group. |
| Bias due to missing outcome data | Low risk | Outcome data were available for all participants. |
| Bias in measurement of the outcome | Low risk | Outcome assessors were blinded to the intervention received by participants. |
| Bias in selection of the reported result | Some concerns | No information. |

Geleris et al. “Observational Study of Hydroxychloroquine in Hospitalized Patients with Covid-19”.

ROBINS-I (risk of bias assessment for nonrandomized studies)

| Bias | Author’s judgment | Support for judgment |
| --- | --- | --- |
| **Pre-intervention domains** | | |
| Bias due to confounding  (Confounding) | Low risk | Observational study. Three propensity-score methods were used to reduce effects of confounding, using a multivariable logistic-regression model that included demographic factors, clinical factors, laboratory tests, and medications. |
| Bias in selection of participants into the study  (Selection bias) | Moderate risk | Included all consecutive adults admitted to the study center who had positive test result for SARS-CoV-2 from nasopharyngeal or oropharyngeal swab specimens. Of those who received hydroxychloroquine, 85.9% received treatment within 48 hours. |
| **At-intervention domain** | | |
| Bias in classification of interventions  (Information bias) | Low risk | Patients were classified by whether or not they received hydroxychloroquine; interventions should be classified correctly for all patients. |
| **Post-intervention domains** | | |
| Bias due to deviations from intended interventions  (Confounding) | Low risk | Interventions consist of medication administration, data for which was collected retrospectively. Deviations should be insignificant. |
| Bias due to missing data  (Selection bias) | Low risk | Missing data is unlikely to have a significant effect on the study. |
| Bias in measurement of the outcome  (Information bias) | Low risk | Main outcome measure consisted of a composite rate of intubation and death. Risk of measurement bias is inherently low, and assessment of outcome is unlikely to have been affected by knowledge of intervention received. |
| Bias in selection of the reported result  (Reporting bias) | No information | Main outcome measure is a composite rate of intubation and death; there is no explicit explanation as to why this measure was used as the primary endpoint. Unclear if the outcome measure was selected a priori. |

Cao B. et al. “A trial of lopinavir-ritonavir in adults hospitalized with severe Covid-19”

RoB 2 tool

| Bias | Author’s judgement | Support for judgement |
| --- | --- | --- |
| Bias arising from the randomization process | Low risk | Randomization sequence, including stratification, prepared by statistician not involved in trial. Interactive web-based response system used for allocation concealment until randomization was finished. |
| Bias due to deviations from intended interventions | High risk | Nearly 14% of the lopinavir-ritonavir group patients were unable to complete treatment course. |
| Bias due to missing outcome data | Low risk | Outcome data were available for all participants. |
| Bias in measurement of the outcome | Some concerns | Open label trial. Knowledge of treatment assignment could have influenced clinical decisions. However, the outcome measure itself was relatively objective as it was based on the treatments received. |
| Bias in selection of the reported result | Some concerns | Multiple outcome measures were collected. Unclear if authors selected which outcome to report as the primary outcome before or after results were obtained; but the difference in results reported in primary/secondary outcome measures should be noted. |

Hung et al. “Triple combination of interferon beta-1b, lopinavir-ritonavir, and ribavirin in the treatment of patients admitted to hospital with COVID-19: an open-label, randomized, phase 2 trial”.

RoB 2 tool

| Bias | Author’s judgement | Support for judgement |
| --- | --- | --- |
| Bias arising from the randomization process | Low risk | Patients assigned serial numbers by the study coordinator, and each serial number linked to a computer-generated randomization list that assigns treatment regimens. |
| Bias due to deviations from intended interventions | High risk | 34 out of 86 patients in the combination treatment arm did not receive interferon beta-1b. |
| Bias due to missing outcome data | Low risk | There was no missing data. |
| Bias in measurement of the outcome | Some concerns | Open label study, but the primary outcome of time from start of treatment to negative nasopharyngeal swab is unlikely to have been affected by the assessors’ knowledge of treatment received. |
| Bias in selection of the reported result | Some concerns | No information. |

Wang et al. “Remdesivir in adults with severe COVID-19: a randomized, double-blind, placebo-controlled, multicentre trial”.

RoB 2 tool

| Bias | Author’s judgement | Support for judgement |
| --- | --- | --- |
| Bias arising from the randomization process | Low risk | Permuted block randomization sequence including stratification was generated by a statistician not involved in the trial using SAS software. |
| Bias due to deviations from intended interventions | Low risk | 12% of patients in the remdesivir group and 5% of patients in the control group discontinued their treatments due to adverse effects, but this is deemed unlikely to affect the outcome. |
| Bias due to missing outcome data | Low risk | No missing data. |
| Bias in measurement of the outcome | Low risk | Assessors were unaware of intervention received by study participants (double-blind). |
| Bias in selection of the reported result | Low risk | The study provides outcome measures based on the study protocol published beforehand. |

Cai et al. “Experimental treatment with favipravir for COVID-19: an open-label cohort study”.

ROBINS-I (risk of bias assessment for nonrandomized studies)

| Bias | Author’s judgment | Support for judgment |
| --- | --- | --- |
| **Pre-intervention domains** | | |
| Bias due to confounding  (Confounding) | Low risk | Patients were not randomized but assigned to each treatment group based on their date of enrolment. The context of the investigation and the results suggest that risk for confounding is low. |
| Bias in selection of participants into the study  (Selection bias) | Low risk | All patients in the enrolment period were screened for eligibility; start of follow-up and start of intervention coincided. |
| **At-intervention domain** | | |
| Bias in classification of interventions  (Information bias) | Low risk | Intervention was lopinavir-ritonavir or favipravir, administered from the beginning of the follow-up period. |
| **Post-intervention domains** | | |
| Bias due to deviations from intended interventions  (Confounding) | Low risk | Deviations are unlikely, and any deviations will reflect usual clinical practice |
| Bias due to missing data  (Selection bias) | Low risk | Data were available from all patients who underwent treatment in each arm. |
| Bias in measurement of the outcome  (Information bias) | Low risk | CT-based outcome assessors (2 diagnostic radiographers) were blinded to treatment allocation; and RT-PCR outcome was unlikely to be influenced by outcome assessors’ knowledge of treatment allocation. |
| Bias in selection of the reported result (Reporting bias) | No information | No information as to whether authors selected outcomes measure a priori. |

Gautret et al. “Hydroxychloroquine and azithromycin as a treatment of COVID-19: results of an open-label non-randomized clinical trial”

ROBINS-I (risk of bias assessment for nonrandomized studies)

| Bias | Author’s judgment | Support for judgment |
| --- | --- | --- |
| **Pre-intervention domains** | | |
| Bias due to confounding  (Confounding) | Moderate risk | Nonrandomized controlled study. Eligible patients in one center were all offered hydroxychloroquine treatment; those who refused, those who were excluded, and those who were treated in other centers were used as controls. No explicit comparison of other potentially confounding variables. |
| Bias in selection of participants into the study  (Selection bias) | Moderate risk | Eligible patients were offered hydroxychloroquine treatment, but it appears that not all eligible patients were included; start of follow-up and start of intervention coincided. |
| **At-intervention domain** | | |
| Bias in classification of interventions  (Information bias) | Low risk | Intervention is well-defined and based solely on information collected at time of intervention. |
| **Post-intervention domains** | | |
| Bias due to deviations from intended interventions  (Confounding) | Low risk | Deviations are unlikely, and any deviations will reflect usual clinical practice |
| Bias due to missing data  (Selection bias) | Serious risk | 6 out of 26 patients in the hydroxychloroquine group were lost to follow-up and not analyzed. These patients mostly had poor outcomes; had they been included, the results of the study may have been different. No such missing data observed in the control group |
| Bias in measurement of the outcome  (Information bias) | Moderate risk | Open label study. Outcome assessors were not blinded to the treatment allocation of patients; this may have a slight impact on the results. |
| Bias in selection of reported result (Reporting bias) | No information | No information as to whether authors selected outcomes measure a priori. |

Bessiere et al. “Assessment of QT intervals in a case series of patients with coronavirus disease 2019 (COVID-19) infection treated with hydroxychloroquine alone or in combination with azithromycin in an intensive care unit”.

ROBINS-I (risk of bias assessment for nonrandomized studies)

| Bias | Author’s judgment | Support for judgment |
| --- | --- | --- |
| **Pre-intervention domains** | | |
| Bias due to confounding  (Confounding) | Moderate risk | Before-after study; the effect of medication on QT interval are expected to be almost immediate. Although the viral infection itself may be a major confounding factor, in this network meta-analysis this study was exclusively used in QT prolongation analysis; and delta QTc was used rather than absolute post-treatment QTc interval to minimize the effect of potential confounders. |
| Bias in selection of participants into the study  (Selection bias) | Low risk | Retrospective study including all consecutive patients with RT-PCR-confirmed COVID-19 and have received hydroxychloroquine with or without azithromycin. |
| **At-intervention domain** | | |
| Bias in classification of interventions  (Information bias) | Low risk | Intervention was hydroxychloroquine with or without azithromycin. Intervention status well-defined and intervention definition based on information collected at time of intervention. |
| **Post-intervention domains** | | |
| Bias due to deviations from intended interventions  (Confounding) | Moderate risk | Antiviral treatment ceased before completion for 42.5% of patients due to adverse effects; however, this is expected to dampen the result of this letter rather than skew the results in favor of it, so this was not assessed to confer a serious or critical risk of bias. |
| Bias due to missing data  (Selection bias) | Low risk | EKG was taken from all patients. |
| Bias in measurement of the outcome  (Information bias) | Low risk | EKG results of all patients were reviewed by 2 masked cardiac electrophysiologists for objective measures of QTc prolongation. |
| Bias in selection of the reported result (reporting bias) | No information | QTc prolongation was defined by increase in QTc interval of more than 60 milliseconds compared to baseline or as QTc of 500 milliseconds or greater. While objective, unclear if authors selected this outcome measure a priori. |

Mercuro et al. “Risk of QT interval prolongation associated with use of hydroxychloroquine with or without concomitant azithromycin among hospitalized patients testing positive for coronavirus disease 2019 (COVID-19)”.

ROBINS-I (risk of bias assessment for nonrandomized studies)

| Bias | Author’s judgment | Support for judgment |
| --- | --- | --- |
| **Pre-intervention domains** | | |
| Bias due to confounding  (Confounding) | Moderate risk | Before-after study; the effect of medication on QT interval are expected to be almost immediate. Although the viral infection itself may be a major confounding factor, in this network meta-analysis this study was exclusively used in QT prolongation analysis, and delta QTc was used rather than absolute post-treatment QTc interval to minimize the effect of potential confounders. |
| Bias in selection of participants into the study  (Selection bias) | Low risk | Included patients who received at least 1 day of hydroxychloroquine and at least 1 positive COVID-19 nasopharyngeal PCR test result. |
| **At-intervention domain** | | |
| Bias in classification of interventions  (Information bias) | Low risk | Intervention was hydroxychloroquine with or without azithromycin. Intervention status well-defined and intervention definition based on information collected at time of intervention. |
| **Post-intervention domains** | | |
| Bias due to deviations from intended interventions  (Confounding) | Moderate risk | Ten patients (11%) stopped taking hydroxychloroquine prior to day 5 due to side effects; however, this is expected to dampen the result of this report rather than skew the results in favor of it, so this was not assessed to confer a serious or critical risk of bias. |
| Bias due to missing data  (Selection bias) | Moderate risk | 19 out of 90 patients had no follow-up EKGs. |
| Bias in measurement of the outcome  (Information bias) | Moderate risk | EKGs were manually evaluated by non-blinded cardiologists to calculate QTc intervals |
| Bias in selection of reported result (Reporting bias) | No information | QTc prolongation was defined by increase in QTc interval of more than 60 milliseconds compared to baseline or as QTc of 500 milliseconds or greater. While objective, unclear if authors selected this outcome measure a priori. |

Li et al. “Efficacy and safety of lopinavir/ritonavir or arbidol in adult patients with mild/moderate COVID-19: an exploratory randomized controlled trial.”

RoB 2 tool

| Bias | Author’s judgement | Support for judgement |
| --- | --- | --- |
| Bias arising from the randomization process | Low risk | Allocation concealment by centralized web-based randomization system in which the participant identifier was entered before the allocation was revealed |
| Bias due to deviations from intended interventions | Low risk | One patient stopped the assigned treatment, but this was unlikely to affect the outcome. No participants were analyzed in the wrong group. |
| Bias due to missing outcome data | Low risk | Data were available for all participants. |
| Bias in measurement of the outcome | Low risk | Outcome assessors were unaware of the intervention received by study participants. Clinicians taking care of patients were not blinded, but this is deemed unlikely to affect the outcome. |
| Bias in selection of the reported result | Some concerns | No information as to whether the reported data were selected based on the results of multiple outcome measurements or multiple analyses of data. |

Tang et al. “Hydroxychloroquine in patients with mainly mild to moderate coronavirus disease 2019: open label, randomized controlled trial”.

RoB 2 tool

| Bias | Author’s judgement | Support for judgement |
| --- | --- | --- |
| Bias arising from the randomization process | Low risk | Independent statistician not involved in data analysis implemented randomization rules. Allocation cards were randomly generated by a computer in sequentially numbered envelopes that were opened as patients were enrolled. |
| Bias due to deviations from intended interventions | Low risk | Deviations from intended intervention were minor and unlikely to affect the outcome in a major way. |
| Bias due to missing outcome data | Low risk | Data available for all participants. |
| Bias in measurement of the outcome | Low risk | Open label study. Outcome assessors were unaware of the intervention received by study participants. Clinicians were not blinded. |
| Bias in selection of the reported result | Low risk | The original protocol prespecified primary endpoint as negative conversion rate by day 10, but this was modified to negative conversion rate by day 28 after new discoveries about SARS-CoV-2. This was deemed at low risk of selective reporting bias. |

Mahevas et al. “Clinical efficacy of hydroxychloroquine in patients with covid-19 pneumonia who require oxygen: observational comparative study using routine care data”.

ROBINS-I (risk of bias assessment for nonrandomized studies)

| Bias | Author’s judgment | Support for judgment |
| --- | --- | --- |
| **Pre-intervention domains** | | |
| Bias due to confounding  (Confounding) | Low risk | Decision of whether to treat patients with hydroxychloroquine based on local medical consensus and clinicians’ own opinions. Inverse probability weighting approach used to balance differences in baseline variables; four variables could not be accounted for (this is not deemed to confer a significant risk of confounding bias). |
| Bias in selection of participants into the study  (Selection bias) | Low risk | All patients admitted with covid-19 pneumonia were screened for enrolment. Patients who received hydroxychloroquine within 48 hours were designated to be the treatment group (start of intervention i.e. within 48hrs was considered to coincide with start of follow-up). |
| **At-intervention domain** | | |
| Bias in classification of interventions  (Information bias) | Low risk | Intervention is treatment with hydroxychloroquine – well-defined and definition based solely on information collected at the time of intervention |
| **Post-intervention domains** | | |
| Bias due to deviations from intended interventions  (Confounding) | Low risk | Any deviations reflect usual clinical practice. |
| Bias due to missing data  (Selection bias) | Low risk | Data were complete. |
| Bias in measurement of the outcome  (Information bias) | Low risk | Outcomes of mortality, ICU admission, and ARDS – objective, robust measures – were used (unlikely to be affected by knowledge of intervention received). |
| Bias in selection of reported result (Reporting bias) | Low risk | While unclear if authors selected the reported outcome measures a prior, the outcome measures are objective; and all outcome measures agreed with each other. |

Lu et al. “Adjuvant corticosteroid therapy for critically ill patients with COVID-19”.

ROBINS-I (risk of bias assessment for nonrandomized studies)

| Bias | Author’s judgment | Support for judgment |
| --- | --- | --- |
| **Pre-intervention domains** | | |
| Bias due to confounding  (Confounding) | Low risk | Retrospective review of records for corticosteroid use. Multivariate analysis that adjusted for major mortality-associated variables and propensity scores conducted. |
| Bias in selection of participants into the study  (Selection bias) | Moderate risk | Medical records of adult patients with COVID-19 admitted to Tongji Hospital were screened. Patients who were critically ill and were treated with antivirals were enrolled. Start of intervention and follow-up were different for every patient; median corticosteroid treatment duration was 8 days. |
| **At-intervention domain** | | |
| Bias in classification of interventions  (Information bias) | Low risk | Intervention is corticosteroid or equivalent medication. Well-defined and definition based solely on information collected at time of intervention |
| **Post-intervention domains** | | |
| Bias due to deviations from intended interventions  (Confounding) | Low risk | Deviations from intended intervention would reflect usual practice |
| Bias due to missing data  (Selection bias) | Low risk | Data were complete |
| Bias in measurement of the outcome  (Information bias) | Low risk | Main outcome measure was mortality – objective and robust measure. |
| Bias in selection of reported result (Reporting bias) | Low risk | No information provided as to whether the outcome measure was selected a priori; however, because mortality is the most important, objective outcome measure in severe viral pneumonia, risk of reporting bias is deemed low. |

Chen et al. “Associations of clinical characteristics and antiviral drugs with viral RNA clearance in patients with COVID-19 in Guangzhou, China”.

ROBINS-I (risk of bias assessment for nonrandomized studies)

| Bias | Author’s judgment | Support for judgment |
| --- | --- | --- |
| **Pre-intervention domains** | | |
| Bias due to confounding  (Confounding) | Moderate risk | Retrospective study. Confounders were adjusted for with Cox regression, but description of this process is not clear. |
| Bias in selection of participants into the study  (Selection bias) | Moderate risk | Medical records of consecutive patients with COVID-19 admitted to Guangzhou Eight People’s Hospital in a certain period were reviewed. Because this study is a retrospective record review, antiviral treatment initiation and follow-up initiation were likely different for most patients |
| **At-intervention domain** | | |
| Bias in classification of interventions  (Information bias) | Low risk | Interventions consist of medication – well-defined and definition of intervention based solely on information collected at time of intervention. |
| **Post-intervention domains** | | |
| Bias due to deviations from intended interventions  (Confounding) | Low risk | Deviations would most likely reflect usual clinical practice. |
| Bias due to missing data  (Selection bias) | Low risk | Data were nearly complete; 98.6% had valid data on time to viral RNA clearance. |
| Bias in measurement of the outcome  (Information bias) | Low risk | Outcome assessment was same for all participants, and outcome measures are unlikely to be influenced by knowledge of assessment received by participants. |
| Bias in selection of reported result (Reporting bias) | No information | Time to viral clearance was used as main outcome measure. While objective, unclear if authors selected this outcome measure a priori. |

Shao et al. “Clinical efficacy of intravenous immunoglobulin therapy in critical patients with COVID-19: a multicenter retrospective cohort study”.

ROBINS-I (risk of bias assessment for nonrandomized studies)

| Bias | Author’s judgment | Support for judgment |
| --- | --- | --- |
| **Pre-intervention domains** | | |
| Bias due to confounding  (Confounding) | Low risk | Retrospective study. Multivariable Cox regression analysis was used to adjust for confounders. |
| Bias in selection of participants into the study  (Selection bias) | Moderate risk | Electronic medical records were drawn from eight treatment centers and screened for eligible patients. Because this study is a retrospective record review, IVIG treatment initiation and follow-up initiation were likely different for most patients. |
| **At-intervention domain** | | |
| Bias in classification of interventions  (Information bias) | Low risk | Intervention of interest is IVIG. Intervention is well-defined and intervention definition is based solely on information collected at time of intervention. |
| **Post-intervention domains** | | |
| Bias due to deviations from intended interventions  (Confounding) | Low risk | Any deviations from intended intervention would likely reflect usual clinical practice. |
| Bias due to missing data  (Selection bias) | Low risk | 13 out of 338 patients were missing key information; data were nearly complete. |
| Bias in measurement of the outcome  (Information bias) | Low risk | Main outcome measure of mortality is objective and robust - unlikely to be influenced by knowledge of intervention received. |
| Bias in selection of reported result (Reporting bias) | Low risk. | While unclear if authors selected the reported outcome measures a prior, the main outcome measure of mortality is robust and relevant; therefore, risk of reporting bias is deemed low. |

Deng et al. “Arbidol combined with LPV/r versus LPV/r alone against corona virus disease 2019: a retrospective cohort study”.

ROBINS-I (risk of bias assessment for nonrandomized studies)

| Bias | Author’s judgment | Support for judgment |
| --- | --- | --- |
| **Pre-intervention domains** | | |
| Bias due to confounding  (Confounding) | Serious risk | Baseline characteristics were generally comparable, but use of corticosteroid therapy was significantly different between groups. This raises concern for imbalance of severity between the two groups for which no control was attempted. However, in this network meta-analysis, the reviewers only used the specific adverse event analyses that is less likely to be affected by corticosteroid use. |
| Bias in selection of participants into the study  (Selection bias) | Low risk | Individuals diagnosed with laboratory-confirmed COVID-19 in a certain period were enrolled. Start of intervention and follow-up coincide. |
| **At-intervention domain** | | |
| Bias in classification of interventions  (Information bias) | Low risk | Intervention was LPV/r plus arbidol or LPV/r alone. Intervention definition was clear and based solely on information collected at the time of intervention. |
| **Post-intervention domains** | | |
| Bias due to deviations from intended interventions  (Confounding) | Low risk | Deviations are unlikely, and deviations that do occur are likely to reflect usual clinical practice. |
| Bias due to missing data  (Selection bias) | Low risk | Data were complete. |
| Bias in measurement of the outcome  (Information bias) | Low risk | Outcome assessors were unaware of the treatment allocations of patients. |
| Bias in selection of reported result (Reporting bias) | No information. | Viral clearance at day 7 and 14 were used as main outcome measure. Unclear if authors selected this outcome measure a priori. |

Wang et al. “A retrospective cohort study of methylprednisolone therapy in severe patients with COVID-19 pneumonia”.

ROBINS-I (risk of bias assessment for nonrandomized studies)

| Bias | Author’s judgment | Support for judgment |
| --- | --- | --- |
| **Pre-intervention domains** | | |
| Bias due to confounding  (Confounding) | Moderate risk | No significant differences were found between treatment groups among the measured variables including SpO2 at rest on admission. However, potential for unknown confounders remains. |
| Bias in selection of participants into the study  (Selection bias) | Moderate risk | 46 patients with COVID-19 pneumonia at an isolation ward was studied. Start of follow-up and start of intervention did not likely coincide for most patients in the corticosteroid group. |
| **At-intervention domain** | | |
| Bias in classification of interventions  (Information bias) | Low risk | Intervention of interest is corticosteroid. Intervention definition is clear and based solely on information collected at the time of intervention. |
| **Post-intervention domains** | | |
| Bias due to deviations from intended interventions  (Confounding) | Low risk | Any deviation from intended intervention most likely reflect clinical practice. |
| Bias due to missing data  (Selection bias) | Low risk | Data were complete. |
| Bias in measurement of the outcome  (Information bias) | Moderate risk | Certain outcome measures (e.g. length of ICU stay, length of hospitalization) may be minimally influenced by knowledge of intervention received by patients. |
| Bias in selection of reported result (Reporting bias) | No information. | Unclear if authors selected outcome measures a priori. |

Saleh et al. “The effect of chloroquine, hydroxychloroquine, azithromycin on the corrected QT interval in patients with SARS-CoV-2 infection”.

ROBINS-I (risk of bias assessment for nonrandomized studies)

| Bias | Author’s judgment | Support for judgment |
| --- | --- | --- |
| **Pre-intervention domains** | | |
| Bias due to confounding  (Confounding) | Moderate risk | Before-after study; the effect of medication on QT interval are expected to be almost immediate. Although the viral infection itself may be a major confounding factor, in this network meta-analysis this study was exclusively used in QT prolongation analysis, and delta QTc was used rather than absolute post-treatment QTc interval to minimize the effect of potential confounders. |
| Bias in selection of participants into the study  (Selection bias) | Low risk | All hospitalized patients with PCR-confirmed COVID-19 treated with chloroquine/hydroxychloroquine with or without azithromycin were identified. Start of treatment and start of follow-up coincided. |
| **At-intervention domain** | | |
| Bias in classification of interventions  (Information bias) | Low risk | Intervention of interest is medication with hydroxychloroquine/chloroquine with or without azithromycin. Intervention definition is clear and based solely on information collected at time of intervention. |
| **Post-intervention domains** | | |
| Bias due to deviations from intended interventions  (Confounding) | Low risk | Any deviation from intended intervention likely reflects usual clinical practice |
| Bias due to missing data  (Selection bias) | Low risk | Data were complete |
| Bias in measurement of the outcome  (Information bias) | Moderate risk | Outcome measure may be minimally influenced by knowledge of intervention received by study participants. |
| Bias in selection of reported result (Reporting bias) | No information | Unclear if authors selected outcome measures a priori. |

Beigel et al. “Remdesivir for the treatment of Covid-19 – preliminary report”.

RoB 2 tool

| Bias | Author’s judgement | Support for judgement |
| --- | --- | --- |
| Bias arising from the randomization process | Low risk | Randomization was performed using a web-based Data Entry System, Advantage eClinical^SM^. No substantial differences in the baseline characteristics of both groups. |
| Bias due to deviations from intended interventions | Low risk | Deviations due to trial context unlikely. |
| Bias due to missing outcome data | Low risk | Missing data were unlikely to have had a significant impact on the results |
| Bias in measurement of the outcome | Low risk | Double-blind, placebo-controlled trial. |
| Bias in selection of the reported result | Low risk | Outcome measure was clinical recovery defined by discharge from hospital or equivalent, as per the pre-established protocol (changed before results were available). |

Goldman et al. “Remdesivir for 5 or 10 days in patients with severe Covid-19”.

RoB 2 tool

| Bias | Author’s judgement | Support for judgement |
| --- | --- | --- |
| Bias arising from the randomization process | Some concerns | Randomization and allocation were conducted by the sponsor (Gilead Sciences), but baseline imbalances were present. |
| Bias due to deviations from intended interventions | Low risk | Open label trial but no deviations due to trial context. |
| Bias due to missing outcome data | Low risk | Missing outcome data is sufficiently small that they were unlikely to impact the outcome |
| Bias in measurement of the outcome | Some concerns | Open label trial; assessment or clinical care could have been influenced by knowledge of intervention, but it is unlikely that this would have happened. |
| Bias in selection of the reported result | Low risk | Outcome measure was clinical recovery defined on a seven-point scale, as per the pre-established protocol. |

Zheng et al. “A novel protein drug, Novaferon, as the potential antiviral drug for COVID-19”.

RoB 2 tool

| Bias | Author’s judgement | Support for judgement |
| --- | --- | --- |
| Bias arising from the randomization process | Low risk | SAS package was used to generate a simple 1:1:1 randomization schedule by a statistician not involved in the trial. There were no differences in the baseline characteristics of patients in the three groups. |
| Bias due to deviations from intended interventions | Low risk | There were no deviations from the intended intervention. |
| Bias due to missing outcome data | Low risk | Worst case scenario used to impute missing data as a sensitivity analysis; no change in results were seen |
| Bias in measurement of the outcome | Low risk | Outcome assessment was unlikely to be influenced by the knowledge of intervention received. |
| Bias in selection of the reported result | Some concerns | Not enough information to determine selective reporting bias. |

Zhong et al. “A randomized, single-blind, group sequential, active-controlled study to evaluate the clinical efficacy and safety of a-lipoic acid for critically ill patients with coronavirus disease 2019 (COVID-19)”.

RoB 2 tool

| Bias | Author’s judgement | Support for judgement |
| --- | --- | --- |
| Bias arising from the randomization process | Low risk | Allocation sequences were placed in sealed opaque envelopes. There were no imbalances in the two treatment groups. |
| Bias due to deviations from intended interventions | Low risk | No reported deviations from intended intervention. |
| Bias due to missing outcome data | Low risk | Data were complete. |
| Bias in measurement of the outcome | Low risk | Assessment of outcome unlikely to be influenced by the assessors’ knowledge of intervention received (qSOFA score and mortality) |
| Bias in selection of the reported result | Some concerns | Not enough information to determine selective reporting bias. |

Lou et al. “Clinical outcomes and plasma concentrations of baloxavir marboxil and favipravir in COVID-19 patients: an exploratory randomized, controlled trial”.

RoB 2 tool

| Bias | Author’s judgement | Support for judgement |
| --- | --- | --- |
| Bias arising from the randomization process | Some concerns | Description of the randomization process unclear (perhaps because it is a preprint), but no baseline imbalances. |
| Bias due to deviations from intended interventions | Low risk | No reported deviations from intended intervention. |
| Bias due to missing outcome data | Low risk | Data were nearly complete. |
| Bias in measurement of the outcome | Some concerns | Assessment of clinical improvement may be minimally influenced by knowledge of intervention received. |
| Bias in selection of the reported result | Some concerns | No information as to whether the reported data were selected based on the results of multiple outcome measurements or multiple analyses of data. |

Ramireddy et al. “Experience with hydroxychloroquine and azithromycin in the COVID-19 pandemic: implications for QT interval monitoring”.

ROBINS-I (risk of bias assessment for nonrandomized studies)

| Bias | Author’s judgment | Support for judgment |
| --- | --- | --- |
| **Pre-intervention domains** | | |
| Bias due to confounding  (Confounding) | Moderate risk | All included patients had confirmed COVID-19 or were under investigation for COVID-19. Baseline ECG features (i.e., QTc interval) between medication groups were not comparable; thereafter, delta QTc was used rather than absolute post-treatment QTc interval to compare pure differences in QTc change after treatment. Since no other descriptions for difference between baseline characteristics other than ECG profile were provided, this study was deemed at moderate risk for confounding bias as it was used exclusively for QT prolongation analysis. While the study as a whole (i.e. efficacy analysis) may be at serious risk of confounding bias according to Cochrane definitions, the reviewers assigned moderate risk as we only focused on the QT prolongation-related aspects of this study. |
| Bias in selection of participants into the study  (Selection bias) | Serious risk | All patients with confirmed COVID-19 as well as patients under investigation admitted at a center who received azithromycin and/or hydroxychloroquine were included. However, 122 patients were excluded because no EKG was available; this insignificant population may have introduced a selection bias. |
| **At-intervention domain** | | |
| Bias in classification of interventions  (Information bias) | Low risk | Intervention consisted of treatment with hydroxychloroquine and/or azithromycin. Intervention classifications should be correct. |
| **Post-intervention domains** | | |
| Bias due to deviations from intended interventions  (Confounding) | Low risk | Any deviations from intended intervention should reflect usual clinical practice |
| Bias due to missing data  (Selection bias) | Moderate risk | 94 patients with inadequate EKG work-up were excluded; this may have introduced a certain degree of selection bias. |
| Bias in measurement of the outcome  (Information bias) | Low risk | Outcome assessment was consistent before and after intervention. |
| Bias in selection of reported result (Reporting bias) | No information | Unclear if authors selected the reported outcome measures a priori |

Freedberg et al. “Famotidine use is associated with improved clinical outcomes in hospitalized COVID-19 patients: a propensity score matched retrospective cohort study”.

ROBINS-I (risk of bias assessment for nonrandomized studies)

| Bias | Author’s judgment | Support for judgment |
| --- | --- | --- |
| **Pre-intervention domains** | | |
| Bias due to confounding  (Confounding) | Low risk | Retrospective cohort study. Multivariable Cox proportional hazards model was constructed adjusting for baseline characteristics. |
| Bias in selection of participants into the study  (Selection bias) | Moderate risk | Famotidine therapy began before the start of follow-up in this investigation for many patients who received famotidine. |
| **At-intervention domain** | | |
| Bias in classification of interventions  (Information bias) | Low risk | Intervention classifications are deemed reliable. |
| **Post-intervention domains** | | |
| Bias due to deviations from intended interventions  (Confounding) | Low risk | Any deviation from intended intervention likely reflect usual clinical practice |
| Bias due to missing data  (Selection bias) | Low risk | Data were complete |
| Bias in measurement of the outcome  (Information bias) | Low risk | Main outcome measure was a composite of death and intubation which are robust and unlikely to be influenced by knowledge of intervention received. |
| Bias in selection of reported result (Reporting bias) | No information | Unclear if authors selected the reported outcome measures a priori |

Magagnoli et al. “Outcomes of hydroxychloroquine usage in United States veterans hospitalized with Covid-19”.

ROBINS-I (risk of bias assessment for nonrandomized studies)

| Bias | Author’s judgment | Support for judgment |
| --- | --- | --- |
| **Pre-intervention domains** | | |
| Bias due to confounding  (Confounding) | Low risk | Retrospective cohort study. Competing risk hazard regression adjusting for clinical characteristics via propensity scores performed. |
| Bias in selection of participants into the study  (Selection bias) | Moderate risk | Start of intervention and start of follow-up do not necessarily coincide for many patients |
| **At-intervention domain** | | |
| Bias in classification of interventions  (Information bias) | Low risk | Interventions of interest are hydroxychloroquine and/or azithromycin pharmacotherapy. Intervention status is well-defined, and intervention definition is based solely on information collected at time of intervention. |
| **Post-intervention domains** | | |
| Bias due to deviations from intended interventions  (Confounding) | Low risk | Deviations likely reflect usual clinical practice. |
| Bias due to missing data  (Selection bias) | Low risk | Data were complete. |
| Bias in measurement of the outcome  (Information bias) | Low risk | Main outcome measures were death vs discharge and ventilation, which is deemed unlikely to be affected by knowledge of intervention received. |
| Bias in selection of reported result (Reporting bias) | No information. | Unclear if authors selected the reported outcome measures a priori |

Chen et al. “Efficacy of hydroxychloroquine in patients with COVID-19: results of a randomized clinical trial”.

RoB 2 tool

| Bias | Author’s judgement | Support for judgement |
| --- | --- | --- |
| Bias arising from the randomization process | High risk | Description of randomization process is unclear, and baseline characteristics are not well described. |
| Bias due to deviations from intended interventions | Low risk | No deviations from intended intervention due to trial context are expected. |
| Bias due to missing outcome data | Low risk | Data were complete |
| Bias in measurement of the outcome | Low risk | Outcome assessors were blinded to treatment assignment |
| Bias in selection of the reported result | Some concerns | Not enough information to determine whether the reported data were selected based on the results of multiple outcome measurements or multiple analyses of data. |

Carlucci et al. “Hydroxychloroquine and azithromycin plus zinc vs hydroxychloroquine and azithromycin alone: outcomes in hospitalized COVID-19 patients”.

ROBINS-I (risk of bias assessment for nonrandomized studies)

| Bias | Author’s judgment | Support for judgment |
| --- | --- | --- |
| **Pre-intervention domains** | | |
| Bias due to confounding  (Confounding) | Moderate risk | Retrospective cohort study. Although several baseline characteristics were deemed significantly different between groups, we judged that the statistical significance of these differences were potentially overestimated due to large number of sample size in each arm (>400). This is supported by the fact that absolute values (including variances) of baseline features were very similar across groups and presumably pose only small impact on the outcome clinically (for example, level of troponin was 0.01 (0.01-0.02) ng/mL for one group and 0.015 (0.01-0.02) for the other group, and p-value was 0.011. It is unlikely such a minor difference would affect clinical outcomes). Also, adjustment for difference in timing between patients who received zinc and those who did not were performed along with sensitivity analyses for different severities to tackle certain confounding variables. |
| Bias in selection of participants into the study  (Selection bias) | Moderate risk | Start of intervention and start of follow-up did not necessarily coincide for all patients. |
| **At-intervention domain** | | |
| Bias in classification of interventions  (Information bias) | Low risk | Intervention of interest is pharmacotherapy with hydroxychloroquine/azithromycin and/or zinc. Intervention status is well-defined, and intervention definition is based solely on information collected at time of intervention. |
| **Post-intervention domains** | | |
| Bias due to deviations from intended interventions  (Confounding) | Low risk | Any deviations from intended intervention likely reflect usual clinical practice. |
| Bias due to missing data  (Selection bias) | Low risk | Data were complete. |
| Bias in measurement of the outcome  (Information bias) | Low risk | Main outcome measures were death, discharge, or transition to hospice – which are unlikely to be affected by knowledge of treatment allocation. |
| Bias in selection of reported result (Reporting bias) | No information. | Unclear if authors selected the reported outcome measures a priori |

Lan et al. “Lopinavir-ritonavir alone or combined with arbidol in the treatment of 73 hospitalized patients with COVID-19: a pilot retrospective study”.

ROBINS-I (risk of bias assessment for nonrandomized studies)

| Bias | Author’s judgment | Support for judgment |
| --- | --- | --- |
| **Pre-intervention domains** | | |
| Bias due to confounding  (Confounding) | Moderate risk | While baseline characteristics were generally comparable, sex difference was significant between groups. However, other factors (e.g. severity, symptoms, comorbidities) were similar. |
| Bias in selection of participants into the study  (Selection bias) | Moderate risk | Start of intervention and start of follow-up did not necessarily coincide for all patients. |
| **At-intervention domain** | | |
| Bias in classification of interventions  (Information bias) | Low risk | Intervention of interest is pharmacotherapy with lopinavir/ritonavir and/or arbidol. Intervention status is well-defined, and intervention definition is based solely on information collected at time of intervention. |
| **Post-intervention domains** | | |
| Bias due to deviations from intended interventions  (Confounding) | Low risk | Any deviations from intended intervention likely reflect usual clinical practice. |
| Bias due to missing data  (Selection bias) | Low risk | Data were complete |
| Bias in measurement of the outcome  (Information bias) | Serious risk | Certain outcome measures (e.g. improvement in pulmonary symptoms) were subjective and vulnerable to influence by knowledge of intervention received by study participants. |
| Bias in selection of reported result (Reporting bias) | No information | Unclear if authors selected the reported outcome measures a priori |

Huang et al. “Preliminary evidence from a multicenter prospective observational study of the safety and efficacy of chloroquine for the treatment of COVID-19”.

ROBINS-I (risk of bias assessment for nonrandomized studies)

| Bias | Author’s judgment | Support for judgment |
| --- | --- | --- |
| **Pre-intervention domains** | | |
| Bias due to confounding  (Confounding) | Moderate risk | Imbalances in the baseline characteristics are accounted for using post hoc analyses within subgroups divided by important factors such as dosage options, clinical manifestations, and interval time between symptom onset and treatment initiation; however, potential for confounding remains. |
| Bias in selection of participants into the study  (Selection bias) | Low risk | Patients enrolled according to eligibility criteria (the reviewers assumed from context that all eligible patients were enrolled although this was not explicitly stated). Start of follow-up coincided with start of intervention. |
| **At-intervention domain** | | |
| Bias in classification of interventions  (Information bias) | Low risk | Intervention of interest is chloroquine therapy. Intervention status is well-defined, and intervention definition is based solely on information collected at time of intervention. |
| **Post-intervention domains** | | |
| Bias due to deviations from intended interventions  (Confounding) | Low risk | Any deviations from intended intervention likely reflect usual clinical practice. |
| Bias due to missing data  (Selection bias) | Low risk | Data were complete |
| Bias in measurement of the outcome  (Information bias) | Moderate risk | Main outcome measure was time from treatment initiation to undetectable viral RNA. While an objective measure, knowledge of intervention received may have minimally affected certain aspects of patient care and testing. |
| Bias in selection of reported result (Reporting bias) | No information. | Unclear if authors selected the reported outcome measures a priori |

Wu et al. “Systemic corticosteroids show no benefit in severe and critical COVID-19 patients in Wuhan, China: a retrospective cohort study”.

ROBINS-I (risk of bias assessment for nonrandomized studies)

| Bias | Author’s judgment | Support for judgment |
| --- | --- | --- |
| **Pre-intervention domains** | | |
| Bias due to confounding  (Confounding) | Low risk | Retrospective cohort study. Inverse probability weighting technique and propensity score matching are used to adjust for confounders. |
| Bias in selection of participants into the study  (Selection bias) | Low risk | Consecutive COVID-19 patients with severe/critical illness are enrolled. Start of treatment and start of follow-up did not necessarily coincide, but median initial time of intervention was 2.2 hours and 0.1 hours after starting follow-up in severe and critical patients respectively, which reduces concern for selection bias. |
| **At-intervention domain** | | |
| Bias in classification of interventions  (Information bias) | Low risk | Intervention of interest is corticosteroid therapy. Intervention status is well-defined, and intervention definition is based solely on information collected at time of intervention. |
| **Post-intervention domains** | | |
| Bias due to deviations from intended interventions  (Confounding) | Low risk | Any deviations from intended intervention likely reflect usual clinical practice. |
| Bias due to missing data  (Selection bias) | Low risk | Data were complete for main outcome measure of mortality; multiple imputation was used for missing data in multivariable analysis. |
| Bias in measurement of the outcome  (Information bias) | Low risk | Main outcome measure was mortality, which is highly unlikely to be affected by the assessors’ knowledge of intervention received by patients |
| Bias in selection of reported result (Reporting bias) | Low risk | Although it is unclear if authors selected the reported outcome measures a priori, the objectively most important outcome for severe/critical COVID-19 is mortality, and this is used as the main outcome measure. |

Kim et al. “Treatment response to hydroxychloroquine, lopinavir-ritonavir, and antibiotics for moderate COVID-19: a first report on the pharmacological outcomes from South Korea”.

ROBINS-I (risk of bias assessment for nonrandomized studies)

| Bias | Author’s judgment | Support for judgment |
| --- | --- | --- |
| **Pre-intervention domains** | | |
| Bias due to confounding  (Confounding) | Moderate risk | Retrospective cohort study. Baseline characteristics were shown to be similar between HQ/AZ and Lop/R groups, but conservative treatment group was different in that it was composed of milder cases. As this study was conducted by our research group, we conducted multivariate-adjusted cox proportional regression, and confirmed that HQ plus AZ was better than Lop/R plus AZ and conservative after adjusted for baseline characteristics. |
| Bias in selection of participants into the study  (Selection bias) | Low risk | Patients enrolled according to eligibility criteria (the reviewers assumed from context that all eligible patients were enrolled although this was not explicitly stated). Start of follow-up coincided with start of intervention |
| **At-intervention domain** | | |
| Bias in classification of interventions  (Information bias) | Low risk | Interventions of interest are treatment with hydroxychloroquine or lopinavir/ritonavir. Intervention status is well-defined, and intervention definition is based solely on information collected at time of intervention. |
| **Post-intervention domains** | | |
| Bias due to deviations from intended interventions  (Confounding) | Low risk | Deviations from intended intervention likely reflect usual clinical practice. |
| Bias due to missing data  (Selection bias) | Low risk | Data were complete |
| Bias in measurement of the outcome  (Information bias) | Moderate risk | Main outcome measure was time to viral clearance on RT-PCR. While an objective measure, knowledge of intervention received may have minimally affected certain aspects of patient care and testing. |
| Bias in selection of reported result (Reporting bias) | No information | Unclear if authors selected the reported outcome measures a priori |

Shi et al. “The potential of low molecular weight heparin to mitigate cytokine storm in severe COVID-19 patients: a retrospective clinical study.”

ROBINS-I (risk of bias assessment for nonrandomized studies)

| Bias | Author’s judgment | Support for judgment |
| --- | --- | --- |
| **Pre-intervention domains** | | |
| Bias due to confounding  (Confounding) | Moderate risk | Retrospective observational study. Baseline characteristics were considered similar between groups. |
| Bias in selection of participants into the study  (Selection bias) | Moderate risk | Patients enrolled according to eligibility criteria (the reviewers assumed from context that all eligible patients were enrolled although this was not explicitly stated). Start of follow-up did not necessarily coincide with start of intervention. |
| **At-intervention domain** | | |
| Bias in classification of interventions  (Information bias) | Low risk | Intervention of interest is low molecular weight heparin. Intervention status is well-defined, and intervention definition is based solely on information collected at time of intervention. |
| **Post-intervention domains** | | |
| Bias due to deviations from intended interventions  (Confounding) | Low risk | Deviations from intended intervention likely reflect usual clinical practice. |
| Bias due to missing data  (Selection bias) | Low risk | Data were complete |
| Bias in measurement of the outcome  (Information bias) | Moderate risk | Main outcome measures were time to negative conversion, length of hospital stay, and laboratory parameters. While mostly objective measures, knowledge of intervention received may have minimally affected certain aspects of patient care and testing. |
| Bias in selection of reported result (Reporting bias) | No information | Unclear if authors selected the reported outcome measures a priori |

Feng et al. “The use of adjuvant therapy in preventing progression to severe pneumonia in patients with coronavirus disease 2019: a multicenter data analysis”.

(This ROB assessment focuses on the chloroquine assessment part of this study).

ROBINS-I (risk of bias assessment for nonrandomized studies)

| Bias | Author’s judgment | Support for judgment |
| --- | --- | --- |
| **Pre-intervention domains** | | |
| Bias due to confounding  (Confounding) | Low risk | Retrospective data analysis. Propensity score matching method was used to control for confounders. |
| Bias in selection of participants into the study  (Selection bias) | Moderate risk | All consecutive COVID-19 patients at the study centers in a certain period were enrolled. Start of follow-up and start of intervention did not necessarily coincide for all patietns. |
| **At-intervention domain** | | |
| Bias in classification of interventions  (Information bias) | Low risk | Intervention of interest is antiviral pharmacotherapy (including chloroquine). Intervention status is well-defined, and intervention definition is based solely on information collected at time of intervention. |
| **Post-intervention domains** | | |
| Bias due to deviations from intended interventions  (Confounding) | Low risk | Deviations from intended intervention likely reflect usual clinical practice. |
| Bias due to missing data  (Selection bias) | Low risk | Data were complete |
| Bias in measurement of the outcome  (Information bias) | Moderate risk | Main outcome of interest was development of severe pneumonia. Knowledge of intervention received may have minimally affected certain aspects of patient care and testing. |
| Bias in selection of reported result (Reporting bias) | No information | Unclear if authors selected the reported outcome measures a priori |

Bian et al. “Meplazumab treats COVID-19 pneumonia: an open-labelled, concurrent controlled add-on clinical trial”.

ROBINS-I (risk of bias assessment for nonrandomized studies)

| Bias | Author’s judgment | Support for judgment |
| --- | --- | --- |
| **Pre-intervention domains** | | |
| Bias due to confounding  (Confounding) | Moderate risk | Non-randomized trial. Baseline characteristics were considered similar between groups. |
| Bias in selection of participants into the study  (Selection bias) | Moderate risk | Prospective, single center, open-labelled trial. Eligible patients were approached for meplazumab treatment; those who did not consent to meplazumab treatment were used as controls. |
| **At-intervention domain** | | |
| Bias in classification of interventions  (Information bias) | Low risk | Intervention of interest is meplazumab add-on therapy. Intervention status is well-defined, and intervention definition is based solely on information collected at time of intervention. |
| **Post-intervention domains** | | |
| Bias due to deviations from intended interventions  (Confounding) | Low risk | Deviations from intended intervention likely reflect usual clinical practice. |
| Bias due to missing data  (Selection bias) | Low risk | Data were complete |
| Bias in measurement of the outcome  (Information bias) | Moderate risk | Main outcome measure was time to negative conversion on RT-PCT. While mostly objective measures, knowledge of intervention received may have minimally affected certain aspects of patient care and testing. |
| Bias in selection of reported result (Reporting bias) | No information | Unclear if authors selected the reported outcome measures a priori |

Singh et al. “Outcomes of hydroxychloroquine treatment among hospitalized COVID-19 patients in the United States – real-world evidence from a federated electronic medical record network”.

ROBINS-I (risk of bias assessment for nonrandomized studies)

| Bias | Author’s judgment | Support for judgment |
| --- | --- | --- |
| **Pre-intervention domains** | | |
| Bias due to confounding  (Confounding) | Low risk | Federated electronic medical record review. Propensity score matching method was used to control for confounders. |
| Bias in selection of participants into the study  (Selection bias) | Moderate risk | All eligible patients were analyzed, but start of follow-up did not necessarily coincide with start of intervention for all patients. |
| **At-intervention domain** | | |
| Bias in classification of interventions  (Information bias) | Low risk | Intervention of interest is hydroxychloroquine therapy. Intervention status is well-defined, and intervention definition is based solely on information collected at time of intervention. |
| **Post-intervention domains** | | |
| Bias due to deviations from intended interventions  (Confounding) | Low risk | Deviations from intended intervention likely reflect usual clinical practice. |
| Bias due to missing data  (Selection bias) | Low risk | Data were complete |
| Bias in measurement of the outcome  (Information bias) | Low risk | Main outcome measure was mortality and mechanical ventilation, which is unlikely to be affected by the assessors’ knowledge of intervention received by patients |
| Bias in selection of reported result (Reporting bias) | Low risk | Although it is unclear if authors selected the reported outcome measures a priori, the objectively most important outcome for severe/critical COVID-19 is mortality, and this is used as the main outcome measure. |

Fernandez et al. “Impact of glucocorticoid treatment in SARS-CoV-2 infection mortality: a retrospective controlled study”.

ROBINS-I (risk of bias assessment for nonrandomized studies)

| Bias | Author’s judgment | Support for judgment |
| --- | --- | --- |
| **Pre-intervention domains** | | |
| Bias due to confounding  (Confounding) | Low risk | Retrospective cohort study. Propensity score matching method was used to control for confounders. |
| Bias in selection of participants into the study  (Selection bias) | Low risk | Patients enrolled according to eligibility criteria (the reviewers assumed from context that all eligible patients were enrolled although this was not explicitly stated). Start of follow-up coincided with the start of intervention. |
| **At-intervention domain** | | |
| Bias in classification of interventions  (Information bias) | Low risk | Intervention of interest is corticosteroid therapy. Intervention status is well-defined, and intervention definition is based solely on information collected at time of intervention. |
| **Post-intervention domains** | | |
| Bias due to deviations from intended interventions  (Confounding) | Low risk | Steroid treatment group was disaggregated into 1mg/kg/day methylprednisolone equivalent subgroup and steroid pulse subgroup. Although there is a chance of deviation to and from one subgroup to another, because the focus of our (the reviewers’) analysis was on the comparison between steroid use versus control, deviation within the steroid group is deemed unimportant. As such, since the risk for deviation from steroid treatment group or control group is low, risk of information bias is low. |
| Bias due to missing data  (Selection bias) | Low risk | Data were complete |
| Bias in measurement of the outcome  (Information bias) | Low risk | Main outcome measure was mortality, which is unlikely to be affected by the assessors’ knowledge of intervention received by patients |
| Bias in selection of reported result (Reporting bias) | Low risk | Although it is unclear if authors selected the reported outcome measures a priori, the objectively most important outcome for severe/critical COVID-19 is mortality, and this is used as the main outcome measure. |

Boulware et al. “A randomized trial of hydroxychloroquine as postexposure prophylaxis for Covid-19”.

(Only the adverse event-related data from this study was used in this network meta-analysis, as this study investigates the effect of hydroxychloroquine as a prophylactic measure which is not the focus of our meta-analysis. Thus, the risk of bias assessment is also performed from the perspective of adverse event-related data.)

RoB 2 tool

| Bias | Author’s judgement | Support for judgement |
| --- | --- | --- |
| Bias arising from the randomization process | Low risk | Allocation sequence was concealed and random. No baseline imbalances were found. |
| Bias due to deviations from intended interventions | Low risk | Double-blind, placebo-controlled trial. |
| Bias due to missing outcome data | Some concerns | 10.7% (46 in the hydroxychloroquine group and 42 in the placebo group) did not complete the full survey. While sensitivity analysis for the primary outcome was performed and shown to be robust to inclusion and exclusion of these patients, with regards to the adverse events for which this study was used, there is possibility that missingness could depend on the adverse event rate of each group. |
| Bias in measurement of the outcome | Low risk | Measurement of outcome were comparable between groups, and outcome assessors were unaware of the intervention received. |
| Bias in selection of the reported result | Low risk | The study is conducted in accordance with a pre-specified protocol. |

Li et al. “Effect of convalescent plasma therapy on time to clinical improvement in patients with severe and life-threatening COVID-19”.

RoB 2 tool

| Bias | Author’s judgement | Support for judgement |
| --- | --- | --- |
| Bias arising from the randomization process | Low risk | Computer-generated numbering (1:1) used to assign standard treatment or convalescent plasma infusion. No baseline imbalances to suggest a problem. |
| Bias due to deviations from intended interventions | Some concerns | Standard therapy in both groups were not protocolized and could be different in the context of plasma transfusion; however, such deviations are concomitant to plasma transfusions and are not therefore deemed to be serious deviations from the intended intervention. |
| Bias due to missing outcome data | Low risk | Primary outcome data were complete. |
| Bias in measurement of the outcome | Some concerns | Open-label study; and the primary outcome was based to some degree on the caregivers’ clinical management decisions which could have been impacted by the knowledge of the intervention received. The outcome assessors were, however, blinded to treatment allocation. |
| Bias in selection of the reported result | Low risk | The study is conducted in accordance with a pre-specified protocol. |

Campochiaro et al. “Efficacy and safety of tocilizumab in severe COVID-19 patients: a single-centre retrospective cohort study”.

ROBINS-I

| Bias | Author’s judgment | Support for judgment |
| --- | --- | --- |
| **Pre-intervention domains** | | |
| Bias due to confounding  (Confounding) | Low risk | Retrospective cohort study. Multivariate analysis conducted. |
| Bias in selection of participants into the study  (Selection bias) | Low risk | Hospitalized patients at a single institution included. Start of follow-up and start of intervention were considered to coincide. |
| **At-intervention domain** | | |
| Bias in classification of interventions  (Information bias) | Low risk | Intervention of interest is tocilizumab therapy. Intervention status is well-defined, and intervention definition is based solely on information collected at time of intervention. |
| **Post-intervention domains** | | |
| Bias due to deviations from intended interventions  (Confounding) | Low risk | Deviations from intended intervention likely reflect usual clinical practice. |
| Bias due to missing data  (Selection bias) | Low risk | Data were complete |
| Bias in measurement of the outcome  (Information bias) | Moderate risk | Main outcome measure was clinical improvement, which can be minimally affected by the assessors’ knowledge of intervention received by patients |
| Bias in selection of reported result (Reporting bias) | No information | Unclear if authors selected the reported outcome measures a priori |

Moreno-Garcia et al. “Tocilizumab is associated with reduced risk of ICU admission and mortality in patients with SARS-CoV-2 infection”.

ROBINS-I (risk of bias assessment for nonrandomized studies)

| Bias | Author’s judgment | Support for judgment |
| --- | --- | --- |
| **Pre-intervention domains** | | |
| Bias due to confounding  (Confounding) | Low risk | Retrospective cohort study. Propensity score to receive tocilizumab as the predicted probability from a logistic regression model using tocilizumab as the dependent variable estimated and included in the multivariable analysis of the main outcome. |
| Bias in selection of participants into the study  (Selection bias) | Low risk | 171 patients who did not require transfer to ICU in the first 24 hours of admission were included. Start of follow-up and start of intervention were considered to coincide. |
| **At-intervention domain** | | |
| Bias in classification of interventions  (Information bias) | Low risk | Intervention of interest is tocilizumab therapy. Intervention status is well-defined, and intervention definition is based solely on information collected at time of intervention. |
| **Post-intervention domains** | | |
| Bias due to deviations from intended interventions  (Confounding) | Low risk | Deviations from intended intervention likely reflect usual clinical practice. |
| Bias due to missing data  (Selection bias) | Low risk | Data were complete |
| Bias in measurement of the outcome  (Information bias) | Moderate risk | Main outcome measure was ICU transfer, which is only minimally affected by the assessors’ knowledge of intervention received by patients |
| Bias in selection of reported result (Reporting bias) | No information | Unclear if authors selected the reported outcome measures a priori |

Somers et al. “Tocilizumab for treatment of mechanically ventilated patients with COVID-19”.

ROBINS-I

| Bias | Author’s judgment | Support for judgment |
| --- | --- | --- |
| **Pre-intervention domains** | | |
| Bias due to confounding  (Confounding) | Low risk | Retrospective cohort study. Multivariable Cox regression with propensity score inverse probability weighting used. |
| Bias in selection of participants into the study  (Selection bias) | Moderate risk | All patients in the enrolment period were screened for eligibility; start of follow-up and start of intervention did not necessarily coincide (26% of patients received tocilizumab 48 hours after intubation). |
| **At-intervention domain** | | |
| Bias in classification of interventions  (Information bias) | Low risk | Intervention of interest is tocilizumab therapy. Intervention status is well-defined, and intervention definition is based solely on information collected at time of intervention. |
| **Post-intervention domains** | | |
| Bias due to deviations from intended interventions  (Confounding) | Low risk | Deviations from intended intervention likely reflect usual clinical practice. |
| Bias due to missing data  (Selection bias) | Low risk | Data for main outcome were complete; missing laboratory values were imputed using multiple imputation. |
| Bias in measurement of the outcome  (Information bias) | Low risk | Main outcome measure was mortality, which is unlikely to be affected by the assessors’ knowledge of intervention received by patients |
| Bias in selection of reported result (Reporting bias) | Low risk | Although it is unclear if authors selected the reported outcome measures a priori, the objectively most important outcome for severe/critical COVID-19 is mortality, and this is used as the main outcome measure. |

Davoudi-Monfrared et al. “Efficacy and safety of interferon β-1a in treatment of severe COVID-19: a randomized clinical trial.”

RoB 2 tool

| Bias | Author’s judgement | Support for judgement |
| --- | --- | --- |
| Bias arising from the randomization process | Some concerns | No information as to whether allocation sequence was concealed. There are no baseline imbalances. |
| Bias due to deviations from intended interventions | Low risk | No deviations from intended intervention due to trial context are expected. |
| Bias due to missing outcome data | Low risk | Data were nearly complete. |
| Bias in measurement of the outcome | Some concerns | Open-label study. Primary outcome was improvement in clinical score, which has potential to be influenced by the assessor’s knowledge of intervention. |
| Bias in selection of the reported result | Some concerns | Not enough information to determine whether the reported data were selected based on the results of multiple outcome measurements or multiple analyses of data. |

Rossi et al “Effect of tocilizumab in hospitalized patients with severe pneumonia COVID-19: a cohort study”.

ROBINS-I

| Bias | Author’s judgment | Support for judgment |
| --- | --- | --- |
| **Pre-intervention domains** | | |
| Bias due to confounding  (Confounding) | Low risk | Retrospective cohort study. Propensity score matching, multivariable Cox regression, and inverse probability score weighting were all applied; and results were presented only if the three analyses revealed concordant results. |
| Bias in selection of participants into the study  (Selection bias) | Moderate risk | All patients in the enrolment period were screened for eligibility; start of follow-up and start of intervention did not necessarily coincide for all patients. |
| **At-intervention domain** | | |
| Bias in classification of interventions  (Information bias) | Low risk | Intervention of interest is tocilizumab therapy. Intervention status is well-defined, and intervention definition is based solely on information collected at time of intervention. |
| **Post-intervention domains** | | |
| Bias due to deviations from intended interventions  (Confounding) | Low risk | Deviations from intended intervention likely reflect usual clinical practice. |
| Bias due to missing data  (Selection bias) | Low risk | Data for main outcome were complete. |
| Bias in measurement of the outcome  (Information bias) | Low risk | Main outcome measure consisted of a composite rate of intubation and death. Risk of measurement bias is inherently low, and assessment of outcome is unlikely to have been affected by knowledge of intervention received. |
| Bias in selection of reported result (Reporting bias) | No information | Main outcome measure is a composite rate of intubation and death; there is no explicit explanation as to why this measure was used as the primary endpoint. Unclear if the outcome measure was selected a priori. |

Cao Y. et al. “Ruxolitinib in treatment of severe coronavirus disease 2019 (COVID-19): a multicenter, single-blind, randomized controlled trial”.

RoB 2 tool

| Bias | Author’s judgement | Support for judgement |
| --- | --- | --- |
| Bias arising from the randomization process | Low risk | Allocation sequence was concealed from assessors; there were no baseline imbalances. |
| Bias due to deviations from intended interventions | Low risk | No deviations from intended intervention due to trial context are expected. |
| Bias due to missing outcome data | Low risk | Data were complete. |
| Bias in measurement of the outcome | Low risk | Assessors were unaware of intervention received by study participants (double-blind). |
| Bias in selection of the reported result | Some concerns | Not enough information to determine whether the reported data were selected based on the results of multiple outcome measurements or multiple analyses of data. |

Huet et al. “Anakinra for severe forms of COVID-19: a cohort study”.

ROBINS-I

| Bias | Author’s judgment | Support for judgment |
| --- | --- | --- |
| **Pre-intervention domains** | | |
| Bias due to confounding  (Confounding) | Low risk | Prospective cohort study with historical control. Adjustments for potential confounding made using multivariable Cox proportional hazards model. |
| Bias in selection of participants into the study  (Selection bias) | Low risk | Consecutive patients enrolled prospectively. Start of follow-up and start of intervention coincided for each patient (day 0 marked as the start of anakinra therapy for treatment group and as the day inclusion criteria was deemed met for control group) |
| **At-intervention domain** | | |
| Bias in classification of interventions  (Information bias) | Low risk | Intervention of interest is anakinra therapy. Intervention status is well-defined, and intervention definition is based solely on information collected at time of intervention. |
| **Post-intervention domains** | | |
| Bias due to deviations from intended interventions  (Confounding) | Low risk | Deviations from intended intervention likely reflect usual clinical practice. |
| Bias due to missing data  (Selection bias) | Low risk | Data for main outcome were complete. |
| Bias in measurement of the outcome  (Information bias) | Low risk | Main outcome measure consisted of a composite rate of ICU transfer for mechanical ventilation and death. Risk of measurement bias is inherently low, and assessment of outcome is unlikely to have been affected by knowledge of intervention received. |
| Bias in selection of reported result (Reporting bias) | Low risk | Main outcome measure is a composite rate of ICU transfer for mechanical ventilation and death. Although it is unclear if authors selected the reported outcome measures a priori, they are objectively the two most important outcomes for severe COVID-19, and separate analysis of each (i.e. ICU transfer rate and mortality rate) also yielded the same result. |

Capra et al. “Impact of low dose tocilizumab on mortality rate in patients with COVID-19 related pneumonia”.

ROBINS-I

| Bias | Author’s judgment | Support for judgment |
| --- | --- | --- |
| **Pre-intervention domains** | | |
| Bias due to confounding  (Confounding) | Low risk | Retrospective observational study. Adjustments for potential confounding made using multivariable Cox proportional hazards model. |
| Bias in selection of participants into the study  (Selection bias) | Low risk | Consecutive patients included if inclusion criteria were met. Only patients who initiated tocilizumab within 4 days of hospital admission were included in the treatment group; therefore, start of follow-up and start of treatment were considered to coincide. |
| **At-intervention domain** | | |
| Bias in classification of interventions  (Information bias) | Low risk | Intervention of interest is tocilizumab therapy. Intervention status is well-defined, and intervention definition is based solely on information collected at time of intervention. |
| **Post-intervention domains** | | |
| Bias due to deviations from intended interventions  (Confounding) | Low risk | Deviations from intended intervention likely reflect usual clinical practice. |
| Bias due to missing data  (Selection bias) | Low risk | Data for main outcome were considered complete. |
| Bias in measurement of the outcome  (Information bias) | Low risk | Main outcome measure was mortality, which is unlikely to be affected by the assessors’ knowledge of intervention received by patients |
| Bias in selection of reported result (Reporting bias) | Low risk | Although it is unclear if authors selected the reported outcome measures a priori, the objectively most important outcome for severe/critical COVID-19 is mortality, and this is used as the main outcome measure. |

Yuan et al. “Effects of corticosteroid treatment for non-severe COVID-19 pneumonia: a propensity score-based analysis.”

ROBINS-I

| Bias | Author’s judgment | Support for judgment |
| --- | --- | --- |
| **Pre-intervention domains** | | |
| Bias due to confounding  (Confounding) | Low risk | Retrospective cohort study. Propensity score matching used. |
| Bias in selection of participants into the study  (Selection bias) | Low risk | All patients in the enrolment period were screened for eligibility; treatment started at a median of 1.9 days of hospital admission, and thus start of follow-up and start of treatment were considered to coincide. |
| **At-intervention domain** | | |
| Bias in classification of interventions  (Information bias) | Low risk | Intervention of interest is corticosteroid therapy. Intervention status is well-defined, and intervention definition is based solely on information collected at time of intervention. |
| **Post-intervention domains** | | |
| Bias due to deviations from intended interventions  (Confounding) | Low risk | Deviations from intended intervention likely reflect usual clinical practice. |
| Bias due to missing data  (Selection bias) | No information | Concern of missing data is mentioned in the discussion, but no further details are provided. |
| Bias in measurement of the outcome  (Information bias) | Serious risk | Main outcome measures are clinical and radiological progression of disease. Outcome measures may be influenced by knowledge of intervention received by study participants; furthermore, no clear definition of clinical progression to severe case was delineated in the study. |
| Bias in selection of reported result (Reporting bias) | No information | Main outcome measure is progression of disease; unclear if the outcome measure was selected a priori. |

Skipper et al. “Hydroxychloroquine in nonhospitalized adults with early COVID-19 – a randomized trial”.

(Only the adverse event-related data from this study was used in this network meta-analysis)

RoB 2 tool

| Bias | Author’s judgement | Support for judgement |
| --- | --- | --- |
| Bias arising from the randomization process | Low risk | Central randomization and allocation used to conceal allocation sequence. No baseline imbalances. |
| Bias due to deviations from intended interventions | Low risk | No deviations from intended intervention due to trial context are expected. |
| Bias due to missing outcome data | Some concerns | Missing outcome data for adverse events (which is the data used for this review) could depend on the true value but does not seem likely in the context. |
| Bias in measurement of the outcome | Low risk | Assessors were unaware of intervention received by study participants (double-blind). |
| Bias in selection of the reported result | Low risk | All types of adverse events were noted and therefore there is minimal concern for selection of reported results for adverse event data. |

Miller et al. “Auxora versus standard of care for the treatment of severe or critical COVID-19 pneumonia: results from a randomized controlled trial”.

RoB 2 tool

| Bias | Author’s judgement | Support for judgement |
| --- | --- | --- |
| Bias arising from the randomization process | Some concerns | Not enough information is provided on the allocation process; there are no baseline demographics to suggest a problem for the low flow supplemental oxygen group (Arm A). Only 4 cases were included for high flow supplemental oxygen group (Arm B), making assessment difficult. |
| Bias due to deviations from intended interventions | Some concerns | Open label trial. Knowledge of treatment assignment could have influenced clinical decisions. |
| Bias due to missing outcome data | Low risk | Data were complete. |
| Bias in measurement of the outcome | Some concerns | Open label trial. Assessment of outcome on the 8-point scale unlikely to have been influenced by knowledge of intervention but not impossible. |
| Bias in selection of the reported result | Low risk | The study is conducted in accordance with a pre-specified protocol (found on ClinicalTrials.gov). |

Lecronier et al. “Comparison of hydroxychloroquine, lopinavir/ritonavir, and standard of care in critically ill patients with SARS-CoV-2 pneumonia: an opportunistic retrospective analysis”.

ROBINS-I

| Bias | Author’s judgment | Support for judgment |
| --- | --- | --- |
| **Pre-intervention domains** | | |
| Bias due to confounding  (Confounding) | Moderate risk | Retrospective analysis. Although respiratory rate is significantly different between treatment groups and no adjustments were made, severity index (SOFA) was comparable between groups; and thus we judged that respiratory rate was unlikely to substantially impact treatment outcomes. |
| Bias in selection of participants into the study  (Selection bias) | Low risk | All eligible patients were included; start of follow-up and start of intervention are considered to coincide. |
| **At-intervention domain** | | |
| Bias in classification of interventions  (Information bias) | Low risk | Interventions are well-defined and based solely on information collected at time of intervention. |
| **Post-intervention domains** | | |
| Bias due to deviations from intended interventions  (Confounding) | Low risk | Co-interventions were balanced across intervention groups, and deviations from intended interventions are unlikely. |
| Bias due to missing data  (Selection bias) | Low risk | Data were complete. |
| Bias in measurement of the outcome  (Information bias) | Low risk | Methods of outcome assessment were the same for all intervention groups and unlikely to be influenced by knowledge of the intervention received. Errors in measurement of outcomes are unlikely. |
| Bias in selection of reported result (Reporting bias) | No information | Main outcome measure is the number of patients needing treatment escalation in the first month of treatment; unclear if the outcome measure was selected a priori. |

Ivashchenko et al. “Avifavir for treatment of patients with moderate COVID-19: interim results of a phase II/III multicenter randomized clinical trial”.

RoB 2 tool

| Bias | Author’s judgement | Support for judgement |
| --- | --- | --- |
| Bias arising from the randomization process | Some concerns | There is no information on allocation sequence and randomization. There is also not enough information on the baseline characteristics of different intervention groups. |
| Bias due to deviations from intended interventions | High risk | Deviations may have arose from intended treatments as patients in the control group were allowed to use other antiviral/antimalarial medications while avifavir group patients were not. |
| Bias due to missing outcome data | Low risk | Data were complete. |
| Bias in measurement of the outcome | Low risk | Open label study but the primary endpoint of SARS-CoV-2 elimination defined by two negative PCR tests is unlikely to have been affected by knowledge of intervention. |
| Bias in selection of the reported result | Some concerns | Not enough information to determine whether the reported data were selected based on the results of multiple outcome measurements or multiple analyses of data. |

Nelson et al. “Clinical outcomes associated with methylprednisolone in mechanically ventilated patients with COVID-19”.

ROBINS-I

| Bias | Author’s judgment | Support for judgment |
| --- | --- | --- |
| **Pre-intervention domains** | | |
| Bias due to confounding  (Confounding) | Moderate risk | Propensity-score matching was used to yield a cohort of 42 pairs. Although rate of hydroxychloroquine/azithromycin usage was different between groups even after matching, there is increasing evidence that these medications do not confer a substantial effect on patient outcome. |
| Bias in selection of participants into the study  (Selection bias) | Moderate risk | All consecutive COVID-19 patients requiring intubation and mechanical ventilation were included, but start of intervention and start of follow-up do not necessarily coincide for all patients in methylprednisolone group. |
| **At-intervention domain** | | |
| Bias in classification of interventions  (Information bias) | Low risk | Intervention status is well-defined and based solely on information collected at time of intervention. |
| **Post-intervention domains** | | |
| Bias due to deviations from intended interventions  (Confounding) | Low risk | Any deviations from intended intervention likely reflect usual practice. |
| Bias due to missing data  (Selection bias) | Low risk | Data were complete |
| Bias in measurement of the outcome  (Information bias) | Low risk | Outcome assessment were the same between groups, and the primary outcome of ventilator-free days is deemed unlikely to have been influenced by knowledge of intervention by patients or assessors. |
| Bias in selection of reported result (Reporting bias) | No information | Main outcome measure is the number of ventilator-free days in the first 28 days of treatment; unclear if the outcome measure was selected a priori. |

Mitjà et al. “Hydroxychloroquine for early treatment of adults with mild COVID-19: a randomized-controlled trial”.

RoB 2 tool

| Bias | Author’s judgement | Support for judgement |
| --- | --- | --- |
| Bias arising from the randomization process | Low risk | Random allocation done remotely by a researcher not involved in patient enrollment. No baseline imbalances to suggest a problem. |
| Bias due to deviations from intended interventions | Some concerns | Open label trial. Knowledge of treatment assignment could have influenced patient behavior and deviations from intended interventions. |
| Bias due to missing outcome data | Some concerns | Missingness could but is unlikely to be dependent on true value. |
| Bias in measurement of the outcome | Low risk | Outcome assessors were unaware of the interventions received by patients. |
| Bias in selection of the reported result | Some concerns | Not enough information to determine whether the reported primary outcomes were selected based on the results of multiple outcome measurements or multiple analyses of data. |

Jeronimo et al. “Methylprednisolone as adjunctive therapy for patients hospitalized with COVID-19 (Metcovid): a randomized, double-blined, phase IIb, placebo-controlled trial”.

RoB 2 tool

| Bias | Author’s judgement | Support for judgement |
| --- | --- | --- |
| Bias arising from the randomization process | Low risk | Randomization sequence was generated by an independent statistician and was concealed. No baseline imbalances to suggest a problem. |
| Bias due to deviations from intended interventions | Low risk | In this placebo-controlled trial, no deviations are likely to have arisen from the trial context |
| Bias due to missing outcome data | Some concerns | Missingness could but is deemed unlikely to be dependent on true value. |
| Bias in measurement of the outcome | Low risk | Outcome assessors were unaware of interventions received. |
| Bias in selection of the reported result | Low risk | The study was conducted according to the protocol provided in the supplementary materials. |

Olender et al. “Remdesivir for severe COVID-19 versus a cohort receiving standard of care”.

ROBINS-I

| Bias | Author’s judgment | Support for judgment |
| --- | --- | --- |
| **Pre-intervention domains** | | |
| Bias due to confounding  (Confounding) | Moderate risk | Propensity score method used, but there was still imbalance in the use of hydroxychloroquine even after inverse probability of treatment weighting. This imbalance was adjusted for in the final weighted logistic regression analysis. |
| Bias in selection of participants into the study  (Selection bias) | Low risk | Patients were taken from two separate studies designed to align with each other. One is a open label randomized controlled trial; the other is a retrospective longitudinal cohort study. |
| **At-intervention domain** | | |
| Bias in classification of interventions  (Information bias) | Low risk | Intervention status is well-defined and based solely on information collected at time of intervention. |
| **Post-intervention domains** | | |
| Bias due to deviations from intended interventions  (Confounding) | Moderate risk | The open-label design of the study from which the remdesivir data were drawn may have led to deviations from intended intervention for some patients, but this effect is not deemed impactful. |
| Bias due to missing data  (Selection bias) | Low risk | Data were deemed complete for both arms. |
| Bias in measurement of the outcome  (Information bias) | Moderate risk | Outcome assessment were the same between groups, but the definition of recovery used in this investigation is potentially mildly influenced by knowledge of the intervention received. |
| Bias in selection of reported result (Reporting bias) | No information | The 7-point scale for assessment was defined a priori on ClinicalTrials.gov, but the definition of recovery used here is not. |

Arshad et al. “Treatment with hydroxychloroquine, azithromycin, and combination in patients hospitalized with COVID-19”.

ROBINS-I

| Bias | Author’s judgment | Support for judgment |
| --- | --- | --- |
| **Pre-intervention domains** | | |
| Bias due to confounding  (Confounding) | Low risk | Cox regression model and propensity score matching methods used to adjust for confounders. |
| Bias in selection of participants into the study  (Selection bias) | Low risk | All consecutive patients were included, and beginning of follow-up and intervention were considered to coincide as median time from admission to treatment was 1 day for hydroxychloroquine. |
| **At-intervention domain** | | |
| Bias in classification of interventions  (Information bias) | Low risk | Intervention status is well-defined and defined solely on information collected at time of intervention. |
| **Post-intervention domains** | | |
| Bias due to deviations from intended interventions  (Confounding) | Low risk | Deviations from intended intervention likely reflect usual practice. |
| Bias due to missing data  (Selection bias) | Low risk | Data regarding mortality were likely reasonably complete; and to mitigate missingness in electronic health records, the authors state that they conducted a careful manual review of the records. |
| Bias in measurement of the outcome  (Information bias) | Low risk | Main outcome measure was mortality, which is unlikely to be affected by the assessors’ knowledge of intervention received by patients |
| Bias in selection of reported result (Reporting bias) | Low risk | Although it is unclear if authors selected the reported outcome measures a priori, the objectively most important outcome for severe/critical COVID-19 is mortality, and this is used as the main outcome measure. |

Biran et al. “Tocilizumab among patients with COVID-19 in the intensive care unit: a multicentre observational study”.

ROBINS-I

| Bias | Author’s judgment | Support for judgment |
| --- | --- | --- |
| **Pre-intervention domains** | | |
| Bias due to confounding  (Confounding) | Low risk | Multivariable Cox regression with propensity score matching used to adjust for confounding. |
| Bias in selection of participants into the study  (Selection bias) | Low risk | All eligible patients in a prospective observational database were included. Start of follow-up and intervention were considered to coincide as tocilizumab was administered within in median of 0 days from the day of ICU care. |
| **At-intervention domain** | | |
| Bias in classification of interventions  (Information bias) | Low risk | Intervention status is well-defined and defined solely on information collected at time of intervention. |
| **Post-intervention domains** | | |
| Bias due to deviations from intended interventions  (Confounding) | Low risk | Deviations from intended intervention likely reflect usual practice. |
| Bias due to missing data  (Selection bias) | Low risk | Missing data were meticulously noted, and sensitivity analysis was performed to show the result did not drastically differ when patients with missing data were excluded. |
| Bias in measurement of the outcome  (Information bias) | Low risk | Main outcome measure was mortality, which is unlikely to be affected by the assessors’ knowledge of intervention received by patients. |
| Bias in selection of reported result (Reporting bias) | Low risk | Although it is unclear if authors selected the reported outcome measures a priori, the objectively most important outcome for severe/critical COVID-19 is mortality, and this is used as the main outcome measure. |

RECOVERY trial. “Dexamethasone in hospitalized patients with Covid-19 – preliminary report”.

RoB 2 tool

| Bias | Author’s judgement | Support for judgement |
| --- | --- | --- |
| Bias arising from the randomization process | Low risk | Allocation sequence was concealed and random. Although age was a significantly imbalanced baseline characteristic, the authors accounted for this imbalance by adjusting the rate ratios for baseline age in three categories. |
| Bias due to deviations from intended interventions | Some concerns | Open label trial. Knowledge of treatment assignment could have influenced patient behavior and deviations from intended interventions. |
| Bias due to missing outcome data | Low risk | Data were complete |
| Bias in measurement of the outcome | Low risk | Main outcome measure was mortality, which is unlikely to be affected by the assessors’ knowledge of intervention received by patients |
| Bias in selection of the reported result | Low risk | The trial is conducted and reported in accordance to the trial protocol available on recoverytrial.net. |

Cavalcanti et al. “Hydroxychloroquine with or without azithromycin in mild-to-moderate Covid-19”.

RoB 2 tool

| Bias | Author’s judgement | Support for judgement |
| --- | --- | --- |
| Bias arising from the randomization process | Low risk | An independent, uninvolved statistician generated randomization tables; and allocation sequence was adequately concealed. No baseline imbalances to suggest a problem. |
| Bias due to deviations from intended interventions | Some concerns | Open label trial. Knowledge of treatment assignment could have influenced patient behavior and deviations from intended interventions. |
| Bias due to missing outcome data | Low risk | Data were reasonably complete. |
| Bias in measurement of the outcome | Some concerns | Open label trial. Assessment of outcome on the 7-point scale possibly but not likely to have been influenced by knowledge of intervention. |
| Bias in selection of the reported result | Low risk | While there is no prepublished protocol, the appendix provides a detailed account of the protocol establishment and revision. Selective reporting bias is thus deemed unlikely. |

Ip et al. “Hydroxychloroquine and tocilizumab therapy in COVID-19 patients – an observational study”.

ROBINS-I

| Bias | Author’s judgment | Support for judgment |
| --- | --- | --- |
| **Pre-intervention domains** | | |
| Bias due to confounding  (Confounding) | Low risk | Propensity modeling used to adjust for confounders. |
| Bias in selection of participants into the study  (Selection bias) | Low risk | All eligible patients were included in the study. As median number of days in hospital before first dose was 1 for hydroxychloroquine, start of intervention and start of follow-up were considered to coincide. For tocilizumab, not enough information is present to determine whether start of follow-up and intervention coincided. |
| **At-intervention domain** | | |
| Bias in classification of interventions  (Information bias) | Low risk | Intervention status is well-defined and defined solely on information collected at time of intervention. |
| **Post-intervention domains** | | |
| Bias due to deviations from intended interventions  (Confounding) | Low risk | Deviations from intended intervention likely reflect usual practice. |
| Bias due to missing data  (Selection bias) | Low risk | Data were reasonably complete. Some data regarding baseline characteristics were missing, but this is not deemed to be an impactful source of bias. |
| Bias in measurement of the outcome  (Information bias) | Low risk | Main outcome measure was mortality, which is unlikely to be affected by the assessors’ knowledge of intervention received by patients. |
| Bias in selection of reported result (Reporting bias) | Low risk | Although it is unclear if authors selected the reported outcome measures a priori, the objectively most important outcome for severe/critical COVID-19 is mortality, and this is used as the main outcome measure. |

Ma et al. “Corticosteroid therapy for patients with severe novel Coronavirus disease 2019”.

ROBINS-I

| Bias | Author’s judgment | Support for judgment |
| --- | --- | --- |
| **Pre-intervention domains** | | |
| Bias due to confounding  (Confounding) | Moderate risk | Baseline characteristics are established to be similar, but no adjustments were made for confounders. |
| Bias in selection of participants into the study  (Selection bias) | Moderate risk | All eligible patients were included, but start of follow-up and start of corticosteroid treatment do not necessarily coincide for all patients. |
| **At-intervention domain** | | |
| Bias in classification of interventions  (Information bias) | Low risk | Intervention status is well-defined and defined solely on information collected at time of intervention. |
| **Post-intervention domains** | | |
| Bias due to deviations from intended interventions  (Confounding) | Low risk | Deviations from intended intervention likely reflect usual practice. |
| Bias due to missing data  (Selection bias) | Low risk | Data were complete. |
| Bias in measurement of the outcome  (Information bias) | Low risk | Primary outcomes were mortality, hospital length of stay, and time to viral clearance. Methods of outcome assessment were the same for both treatment arms, and these outcomes were deemed largely unlikely to be influenced by knowledge of the intervention. |
| Bias in selection of reported result (Reporting bias) | Moderate risk | It is unclear whether the outcome measures were determined a priori, but the use of multiple primary and secondary endpoints raises concerns for multiplicity. |

Hu et al. “Clinical use of short-course and low-dose corticosteroids in patients with non-severe COVID-19 during pneumonia progression”.

ROBINS-I

| Bias | Author’s judgment | Support for judgment |
| --- | --- | --- |
| **Pre-intervention domains** | | |
| Bias due to confounding  (Confounding) | Low risk | Inverse probability weighting used to adjust for confounders. |
| Bias in selection of participants into the study  (Selection bias) | Moderate risk | All eligible patients were included, but start of follow-up and intervention do not necessarily coincide for all patients as corticosteroids were administered when symptoms or radiological findings did not subside. |
| **At-intervention domain** | | |
| Bias in classification of interventions  (Information bias) | Low risk | Intervention status is well-defined and defined solely on information collected at time of intervention. |
| **Post-intervention domains** | | |
| Bias due to deviations from intended interventions  (Confounding) | Serious risk | IVIG was co-administered with corticosteroids, acting as a potentially significant confounder. |
| Bias due to missing data  (Selection bias) | Low risk | Data were presented as complete. |
| Bias in measurement of the outcome  (Information bias) | Low risk | Main outcome measures of progression to severe disease and viral clearance are deemed unlikely to be affected by knowledge of intervention. |
| Bias in selection of reported result (Reporting bias) | No information | No information as to whether authors selected outcomes measure a priori. |

Abolghasemi et al. “Clinical efficacy of convalescent plasma for treatment of COVID-19 infections: results of a multicenter clinical study”.

ROBINS-I

| Bias | Author’s judgment | Support for judgment |
| --- | --- | --- |
| **Pre-intervention domains** | | |
| Bias due to confounding  (Confounding) | Moderate risk | Control group consisted of patients with mild symptoms or patients who did not have matching donor convalescent plasma; the two treatment groups were matched for age, gender, hypertension, diabetes, and severity based on CT. |
| Bias in selection of participants into the study  (Selection bias) | Low risk | All eligible patients were included, and start of follow-up and intervention are deemed to coincide as convalescent plasma was administered within 3 days of admission. |
| **At-intervention domain** | | |
| Bias in classification of interventions  (Information bias) | Low risk | Intervention status is well-defined and defined solely on information collected at time of intervention. |
| **Post-intervention domains** | | |
| Bias due to deviations from intended interventions  (Confounding) | Moderate risk | Plasma transfusions may have led to deviations that could have affected the clinical outcome; however, they are mostly considered to reflect usual clinical practice. |
| Bias due to missing data  (Selection bias) | Low risk | Data were complete. |
| Bias in measurement of the outcome  (Information bias) | Low risk | Method of outcome assessment was equivalent in both arms, and majority of outcome measures (mortality and intubation rates) were deemed unlikely to be influenced by knowledge of intervention. |
| Bias in selection of reported result (Reporting bias) | Moderate risk | Multiple endpoints are presented – mortality, hospital stay, intubation – raising concern for multiplicity. |

Chen et al. “Antiviral activity and safety of darunavir/cobicistat for the treatment of COVID-19”.

RoB 2 tool

| Bias | Author’s judgement | Support for judgement |
| --- | --- | --- |
| Bias arising from the randomization process | High risk | Randomization was based on parity of medical record number and allocation was therefore unlikely to be concealed. |
| Bias due to deviations from intended interventions | Some concerns | Open label trial. Knowledge of treatment assignment could have influenced patient behavior and deviations from intended interventions. |
| Bias due to missing outcome data | Low risk | Data were complete. |
| Bias in measurement of the outcome | Low risk | Primary outcome measure was viral clearance at day 7 determined by RT-PCR, which is unlikely to be affected by the assessors’ knowledge of intervention received by patients |
| Bias in selection of the reported result | Some concerns | Unclear if the outcome measure was selected a priori. |

Cantini et al. “Beneficial impact of baricitinib in COVID-19 moderate pneumonia; multicentre study”.

ROBINS-I

| Bias | Author’s judgment | Support for judgment |
| --- | --- | --- |
| **Pre-intervention domains** | | |
| Bias due to confounding  (Confounding) | Moderate risk | Baseline characteristics were shown to be similar. Although the control group was treated with hydroxychloroquine while the baricitinib group was not, with increasing evidence that hydroxychloroquine does not substantially impact patient outcome, this risk of confounding is not deemed serious. |
| Bias in selection of participants into the study  (Selection bias) | Low risk | All eligible patients were consecutively included. Start of follow-up and start of intervention coincided. |
| **At-intervention domain** | | |
| Bias in classification of interventions  (Information bias) | Low risk | Intervention status is well-defined and defined solely on information collected at time of intervention. |
| **Post-intervention domains** | | |
| Bias due to deviations from intended interventions  (Confounding) | Low risk | Deviations from intended intervention likely reflect usual practice. |
| Bias due to missing data  (Selection bias) | Low risk | Data were complete. |
| Bias in measurement of the outcome  (Information bias) | Low risk | Primary outcomes were mortality in two weeks. Methods of outcome assessment were the same for both treatment arms, and this outcome is deemed unlikely to be influenced by knowledge of the intervention. |
| Bias in selection of reported result (Reporting bias) | Low risk | Although it is unclear if authors selected the reported outcome measures a priori, the objectively most important outcome for severe/critical COVID-19 is mortality, and this is used as the main outcome measure. |

Wang et al. “Exploring an integrative therapy for treating COVID-10: a randomized controlled trial”.

RoB 2 tool

| Bias | Author’s judgement | Support for judgement |
| --- | --- | --- |
| Bias arising from the randomization process | Low risk | Random allocation was prepared by an independent statistician and was concealed. There were no baseline imbalances to suggest a problem. |
| Bias due to deviations from intended interventions | Some concerns | Open label trial. However, no significant deviations from the usual treatment course is expected for both intervention and control groups. |
| Bias due to missing outcome data | Low risk | Data were complete. |
| Bias in measurement of the outcome | Some concerns | Main outcome measure was incidence of ARDS. Outcome assessors were aware of the intervention received, and it is possible though unlikely that the outcome assessment was influenced by this knowledge. |
| Bias in selection of the reported result | Some concerns | The primary outcome measure was changed during the trial. |

Ramiro et al. “Historically controlled comparison of glucocorticoids with or without tocilizumab versus supportive care only in patients with COVID-19-associated cytokine storm syndrome: results of the CHIC study”.

ROBINS-I

| Bias | Author’s judgment | Support for judgment |
| --- | --- | --- |
| **Pre-intervention domains** | | |
| Bias due to confounding  (Confounding) | Low risk | Cox regression analysis was used to adjust for confounders. |
| Bias in selection of participants into the study  (Selection bias) | Low risk | All eligible participants were enrolled, and start of follow-up and intervention coincided as methylprednisolone was administered on the day cytokine storm was identified in a patient. |
| **At-intervention domain** | | |
| Bias in classification of interventions  (Information bias) | Low risk | Intervention status is well-defined and defined solely on information collected at time of intervention. |
| **Post-intervention domains** | | |
| Bias due to deviations from intended interventions  (Confounding) | Low risk | Deviations from intended interventions likely reflect usual practice for cytokine storm syndrome patients. |
| Bias due to missing data  (Selection bias) | Low risk | Data were complete. Certain laboratory values were missing (e.g. D-dimer) in a large proportion of the patients, but this was not deemed significant as there would have been no difficulty in establishing cytokine storm syndrome diagnosis without it and the outcomes are not impacted. |
| Bias in measurement of the outcome  (Information bias) | Moderate risk | Outcome measure is possibly but unlikely to have been impacted by the knowledge of intervention received. |
| Bias in selection of reported result (Reporting bias) | No information | No information as to whether the outcome measure WHO-scale was selected a priori to the investigation. |

Karolyi et al. “Hydroxychloroquine versus lopinavir/ritonavir in severe COVID-19 patients: Results from a real-life patient cohort”.

ROBINS-I

| Bias | Author’s judgment | Support for judgment |
| --- | --- | --- |
| **Pre-intervention domains** | | |
| Bias due to confounding  (Confounding) | Serious risk | Retrospective study. Baseline imbalances were present in hypertension and coronary heart disease, and no adjustments were made. |
| Bias in selection of participants into the study  (Selection bias) | Low risk | All eligible patients were included, and start of follow-up and intervention were considered to coincide as median time from symptom onset to hospitalization was 7 days and median time from symptom onset to antiviral treatment was 8 days for both groups. |
| **At-intervention domain** | | |
| Bias in classification of interventions  (Information bias) | Low risk | Intervention status is well-defined and defined solely on information collected at time of intervention. |
| **Post-intervention domains** | | |
| Bias due to deviations from intended interventions  (Confounding) | Low risk | Deviations from intended intervention are likely to reflect usual practice. |
| Bias due to missing data  (Selection bias) | Low risk | Data for main outcomes were complete. |
| Bias in measurement of the outcome  (Information bias) | Low risk | Primary outcomes were mortality (in-hospital) and ICU admission. Methods of outcome assessment were the same for both treatment arms, and these outcomes are deemed unlikely to be influenced by knowledge of the intervention. |
| Bias in selection of reported result (Reporting bias) | Low risk | Although it is unclear if authors selected the reported outcome measures a priori, the objectively most important outcome for severe/critical COVID-19 is mortality, and this is used as a main outcome measure. |

Lian et al. “Umifenovir treatment is not associated with improved outcomes in patients with coronavirus disease 2019: a retrospective study”.

ROBINS-I

| Bias | Author’s judgment | Support for judgment |
| --- | --- | --- |
| **Pre-intervention domains** | | |
| Bias due to confounding  (Confounding) | Low risk | Baseline characteristics of the intervention and control groups were considered similar except for CT scores; subgroup analysis based on CT scores was performed, and results were shown to be similar. |
| Bias in selection of participants into the study  (Selection bias) | Low risk | All eligible patients were included. Start of intervention and follow-up were considered to coincide as umifenovir was given on admission or within 24 hours. |
| **At-intervention domain** | | |
| Bias in classification of interventions  (Information bias) | Low risk | Intervention status is well-defined and defined solely on information collected at time of intervention. |
| **Post-intervention domains** | | |
| Bias due to deviations from intended interventions  (Confounding) | Low risk | Deviations from intended intervention are likely to reflect usual practice. |
| Bias due to missing data  (Selection bias) | Low risk | Data were complete (those with crucial missing data were excluded from the analysis – 6 patients). |
| Bias in measurement of the outcome  (Information bias) | Low risk | Main outcome measure of pharyngeal swab for SARS-CoV-1 is unlikely to be influenced by knowledge of intervention. |
| Bias in selection of reported result (Reporting bias) | No information | No information as to whether the outcome measures were selected a priori. |

Hao et al. “Interferon-α2b spray inhalation did not shorten virus shedding time of SARS-CoV-2 in hospitalized patients: a preliminary matched case-control study”.

ROBINS-I

| Bias | Author’s judgment | Support for judgment |
| --- | --- | --- |
| **Pre-intervention domains** | | |
| Bias due to confounding  (Confounding) | Low risk | Propensity score matching used to balance confounders. |
| Bias in selection of participants into the study  (Selection bias) | Low risk | All eligible patients were entered, and start of intervention and follow-up were considered to coincide as interferon was given on admission. |
| **At-intervention domain** | | |
| Bias in classification of interventions  (Information bias) | Low risk | Intervention status is well-defined and defined solely on information collected at time of intervention. |
| **Post-intervention domains** | | |
| Bias due to deviations from intended interventions  (Confounding) | Low risk | Deviations from intended intervention are likely to reflect usual practice. |
| Bias due to missing data  (Selection bias) | Low risk | Data were complete. |
| Bias in measurement of the outcome  (Information bias) | Low risk | Viral shedding was assessed by assaying respiratory specimen for SARS-CoV-2 RNA. The method was the same for the two groups, and this outcome measure is unlikely to be influenced by knowledge of intervention. |
| Bias in selection of reported result (Reporting bias) | No information | No information as to whether the outcome measures were selected a priori. |

Canziani et al. “Interleukin-6 receptor blocking with intravenous tocilizumab in COVID-19 severe acute respiratory distress syndrome: a retrospective case-control survival analysis of 128 patients”.

ROBINS-I

| Bias | Author’s judgment | Support for judgment |
| --- | --- | --- |
| **Pre-intervention domains** | | |
| Bias due to confounding  (Confounding) | Low risk | Multivariable survival analysis conducted using variables that proved significant in univariable Cox analysis. |
| Bias in selection of participants into the study  (Selection bias) | Moderate risk | Not all eligible patients were included because of insufficiency of drug availability during a period of excessive demand. |
| **At-intervention domain** | | |
| Bias in classification of interventions  (Information bias) | Low risk | Intervention status is well-defined and defined solely on information collected at time of intervention. |
| **Post-intervention domains** | | |
| Bias due to deviations from intended interventions  (Confounding) | Moderate risk | This investigation was conducted at a time of patient surge and low availability of treatment facilities. The timing of patient admission and drug/supportive care measure availability may have impacted treatment course. |
| Bias due to missing data  (Selection bias) | Low risk | Data were complete. |
| Bias in measurement of the outcome  (Information bias) | Low risk | Primary outcome was mortality (30-days). Methods of outcome assessment were the same for both treatment arms, and this outcome is deemed unlikely to be influenced by knowledge of the intervention. |
| Bias in selection of reported result (Reporting bias) | Low risk | Although it is unclear if authors selected the reported outcome measures a priori, the objectively most important outcome for severe/critical COVID-19 is mortality, and this is used as a main outcome measure. (Seeing as the authors provide a post hoc analysis of mortality between 6-30 days that do favor tocilizumab as a treatment, it seems likely that the primary outcome presented here is the originally selected outcome measure). |

Fang et al. “Low-dose corticosteroid therapy does not delay viral clearance in patients with COVID-19”.

ROBINS-I

| Bias | Author’s judgment | Support for judgment |
| --- | --- | --- |
| **Pre-intervention domains** | | |
| Bias due to confounding  (Confounding) | Moderate risk | Corticosteroids were administered to patients with more severe disease. Patients were divided into a general and severe group, and data analysis was conducted separately. Even still, there were imbalances in the baseline characteristics within general/severe groups (e.g. albumin), but these are not deemed serious. |
| Bias in selection of participants into the study  (Selection bias) | Moderate risk | All eligible patients were included, but start of follow-up and intervention did not necessarily coincide for all patients. |
| **At-intervention domain** | | |
| Bias in classification of interventions  (Information bias) | Low risk | Intervention status is well-defined and defined solely on information collected at time of intervention. |
| **Post-intervention domains** | | |
| Bias due to deviations from intended interventions  (Confounding) | Low risk | Deviations from intended intervention are likely to reflect usual practice. |
| Bias due to missing data  (Selection bias) | Low risk | Data were complete. |
| Bias in measurement of the outcome  (Information bias) | Low risk | Throat swab or sputum samples collected to test for viral clearance with RT-PCR. Unlikely to have been affected by knowledge of treatment. |
| Bias in selection of reported result (Reporting bias) | No information | No information as to whether the outcome measures were selected a priori. |

Kim et al. “Lopinavir-ritonavir versus hydroxychloroquine for viral clearance and clinical improvement in patients with mild to moderate coronavirus disease 2019”.

ROBINS-I

| Bias | Author’s judgment | Support for judgment |
| --- | --- | --- |
| **Pre-intervention domains** | | |
| Bias due to confounding  (Confounding) | Low risk | Cox proportional hazards regression used to find factors associated with primary endpoint. |
| Bias in selection of participants into the study  (Selection bias) | Moderate risk | All eligible patients were included. Unclear if the start of follow-up and intervention coincided for all patients. |
| **At-intervention domain** | | |
| Bias in classification of interventions  (Information bias) | Low risk | Intervention status is well-defined and defined solely on information collected at time of intervention. |
| **Post-intervention domains** | | |
| Bias due to deviations from intended interventions  (Confounding) | Low risk | Deviations from intended intervention are likely to reflect usual practice. |
| Bias due to missing data  (Selection bias) | Low risk | Data were complete. |
| Bias in measurement of the outcome  (Information bias) | Low risk | Main outcome was viral clearance determined by two consecutive negative RT-PCR results. This is unlikely to be affected by knowledge of treatment. |
| Bias in selection of reported result (Reporting bias) | No information | No information as to whether outcome was selected a priori. |

Della-Torre et al. “Interleukin-6 blockade with sarilumab in severe COVID-19 pneumonia with systemic hyperinflammation: an open-label cohort study”.

ROBINS-I

| Bias | Author’s judgment | Support for judgment |
| --- | --- | --- |
| **Pre-intervention domains** | | |
| Bias due to confounding  (Confounding) | Low risk | Cox proportional hazards model used to analyze survival and baseline clinical/laboratory features. |
| Bias in selection of participants into the study  (Selection bias) | Low risk | All eligible patients were included. Start of follow-up and intervention were considered to coincide as sarilumab was initiated within 24hrs of fulfilling inclusion criteria. |
| **At-intervention domain** | | |
| Bias in classification of interventions  (Information bias) | Low risk | Intervention status is well-defined and defined solely on information collected at time of intervention. |
| **Post-intervention domains** | | |
| Bias due to deviations from intended interventions  (Confounding) | Low risk | Deviations from intended intervention are likely to reflect usual practice. |
| Bias due to missing data  (Selection bias) | Low risk | Data were complete. |
| Bias in measurement of the outcome  (Information bias) | Moderate risk | A main outcome of clinical improvement was measured by a 6-point ordinal scale. This was possibly but not likely to have been affected by knowledge of intervention received. |
| Bias in selection of reported result (Reporting bias) | No information | Unclear if outcome measures were selected a priori. Use of multiple endpoints raises concerns for multiplicity. |

Herrero et al. “Methylprednisolone added to tocilizumab reduces mortality in SARS-CoV-2 pneumonia: an observational study”.

ROBINS-I

| Bias | Author’s judgment | Support for judgment |
| --- | --- | --- |
| **Pre-intervention domains** | | |
| Bias due to confounding  (Confounding) | Serious risk | Cox regression used to find association between treatment arm and endpoint of death. Usage rate of interferon differed between the two groups. |
| Bias in selection of participants into the study  (Selection bias) | Low risk | All eligible patients were included, and start of intervention and follow-up are considered to coincide for all patients. |
| **At-intervention domain** | | |
| Bias in classification of interventions  (Information bias) | Low risk | Intervention status is well-defined and defined solely on information collected at time of intervention. |
| **Post-intervention domains** | | |
| Bias due to deviations from intended interventions  (Confounding) | Low risk | Deviations from intended intervention are likely to reflect usual practice. |
| Bias due to missing data  (Selection bias) | Low risk | Data were complete. |
| Bias in measurement of the outcome  (Information bias) | Low risk | Main outcome measure of mortality is unlikely to be affected by knowledge of intervention. |
| Bias in selection of reported result (Reporting bias) | Low risk | Although it is unclear whether the authors selected the main outcome measure a priori, mortality is the most important outcome for patients with severe disease; risk of selective reporting bias is deemed low. |

Huang et al. “No statistically apparent difference in antiviral effectiveness observed among ribavirin plus interferon-alpha, lopinavir/ritonavir plus interferon-alpha, and ribavirin plus lopinavir/ritonavir plus interferon-alpha in patients with mild to moderate coronavirus disease 2019: results of a randomized, open-labeled prospective study”.

RoB 2 tool

| Bias | Author’s judgement | Support for judgement |
| --- | --- | --- |
| Bias arising from the randomization process | Low risk | Based on the statement that random number sequence generation was conducted free from the potential influence of physicians, allocation sequence is assumed to be concealed. There are no baseline imbalances to suggest a problem. |
| Bias due to deviations from intended interventions | Low risk | Open label trial. However, no significant deviations from the usual treatment course is expected for both intervention and control groups, especially since all treatment groups were given medication. |
| Bias due to missing outcome data | Low risk | Data were complete. |
| Bias in measurement of the outcome | Low risk | Measurement of outcome by RT-PCR of nasopharyngeal swabs was the same for both groups. Outcome assessors (laboratory personnel) were blinded to treatment group. |
| Bias in selection of the reported result | Some concerns | No information as to the primary outcome measure was selected a priori. |

Wang et al. “Retrospective multicenter cohort study shows early interferon therapy is associated with favorable clinical response in COVID-19 patients”.

ROBINS-I

| Bias | Author’s judgment | Support for judgment |
| --- | --- | --- |
| **Pre-intervention domains** | | |
| Bias due to confounding  (Confounding) | Low risk | Logistic regression and Cox proportional hazards model used to adjust for confounders. |
| Bias in selection of participants into the study  (Selection bias) | Low risk | All eligible patients were included. Start of intervention was distinguished between patients and was a major focus of this study. |
| **At-intervention domain** | | |
| Bias in classification of interventions  (Information bias) | Low risk | Intervention status is well-defined and defined solely on information collected at time of intervention. |
| **Post-intervention domains** | | |
| Bias due to deviations from intended interventions  (Confounding) | Low risk | Deviations from intended intervention are likely to reflect usual practice. |
| Bias due to missing data  (Selection bias) | Low risk | Data were complete. |
| Bias in measurement of the outcome  (Information bias) | Low risk | Main outcome measure of mortality (in-hospital) is unlikely to be affected by knowledge of intervention. |
| Bias in selection of reported result (Reporting bias) | Low risk | Although it is unclear whether the authors selected the main outcome measure a priori, mortality is the most important outcome for patients with severe disease; risk of selective reporting bias is deemed low. |

Tong et al. “Ribavirin therapy for severe COVID-19: a retrospective cohort study”.

ROBINS-I

| Bias | Author’s judgment | Support for judgment |
| --- | --- | --- |
| **Pre-intervention domains** | | |
| Bias due to confounding  (Confounding) | Low risk | Multiple logistic regression used to evaluate association between ribavirin and mortality. |
| Bias in selection of participants into the study  (Selection bias) | Moderate risk | All eligible patients were included; but start of intervention and start of follow-up did not necessarily coincide for all patients (range of days between diagnosis and treatment initiation 1-12 days). |
| **At-intervention domain** | | |
| Bias in classification of interventions  (Information bias) | Low risk | Intervention status is well-defined and defined solely on information collected at time of intervention. |
| **Post-intervention domains** | | |
| Bias due to deviations from intended interventions  (Confounding) | Moderate risk | Ribavirin was sometimes out of stock and treatment thus withheld. |
| Bias due to missing data  (Selection bias) | Low risk | Data were complete. |
| Bias in measurement of the outcome  (Information bias) | Low risk | RT-PCR results are unlikely to be affected by knowledge of intervention. |
| Bias in selection of reported result (Reporting bias) | No information | No information as to whether the primary outcome of negative conversion was selected a priori. |

Rossotti et al. “Safety and efficacy of anti-il6-receptor tocilizumab use in severe and critical patients affected by coronavirus disease 2019: a comparative analysis”.

ROBINS-I

| Bias | Author’s judgment | Support for judgment |
| --- | --- | --- |
| **Pre-intervention domains** | | |
| Bias due to confounding  (Confounding) | Low risk | Patients were matched for various confounders, and Cox regression model used to determine risk of mortality. |
| Bias in selection of participants into the study  (Selection bias) | Low risk | All eligible patients were included. Start of follow-up and start of intervention coincided for all tocilizumab-administered patients. |
| **At-intervention domain** | | |
| Bias in classification of interventions  (Information bias) | Low risk | Intervention status is well-defined and defined solely on information collected at time of intervention. |
| **Post-intervention domains** | | |
| Bias due to deviations from intended interventions  (Confounding) | Low risk | Deviations from intended intervention are likely to reflect usual practice. |
| Bias due to missing data  (Selection bias) | Low risk | Data were complete. |
| Bias in measurement of the outcome  (Information bias) | Low risk | Main outcome measure of mortality is unlikely to be affected by knowledge of intervention. |
| Bias in selection of reported result (Reporting bias) | Low risk | Although it is unclear whether the authors selected the main outcome measure a priori, mortality is the most important outcome for patients with severe disease; risk of selective reporting bias is deemed low. |

Spinner et al. “Effect of remdesivir vs standard care on clinical status at 11 days in patients with moderate COVID-19 – a randomized clinical trial”.

RoB 2 tool

| Bias | Author’s judgement | Support for judgement |
| --- | --- | --- |
| Bias arising from the randomization process | Low risk | Allocation sequence was concealed and random. No baseline imbalances to suggest a problem. |
| Bias due to deviations from intended interventions | Some concerns | Open label trial without placebo. |
| Bias due to missing outcome data | Low risk | Sensitivity analysis imputing missing status as dead produced similar results. |
| Bias in measurement of the outcome | Some concerns | Open label trial without placebo. Rates of hospital discharge show that open label study design had an effect on patient discharge. |
| Bias in selection of the reported result | Low risk | The primary outcome measure of improvement on a 7-point ordinal scale on day 11 was prespecified (NCT04292730). (It was selected on the first day of enrollment). |

De Luca et al. “GM-CSF blockade with mavrilimumab in severe COVID-19 pneumonia and systemic hyperinflammation: a single-centre, prospective cohort study”.

ROBINS-I

| Bias | Author’s judgment | Support for judgment |
| --- | --- | --- |
| **Pre-intervention domains** | | |
| Bias due to confounding  (Confounding) | Low risk | Control patients were selected to be comparable for various confounders. |
| Bias in selection of participants into the study  (Selection bias) | Low risk | All eligible patients were included through a dedicated case report form based on the institutional protocol. Mavrilimumab was administered when patients met criteria for COVID-19 with systemic hyperinflammation; thus start of intervention and follow-up coincided. |
| **At-intervention domain** | | |
| Bias in classification of interventions  (Information bias) | Low risk | Intervention status is well-defined and defined solely on information collected at time of intervention. |
| **Post-intervention domains** | | |
| Bias due to deviations from intended interventions  (Confounding) | Low risk | Deviations from intended intervention are likely to reflect usual practice. |
| Bias due to missing data  (Selection bias) | Low risk | Data were complete. |
| Bias in measurement of the outcome  (Information bias) | Moderate risk | Endpoint using clinical score can potentially be influenced by knowledge of intervention received. |
| Bias in selection of reported result (Reporting bias) | No information | No information as to whether the designated primary endpoint was selected a priori. |

Guaraldi et al. “Tocilizumab in patients with severe COVID-19: a retrospective cohort study”.

ROBINS-I

| Bias | Author’s judgment | Support for judgment |
| --- | --- | --- |
| **Pre-intervention domains** | | |
| Bias due to confounding  (Confounding) | Low risk | Adjustments for confounders were made using Cox regression. Additional analyses were conducted as well (e.g. adjustment for baseline inflammation/coagulation, replacement of SOFA score with alternatives, stratification for P/F ratio and age) |
| Bias in selection of participants into the study  (Selection bias) | Moderate risk | All eligible patients were included, but it is unclear whether start of follow-up and intervention coincided for all patients. |
| **At-intervention domain** | | |
| Bias in classification of interventions  (Information bias) | Low risk | Intervention status is well-defined and defined solely on information collected at time of intervention. |
| **Post-intervention domains** | | |
| Bias due to deviations from intended interventions  (Confounding) | Moderate risk | Antiviral drugs were never started after tocilizumab in the tocilizumab group while they were continued for patients who received only standard care. |
| Bias due to missing data  (Selection bias) | Low risk | Data were complete |
| Bias in measurement of the outcome  (Information bias) | Low risk | Primary endpoint of intubation and mortality are deemed at low risk of information bias. |
| Bias in selection of reported result (Reporting bias) | Low risk | The primary endpoint was a composite measure of intubation and death. Although it is unclear whether this outcome was selected a priori, the authors also present that the results were similar for mortality alone; and as mortality is the most important endpoint for severe COVID-19 and intubation the second-most important, the risk of selective reporting bias is deemed low. |

Sadeghi et al. “Sofosbuvir and daclatasvir compared with standard of care in the treatment of patients admitted to hospital with moderate or severe coronavirus infection (COVID-19): a randomized controlled trial”.

RoB 2 tool

| Bias | Author’s judgement | Support for judgement |
| --- | --- | --- |
| Bias arising from the randomization process | Some concerns | Allocation sequence was random and concealed, but there was a baseline imbalance in the usage rate of lopinavir/ritonavir. |
| Bias due to deviations from intended interventions | High risk | Open label trial. Deviations are possible; of special concern is the difference in usage rate of lopinavir/ritonavir, which was attributed to doctors not feeling comfortable with prescribing an additional antiviral. |
| Bias due to missing outcome data | Low risk | Data were complete |
| Bias in measurement of the outcome | Some concerns | Main endpoint used was clinical recovery in 14 days as defined by recovery normalization of fever, respiratory rate, and oxygen saturation. This endpoint can be influenced by knowledge of treatment. |
| Bias in selection of the reported result | Some concerns | Unclear if the main endpoint was selected a priori. (Registered under IRCT20200128046294N2, but content was changed at 2020-06-01 while the manuscript was received 2020-05-29). |

Kasgari et al. “Evaluation of the efficacy of sofosbuvir plus daclatasvir in combination with ribavirin for hospitalized COVID-19 patients with moderate disease compared with standard care: a single-centre, randomized controlled trial”.

RoB 2 tool

| Bias | Author’s judgement | Support for judgement |
| --- | --- | --- |
| Bias arising from the randomization process | Some concerns | Allocation sequence was random and concealed, but there was an imbalance in the baseline characteristics (diabetes). Adjustment for baseline imbalances were made using Cox proportional hazards and competing risks regression models. |
| Bias due to deviations from intended interventions | High risk | Patients in control group were given hydroxychloroquine, lopinavir/ritonavir, or both while patients in the treatment arm were not. |
| Bias due to missing outcome data | Low risk | Data were complete. |
| Bias in measurement of the outcome | Some concerns | Primary outcome was length of hospital stay. It is potentially influenced by knowledge of intervention. |
| Bias in selection of the reported result | High risk | The primary outcome variable presented in the Iranian registry of clinical trials (IRCT20200328046886N1) is clinical recovery within 14 days, not length of hospital stay. |

Deftereos et al. “Effect of colchicine vs standard care on cardiac and inflammatory biomarker and clinical outcomes in patients hospitalized with coronavirus disease 2019: The GRECCO-19 randomized clinical trial”.

RoB 2 tool

| Bias | Author’s judgement | Support for judgement |
| --- | --- | --- |
| Bias arising from the randomization process | Low risk | Allocation was random and concealed. No baseline imbalances to suggest a problem. |
| Bias due to deviations from intended interventions | Some concerns | Open label trial. Deviations may have arose. |
| Bias due to missing outcome data | Low risk | Data were complete. |
| Bias in measurement of the outcome | Some concerns | Primary clinical endpoint of clinical improvement on a 7-point scale is potentially affected by physicians’ knowledge of intervention. |
| Bias in selection of the reported result | Some concerns | Unclear if the presented outcome measures were selected a priori. |

Eslami et al. “The impact of sofosbuvir/daclatasvir or ribavirin in patients with sever COVID-19”.

ROBINS-I

| Bias | Author’s judgment | Support for judgment |
| --- | --- | --- |
| **Pre-intervention domains** | | |
| Bias due to confounding  (Confounding) | Low risk | Cox proportional hazards model used to adjust for possible confounders. |
| Bias in selection of participants into the study  (Selection bias) | Low risk | This study was done in a trial format. All eligible patients were included; and start of follow-up and start of intervention coincided. |
| **At-intervention domain** | | |
| Bias in classification of interventions  (Information bias) | Low risk | Intervention status is well-defined and defined solely on information collected at time of intervention. |
| **Post-intervention domains** | | |
| Bias due to deviations from intended interventions  (Confounding) | Low risk | Deviations likely reflect usual clinical practice. |
| Bias due to missing data  (Selection bias) | Low risk | Data were complete. |
| Bias in measurement of the outcome  (Information bias) | Moderate risk | Primary endpoint was time from medication initiation to hospital discharge. It is potentially influenced by knowledge of intervention received. |
| Bias in selection of reported result (Reporting bias) | No information | Unclear if the endpoints were selected a priori. (Registered under IRCT20200324046850N2 but contents were edited on 2020-05-01). |

Salazar et al. “Treatment of COVID-19 patients with convalescent plasma reveals a signal of significantly decreased mortality”.

ROBINS-I

| Bias | Author’s judgment | Support for judgment |
| --- | --- | --- |
| **Pre-intervention domains** | | |
| Bias due to confounding  (Confounding) | Critical risk | Controls were matched to cases for various confounders using propensity scores, and Cox proportional hazards model is used to determine associations with mortality. However, even after matching significant difference was noted in the usage rate of prednisone, hydroxychloroquine, ribavirin, and tocilizumab. |
| Bias in selection of participants into the study  (Selection bias) | Low risk | All eligible patients were included. Patients were divided into subgroups based on timing of transfusion, and thus risk of selection bias is low. |
| **At-intervention domain** | | |
| Bias in classification of interventions  (Information bias) | Low risk | Intervention status is well-defined and defined solely on information collected at time of intervention. |
| **Post-intervention domains** | | |
| Bias due to deviations from intended interventions  (Confounding) | Moderate risk | Plasma transfusions may have led to deviations that could have affected the clinical outcome; however, they are mostly considered to reflect usual clinical practice. |
| Bias due to missing data  (Selection bias) | Serious risk | This is an interim analysis, and of the 2724 patients evaluated, 1642 patients were not included in this analysis (because they have not yet reached their 28-day outcome). |
| Bias in measurement of the outcome  (Information bias) | Low risk | Main outcome measure of mortality is unlikely to be affected by knowledge of intervention. |
| Bias in selection of reported result (Reporting bias) | Low risk | Although it is unclear whether the authors selected the main outcome measure a priori, mortality is the most important outcome for patients with severe disease; risk of selective reporting bias is deemed low. |

Klopfenstein et al. “Impact of tocilizumab on mortality and/or invasive mechanical ventilation requirement in a cohort of 206 COVID-19 patients”.

ROBINS-I

| Bias | Author’s judgment | Support for judgment |
| --- | --- | --- |
| **Pre-intervention domains** | | |
| Bias due to confounding  (Confounding) | Moderate risk | No adjustment methods for confounders are used. Baseline characteristics are established to be similar for the most part. |
| Bias in selection of participants into the study  (Selection bias) | Moderate risk | All eligible patients were included. Start of follow-up and start of intervention did not necessarily coincide for all patients, and average time from admission to tocilizumab administration was 7 days. Patients who received tocilizumab less than 24 hours before intubation or death were excluded. |
| **At-intervention domain** | | |
| Bias in classification of interventions  (Information bias) | Low risk | Intervention status is well-defined and defined solely on information collected at time of intervention. |
| **Post-intervention domains** | | |
| Bias due to deviations from intended interventions  (Confounding) | Low risk | Deviations likely reflect usual clinical practice. |
| Bias due to missing data  (Selection bias) | Low risk | Data were complete. |
| Bias in measurement of the outcome  (Information bias) | Low risk | The main endpoint of intubation and mortality are deemed at low risk of being influenced by the assessors’ knowledge of intervention. |
| Bias in selection of reported result (Reporting bias) | No information | The primary endpoint was a composite measure of intubation and death. It is unclear whether this outcome was selected a priori, and results were not significant for mortality alone. |

Li et al. “Corticosteroid prevents COVID-19 progression within its therapeutic window: a multicentre, proof-of-concept, observational study”.

ROBINS-I

| Bias | Author’s judgment | Support for judgment |
| --- | --- | --- |
| **Pre-intervention domains** | | |
| Bias due to confounding  (Confounding) | Low risk | Multivariable logistic analysis was used to find independent factors associated with outcome. |
| Bias in selection of participants into the study  (Selection bias) | Low risk | All eligible patients were enrolled. Start of intervention was determined based on radiographic and laboratory measures signaling state of excessive inflammation; start of intervention and start of follow-up were thus considered to coincide. |
| **At-intervention domain** | | |
| Bias in classification of interventions  (Information bias) | Low risk | Intervention status is well-defined and defined solely on information collected at time of intervention. |
| **Post-intervention domains** | | |
| Bias due to deviations from intended interventions  (Confounding) | Low risk | Deviations likely reflect usual clinical practice. |
| Bias due to missing data  (Selection bias) | Low risk | Data were complete. |
| Bias in measurement of the outcome  (Information bias) | Low risk | The primary endpoint of invasive mechanical ventilation is deemed at low risk of being influenced by the outcome assessors’ knowledge of intervention. |
| Bias in selection of reported result (Reporting bias) | No information | Unclear if the primary endpoint of invasive mechanical ventilation was specified a priori. |

Mitjà et al, “A Cluster-Randomized Trial of Hydroxychloroquine as Prevention of Covid-19 Transmission and Disease”

RoB 2 tool

| Bias | Author’s judgement | Support for judgement |
| --- | --- | --- |
| Bias arising from the randomization process | Low risk | Based on the statement that random number sequence generation was conducted free from the potential influence of physicians, allocation sequence is assumed to be concealed. There are no baseline imbalances to suggest a problem. |
| Bias due to deviations from intended interventions | Low risk | Participants and personnel are aware of interventions but non protocol interventions were balanced and there were no failures in implementation affecting outcome. |
| Bias due to missing outcome data | Low risk | Data were complete. |
| Bias in measurement of the outcome | High risk | Open label study, assessment of clinical outcome including symptoms maybe subjective which may influence the assessment. |
| Bias in selection of the reported result | Some concerns | No information as to whether the primary outcome measure was selected a priori. |

Lopes et al, “Beneficial effects of colchicine for moderate to severe COVID-19: an interim analysis of a randomized, double-blinded, placebo controlled clinical trial”.

RoB2 tool

| Bias | Author’s judgement | Support for judgement |
| --- | --- | --- |
| Bias arising from the randomization process | Low risk | Randomization was conducted through using online tool at randomization.org; and allocation sequence was adequately concealed. No baseline imbalances to suggest a problem. |
| Bias due to deviations from intended interventions | Low risk | double blind, placebo controlled clinical trial |
| Bias due to missing outcome data | Low risk | Data were reasonably complete. |
| Bias in measurement of the outcome | Low risk | double blind, placebo controlled clinical trial |
| Bias in selection of the reported result | Some concerns | No information provided |

Chen et al, “A Multicenter, randomized, open-label, controlled trial to evaluate the efficacy and tolerability of hydroxychloroquine and a retrospective study in adult patients with mild to moderate Coronavirus disease 2019 (COVID-19)”

RoB2 tool

| Bias | Author’s judgement | Support for judgement |
| --- | --- | --- |
| Bias arising from the randomization process | Low risk | Randomization was conducted through using interactive web response system. and allocation sequence was adequately concealed. No baseline imbalances to suggest a problem. |
| Bias due to deviations from intended interventions | Low risk | Open label study, but deviations due to trial context unlikely. |
| Bias due to missing outcome data | Low risk | Data were reasonably complete. |
| Bias in measurement of the outcome | Some concerns | Open label trial, but it is not likely that assessment have been influenced by knowledge of intervention. |
| Bias in selection of the reported result | Some concerns | No information provided. |

Majmundar et al. “Efficacy of corticosteroids in non-intensive care unit patients with COVID-10 pneumonia from the New York Metropolitan region.”

ROBINS-I

| Bias | Author’s judgment | Support for judgment |
| --- | --- | --- |
| **Pre-intervention domains** | | |
| Bias due to confounding  (Confounding) | Low risk | Cox regression used to adjust for confounders. |
| Bias in selection of participants into the study  (Selection bias) | Low risk | All eligible patients were included. Start of intervention and start of follow-up were considered to coincide as index date for patients who received corticosteroids was marked as the day corticosteroid was started. |
| **At-intervention domain** | | |
| Bias in classification of interventions  (Information bias) | Low risk | Intervention status is well-defined and defined solely on information collected at time of intervention. |
| **Post-intervention domains** | | |
| Bias due to deviations from intended interventions  (Confounding) | Low risk | Deviations likely reflect usual clinical practice. |
| Bias due to missing data  (Selection bias) | Low risk | Although a portion of the data regarding inflammatory markers are missing, this is not deemed to confer a significant degree of risk of bias. |
| Bias in measurement of the outcome  (Information bias) | Moderate risk | Composite endpoint of ICU transfer, intubation, and mortality was used. ICU transfer and intubation may be affected by knowledge of intervention of the caregivers. |
| Bias in selection of reported result (Reporting bias) | No information | No information as to whether the composite endpoint was selected a priori. |

Corral-Gudino et al, “GLUCOCOVID: A controlled trial of methylprednisolone in adults hospitalized

with COVID-19 pneumonia”

RoB2 tool

| Bias | Author’s judgement | Support for judgement |
| --- | --- | --- |
| Bias arising from the randomization process | Some concerns | Partially randomized trial, but randomization method was not provided in the script, however baseline imbalance does not suggest a problem |
| Bias due to deviations from intended interventions | Low risk | open label study, but deviations due to trial context unlikely. |
| Bias due to missing outcome data | Low risk | Data were reasonably complete. |
| Bias in measurement of the outcome | Low risk | Open label trial, but assessment could not have been influenced by knowledge of intervention since the describe endpoint were relatively objective such as mortality, and ICU admissions etc. |
| Bias in selection of the reported result | Some concerns | No information provided |

Sbidian et al. “Hydroxychloroquine with or without azithromycin and in-hospital mortality or discharge in patients hospitalized for COVID-19 infection: a cohort study of 4,642 patients in France”.

ROBINS-I

| Bias | Author’s judgment | Support for judgment |
| --- | --- | --- |
| **Pre-intervention domains** | | |
| Bias due to confounding  (Confounding) | Low risk | Augmented inverse probability of treatment weighted estimates of treatment effect was used to account for confounding. Cox regression used to derive doubly robust estimation. Several sensitivity analyses performed. |
| Bias in selection of participants into the study  (Selection bias) | Moderate risk | All eligible patients were included. Start of intervention and start of follow-up do not necessarily coincide for all patients. |
| **At-intervention domain** | | |
| Bias in classification of interventions  (Information bias) | Low risk | Intervention status is well-defined and defined solely on information collected at time of intervention. |
| **Post-intervention domains** | | |
| Bias due to deviations from intended interventions  (Confounding) | Low risk | Deviations likely reflect usual clinical practice. |
| Bias due to missing data  (Selection bias) | Low risk | Multiple imputation methods used to handle missing data. |
| Bias in measurement of the outcome  (Information bias) | Low risk | Main outcome measure of mortality (28-day) is unlikely to be affected by knowledge of intervention. |
| Bias in selection of reported result (Reporting bias) | Low risk | Although it is unclear whether the authors selected the main outcome measure a priori, mortality (28-day) is the most important outcome for patients with severe disease; risk of selective reporting bias is deemed low. |

Rajter et al. “ICON (Ivermectin in COvid Nineteen) study: Use of ivermectin is associated with lower mortality in hospitalized patients with COVID19”.

ROBINS-I

| Bias | Author’s judgment | Support for judgment |
| --- | --- | --- |
| **Pre-intervention domains** | | |
| Bias due to confounding  (Confounding) | Low risk | Multivariate analysis using stepwise binary logistic regression was used to adjust for confounders. Cox regression used to determine effect of ivermectin. |
| Bias in selection of participants into the study  (Selection bias) | Moderate risk | All eligible patients were included. Start of follow-up and start of intervention did not necessarily coincide for all patients (especially because more of the control group was enrolled earlier). |
| **At-intervention domain** | | |
| Bias in classification of interventions  (Information bias) | Low risk | Intervention status is well-defined and defined solely on information collected at time of intervention. |
| **Post-intervention domains** | | |
| Bias due to deviations from intended interventions  (Confounding) | Low risk | Deviations are likely to reflect usual clinical practice. |
| Bias due to missing data  (Selection bias) | Low risk | Data were reasonably complete. |
| Bias in measurement of the outcome  (Information bias) | Low risk | Main outcome measure of mortality (in-hospital) is unlikely to be affected by knowledge of intervention. |
| Bias in selection of reported result (Reporting bias) | Low risk | Although it is unclear whether the authors selected the main outcome measure a priori, mortality (in-hospital) is the most important outcome for patients with severe disease; risk of selective reporting bias is deemed low. |

Gorial et al. “Effectiveness of ivermectin as add-on therapy in COVID-19 management (pilot trial)”.

ROBINS-I

| Bias | Author’s judgment | Support for judgment |
| --- | --- | --- |
| **Pre-intervention domains** | | |
| Bias due to confounding  (Confounding) | Low risk | Controls were matched to cases for age, gender, clinical features, and comorbidities. |
| Bias in selection of participants into the study  (Selection bias) | Low risk | Consecutive patients eligible for the study were included. Start of intervention and start of follow-up coincided as ivermectin was administered on the admission day. |
| **At-intervention domain** | | |
| Bias in classification of interventions  (Information bias) | Low risk | Intervention status is well-defined and defined solely on information collected at time of intervention. |
| **Post-intervention domains** | | |
| Bias due to deviations from intended interventions  (Confounding) | Low risk | Deviations from intended intervention likely reflect usual clinical practice. |
| Bias due to missing data  (Selection bias) | Low risk | Data were complete. |
| Bias in measurement of the outcome  (Information bias) | Moderate risk | Primary outcome was resolution of symptoms and 2 consecutive negative PCR test from nasopharyngeal swabs. Symptoms are subjective and may be biased due to physicians’ knowledge of intervention while PCR tests are at less risk of bias. |
| Bias in selection of reported result (Reporting bias) | No information | No information as to whether the presented endpoints were chosen a priori. However, the study claims the beneficial effect of ivermectin even though the primary endpoint does not appear to be different between the two treatment arms. Risk of bias is deemed moderate within this NMA because only the data presented in the article is relevant to our analysis; however, this study’s claim that addition of ivermectin to hydroxychloroquine and azithromycin is beneficial should be interpreted with caution as bias has not been ruled out. |

Tat et al. “Adjunctive corticosteroids for COVID-19: a retrospective cohort study”.

ROBINS-I

| Bias | Author’s judgment | Support for judgment |
| --- | --- | --- |
| **Pre-intervention domains** | | |
| Bias due to confounding  (Confounding) | Low risk | Entropy balancing used to weigh the covariates. Weighted and adjusted Cox regression used to assess effect. |
| Bias in selection of participants into the study  (Selection bias) | Low risk | All eligible patients were included. Start of follow-up and start of intervention did not necessarily coincide for all patients. |
| **At-intervention domain** | | |
| Bias in classification of interventions  (Information bias) | Low risk | Intervention status is well-defined and defined solely on information collected at time of intervention. |
| **Post-intervention domains** | | |
| Bias due to deviations from intended interventions  (Confounding) | Low risk | Deviations from intended interventions likely reflect usual clinical practice. |
| Bias due to missing data  (Selection bias) | Low risk | Missing data were limited to certain lab values, and this was deemed not significant. |
| Bias in measurement of the outcome  (Information bias) | Moderate risk | Composite outcome of clinical progression, invasive mechanical ventilation, or death was used. It may be affected by the assessors’ knowledge of intervention. |
| Bias in selection of reported result (Reporting bias) | No information | No information as to whether the presented outcomes were selected a priori. |

Kamran et al, “Clearing the fog: Is HCQ effective in reducing COVID-19 progression: A randomized controlled trial”

RoB2 tool

| Bias | Author’s judgement | Support for judgement |
| --- | --- | --- |
| Bias arising from the randomization process | Low risk | Randomization were implemented by an independent statistician who was not involved in data analysis. And random number were generated by the computation. |
| Bias due to deviations from intended interventions | Low risk | Open label study, but no deviation arose because of the trial context |
| Bias due to missing outcome data | Low risk | Data were reasonably complete. |
| Bias in measurement of the outcome | Some concerns | Open label trial but measurement outcome includes relatively objective body temperature, 10-step walk test, derangement of basic lab parameters etc, thus, less likely that assessment was influenced by knowledge of intervention |
| Bias in selection of the reported result | Some concerns | No information provided |

Gharbharan et al. “Convalescent Plasma for COVID-19. A randomized clinical trial”.

RoB2 tool

| Bias | Author’s judgement | Support for judgement |
| --- | --- | --- |
| Bias arising from the randomization process | Some concerns | Allocation process was not described, but there are no baseline imbalances suggest a problem. |
| Bias due to deviations from intended interventions | Low risk | Open label study, but no deviations were likely to arise due to the trial context |
| Bias due to missing outcome data | Low risk | Data were reasonably complete. |
| Bias in measurement of the outcome | Low risk | Open label trial but measurement outcome includes overall mortality which could not be manipulated by knowledge of assessor. |
| Bias in selection of the reported result | Low risk | Outcome measures were overall mortality until discharge from the hospital or a maximum of 60 days after admission whichever comes first were used as pre-specified. |

Albani et al. “Effect of corticosteroid treatment on 1376 hospitalized COVID-19 patients. A cohort study”.

ROBINS-I

| Bias | Author’s judgment | Support for judgment |
| --- | --- | --- |
| **Pre-intervention domains** | | |
| Bias due to confounding  (Confounding) | Low risk | Weight propensity score for treatment allocation was calculated using a multivariable model containing various potential confounders. |
| Bias in selection of participants into the study  (Selection bias) | Moderate risk | All eligible patients were included. Start of intervention and start of follow-up did not necessarily coincide for all patients. |
| **At-intervention domain** | | |
| Bias in classification of interventions  (Information bias) | Low risk | Intervention status is well-defined and defined solely on information collected at time of intervention. |
| **Post-intervention domains** | | |
| Bias due to deviations from intended interventions  (Confounding) | Low risk | Deviations from intended interventions likely reflect usual clinical practice. |
| Bias due to missing data  (Selection bias) | Low risk | Missing values were replaced by mean substitution. Outcome data were complete. |
| Bias in measurement of the outcome  (Information bias) | Low risk | Main outcome measure of mortality (in-hospital) is unlikely to be affected by knowledge of intervention. |
| Bias in selection of reported result (Reporting bias) | Low risk | Although it is unclear whether the authors selected the main outcome measure a priori, mortality (in-hospital) is the most important outcome for patients with severe disease; risk of selective reporting bias is deemed low. |

RECOVERY group, “Effect of Hydroxychloroquine in Hospitalized Patients with COVID-19: Preliminary results from a multi-centre, randomized, controlled trial.”

RoB2 tool

| Bias | Author’s judgement | Support for judgement |
| --- | --- | --- |
| Bias arising from the randomization process | Low risk | Web based simple randomization with allocation concealment was used. there were no significant baseline imbalances observed |
| Bias due to deviations from intended interventions | Low risk | Open label study, but no reported deviations from intended intervention. |
| Bias due to missing outcome data | Low risk | Data were complete |
| Bias in measurement of the outcome | Low risk | Open label study, but the assessment was objective so that it could not have influenced clinical decisions. |
| Bias in selection of the reported result | Low risk | No multiple primary outcome measurement and trial analyzed in accordance with a prespecified plan since there were no changes in outcome measurement and selection |

Martinez-Sanz et al. “Effects of tocilizumab on mortality in hospitalized patients with COVID-19: a multicenter cohort study”.

ROBINS-I

| Bias | Author’s judgment | Support for judgment |
| --- | --- | --- |
| **Pre-intervention domains** | | |
| Bias due to confounding  (Confounding) | Low risk | Inverse-probability weighting used to adjust for confounders. |
| Bias in selection of participants into the study  (Selection bias) | Moderate risk | All eligible patients were included. Start of intervention and start of follow-up did not necessarily coincide for all patients. |
| **At-intervention domain** | | |
| Bias in classification of interventions  (Information bias) | Low risk | Intervention status is well-defined and defined solely on information collected at time of intervention. |
| **Post-intervention domains** | | |
| Bias due to deviations from intended interventions  (Confounding) | Low risk | Deviations from intended interventions likely reflect usually clinical practice. |
| Bias due to missing data  (Selection bias) | Low risk | Characteristics of individuals not included due to missing data were presented in the supplementary materials and were similar to those of the included patients. |
| Bias in measurement of the outcome  (Information bias) | Low risk | Main outcome measure of mortality (rate per person-days) is unlikely to be affected by knowledge of intervention. |
| Bias in selection of reported result (Reporting bias) | Low risk | Although it is unclear whether the authors selected the main outcome measure a priori, mortality (rate per person-days) is the most important outcome for patients with severe disease; risk of selective reporting bias is deemed low. |

Chen et al, “Efficacy and safety of chloroquine or hydroxychloroquine in moderate type of COVID-19: a prospective open-label randomized controlled study”.

RoB2 tool

| Bias | Author’s judgement | Support for judgement |
| --- | --- | --- |
| Bias arising from the randomization process | Some Concerns | No information was revealed on concealment of allocation sequence, but no significant baseline imbalance was observed |
| Bias due to deviations from intended interventions | Some concerns | Open label trial, one subject discontinued chloroquine due to ALT flare in chloroquine group, and 3 patients with no symptoms were excluded in hydroxychloroquine group. |
| Bias due to missing outcome data | Low risk | Data were reasonably complete |
| Bias in measurement of the outcome | High risk | Time to clinical recovery was used for measurement of outcome, though contents of clinical recovery include body temperature as objective value, relief of symptoms is subjective. |
| Bias in selection of the reported result | Some concerns | Not enough information. |

Tsai et al. “Impact of tocilizumab administration on mortality in severe COVID-19”.

ROBINS-I

| Bias | Author’s judgment | Support for judgment |
| --- | --- | --- |
| **Pre-intervention domains** | | |
| Bias due to confounding  (Confounding) | Low risk | Propensity scores were used to match patients to treatment allocations. |
| Bias in selection of participants into the study  (Selection bias) | Moderate risk | Only patients with at least one ferritin measurement was required to be eligible for the study. Start of intervention and start of follow-up did not necessarily coincide for all patients as administration of tocilizumab was dependent on the physician. |
| **At-intervention domain** | | |
| Bias in classification of interventions  (Information bias) | Low risk | Intervention status is well-defined and defined solely on information collected at time of intervention. |
| **Post-intervention domains** | | |
| Bias due to deviations from intended interventions  (Confounding) | Low risk | Deviation from the intended intervention likely reflects usual clinical practice. |
| Bias due to missing data  (Selection bias) | Low risk | Multiple imputation was used to handle missing data. Data on outcomes were deemed complete. |
| Bias in measurement of the outcome  (Information bias) | Low risk | Main outcome measure of mortality (in-hospital) is unlikely to be affected by knowledge of intervention. |
| Bias in selection of reported result (Reporting bias) | Low risk | Although it is unclear whether the authors selected the main outcome measure a priori, mortality (in-hospital) is the most important outcome for patients with severe disease; risk of selective reporting bias is deemed low. |

Sakoulas et al, “Intravenous Immunoglobulin (IVIG) Significantly Reduces Respiratory Morbidity in COVID-19 Pneumonia: A Prospective Randomized Trial”

RoB2 tool

| Bias | Author’s judgement | Support for judgement |
| --- | --- | --- |
| Bias arising from the randomization process | Low risk | Allocation was adequately concealed, and there were no baseline imbalances suggest a problem. |
| Bias due to deviations from intended interventions | Some concerns | Open label trial, both of group were able to receive remdesivir but deviations balanced between groups. |
| Bias due to missing outcome data | Low risk | Data were reasonably complete |
| Bias in measurement of the outcome | Low risk | Open label study, but outcome assessment was mechanical ventilation, and this could not have been influenced by knowledge of intervention. |
| Bias in selection of the reported result | Some concerns | Insufficient information |

Salton et al. “Prolonged low-dose methylprednisolone in patients with severe COVID-19 pneumonia”.

ROBINS-I

| Bias | Author’s judgment | Support for judgment |
| --- | --- | --- |
| **Pre-intervention domains** | | |
| Bias due to confounding  (Confounding) | Low risk | Multivariable Cox model used to estimate the hazard ratio for endpoints while accounting for various confounders. |
| Bias in selection of participants into the study  (Selection bias) | Moderate risk | Consecutive patients meeting eligibility were all included. Start of follow-up and intervention did not necessarily coincide as the treating team was responsible for the decision to use methylprednisolone. |
| **At-intervention domain** | | |
| Bias in classification of interventions  (Information bias) | Low risk | Intervention status is well-defined and defined solely on information collected at time of intervention. |
| **Post-intervention domains** | | |
| Bias due to deviations from intended interventions  (Confounding) | Low risk | Deviation from the intended intervention likely reflects usual clinical practice. |
| Bias due to missing data  (Selection bias) | Low risk | Data were complete |
| Bias in measurement of the outcome  (Information bias) | Moderate risk | Composite primary endpoint of ICU referral, intubation, or death within 28 days was used. This endpoint may be affected by the physicians’ knowledge of intervention. |
| Bias in selection of reported result (Reporting bias) | No information | No information as to whether the primary outcome was selected a priori. |

Lofgren et al, “Safety of Hydroxychloroquine among Outpatient Clinical Trial Participants for COVID-19”

RoB2 tool

| Bias | Author’s judgement | Support for judgement |
| --- | --- | --- |
| Bias arising from the randomization process | Some concerns | Exact method of randomization and allocation were not specified. Baseline was not compared between two groups in the script. |
| Bias due to deviations from intended interventions | Low risk | According to the protocol, masking was quadrupled (participants, care provider, investigator, outcome assessor) |
| Bias due to missing outcome data | Low risk | Data were complete. |
| Bias in measurement of the outcome | Low risk | According to the protocol, masking was quadrupled (participants, care provider, investigator, outcome assessor) so that outcome assessors was not able to be aware of intervention received. |
| Bias in selection of the reported result | Some concerns | The primary outcome was changed to incidence of COVID-10 among those who are asymptomatic at baseline. |

Díaz et al. “Use of a humanized anti-CD6 monoclonal antibody (itolizumab) in elderly patients with moderate COVID-19”.

ROBINS-I

| Bias | Author’s judgment | Support for judgment |
| --- | --- | --- |
| **Pre-intervention domains** | | |
| Bias due to confounding  (Confounding) | Low risk | Controls were matched to cases for age, comorbidities, and severity of disease. |
| Bias in selection of participants into the study  (Selection bias) | Moderate risk | Nineteen patients in a nursing home were enrolled to the intervention arm. Start of follow-up and start of intervention coincided for all patients. |
| **At-intervention domain** | | |
| Bias in classification of interventions  (Information bias) | Low risk | Intervention status is well-defined and defined solely on information collected at time of intervention. |
| **Post-intervention domains** | | |
| Bias due to deviations from intended interventions  (Confounding) | Moderate risk | As cases were taken from a single nursing home while controls were taken from a database, presumably consisting of patients treated under different settings, the risk of confounding due to deviation from intended interventions cannot be ignored. |
| Bias due to missing data  (Selection bias) | Low risk | Data were complete. |
| Bias in measurement of the outcome  (Information bias) | Moderate risk | No clear primary endpoint is presented. Among the presented endpoints, transfer to the ICU may be affected by physicians’ knowledge of intervention. |
| Bias in selection of reported result (Reporting bias) | Moderate risk | No information as to whether the presented outcomes were selected a priori. Use of multiple endpoints raises concern for multiplicity. |

1. **NOS and Jadad quality assessment of included studies**

| **Table S1.** Quality rating for case-control/cohort studies using the Newcastle-Ottawa quality assessment scale | | | | | | | | | | | |
| --- | --- | --- | --- | --- | --- | --- | --- | --- | --- | --- | --- |
|  | **Selection** |  |  |  |  | **Comparability** |  | **Exposure /Outcome** |  |  | **Total Score** |
| Case-control studies/  Cohort studies  **Authors** | Adequacy of case definition/  Representativeness of exposed cohort | Representativeness of cases/  Selection of non-exposed cohort | Selection of controls/  Ascertainment of exposure | Definition of controls/  Outcome not present at the start of study |  | Comparability of cases and controls/  Control for age, sex, comorbidities, severity measures, etc. |  | Assessment of exposures/  Assessment of outcomes | Same method of ascertainment for cases and controls/  Length of follow-up | Non-response rate/  Adequacy of follow-up |  |
| 1 Rosenberg et al. | * | * | * | * |  | ** |  | * | * | * | 9 |
| 2 Geleris et al. | * | * | * | * |  | ** |  | * | * | * | 9 |
| 3 Cai et al | * | * | * | * |  | ** |  | * | * | * | 9 |
| 4 Bessiere et al | * | * | * | * |  |  |  | * | * | * | 7 |
| 5 Mercuro et al | * | * | * | * |  |  |  | * | * | * | 7 |
| 6 Mahevas et al | * | * | * | * |  | ** |  | * | * | * | 9 |
| 7 Lu et al | * | * | * | * |  | ** |  | * | * | * | 9 |
| 8 Chen X. et al (Lancet ID) | * | * | * | * |  | ** |  | * | * | * | 9 |
| 9 Shao et al | * | * | * | * |  | ** |  | * | * | * | 9 |
| 10 Deng et al | * | * | * | * |  |  |  | * | * | * | 7 |
| 11 Wang et al (Nature) | * | * | * | * |  | ** |  | * | * | * | 8 |
| 12 Saleh et al | * | * | * | * |  |  |  | * | * | * | 7 |
| 13 Ramireddy et al | * | * | * | * |  |  |  | * | * | * | 7 |
| 14 Freedberg et al | * | * | * | * |  | ** |  | * | * | * | 9 |
| 15 Magagnoli et al | * | * | * | * |  | ** |  | * | * | * | 9 |
| 16 Carlucci et al | * | * | * | * |  |  |  | * | * | * | 7 |
| 17 Lan et al | * | * | * | * |  |  |  | * | * | * | 7 |
| 18 Huang M. et al | * |  | * | * |  | * |  | * | * | * | 7 |
| 19 Wu et al | * | * | * | * |  | ** |  | * | * | * | 9 |
| 20 Kim M. et al | * | * | * | * |  |  |  | * | * | * | 7 |
| 21 Shi et al | * | * | * | * |  |  |  | * | * | * | 7 |
| 22 Feng et al | * | * | * | * |  | ** |  | * | * | * | 9 |
| 23 Bian et al |  | * | * | * |  |  |  | * | * | * | 6 |
| 24 Singh et al | * | * | * | * |  | ** |  | * | * | * | 9 |
| 25 Fernandez et al | * | * | * | * |  | ** |  | * | * | * | 9 |
| 26 Campochiaro et al | * | * | * | * |  | ** |  | * | * | * | 9 |
| 27 Moreno-Garcia et al | * | * | * | * |  | ** |  | * | * | * | 9 |
| 28 Somers et al | * | * | * | * |  | ** |  | * | * | * | 9 |
| 29 Rossi et al | * | * | * | * |  | ** |  | * | * | * | 9 |
| 30 Huet et al | * | * | * | * |  | ** |  | * | * | * | 9 |
| 31 Capral et al | * | * | * | * |  | ** |  | * | * | * | 9 |
| 32 Yuan et al | * | * | * |  |  | ** |  |  | * | * | 7 |
| 33 Lecronier et al | * | * | * | * |  |  |  | * | * | * | 7 |
| 34 Nelson et al | * |  | * | * |  | ** |  | * | * | * | 8 |
| 35 Olender et al | * | * | * | * |  | ** |  | * | * | * | 9 |
| 36 Arshad et al | * | * | * | * |  | ** |  | * | * | * | 9 |
| 37 Biran et al | * | * | * | * |  | ** |  | * | * | * | 9 |
| 38 Ip et al | * | * | * | * |  | ** |  | * | * | * | 9 |
| 39 Ma et al | * | * | * | * |  |  |  | * | * | * | 7 |
| 40 Hu et al | * | * | * | * |  | ** |  | * | * | * | 9 |
| 41 Abolghasemi et al | * |  | * | * |  | * |  | * | * | * | 7 |
| 42 Cantini et al | * | * | * | * |  |  |  | * | * | * | 7 |
| 43 Ramiro et al | * | * | * | * |  | ** |  | * | * | * | 9 |
| 44 Karolyi et al | * | * | * | * |  |  |  | * | * | * | 7 |
| 45 Lian et al | * | * | * | * |  | * |  | * | * | * | 8 |
| 46 Hao et al | * | * | * | * |  | ** |  | * | * | * | 9 |
| 47 Canziani et al |  |  | * | * |  | ** |  | * | * | * | 7 |
| 48 Fang et al | * | * | * | * |  | * |  | * | * | * | 8 |
| 49 Kim J. et al | * | * | * | * |  | ** |  | * | * | * | 9 |
| 50 Della-Torre et al | * | * | * | * |  | ** |  | * | * | * | 9 |
| 51 Herrero et al | * | * | * | * |  | * |  | * | * | * | 8 |
| 52 Wang et al (Cell Host & Microbe) | * | * | * | * |  | ** |  | * | * | * | 9 |
| 53 Tong et al |  | * | * | * |  | ** |  | * | * | * | 8 |
| 54 Rossotti et al | * | * | * | * |  | ** |  | * | * | * | 9 |
| 55 De Luca et al | * | * | * | * |  | ** |  | * | * | * | 9 |
| 56 Guaraldi et al | * | * | * | * |  | ** |  | * | * | * | 9 |
| 57 Eslami et al | * | * | * | * |  | ** |  | * | * | * | 9 |
| 58 Salazar et al | * | * | * | * |  | ** |  | * | * | * | 9 |
| 59 Klopfenstein et al | * | * | * | * |  |  |  | * | * | * | 7 |
| 60 Li et al | * | * | * | * |  | ** |  | * | * | * | 9 |
| 61 Majmundar et al | * | * | * | * |  | ** |  | * | * | * | 9 |
| 62 Sbidian et al | * | * | * | * |  | ** |  | * | * | * | 9 |
| 63 Rajter et al | * | * | * | * |  | ** |  | * | * | * | 9 |
| 64 Gorial et al | * | * | * | * |  | * |  | * | * | * | 8 |
| 65 Tat et al | * | * | * | * |  | ** |  | * | * | * | 9 |
| 66 Albani et al | * | * | * | * |  | ** |  | * | * | * | 9 |
| 67 Martinez-Sanz et al | * | * | * | * |  | ** |  | * | * | * | 9 |
| 68 Tsai et al | * | * | * | * |  | ** |  | * | * | * | 9 |
| 69 Salton et al | * | * | * | * |  | ** |  | * | * | * | 9 |
| 70 Díaz et al |  |  | * | * |  | * |  | * | * | * | 6 |

| **Table S2.** Quality rating for randomized studies using the Jadad assessment scale | | | | | | | | | |
| --- | --- | --- | --- | --- | --- | --- | --- | --- | --- |
| **Author** | **Randomization** | | |  | **Withdrawals** |  | **Blinding** |  | **Total Score** |
|  | Described as randomized | | Randomization method described and appropriate |  | Description of withdrawals |  | Described as double-blind | Double-blinding method described and appropriate |  |
| 1 Borba et al. | * | | * |  | * |  | * | * | 5 |
| 2 Cao B. et al | * | * | |  | * |  |  |  | 3 |
| 3 Hung et al | * | * | |  | * |  |  |  | 3 |
| 4 Wang et al (Lancet) | * | * | |  | * |  | * | * | 5 |
| 5 Li Y. et al | * | * | |  | * |  | * |  | 4 |
| 6 Tang et al | * | * | |  | * |  |  |  | 3 |
| 7 Beigel et al | * | * | |  | * |  | * | * | 5 |
| 8 Goldman et al | * | * | |  | * |  |  |  | 3 |
| 9 Zheng et al | * | * | |  | * |  |  |  | 3 |
| 10 Zhong et al | * | * | |  | * |  |  |  | 3 |
| 11 Lou et al | * |  | |  | * |  |  |  | 2 |
| 12 Chen Z. et al (medRxiv) | * |  | |  | * |  | * |  | 2 |
| 13 Boulware et al | * | * | |  | * |  | * | * | 5 |
| 14 Li L. et al | * | * | |  | * |  | * | * | 5 |
| 15 Davoudi-Monfared et al | * |  | |  | * |  |  |  | 2 |
| 16 Cao Y. et al | * | * | |  | * |  | * | * | 5 |
| 17 Skipper et al | * | * | |  | * |  | * | * | 5 |
| 18 Miller et al | * |  | |  | * |  |  |  | 2 |
| 19 Ivashchenko et al | * |  | |  | * |  |  |  | 2 |
| 20 Mijtà et al | * | * | |  | * |  |  |  | 3 |
| 21 Jeronimo et al | * | * | |  | * |  | * | * | 5 |
| 22 RECOVERY group (dexamethasone) | * | * | |  | * |  |  |  | 3 |
| 23 Cavalcanti et al | * | * | |  | * |  |  |  | 3 |
| 24 Chen J. et al | * |  | |  | * |  |  |  | 2 |
| 25 Wang et al (CJIM) | * | * | |  | * |  |  |  | 3 |
| 26 Huang et al | * | * | |  | * |  |  |  | 3 |
| 27 Spinner et al | * | * | |  | * |  |  |  | 3 |
| 28 Sadeghi et al | * | * | |  | * |  |  |  | 3 |
| 29 Kasgari et al | * | * | |  | * |  |  |  | 3 |
| 30 Deftereos et al | * | * | |  | * |  |  |  | 3 |
| 31 Mitjà et al | * | * | |  | * |  |  |  | 3 |
| 32 Lopes et al | * | * | |  | * |  | * | * | 5 |
| 33 Chen C-P. et al | * | * | |  | * |  |  |  | 3 |
| 34 Corral-Gudino et al | * |  | |  | * |  |  |  | 2 |
| 35 Kamran et al | * | * | |  | * |  |  |  | 3 |
| 36 Gharbharan et al | * |  | |  |  |  |  |  | 1 |
| 37 RECOVERY group (hydroxychloroquine) | * | * | |  | * |  |  |  | 3 |
| 38 Chen L. et al | * |  | |  | * |  |  |  | 2 |
| 39 Sakoulas et al | * | * | |  | * |  |  |  | 3 |
| 40 Lofgren et al | * |  | |  | * |  | * |  | 3 |
