# Supplementary material for: Comparative efficacy and safety of pharmacological interventions for the treatment of COVID-19: A systematic review and network meta-analysis
Source: PLoS Med. 2020 Dec 30;17(12):e1003501. doi: 10.1371/journal.pmed.1003501 (PMC7794037; doi:10.1371/journal.pmed.1003501)

Efficacy outcomes: efficacy

- 1. **Mortality in non-ICU patients at admission (moderate to severe)**
  2. **Mortality in ICU patients (critically ill)**
  3. **Progression to severe course (progress to severe pneumonia or admission to ICU)**
  4. **Viral clearance rate in 7-14 days**
  5. **Time to viral clearance**

1. **Mortality in non-ICU patients at admission (moderate to severe)**
   1. Network map


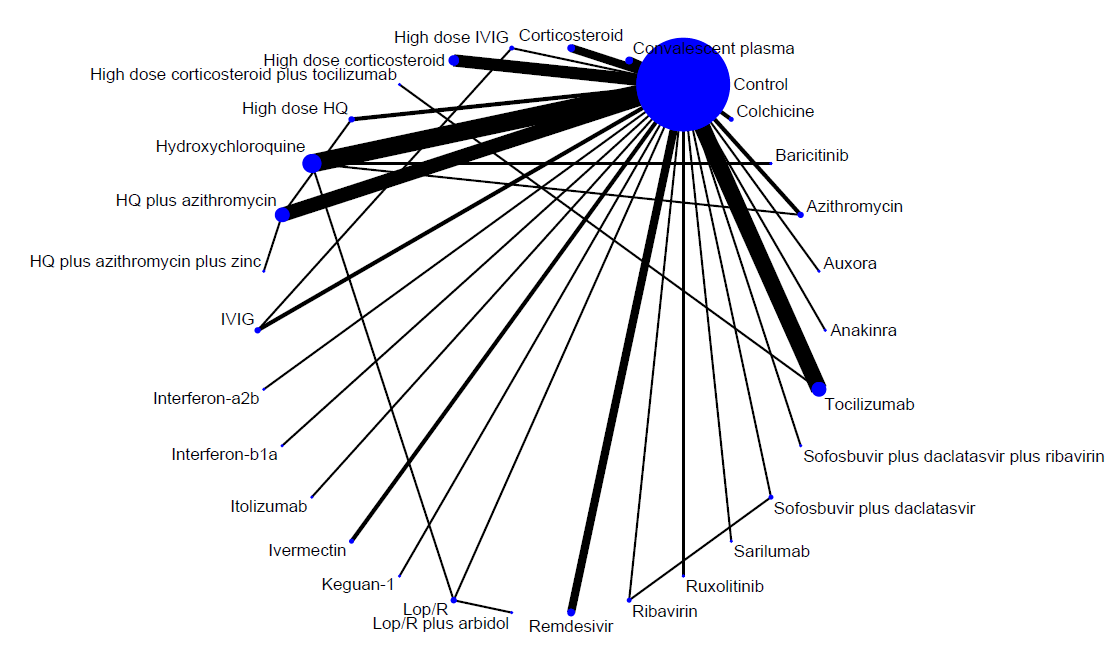


- 1. Forest plot


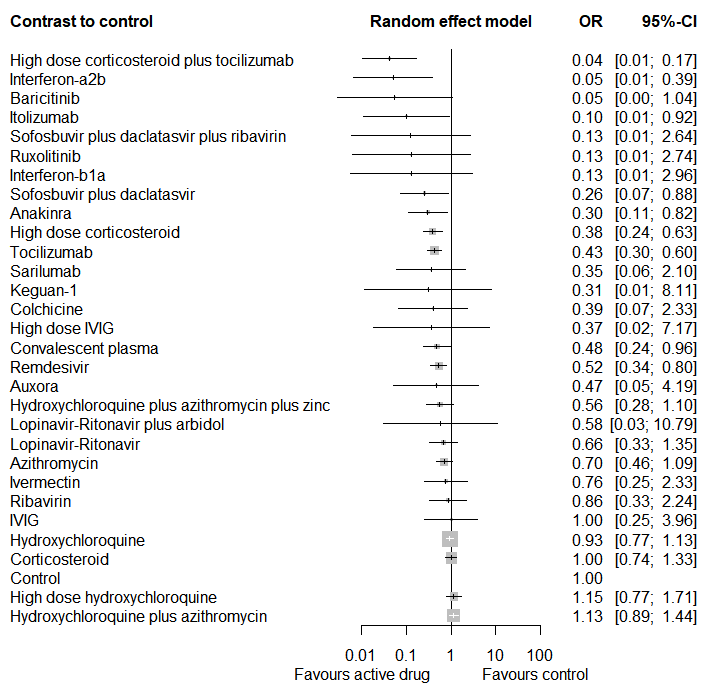


- 1. League table

| High dose corticosteroid plus tocilizumab | . | . | . | . | . | . | . | . | . | 0.10 [0.03 ; 0.38] | . | . | . | . | . | . | . | . | . | . | . | . | . | . | . | . | . | . | . |
| --- | --- | --- | --- | --- | --- | --- | --- | --- | --- | --- | --- | --- | --- | --- | --- | --- | --- | --- | --- | --- | --- | --- | --- | --- | --- | --- | --- | --- | --- |
| 0.84 [0.07 ; 10.00] | Interferon-a2b | . | . | . | . | . | . | . | . | . | . | . | . | . | . | . | . | . | . | . | . | . | . | . | . | . | 0.05 [0.01 ; 0.39] | . | . |
| 0.77 [0.03 ; 20.00] | 0.91 [0.03 ; 32.86] | Baricitinib | . | . | . | . | . | . | . | . | . | . | . | . | . | . | . | . | . | . | . | . | . | . | 0.06 [0.00 ; 1.11] | . | . | . | . |
| 0.42 [0.03 ; 5.82] | 0.50 [0.02 ; 10.24] | 0.55 [0.01 ; 21.95] | Itolizumab | . | . | . | . | . | . | . | . | . | . | . | . | . | . | . | . | . | . | . | . | . | . | . | 0.10 [0.01 ; 0.92] | . | . |
| 0.34 [0.01 ; 9.59] | 0.40 [0.01 ; 15.62] | 0.44 [0.01 ; 30.25] | 0.80 [0.02 ; 34.67] | Sofosbuvir plus daclatasvir plus ribavirin | . | . | . | . | . | . | . | . | . | . | . | . | . | . | . | . | . | . | . | . | . | . | 0.13 [0.01 ; 2.64] | . | . |
| 0.33 [0.01 ; 9.41] | 0.39 [0.01 ; 15.32] | 0.43 [0.01 ; 29.63] | 0.78 [0.02 ; 33.98] | 0.97 [0.01 ; 72.79] | Ruxolitinib | . | . | . | . | . | . | . | . | . | . | . | . | . | . | . | . | . | . | . | . | . | 0.13 [0.01 ; 2.74] | . | . |
| 0.33 [0.01 ; 10.10] | 0.39 [0.01 ; 16.34] | 0.43 [0.01 ; 31.35] | 0.78 [0.02 ; 36.20] | 0.97 [0.01 ; 76.94] | 1.00 [0.01 ; 79.66] | Interferon-b1a | . | . | . | . | . | . | . | . | . | . | . | . | . | . | . | . | . | . | . | . | 0.13 [0.01 ; 2.96] | . | . |
| 0.16 [0.03 ; 1.06] | 0.20 [0.02 ; 2.13] | 0.21 [0.01 ; 5.23] | 0.39 [0.03 ; 4.98] | 0.49 [0.02 ; 13.15] | 0.50 [0.02 ; 13.64] | 0.50 [0.02 ; 14.66] | Sofosbuvir plus daclatasvir | . | . | . | . | . | . | . | . | . | . | . | . | . | . | . | 0.12 [0.02 ; 0.65] | . | . | . | 0.56 [0.12 ; 2.70] | . | . |
| 0.14 [0.03 ; 0.78] | 0.17 [0.02 ; 1.62] | 0.18 [0.01 ; 4.10] | 0.33 [0.03 ; 3.82] | 0.42 [0.02 ; 10.33] | 0.43 [0.02 ; 10.72] | 0.43 [0.02 ; 11.54] | 0.85 [0.17 ; 4.18] | Anakinra | . | . | . | . | . | . | . | . | . | . | . | . | . | . | . | . | . | . | 0.30 [0.11 ; 0.82] | . | . |
| 0.11 [0.02 ; 0.48] | 0.13 [0.02 ; 1.06] | 0.14 [0.01 ; 2.82] | 0.26 [0.03 ; 2.53] | 0.33 [0.01 ; 7.13] | 0.33 [0.02 ; 7.40] | 0.33 [0.01 ; 7.99] | 0.66 [0.18 ; 2.51] | 0.78 [0.26 ; 2.38] | High dose corticosteroid | . | . | . | . | . | . | . | . | . | . | . | . | . | . | . | . | . | 0.38 [0.24 ; 0.63] | . | . |
| 0.10 [0.03 ; 0.38] | 0.12 [0.01 ; 0.93] | 0.13 [0.01 ; 2.50] | 0.23 [0.02 ; 2.23] | 0.29 [0.01 ; 6.32] | 0.30 [0.01 ; 6.56] | 0.30 [0.01 ; 7.09] | 0.60 [0.17 ; 2.16] | 0.70 [0.24 ; 2.04] | 0.90 [0.49 ; 1.65] | Tocilizumab | . | . | . | . | . | . | . | . | . | . | . | . | . | . | . | . | 0.43 [0.30 ; 0.60] | . | . |
| 0.12 [0.01 ; 1.14] | 0.14 [0.01 ; 2.12] | 0.15 [0.00 ; 4.84] | 0.28 [0.02 ; 4.88] | 0.35 [0.01 ; 12.08] | 0.36 [0.01 ; 12.52] | 0.36 [0.01 ; 13.39] | 0.72 [0.08 ; 6.30] | 0.85 [0.11 ; 6.54] | 1.09 [0.17 ; 6.90] | 1.20 [0.20 ; 7.39] | Sarilumab | . | . | . | . | . | . | . | . | . | . | . | . | . | . | . | 0.35 [0.06 ; 2.10] | . | . |
| 0.14 [0.00 ; 4.85] | 0.16 [0.00 ; 7.76] | 0.18 [0.00 ; 14.66] | 0.33 [0.01 ; 17.14] | 0.41 [0.00 ; 35.93] | 0.42 [0.00 ; 37.19] | 0.42 [0.00 ; 39.22] | 0.83 [0.03 ; 27.66] | 0.98 [0.03 ; 30.16] | 1.26 [0.05 ; 34.55] | 1.39 [0.05 ; 37.54] | 1.16 [0.03 ; 48.14] | Keguan-1 | . | . | . | . | . | . | . | . | . | . | . | . | . | . | 0.31 [0.01 ; 8.11] | . | . |
| 0.11 [0.01 ; 1.03] | 0.13 [0.01 ; 1.91] | 0.14 [0.00 ; 4.35] | 0.25 [0.01 ; 4.39] | 0.32 [0.01 ; 10.87] | 0.33 [0.01 ; 11.27] | 0.33 [0.01 ; 12.05] | 0.65 [0.07 ; 5.67] | 0.76 [0.10 ; 5.89] | 0.98 [0.15 ; 6.21] | 1.08 [0.18 ; 6.65] | 0.90 [0.07 ; 11.17] | 0.78 [0.02 ; 32.44] | Colchicine | . | . | . | . | . | . | . | . | . | . | . | . | . | 0.39 [0.07 ; 2.33] | . | . |
| 0.12 [0.00 ; 3.09] | 0.14 [0.00 ; 5.07] | 0.15 [0.00 ; 9.89] | 0.27 [0.01 ; 11.26] | 0.34 [0.00 ; 24.31] | 0.35 [0.00 ; 25.17] | 0.35 [0.00 ; 26.61] | 0.70 [0.03 ; 17.56] | 0.82 [0.04 ; 19.01] | 1.05 [0.05 ; 21.55] | 1.17 [0.06 ; 23.37] | 0.97 [0.03 ; 31.12] | 0.84 [0.01 ; 70.19] | 1.08 [0.03 ; 34.56] | High dose IVIG | . | . | . | . | . | . | . | . | . | 0.28 [0.01 ; 5.64] | . | . | 0.48 [0.02 ; 9.84] | . | . |
| 0.09 [0.02 ; 0.42] | 0.10 [0.01 ; 0.90] | 0.11 [0.01 ; 2.35] | 0.21 [0.02 ; 2.14] | 0.26 [0.01 ; 5.94] | 0.27 [0.01 ; 6.16] | 0.27 [0.01 ; 6.65] | 0.53 [0.13 ; 2.19] | 0.63 [0.19 ; 2.11] | 0.80 [0.34 ; 1.87] | 0.89 [0.41 ; 1.92] | 0.74 [0.11 ; 4.98] | 0.64 [0.02 ; 18.16] | 0.82 [0.12 ; 5.53] | 0.76 [0.04 ; 16.16] | Convalescent plasma | . | . | . | . | . | . | . | . | . | . | . | 0.48 [0.24 ; 0.96] | . | . |
| 0.08 [0.02 ; 0.35] | 0.10 [0.01 ; 0.77] | 0.10 [0.01 ; 2.05] | 0.19 [0.02 ; 1.84] | 0.24 [0.01 ; 5.20] | 0.25 [0.01 ; 5.39] | 0.25 [0.01 ; 5.82] | 0.49 [0.13 ; 1.80] | 0.57 [0.19 ; 1.70] | 0.74 [0.39 ; 1.41] | 0.81 [0.47 ; 1.41] | 0.68 [0.11 ; 4.22] | 0.59 [0.02 ; 15.94] | 0.75 [0.12 ; 4.68] | 0.70 [0.03 ; 14.13] | 0.92 [0.41 ; 2.06] | Remdesivir | . | . | . | . | . | . | . | . | . | . | 0.52 [0.34 ; 0.80] | . | . |
| 0.09 [0.01 ; 1.22] | 0.11 [0.01 ; 2.15] | 0.12 [0.00 ; 4.62] | 0.21 [0.01 ; 4.87] | 0.27 [0.01 ; 11.47] | 0.28 [0.01 ; 11.89] | 0.28 [0.01 ; 12.66] | 0.55 [0.04 ; 6.78] | 0.64 [0.06 ; 7.17] | 0.82 [0.09 ; 7.81] | 0.91 [0.10 ; 8.41] | 0.76 [0.04 ; 12.79] | 0.66 [0.01 ; 33.84] | 0.84 [0.05 ; 14.20] | 0.78 [0.02 ; 31.59] | 1.03 [0.10 ; 10.25] | 1.12 [0.12 ; 10.45] | Auxora | . | . | . | . | . | . | . | . | . | 0.47 [0.05 ; 4.19] | . | . |
| 0.08 [0.02 ; 0.36] | 0.09 [0.01 ; 0.77] | 0.10 [0.00 ; 2.01] | 0.18 [0.02 ; 1.83] | 0.22 [0.01 ; 5.09] | 0.23 [0.01 ; 5.28] | 0.23 [0.01 ; 5.70] | 0.46 [0.11 ; 1.87] | 0.54 [0.16 ; 1.80] | 0.69 [0.30 ; 1.59] | 0.76 [0.36 ; 1.63] | 0.63 [0.09 ; 4.26] | 0.55 [0.02 ; 15.56] | 0.70 [0.10 ; 4.73] | 0.65 [0.03 ; 13.85] | 0.86 [0.33 ; 2.26] | 0.94 [0.42 ; 2.08] | 0.84 [0.08 ; 8.30] | Hydroxychloroquine plus azithromycin plus zinc | . | . | . | . | . | . | . | . | . | . | 0.49 [0.26 ; 0.93] |
| 0.07 [0.00 ; 1.87] | 0.09 [0.00 ; 3.08] | 0.09 [0.00 ; 6.03] | 0.17 [0.00 ; 6.85] | 0.22 [0.00 ; 14.86] | 0.22 [0.00 ; 15.38] | 0.22 [0.00 ; 16.27] | 0.44 [0.02 ; 10.61] | 0.52 [0.02 ; 11.48] | 0.67 [0.03 ; 12.98] | 0.74 [0.04 ; 14.08] | 0.61 [0.02 ; 18.87] | 0.53 [0.01 ; 42.95] | 0.68 [0.02 ; 20.95] | 0.63 [0.01 ; 41.17] | 0.83 [0.04 ; 16.83] | 0.91 [0.05 ; 17.44] | 0.81 [0.02 ; 31.37] | 0.97 [0.05 ; 19.53] | Lopinavir-Ritonavir plus arbidol | 0.87 [0.05 ; 14.87] | . | . | . | . | . | . | . | . | . |
| 0.06 [0.01 ; 0.30] | 0.08 [0.01 ; 0.65] | 0.08 [0.00 ; 1.70] | 0.15 [0.01 ; 1.55] | 0.19 [0.01 ; 4.31] | 0.19 [0.01 ; 4.47] | 0.19 [0.01 ; 4.82] | 0.38 [0.09 ; 1.60] | 0.45 [0.13 ; 1.54] | 0.58 [0.24 ; 1.38] | 0.64 [0.29 ; 1.42] | 0.53 [0.08 ; 3.62] | 0.46 [0.02 ; 13.17] | 0.59 [0.09 ; 4.02] | 0.55 [0.03 ; 11.73] | 0.72 [0.27 ; 1.94] | 0.79 [0.34 ; 1.80] | 0.70 [0.07 ; 7.05] | 0.84 [0.31 ; 2.24] | 0.87 [0.05 ; 14.87] | Lopinavir-Ritonavir | . | . | . | . | 0.53 [0.10 ; 2.74] | . | 0.71 [0.32 ; 1.57] | . | . |
| 0.06 [0.01 ; 0.26] | 0.07 [0.01 ; 0.57] | 0.08 [0.00 ; 1.52] | 0.14 [0.01 ; 1.37] | 0.18 [0.01 ; 3.86] | 0.18 [0.01 ; 4.01] | 0.18 [0.01 ; 4.33] | 0.36 [0.10 ; 1.34] | 0.43 [0.14 ; 1.27] | 0.55 [0.28 ; 1.05] | 0.60 [0.35 ; 1.05] | 0.50 [0.08 ; 3.14] | 0.43 [0.02 ; 11.84] | 0.56 [0.09 ; 3.48] | 0.52 [0.03 ; 10.50] | 0.68 [0.30 ; 1.54] | 0.74 [0.41 ; 1.36] | 0.66 [0.07 ; 6.20] | 0.79 [0.35 ; 1.77] | 0.82 [0.04 ; 15.80] | 0.94 [0.41 ; 2.17] | Azithromycin | . | . | . | 0.52 [0.24 ; 1.13] | . | 0.75 [0.48 ; 1.17] | . | . |
| 0.06 [0.01 ; 0.33] | 0.07 [0.01 ; 0.68] | 0.07 [0.00 ; 1.68] | 0.13 [0.01 ; 1.59] | 0.17 [0.01 ; 4.24] | 0.17 [0.01 ; 4.40] | 0.17 [0.01 ; 4.73] | 0.34 [0.06 ; 1.78] | 0.39 [0.09 ; 1.78] | 0.51 [0.15 ; 1.72] | 0.56 [0.17 ; 1.81] | 0.47 [0.06 ; 3.82] | 0.40 [0.01 ; 12.86] | 0.52 [0.06 ; 4.24] | 0.48 [0.02 ; 11.57] | 0.63 [0.17 ; 2.35] | 0.69 [0.21 ; 2.27] | 0.61 [0.05 ; 7.21] | 0.74 [0.20 ; 2.72] | 0.76 [0.03 ; 17.47] | 0.88 [0.23 ; 3.30] | 0.93 [0.28 ; 3.08] | Ivermectin | . | . | . | . | 0.76 [0.25 ; 2.33] | . | . |
| 0.05 [0.01 ; 0.27] | 0.06 [0.01 ; 0.56] | 0.06 [0.00 ; 1.42] | 0.12 [0.01 ; 1.32] | 0.15 [0.01 ; 3.57] | 0.15 [0.01 ; 3.71] | 0.15 [0.01 ; 3.99] | 0.30 [0.08 ; 1.05] | 0.35 [0.09 ; 1.41] | 0.45 [0.15 ; 1.32] | 0.50 [0.18 ; 1.38] | 0.41 [0.05 ; 3.13] | 0.36 [0.01 ; 10.88] | 0.46 [0.06 ; 3.47] | 0.43 [0.02 ; 9.74] | 0.56 [0.17 ; 1.83] | 0.61 [0.21 ; 1.74] | 0.55 [0.05 ; 5.98] | 0.65 [0.20 ; 2.11] | 0.67 [0.03 ; 14.70] | 0.78 [0.24 ; 2.57] | 0.82 [0.29 ; 2.36] | 0.89 [0.20 ; 3.88] | Ribavirin | . | . | . | 0.60 [0.21 ; 1.73] | . | . |
| 0.04 [0.01 ; 0.30] | 0.05 [0.00 ; 0.59] | 0.05 [0.00 ; 1.42] | 0.10 [0.01 ; 1.37] | 0.13 [0.00 ; 3.56] | 0.13 [0.00 ; 3.69] | 0.13 [0.00 ; 3.96] | 0.26 [0.04 ; 1.63] | 0.30 [0.05 ; 1.65] | 0.39 [0.09 ; 1.66] | 0.43 [0.10 ; 1.77] | 0.35 [0.04 ; 3.37] | 0.31 [0.01 ; 10.72] | 0.39 [0.04 ; 3.74] | 0.37 [0.02 ; 7.20] | 0.48 [0.10 ; 2.24] | 0.52 [0.12 ; 2.21] | 0.47 [0.04 ; 6.23] | 0.56 [0.12 ; 2.60] | 0.58 [0.02 ; 14.70] | 0.67 [0.14 ; 3.14] | 0.71 [0.17 ; 2.99] | 0.76 [0.13 ; 4.49] | 0.86 [0.16 ; 4.60] | IVIG | . | . | 1.00 [0.25 ; 3.96] | . | . |
| 0.05 [0.01 ; 0.19] | 0.05 [0.01 ; 0.42] | 0.06 [0.00 ; 1.11] | 0.11 [0.01 ; 1.00] | 0.13 [0.01 ; 2.85] | 0.14 [0.01 ; 2.96] | 0.14 [0.01 ; 3.20] | 0.27 [0.08 ; 0.96] | 0.32 [0.12 ; 0.90] | 0.41 [0.24 ; 0.70] | 0.46 [0.31 ; 0.68] | 0.38 [0.06 ; 2.28] | 0.33 [0.01 ; 8.76] | 0.42 [0.07 ; 2.53] | 0.39 [0.02 ; 7.75] | 0.52 [0.25 ; 1.06] | 0.56 [0.35 ; 0.89] | 0.50 [0.06 ; 4.54] | 0.60 [0.30 ; 1.21] | 0.62 [0.03 ; 11.64] | 0.71 [0.34 ; 1.48] | 0.76 [0.48 ; 1.20] | 0.82 [0.26 ; 2.54] | 0.92 [0.35 ; 2.45] | 1.07 [0.27 ; 4.31] | Hydroxychloroquine | . | 0.91 [0.75 ; 1.11] | . | . |
| 0.04 [0.01 ; 0.18] | 0.05 [0.01 ; 0.40] | 0.06 [0.00 ; 1.06] | 0.10 [0.01 ; 0.95] | 0.13 [0.01 ; 2.69] | 0.13 [0.01 ; 2.79] | 0.13 [0.01 ; 3.01] | 0.26 [0.07 ; 0.91] | 0.30 [0.11 ; 0.86] | 0.39 [0.22 ; 0.68] | 0.43 [0.27 ; 0.67] | 0.36 [0.06 ; 2.16] | 0.31 [0.01 ; 8.25] | 0.39 [0.06 ; 2.40] | 0.37 [0.02 ; 7.30] | 0.48 [0.23 ; 1.02] | 0.52 [0.31 ; 0.88] | 0.47 [0.05 ; 4.28] | 0.56 [0.27 ; 1.17] | 0.58 [0.03 ; 10.99] | 0.67 [0.31 ; 1.44] | 0.71 [0.42 ; 1.19] | 0.76 [0.24 ; 2.42] | 0.86 [0.31 ; 2.34] | 1.00 [0.25 ; 4.10] | 0.93 [0.66 ; 1.33] | Corticosteroid | 1.00 [0.74 ; 1.33] | . | . |
| 0.04 [0.01 ; 0.17] | 0.05 [0.01 ; 0.39] | 0.05 [0.00 ; 1.04] | 0.10 [0.01 ; 0.92] | 0.13 [0.01 ; 2.64] | 0.13 [0.01 ; 2.74] | 0.13 [0.01 ; 2.96] | 0.26 [0.07 ; 0.88] | 0.30 [0.11 ; 0.82] | 0.38 [0.24 ; 0.63] | 0.43 [0.30 ; 0.60] | 0.35 [0.06 ; 2.10] | 0.31 [0.01 ; 8.11] | 0.39 [0.07 ; 2.33] | 0.37 [0.02 ; 7.17] | 0.48 [0.24 ; 0.96] | 0.52 [0.34 ; 0.80] | 0.47 [0.05 ; 4.19] | 0.56 [0.28 ; 1.10] | 0.58 [0.03 ; 10.79] | 0.66 [0.33 ; 1.35] | 0.70 [0.46 ; 1.09] | 0.76 [0.25 ; 2.33] | 0.86 [0.33 ; 2.24] | 1.00 [0.25 ; 3.96] | 0.93 [0.77 ; 1.13] | 1.00 [0.74 ; 1.33] | Control | 0.89 [0.60 ; 1.34] | 0.88 [0.69 ; 1.11] |
| 0.04 [0.01 ; 0.16] | 0.04 [0.01 ; 0.35] | 0.05 [0.00 ; 0.93] | 0.09 [0.01 ; 0.83] | 0.11 [0.01 ; 2.36] | 0.11 [0.01 ; 2.45] | 0.11 [0.00 ; 2.64] | 0.22 [0.06 ; 0.81] | 0.26 [0.09 ; 0.77] | 0.34 [0.18 ; 0.63] | 0.37 [0.22 ; 0.63] | 0.31 [0.05 ; 1.91] | 0.27 [0.01 ; 7.23] | 0.34 [0.06 ; 2.12] | 0.32 [0.02 ; 6.41] | 0.42 [0.19 ; 0.93] | 0.46 [0.26 ; 0.81] | 0.41 [0.04 ; 3.78] | 0.49 [0.22 ; 1.06] | 0.50 [0.03 ; 9.65] | 0.58 [0.26 ; 1.31] | 0.61 [0.34 ; 1.10] | 0.66 [0.20 ; 2.17] | 0.75 [0.26 ; 2.11] | 0.87 [0.21 ; 3.65] | 0.81 [0.52 ; 1.26] | 0.87 [0.53 ; 1.42] | 0.87 [0.59 ; 1.30] | High dose hydroxychloroquine | 1.54 [0.45 ; 5.30] |
| 0.04 [0.01 ; 0.15] | 0.04 [0.01 ; 0.34] | 0.05 [0.00 ; 0.93] | 0.09 [0.01 ; 0.82] | 0.11 [0.01 ; 2.35] | 0.11 [0.01 ; 2.44] | 0.11 [0.00 ; 2.63] | 0.22 [0.06 ; 0.79] | 0.26 [0.09 ; 0.74] | 0.34 [0.20 ; 0.59] | 0.38 [0.25 ; 0.57] | 0.31 [0.05 ; 1.88] | 0.27 [0.01 ; 7.21] | 0.35 [0.06 ; 2.09] | 0.32 [0.02 ; 6.38] | 0.42 [0.20 ; 0.88] | 0.46 [0.28 ; 0.75] | 0.41 [0.05 ; 3.74] | 0.49 [0.26 ; 0.93] | 0.51 [0.03 ; 9.60] | 0.59 [0.28 ; 1.24] | 0.62 [0.38 ; 1.02] | 0.67 [0.21 ; 2.10] | 0.75 [0.28 ; 2.03] | 0.88 [0.22 ; 3.56] | 0.82 [0.60 ; 1.12] | 0.88 [0.60 ; 1.28] | 0.88 [0.69 ; 1.12] | 1.01 [0.64 ; 1.59] | Hydroxychloroquine plus azithromycin |

Pairwise (upper right portion) and network (lower left portion) meta-analysis results are presented. Pharmacological agents are reported in order of treatment efficacy ranking according to SUCRAs. Comparison should be read from left to right. Effect estimation is presented in odds ratio (OR) with 95% CI and is located in intersection of two agents. OR less than 1 favors the column-defining treatment (lower mortality). Since lower mortality rate reflects better outcome, decrement of OR indicates better treatment. To obtain OR (95% CI) for comparison in the opposite direction, reciprocals should be taken.

- 1. Direct and indirect evidence proportion for each outcome


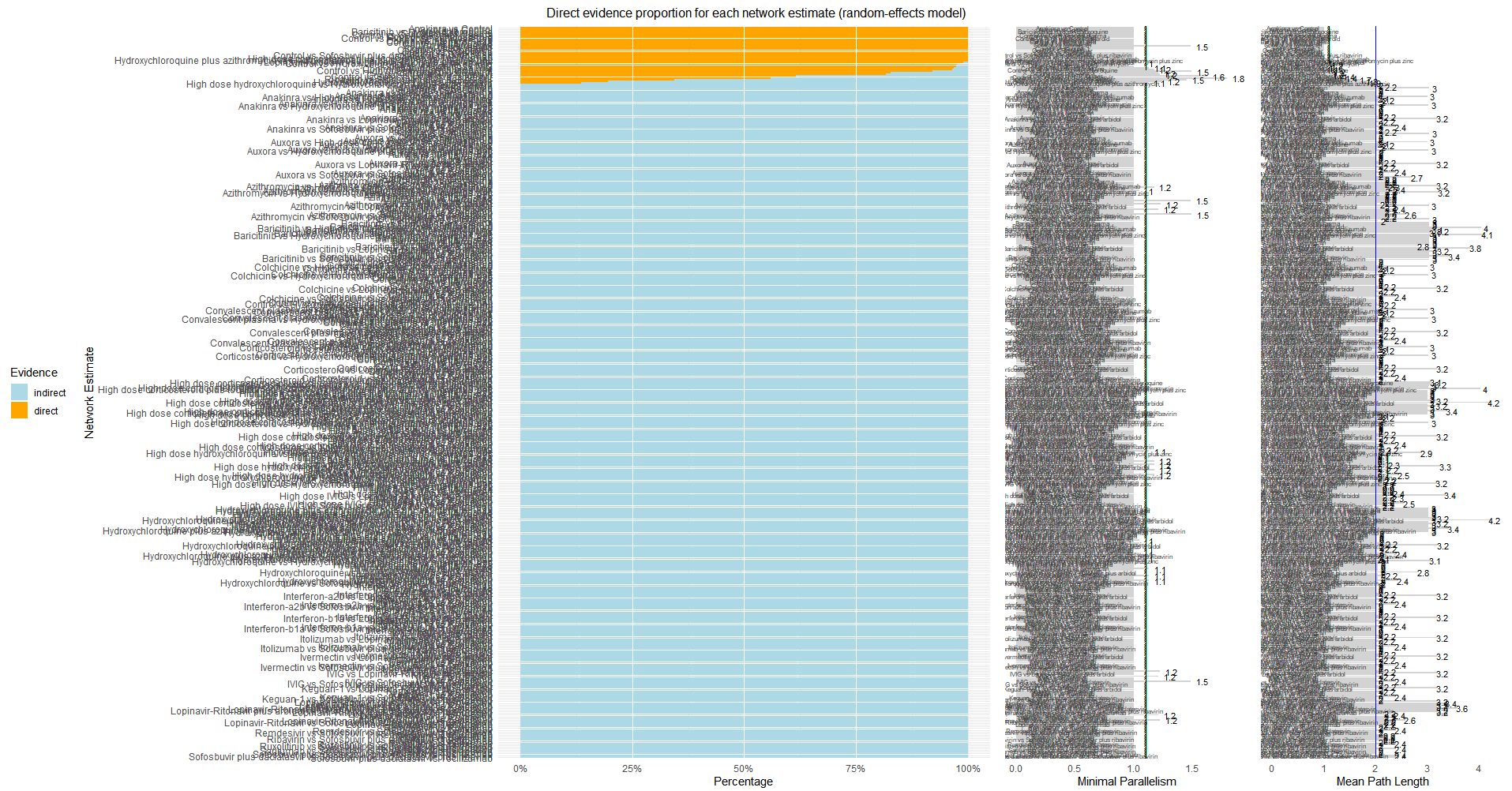


 According to König, Krahn, and Binder ([2013](https://bookdown.org/MathiasHarrer/Doing_Meta_Analysis_in_R/frequentist.html#ref-konig2013visualizing)), lower values of minimal parallelism and Mean Path Length>2 means that results for a specific comparison should be interpreted with caution.

- 1. Heterogeneity
     1. Quantifying heterogeneity: tau^2 = 0.0437; tau = 0.2091; I^2 = 37.6% [9.2%; 57.1%]
  2. Inconsistency
     1. Q statistic to assess consistency under the assumption of a full design-by-treatment interaction random effects model: Q = 5.80, p value = 0.5630
  3. Net heat plot


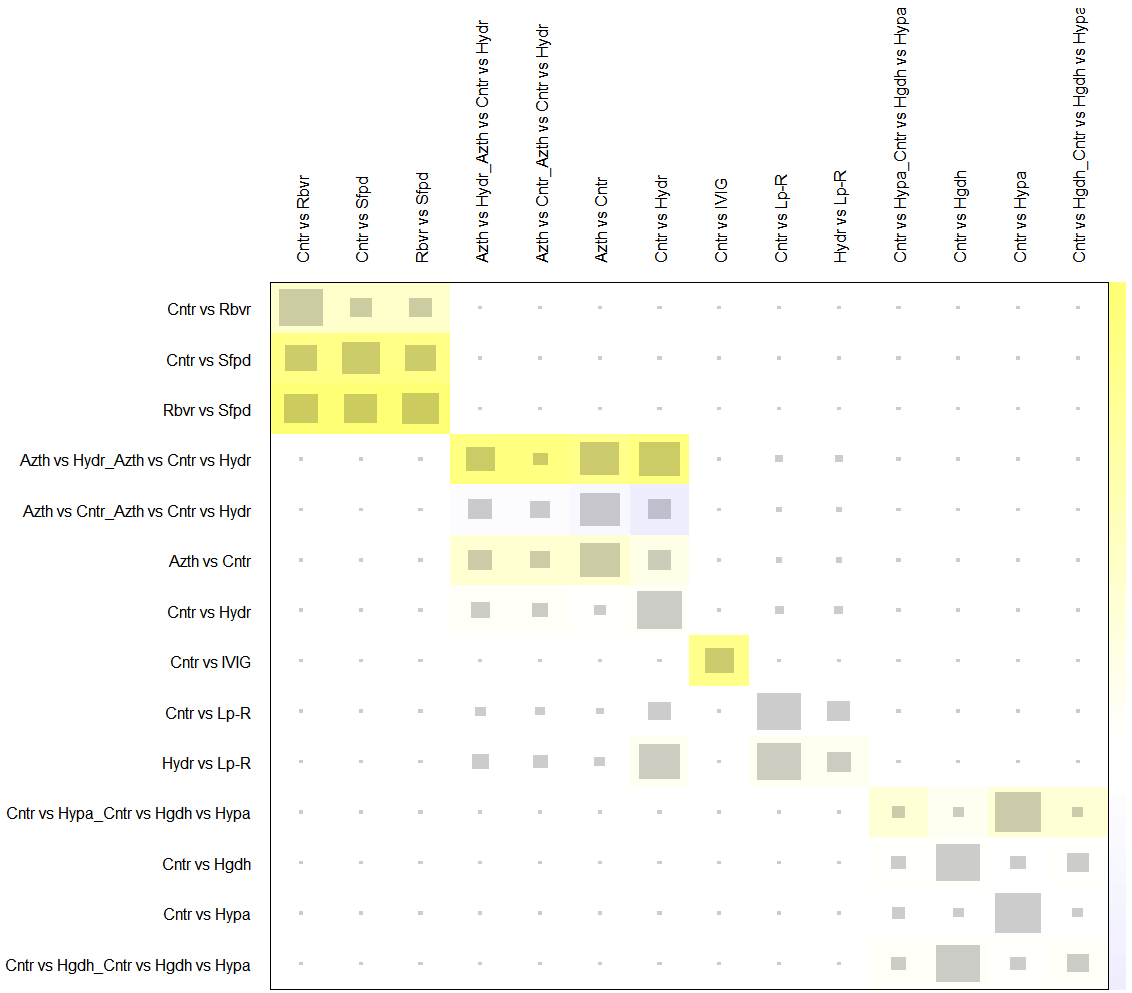


- 1. Comparison-adjusted funnel plot


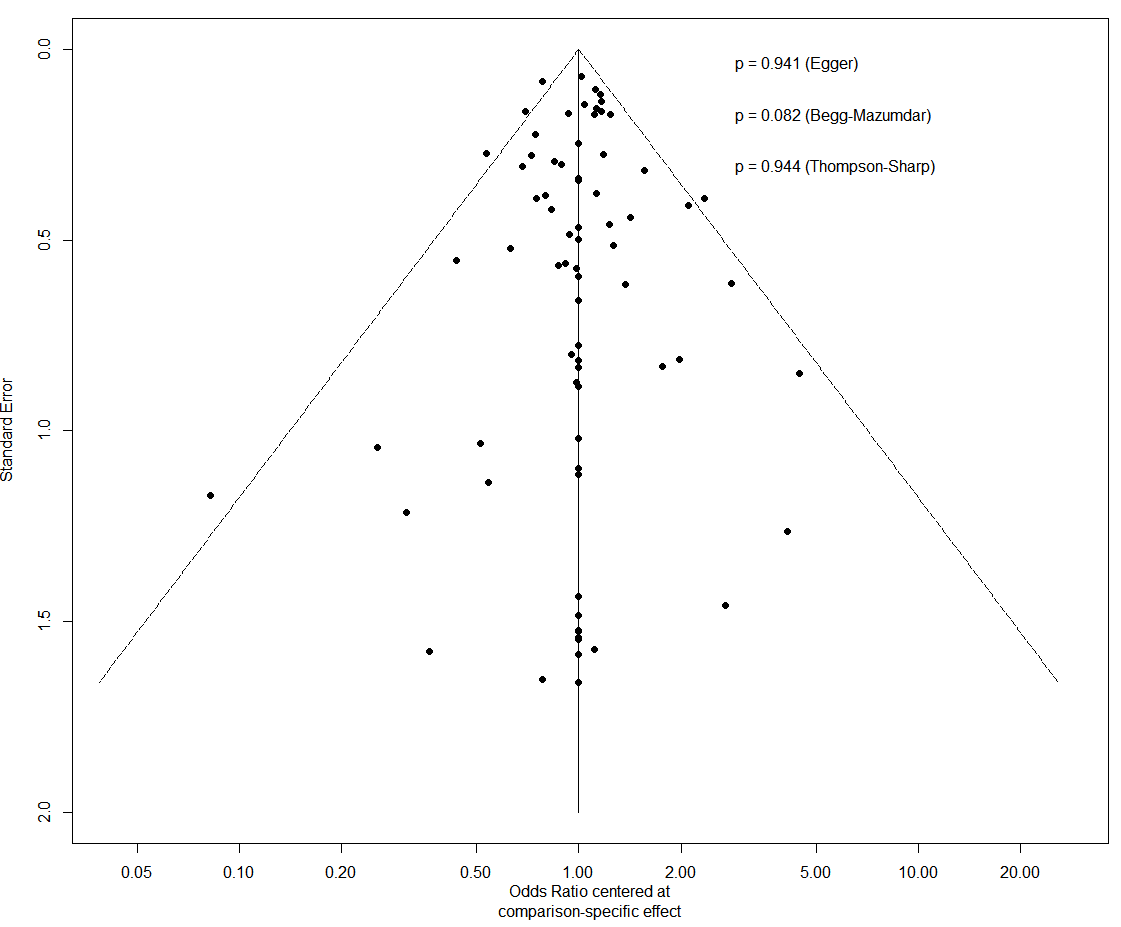


1. **Mortality in ICU patients (critically ill)**
   1. Network map


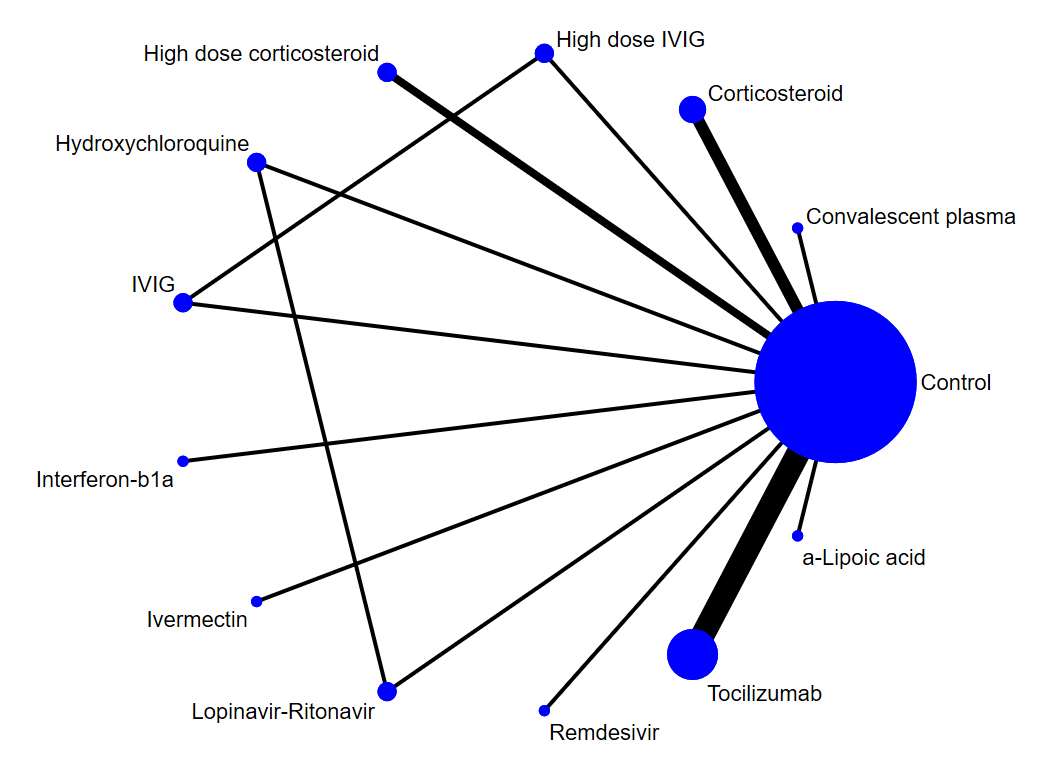


- 1. Forest plot


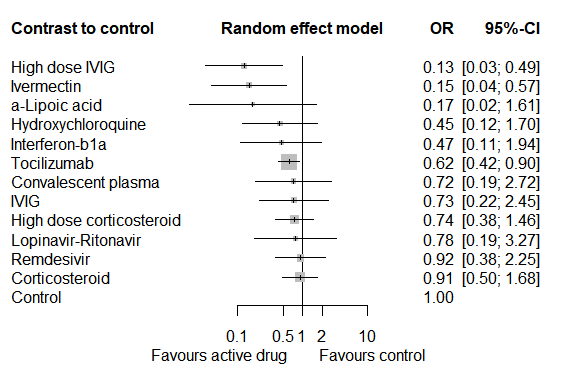


- 1. League table

| High dose IVIG | . | . | . | . | . | . | 0.17 [0.04 ; 0.68] | . | . | . | . | 0.13 [0.03 ; 0.49] |
| --- | --- | --- | --- | --- | --- | --- | --- | --- | --- | --- | --- | --- |
| 0.84 [0.12 ; 5.62] | Ivermectin | . | . | . | . | . | . | . | . | . | . | 0.15 [0.04 ; 0.57] |
| 0.74 [0.05 ;10.10] | 0.88 [0.06 ;11.91] | a-Lipoic acid | . | . | . | . | . | . | . | . | . | 0.17 [0.02 ; 1.61] |
| 0.28 [0.04 ; 1.89] | 0.34 [0.05 ; 2.21] | 0.38 [0.03 ; 5.18] | Hydroxychloroquine | . | . | . | . | . | 0.58 [0.15 ; 2.29] | . | . | 0.45 [0.12 ; 1.70] |
| 0.27 [0.04 ; 1.93] | 0.32 [0.05 ; 2.27] | 0.37 [0.03 ; 5.20] | 0.96 [0.14 ; 6.73] | Interferon-b1a | . | . | . | . | . | . | . | 0.47 [0.11 ; 1.94] |
| 0.20 [0.05 ; 0.84] | 0.24 [0.06 ; 0.98] | 0.28 [0.03 ; 2.69] | 0.73 [0.18 ; 2.91] | 0.76 [0.17 ; 3.31] | Tocilizumab | . | . | . | . | . | . | 0.62 [0.42 ; 0.90] |
| 0.18 [0.03 ; 1.17] | 0.21 [0.03 ; 1.37] | 0.24 [0.02 ; 3.21] | 0.62 [0.09 ; 4.09] | 0.65 [0.09 ; 4.55] | 0.86 [0.22 ; 3.40] | Convalescent plasma | . | . | . | . | . | 0.72 [0.19 ; 2.72] |
| 0.17 [0.04 ; 0.68] | 0.21 [0.03 ; 1.26] | 0.24 [0.02 ; 3.01] | 0.62 [0.10 ; 3.74] | 0.64 [0.10 ; 4.18] | 0.85 [0.24 ; 3.03] | 0.99 [0.16 ; 5.99] | IVIG | . | . | . | . | 0.73 [0.22 ; 2.45] |
| 0.17 [0.04 ; 0.78] | 0.20 [0.05 ; 0.90] | 0.23 [0.02 ; 2.39] | 0.60 [0.14 ; 2.69] | 0.63 [0.13 ; 3.04] | 0.83 [0.38 ; 1.80] | 0.97 [0.22 ; 4.30] | 0.98 [0.24 ; 3.92] | High dose corticosteroid | . | . | . | 0.74 [0.38 ; 1.46] |
| 0.16 [0.02 ; 1.17] | 0.19 [0.03 ; 1.37] | 0.22 [0.02 ; 3.15] | 0.58 [0.15 ; 2.29] | 0.60 [0.08 ; 4.54] | 0.79 [0.18 ; 3.49] | 0.93 [0.13 ; 6.54] | 0.93 [0.14 ; 6.13] | 0.96 [0.20 ; 4.67] | Lopinavir-Ritonavir | . | . | 0.78 [0.19 ; 3.27] |
| 0.14 [0.03 ; 0.70] | 0.16 [0.03 ; 0.81] | 0.19 [0.02 ; 2.07] | 0.49 [0.10 ; 2.42] | 0.51 [0.09 ; 2.72] | 0.67 [0.25 ; 1.76] | 0.78 [0.16 ; 3.87] | 0.79 [0.17 ; 3.56] | 0.81 [0.26 ; 2.47] | 0.84 [0.16 ; 4.57] | Remdesivir | . | 0.92 [0.38 ; 2.25] |
| 0.14 [0.03 ; 0.61] | 0.17 [0.04 ; 0.71] | 0.19 [0.02 ; 1.91] | 0.49 [0.11 ; 2.12] | 0.51 [0.11 ; 2.40] | 0.67 [0.33 ; 1.38] | 0.79 [0.18 ; 3.40] | 0.80 [0.20 ; 3.09] | 0.81 [0.33 ; 2.02] | 0.85 [0.18 ; 4.05] | 1.01 [0.34 ; 2.97] | Corticosteroid | 0.91 [0.50 ; 1.68] |
| 0.13 [0.03 ; 0.49] | 0.15 [0.04 ; 0.57] | 0.17 [0.02 ; 1.61] | 0.45 [0.12 ; 1.70] | 0.47 [0.11 ; 1.94] | 0.62 [0.42 ; 0.90] | 0.72 [0.19 ; 2.72] | 0.73 [0.22 ; 2.45] | 0.74 [0.38 ; 1.46] | 0.78 [0.19 ; 3.27] | 0.92 [0.38 ; 2.25] | 0.91 [0.50 ; 1.68] | Control |

Pairwise (upper right portion) and network (lower left portion) meta-analysis results are presented. Pharmacological agents are reported in order of treatment efficacy ranking according to SUCRAs. Comparison should be read from left to right. Effect estimation is presented in odds ratio (OR) with 95% CI and is located in intersection of two agents. OR less than 1 favors the column-defining treatment (lower mortality). Since lower mortality rate reflects better outcome, decrement of OR indicates better treatment. To obtain OR (95% CI) for comparison in the opposite direction, reciprocals should be taken

- 1. Direct and indirect evidence proportion for each outcome


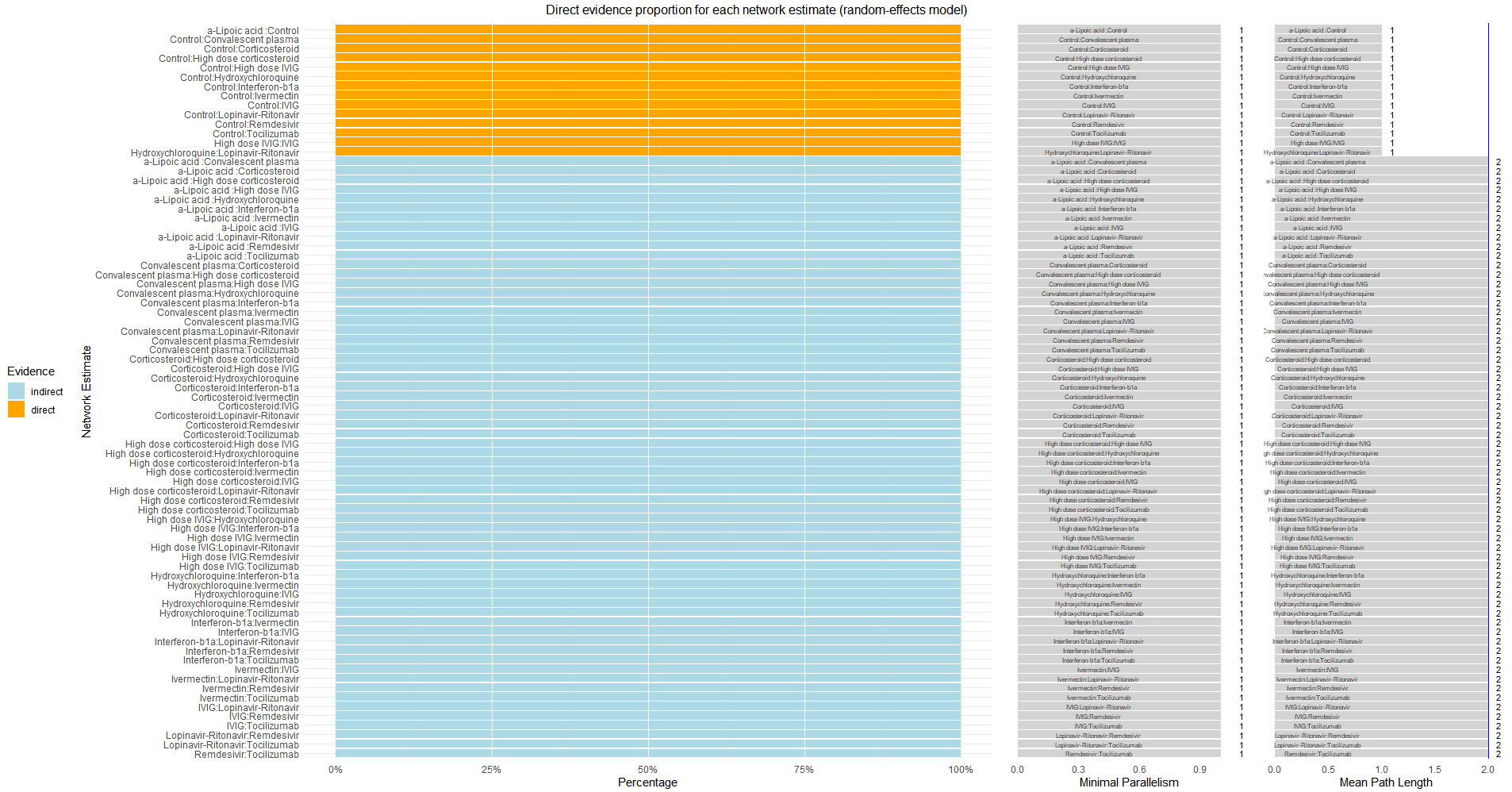


 According to König, Krahn, and Binder ([2013](https://bookdown.org/MathiasHarrer/Doing_Meta_Analysis_in_R/frequentist.html#ref-konig2013visualizing)), lower values of minimal parallelism and Mean Path Length>2 means that results for a specific comparison should be interpreted with caution.

- 1. Heterogeneity
     1. Quantifying heterogeneity: no detectable heterogeneity: tau^2 = 0.1285; tau = 0.3585; I^2 = 62% [21.5%; 81.6%]
  2. Inconsistency
     1. Q statistic to assess consistency under the assumption of a full design-by-treatment interaction random effects model: p value = 1.000
  3. Net heat plot
     1. Net heat plot not available due to insufficient information about between-design heterogeneity
  4. Comparison-adjusted funnel plot


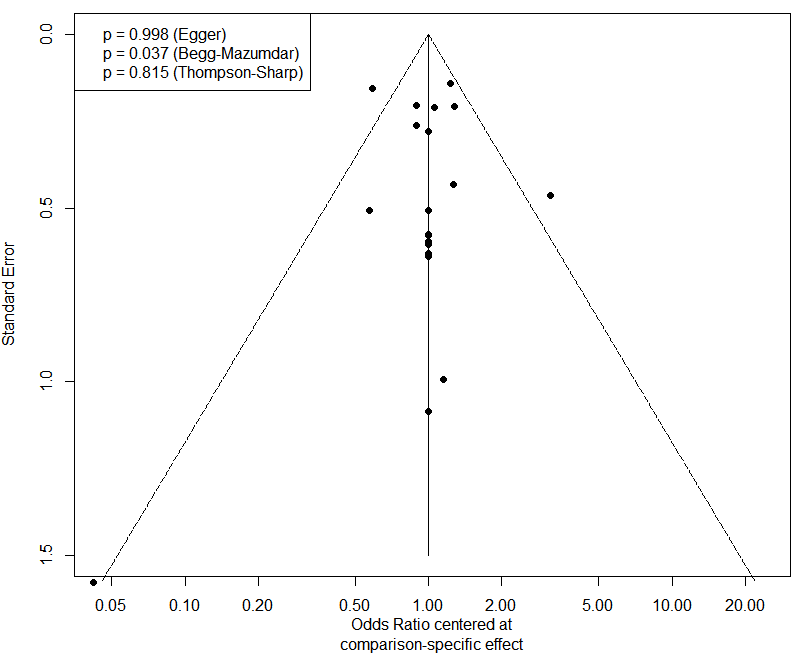


1. **Progression to severe course (progress to severe pneumonia or admission to ICU)**
   1. Network map


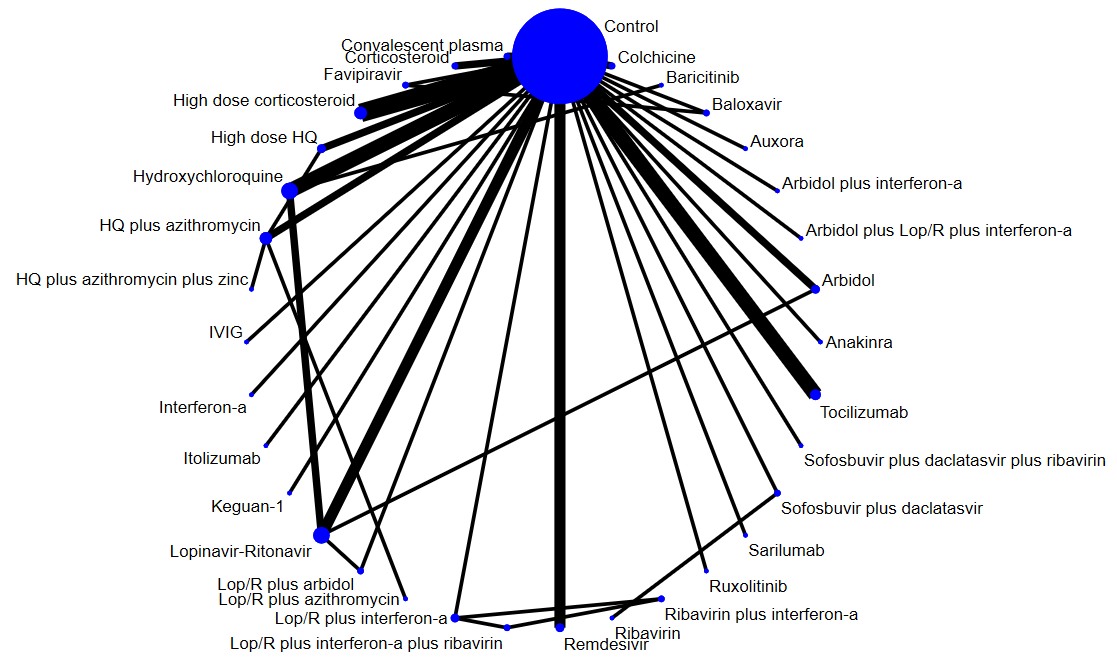


- 1. Forest plot


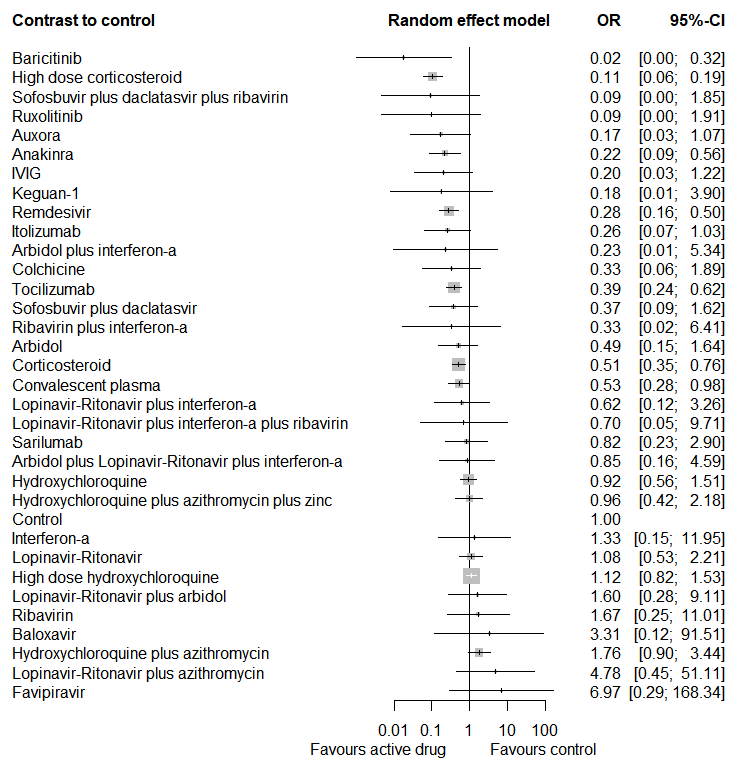


- 1. League table

| Baricitinib | . | . | . | . | . | . | . | . | . | . | . | . | . | . | . | . | . | . | . | . | . | 0.02 [0.00 ; 0.34] | . | . | . | . | . | . | . | . | . | . | . |
| --- | --- | --- | --- | --- | --- | --- | --- | --- | --- | --- | --- | --- | --- | --- | --- | --- | --- | --- | --- | --- | --- | --- | --- | --- | --- | --- | --- | --- | --- | --- | --- | --- | --- |
| 0.17 [0.01 ; 3.25] | High dose corticosteroid | . | . | . | . | . | . | . | . | . | . | . | . | . | . | . | . | . | . | . | . | . | . | 0.11 [0.06 ; 0.19] | . | . | . | . | . | . | . | . | . |
| 0.19 [0.00 ; 12.41] | 1.14 [0.05 ; 24.05] | Sofosbuvir plus daclatasvir plus ribavirin | . | . | . | . | . | . | . | . | . | . | . | . | . | . | . | . | . | . | . | . | . | 0.09 [0.00 ; 1.85] | . | . | . | . | . | . | . | . | . |
| 0.19 [0.00 ; 12.25] | 1.12 [0.05 ; 23.81] | 0.98 [0.01 ; 67.72] | Ruxolitinib | . | . | . | . | . | . | . | . | . | . | . | . | . | . | . | . | . | . | . | . | 0.09 [0.00 ; 1.91] | . | . | . | . | . | . | . | . | . |
| 0.11 [0.00 ; 3.21] | 0.62 [0.09 ; 4.23] | 0.54 [0.02 ; 18.04] | 0.55 [0.02 ; 18.57] | Auxora | . | . | . | . | . | . | . | . | . | . | . | . | . | . | . | . | . | . | . | 0.17 [0.03 ; 1.07] | . | . | . | . | . | . | . | . | . |
| 0.08 [0.00 ; 1.70] | 0.48 [0.16 ; 1.45] | 0.42 [0.02 ; 9.66] | 0.43 [0.02 ; 9.96] | 0.78 [0.10 ; 6.05] | Anakinra | . | . | . | . | . | . | . | . | . | . | . | . | . | . | . | . | . | . | 0.22 [0.09 ; 0.56] | . | . | . | . | . | . | . | . | . |
| 0.09 [0.00 ; 2.64] | 0.52 [0.08 ; 3.41] | 0.46 [0.01 ; 14.82] | 0.46 [0.01 ; 15.25] | 0.84 [0.07 ; 10.81] | 1.08 [0.14 ; 8.05] | IVIG | . | . | . | . | . | . | . | . | . | . | . | . | . | . | . | . | . | 0.20 [0.03 ; 1.22] | . | . | . | . | . | . | . | . | . |
| 0.10 [0.00 ; 7.12] | 0.60 [0.03 ; 14.20] | 0.53 [0.01 ; 39.32] | 0.54 [0.01 ; 40.41] | 0.98 [0.03 ; 35.72] | 1.25 [0.05 ; 31.87] | 1.16 [0.03 ; 41.61] | Keguan-1 | . | . | . | . | . | . | . | . | . | . | . | . | . | . | . | . | 0.18 [0.01 ; 3.90] | . | . | . | . | . | . | . | . | . |
| 0.06 [0.00 ; 1.22] | 0.38 [0.16 ; 0.86] | 0.33 [0.02 ; 6.93] | 0.34 [0.02 ; 7.15] | 0.61 [0.09 ; 4.14] | 0.78 [0.26 ; 2.33] | 0.72 [0.11 ; 4.73] | 0.62 [0.03 ; 14.59] | Remdesivir | . | . | . | . | . | . | . | . | . | . | . | . | . | . | . | 0.28 [0.16 ; 0.50] | . | . | . | . | . | . | . | . | . |
| 0.07 [0.00 ; 1.70] | 0.41 [0.09 ; 1.83] | 0.36 [0.01 ; 9.62] | 0.36 [0.01 ; 9.91] | 0.66 [0.07 ; 6.51] | 0.85 [0.16 ; 4.45] | 0.78 [0.08 ; 7.49] | 0.68 [0.02 ; 20.08] | 1.08 [0.24 ; 4.83] | Itolizumab | . | . | . | . | . | . | . | . | . | . | . | . | . | . | 0.26 [0.07 ; 1.03] | . | . | . | . | . | . | . | . | . |
| 0.08 [0.00 ; 5.62] | 0.46 [0.02 ; 11.32] | 0.40 [0.01 ; 30.98] | 0.41 [0.01 ; 31.84] | 0.75 [0.02 ; 28.32] | 0.96 [0.04 ; 25.37] | 0.89 [0.02 ; 33.00] | 0.76 [0.01 ; 63.16] | 1.23 [0.05 ; 29.98] | 1.13 [0.04 ; 35.03] | Arbidol plus interferon-a | . | . | . | . | . | . | . | . | . | . | . | . | . | 0.23 [0.01 ; 5.34] | . | . | . | . | . | . | . | . | . |
| 0.05 [0.00 ; 1.59] | 0.32 [0.05 ; 2.02] | 0.28 [0.01 ; 8.95] | 0.29 [0.01 ; 9.21] | 0.52 [0.04 ; 6.48] | 0.66 [0.09 ; 4.78] | 0.62 [0.05 ; 7.48] | 0.53 [0.02 ; 18.59] | 0.85 [0.14 ; 5.34] | 0.79 [0.09 ; 7.25] | 0.70 [0.02 ; 25.33] | Colchicine | . | . | . | . | . | . | . | . | . | . | . | . | 0.33 [0.06 ; 1.89] | . | . | . | . | . | . | . | . | . |
| 0.05 [0.00 ; 0.87] | 0.27 [0.13 ; 0.59] | 0.24 [0.01 ; 4.96] | 0.25 [0.01 ; 5.12] | 0.44 [0.07 ; 2.93] | 0.57 [0.20 ; 1.61] | 0.53 [0.08 ; 3.35] | 0.45 [0.02 ; 10.45] | 0.73 [0.35 ; 1.54] | 0.67 [0.16 ; 2.89] | 0.60 [0.02 ; 14.31] | 0.86 [0.14 ; 5.21] | Tocilizumab | . | . | . | . | . | . | . | . | . | . | . | 0.39 [0.24 ; 0.62] | . | . | . | . | . | . | . | . | . |
| 0.05 [0.00 ; 1.24] | 0.29 [0.06 ; 1.40] | 0.25 [0.01 ; 7.01] | 0.26 [0.01 ; 7.22] | 0.46 [0.04 ; 4.82] | 0.59 [0.10 ; 3.37] | 0.55 [0.05 ; 5.55] | 0.47 [0.02 ; 14.61] | 0.76 [0.16 ; 3.68] | 0.70 [0.09 ; 5.25] | 0.62 [0.02 ; 19.93] | 0.89 [0.09 ; 8.71] | 1.04 [0.22 ; 4.86] | Sofosbuvir plus daclatasvir | . | . | . | . | . | . | . | . | . | . | 0.37 [0.09 ; 1.62] | . | . | . | . | 0.22 [0.07 ; 0.73] | . | . | . | . |
| 0.05 [0.00 ; 3.45] | 0.32 [0.02 ; 6.64] | 0.28 [0.00 ; 19.06] | 0.29 [0.00 ; 19.59] | 0.52 [0.02 ; 16.99] | 0.67 [0.03 ; 14.96] | 0.62 [0.02 ; 19.78] | 0.53 [0.01 ; 38.95] | 0.86 [0.04 ; 17.59] | 0.79 [0.03 ; 20.81] | 0.70 [0.01 ; 52.71] | 1.00 [0.03 ; 31.38] | 1.17 [0.06 ; 23.65] | 1.13 [0.04 ; 30.95] | Ribavirin plus interferon-a | . | . | . | 0.53 [0.05 ; 6.22] | 0.47 [0.04 ; 5.50] | . | . | . | . | . | . | . | . | . | . | . | . | . | . |
| 0.04 [0.00 ; 0.82] | 0.21 [0.06 ; 0.82] | 0.19 [0.01 ; 4.71] | 0.19 [0.01 ; 4.85] | 0.35 [0.04 ; 3.08] | 0.44 [0.10 ; 2.02] | 0.41 [0.05 ; 3.54] | 0.35 [0.01 ; 9.85] | 0.57 [0.15 ; 2.15] | 0.53 [0.08 ; 3.26] | 0.47 [0.02 ; 13.45] | 0.67 [0.08 ; 5.54] | 0.78 [0.22 ; 2.82] | 0.75 [0.11 ; 5.00] | 0.67 [0.03 ; 16.34] | Arbidol | . | . | . | . | . | . | . | . | 0.74 [0.19 ; 2.94] | . | 0.30 [0.07 ; 1.29] | . | . | . | . | . | . | . |
| 0.04 [0.00 ; 0.65] | 0.21 [0.10 ; 0.42] | 0.18 [0.01 ; 3.70] | 0.18 [0.01 ; 3.81] | 0.33 [0.05 ; 2.17] | 0.43 [0.16 ; 1.17] | 0.40 [0.06 ; 2.47] | 0.34 [0.02 ; 7.78] | 0.55 [0.27 ; 1.10] | 0.51 [0.12 ; 2.12] | 0.45 [0.02 ; 10.66] | 0.64 [0.11 ; 3.85] | 0.75 [0.41 ; 1.39] | 0.72 [0.16 ; 3.32] | 0.64 [0.03 ; 12.81] | 0.96 [0.27 ; 3.40] | Corticosteroid | . | . | . | . | . | . | . | 0.51 [0.35 ; 0.76] | . | . | . | . | . | . | . | . | . |
| 0.03 [0.00 ; 0.66] | 0.20 [0.09 ; 0.48] | 0.18 [0.01 ; 3.75] | 0.18 [0.01 ; 3.87] | 0.33 [0.05 ; 2.25] | 0.42 [0.14 ; 1.28] | 0.39 [0.06 ; 2.57] | 0.33 [0.01 ; 7.89] | 0.54 [0.23 ; 1.26] | 0.49 [0.11 ; 2.25] | 0.44 [0.02 ; 10.80] | 0.63 [0.10 ; 4.01] | 0.73 [0.34 ; 1.60] | 0.71 [0.14 ; 3.49] | 0.63 [0.03 ; 13.01] | 0.94 [0.24 ; 3.63] | 0.98 [0.47 ; 2.04] | Convalescent plasma | . | . | . | . | . | . | 0.53 [0.28 ; 0.98] | . | . | . | . | . | . | . | . | . |
| 0.03 [0.00 ; 0.81] | 0.17 [0.03 ; 1.00] | 0.15 [0.00 ; 4.58] | 0.15 [0.00 ; 4.72] | 0.28 [0.02 ; 3.27] | 0.35 [0.05 ; 2.37] | 0.33 [0.03 ; 3.77] | 0.28 [0.01 ; 9.53] | 0.45 [0.08 ; 2.64] | 0.42 [0.05 ; 3.63] | 0.37 [0.01 ; 12.99] | 0.53 [0.05 ; 5.92] | 0.62 [0.11 ; 3.49] | 0.60 [0.07 ; 5.50] | 0.53 [0.05 ; 6.22] | 0.80 [0.10 ; 6.17] | 0.83 [0.15 ; 4.56] | 0.85 [0.14 ; 4.99] | Lopinavir-Ritonavir plus interferon-a | 0.88 [0.12 ; 6.75] | . | . | . | . | 0.62 [0.12 ; 3.26] | . | . | . | . | . | . | . | . | . |
| 0.03 [0.00 ; 1.27] | 0.15 [0.01 ; 2.23] | 0.13 [0.00 ; 7.07] | 0.13 [0.00 ; 7.27] | 0.24 [0.01 ; 5.98] | 0.31 [0.02 ; 5.07] | 0.29 [0.01 ; 6.95] | 0.25 [0.00 ; 14.52] | 0.40 [0.03 ; 5.90] | 0.37 [0.02 ; 7.18] | 0.33 [0.01 ; 19.69] | 0.47 [0.02 ; 11.01] | 0.55 [0.04 ; 7.91] | 0.53 [0.03 ; 10.72] | 0.47 [0.04 ; 5.50] | 0.70 [0.04 ; 12.61] | 0.73 [0.05 ; 10.39] | 0.75 [0.05 ; 11.12] | 0.88 [0.12 ; 6.75] | Lopinavir-Ritonavir plus interferon-a plus ribavirin | . | . | . | . | . | . | . | . | . | . | . | . | . | . |
| 0.02 [0.00 ; 0.52] | 0.13 [0.03 ; 0.53] | 0.11 [0.00 ; 2.92] | 0.12 [0.00 ; 3.01] | 0.21 [0.02 ; 1.94] | 0.27 [0.06 ; 1.29] | 0.25 [0.03 ; 2.23] | 0.21 [0.01 ; 6.11] | 0.34 [0.09 ; 1.39] | 0.32 [0.05 ; 2.07] | 0.28 [0.01 ; 8.34] | 0.40 [0.05 ; 3.49] | 0.47 [0.12 ; 1.82] | 0.45 [0.07 ; 3.16] | 0.40 [0.02 ; 10.15] | 0.60 [0.11 ; 3.45] | 0.63 [0.17 ; 2.36] | 0.64 [0.16 ; 2.64] | 0.76 [0.09 ; 6.11] | 0.86 [0.05 ; 15.85] | Sarilumab | . | . | . | 0.82 [0.23 ; 2.90] | . | . | . | . | . | . | . | . | . |
| 0.02 [0.00 ; 0.60] | 0.12 [0.02 ; 0.75] | 0.11 [0.00 ; 3.39] | 0.11 [0.00 ; 3.49] | 0.20 [0.02 ; 2.43] | 0.26 [0.04 ; 1.77] | 0.24 [0.02 ; 2.80] | 0.21 [0.01 ; 7.04] | 0.33 [0.06 ; 1.97] | 0.31 [0.03 ; 2.70] | 0.27 [0.01 ; 9.60] | 0.39 [0.03 ; 4.40] | 0.45 [0.08 ; 2.62] | 0.44 [0.05 ; 4.10] | 0.39 [0.01 ; 11.77] | 0.58 [0.07 ; 4.61] | 0.60 [0.11 ; 3.41] | 0.62 [0.10 ; 3.74] | 0.73 [0.07 ; 7.78] | 0.83 [0.04 ; 18.74] | 0.96 [0.12 ; 7.94] | Arbidol plus Lopinavir-Ritonavir plus interferon-a | . | . | 0.85 [0.16 ; 4.59] | . | . | . | . | . | . | . | . | . |
| 0.02 [0.00 ; 0.34] | 0.12 [0.05 ; 0.25] | 0.10 [0.00 ; 2.09] | 0.10 [0.00 ; 2.15] | 0.19 [0.03 ; 1.23] | 0.24 [0.08 ; 0.68] | 0.22 [0.03 ; 1.41] | 0.19 [0.01 ; 4.40] | 0.31 [0.14 ; 0.65] | 0.28 [0.07 ; 1.22] | 0.25 [0.01 ; 6.02] | 0.36 [0.06 ; 2.20] | 0.42 [0.21 ; 0.83] | 0.40 [0.09 ; 1.90] | 0.36 [0.02 ; 7.24] | 0.54 [0.15 ; 1.87] | 0.56 [0.30 ; 1.05] | 0.57 [0.26 ; 1.26] | 0.67 [0.12 ; 3.80] | 0.76 [0.05 ; 11.03] | 0.89 [0.23 ; 3.45] | 0.92 [0.16 ; 5.35] | Hydroxychloroquine | . | 0.88 [0.52 ; 1.46] | . | 0.95 [0.39 ; 2.27] | . | . | . | . | . | . | . |
| 0.02 [0.00 ; 0.38] | 0.11 [0.04 ; 0.30] | 0.10 [0.00 ; 2.15] | 0.10 [0.00 ; 2.21] | 0.18 [0.02 ; 1.32] | 0.23 [0.07 ; 0.79] | 0.21 [0.03 ; 1.51] | 0.18 [0.01 ; 4.51] | 0.29 [0.11 ; 0.80] | 0.27 [0.05 ; 1.34] | 0.24 [0.01 ; 6.16] | 0.34 [0.05 ; 2.36] | 0.40 [0.16 ; 1.03] | 0.39 [0.07 ; 2.08] | 0.34 [0.02 ; 7.44] | 0.51 [0.12 ; 2.19] | 0.53 [0.21 ; 1.32] | 0.55 [0.19 ; 1.53] | 0.64 [0.10 ; 4.10] | 0.73 [0.05 ; 11.43] | 0.85 [0.19 ; 3.84] | 0.88 [0.14 ; 5.77] | 0.96 [0.37 ; 2.49] | Hydroxychloroquine plus azithromycin plus zinc | . | . | . | . | . | . | . | 0.55 [0.34 ; 0.88] | . | . |
| 0.02 [0.00 ; 0.32] | 0.11 [0.06 ; 0.19] | 0.09 [0.00 ; 1.85] | 0.09 [0.00 ; 1.91] | 0.17 [0.03 ; 1.07] | 0.22 [0.09 ; 0.56] | 0.20 [0.03 ; 1.22] | 0.18 [0.01 ; 3.90] | 0.28 [0.16 ; 0.50] | 0.26 [0.07 ; 1.03] | 0.23 [0.01 ; 5.34] | 0.33 [0.06 ; 1.89] | 0.39 [0.24 ; 0.62] | 0.37 [0.09 ; 1.62] | 0.33 [0.02 ; 6.41] | 0.49 [0.15 ; 1.64] | 0.51 [0.35 ; 0.76] | 0.53 [0.28 ; 0.98] | 0.62 [0.12 ; 3.26] | 0.70 [0.05 ; 9.71] | 0.82 [0.23 ; 2.90] | 0.85 [0.16 ; 4.59] | 0.92 [0.56 ; 1.51] | 0.96 [0.42 ; 2.18] | Control | 0.75 [0.08 ; 6.75] | 0.72 [0.30 ; 1.75] | 0.90 [0.66 ; 1.23] | 1.05 [0.13 ; 8.45] | . | 0.30 [0.01 ; 8.36] | 0.56 [0.26 ; 1.21] | . | 0.14 [0.01 ; 3.47] |
| 0.01 [0.00 ; 0.51] | 0.08 [0.01 ; 0.78] | 0.07 [0.00 ; 2.85] | 0.07 [0.00 ; 2.94] | 0.13 [0.01 ; 2.24] | 0.17 [0.02 ; 1.79] | 0.15 [0.01 ; 2.60] | 0.13 [0.00 ; 5.89] | 0.21 [0.02 ; 2.05] | 0.20 [0.01 ; 2.61] | 0.17 [0.00 ; 8.01] | 0.25 [0.02 ; 4.10] | 0.29 [0.03 ; 2.74] | 0.28 [0.02 ; 3.92] | 0.25 [0.01 ; 9.93] | 0.37 [0.03 ; 4.53] | 0.39 [0.04 ; 3.59] | 0.40 [0.04 ; 3.87] | 0.47 [0.03 ; 7.31] | 0.53 [0.02 ; 16.19] | 0.62 [0.05 ; 7.76] | 0.64 [0.04 ; 10.19] | 0.69 [0.07 ; 6.57] | 0.72 [0.07 ; 7.54] | 0.75 [0.08 ; 6.75] | Interferon-a | . | . | . | . | . | . | . | . |
| 0.02 [0.00 ; 0.31] | 0.10 [0.04 ; 0.25] | 0.09 [0.00 ; 1.85] | 0.09 [0.00 ; 1.91] | 0.16 [0.02 ; 1.12] | 0.20 [0.06 ; 0.65] | 0.19 [0.03 ; 1.29] | 0.16 [0.01 ; 3.89] | 0.26 [0.10 ; 0.65] | 0.24 [0.05 ; 1.13] | 0.21 [0.01 ; 5.33] | 0.31 [0.05 ; 2.00] | 0.36 [0.15 ; 0.83] | 0.34 [0.07 ; 1.75] | 0.30 [0.01 ; 6.42] | 0.46 [0.14 ; 1.50] | 0.47 [0.21 ; 1.07] | 0.48 [0.19 ; 1.25] | 0.57 [0.09 ; 3.48] | 0.65 [0.04 ; 9.84] | 0.75 [0.18 ; 3.22] | 0.78 [0.13 ; 4.89] | 0.85 [0.42 ; 1.74] | 0.89 [0.30 ; 2.63] | 0.92 [0.45 ; 1.88] | 1.23 [0.12 ; 12.32] | Lopinavir-Ritonavir | . | 0.22 [0.01 ; 4.73] | . | . | . | . | . |
| 0.02 [0.00 ; 0.29] | 0.09 [0.05 ; 0.19] | 0.08 [0.00 ; 1.68] | 0.08 [0.00 ; 1.73] | 0.15 [0.02 ; 0.98] | 0.20 [0.07 ; 0.52] | 0.18 [0.03 ; 1.11] | 0.16 [0.01 ; 3.53] | 0.25 [0.13 ; 0.49] | 0.23 [0.06 ; 0.95] | 0.21 [0.01 ; 4.84] | 0.30 [0.05 ; 1.73] | 0.34 [0.20 ; 0.60] | 0.33 [0.07 ; 1.49] | 0.29 [0.01 ; 5.81] | 0.44 [0.13 ; 1.52] | 0.46 [0.28 ; 0.76] | 0.47 [0.23 ; 0.94] | 0.55 [0.10 ; 2.99] | 0.63 [0.04 ; 8.82] | 0.73 [0.20 ; 2.69] | 0.76 [0.14 ; 4.22] | 0.82 [0.46 ; 1.47] | 0.86 [0.38 ; 1.95] | 0.89 [0.65 ; 1.22] | 1.19 [0.13 ; 10.89] | 0.97 [0.44 ; 2.10] | High dose hydroxychloroquine | . | . | . | 0.66 [0.30 ; 1.47] | . | . |
| 0.01 [0.00 ; 0.33] | 0.07 [0.01 ; 0.42] | 0.06 [0.00 ; 1.85] | 0.06 [0.00 ; 1.90] | 0.11 [0.01 ; 1.34] | 0.14 [0.02 ; 0.99] | 0.13 [0.01 ; 1.54] | 0.11 [0.00 ; 3.84] | 0.18 [0.03 ; 1.10] | 0.16 [0.02 ; 1.50] | 0.14 [0.00 ; 5.23] | 0.21 [0.02 ; 2.43] | 0.24 [0.04 ; 1.46] | 0.23 [0.02 ; 2.27] | 0.21 [0.01 ; 6.43] | 0.31 [0.04 ; 2.46] | 0.32 [0.05 ; 1.91] | 0.33 [0.05 ; 2.09] | 0.39 [0.04 ; 4.30] | 0.44 [0.02 ; 10.26] | 0.51 [0.06 ; 4.40] | 0.53 [0.05 ; 6.00] | 0.58 [0.10 ; 3.45] | 0.60 [0.09 ; 4.12] | 0.63 [0.11 ; 3.56] | 0.83 [0.05 ; 13.70] | 0.68 [0.11 ; 4.08] | 0.70 [0.12 ; 4.11] | Lopinavir-Ritonavir plus arbidol | . | . | . | . | . |
| 0.01 [0.00 ; 0.34] | 0.06 [0.01 ; 0.46] | 0.06 [0.00 ; 1.91] | 0.06 [0.00 ; 1.97] | 0.10 [0.01 ; 1.42] | 0.13 [0.02 ; 1.08] | 0.12 [0.01 ; 1.64] | 0.11 [0.00 ; 3.97] | 0.17 [0.02 ; 1.22] | 0.16 [0.02 ; 1.61] | 0.14 [0.00 ; 5.40] | 0.20 [0.02 ; 2.59] | 0.23 [0.03 ; 1.62] | 0.22 [0.07 ; 0.73] | 0.20 [0.01 ; 6.66] | 0.30 [0.03 ; 2.77] | 0.31 [0.04 ; 2.12] | 0.32 [0.04 ; 2.30] | 0.37 [0.03 ; 4.59] | 0.42 [0.02 ; 10.70] | 0.49 [0.05 ; 4.77] | 0.51 [0.04 ; 6.41] | 0.55 [0.08 ; 3.89] | 0.58 [0.07 ; 4.52] | 0.60 [0.09 ; 3.96] | 0.80 [0.04 ; 14.43] | 0.65 [0.09 ; 4.89] | 0.67 [0.10 ; 4.56] | 0.96 [0.07 ; 12.49] | Ribavirin | . | . | . | . |
| 0.01 [0.00 ; 0.45] | 0.03 [0.00 ; 0.94] | 0.03 [0.00 ; 2.45] | 0.03 [0.00 ; 2.52] | 0.05 [0.00 ; 2.30] | 0.07 [0.00 ; 2.09] | 0.06 [0.00 ; 2.68] | 0.05 [0.00 ; 4.99] | 0.09 [0.00 ; 2.48] | 0.08 [0.00 ; 2.87] | 0.07 [0.00 ; 6.74] | 0.10 [0.00 ; 4.26] | 0.12 [0.00 ; 3.34] | 0.11 [0.00 ; 4.25] | 0.10 [0.00 ; 8.57] | 0.15 [0.00 ; 5.11] | 0.16 [0.01 ; 4.40] | 0.16 [0.01 ; 4.67] | 0.19 [0.00 ; 7.68] | 0.21 [0.00 ; 14.66] | 0.25 [0.01 ; 8.66] | 0.26 [0.01 ; 10.67] | 0.28 [0.01 ; 8.01] | 0.29 [0.01 ; 8.91] | 0.30 [0.01 ; 8.38] | 0.40 [0.01 ; 21.56] | 0.33 [0.01 ; 9.80] | 0.34 [0.01 ; 9.53] | 0.48 [0.01 ; 20.55] | 0.50 [0.01 ; 23.00] | Baloxavir | . | . | 0.47 [0.05 ; 4.48] |
| 0.01 [0.00 ; 0.20] | 0.06 [0.02 ; 0.15] | 0.05 [0.00 ; 1.13] | 0.05 [0.00 ; 1.16] | 0.10 [0.01 ; 0.68] | 0.12 [0.04 ; 0.39] | 0.12 [0.02 ; 0.78] | 0.10 [0.00 ; 2.37] | 0.16 [0.07 ; 0.39] | 0.15 [0.03 ; 0.68] | 0.13 [0.01 ; 3.25] | 0.19 [0.03 ; 1.21] | 0.22 [0.10 ; 0.49] | 0.21 [0.04 ; 1.06] | 0.19 [0.01 ; 3.91] | 0.28 [0.07 ; 1.11] | 0.29 [0.13 ; 0.63] | 0.30 [0.12 ; 0.74] | 0.35 [0.06 ; 2.10] | 0.40 [0.03 ; 5.99] | 0.46 [0.11 ; 1.94] | 0.48 [0.08 ; 2.96] | 0.52 [0.23 ; 1.20] | 0.55 [0.34 ; 0.88] | 0.57 [0.29 ; 1.11] | 0.75 [0.08 ; 7.48] | 0.61 [0.23 ; 1.63] | 0.64 [0.32 ; 1.25] | 0.91 [0.14 ; 5.84] | 0.94 [0.13 ; 7.00] | 1.87 [0.06 ; 55.45] | Hydroxychloroquine plus azithromycin | 0.37 [0.04 ; 3.58] | . |
| 0.00 [0.00 ; 0.16] | 0.02 [0.00 ; 0.26] | 0.02 [0.00 ; 0.88] | 0.02 [0.00 ; 0.91] | 0.04 [0.00 ; 0.71] | 0.05 [0.00 ; 0.59] | 0.04 [0.00 ; 0.83] | 0.04 [0.00 ; 1.82] | 0.06 [0.01 ; 0.68] | 0.05 [0.00 ; 0.84] | 0.05 [0.00 ; 2.47] | 0.07 [0.00 ; 1.31] | 0.08 [0.01 ; 0.90] | 0.08 [0.00 ; 1.26] | 0.07 [0.00 ; 3.07] | 0.10 [0.01 ; 1.47] | 0.11 [0.01 ; 1.19] | 0.11 [0.01 ; 1.27] | 0.13 [0.01 ; 2.34] | 0.15 [0.00 ; 5.05] | 0.17 [0.01 ; 2.51] | 0.18 [0.01 ; 3.26] | 0.19 [0.02 ; 2.17] | 0.20 [0.02 ; 2.05] | 0.21 [0.02 ; 2.24] | 0.28 [0.01 ; 7.03] | 0.23 [0.02 ; 2.69] | 0.23 [0.02 ; 2.51] | 0.33 [0.02 ; 6.32] | 0.35 [0.02 ; 7.21] | 0.69 [0.01 ; 40.88] | 0.37 [0.04 ; 3.58] | Lopinavir-Ritonavir plus azithromycin | . |
| 0.00 [0.00 ; 0.19] | 0.02 [0.00 ; 0.39] | 0.01 [0.00 ; 1.05] | 0.01 [0.00 ; 1.08] | 0.02 [0.00 ; 0.97] | 0.03 [0.00 ; 0.87] | 0.03 [0.00 ; 1.13] | 0.03 [0.00 ; 2.14] | 0.04 [0.00 ; 1.03] | 0.04 [0.00 ; 1.20] | 0.03 [0.00 ; 2.90] | 0.05 [0.00 ; 1.79] | 0.06 [0.00 ; 1.38] | 0.05 [0.00 ; 1.78] | 0.05 [0.00 ; 3.67] | 0.07 [0.00 ; 2.13] | 0.07 [0.00 ; 1.82] | 0.08 [0.00 ; 1.93] | 0.09 [0.00 ; 3.23] | 0.10 [0.00 ; 6.25] | 0.12 [0.00 ; 3.61] | 0.12 [0.00 ; 4.48] | 0.13 [0.01 ; 3.32] | 0.14 [0.01 ; 3.70] | 0.14 [0.01 ; 3.46] | 0.19 [0.00 ; 9.13] | 0.16 [0.01 ; 4.06] | 0.16 [0.01 ; 3.94] | 0.23 [0.01 ; 8.63] | 0.24 [0.01 ; 9.69] | 0.47 [0.05 ; 4.49] | 0.25 [0.01 ; 6.55] | 0.69 [0.01 ; 36.30] | Favipiravir |

Pairwise (upper right portion) and network (lower left portion) meta-analysis results are presented. Pharmacological agents are reported in order of treatment efficacy ranking according to SUCRAs. Comparison should be read from left to right. Effect estimation is presented in odds ratio (OR) with 95% CI and is located in intersection of two agents. OR less than 1 favors the column-defining treatment (lower admission rate to ICU or progression to ARDS). Since aggravation rate reflects better outcome, decrement of OR indicates better treatment. To obtain OR (95% CI) for comparison in the opposite direction, reciprocals should be taken.

- 1. Direct and indirect evidence proportion for each outcome


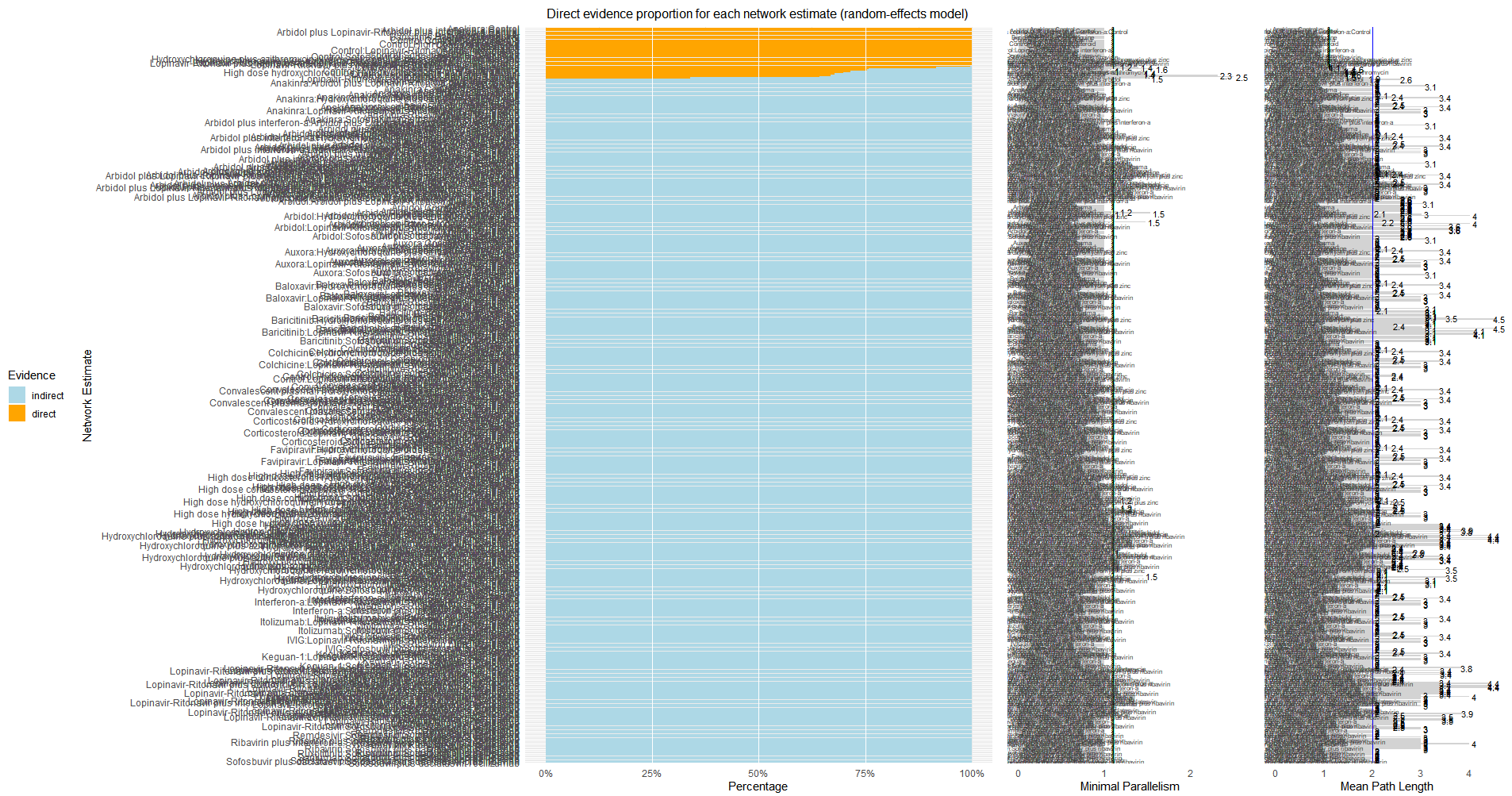


 According to König, Krahn, and Binder ([2013](https://bookdown.org/MathiasHarrer/Doing_Meta_Analysis_in_R/frequentist.html#ref-konig2013visualizing)), lower values of minimal parallelism and Mean Path Length>2 means that results for a specific comparison should be interpreted with caution.

- 1. Heterogeneity
     1. Quantifying heterogeneity: tau^2 = 0.0150; tau = 0.1226; I^2 = 3.9% [0.0%; 47.5%]
  2. Inconsistency
     1. Q statistic to assess consistency under the assumption of a full design-by-treatment interaction random effects model: Q = 3.41, p value = 0.906
  3. Net heat plot


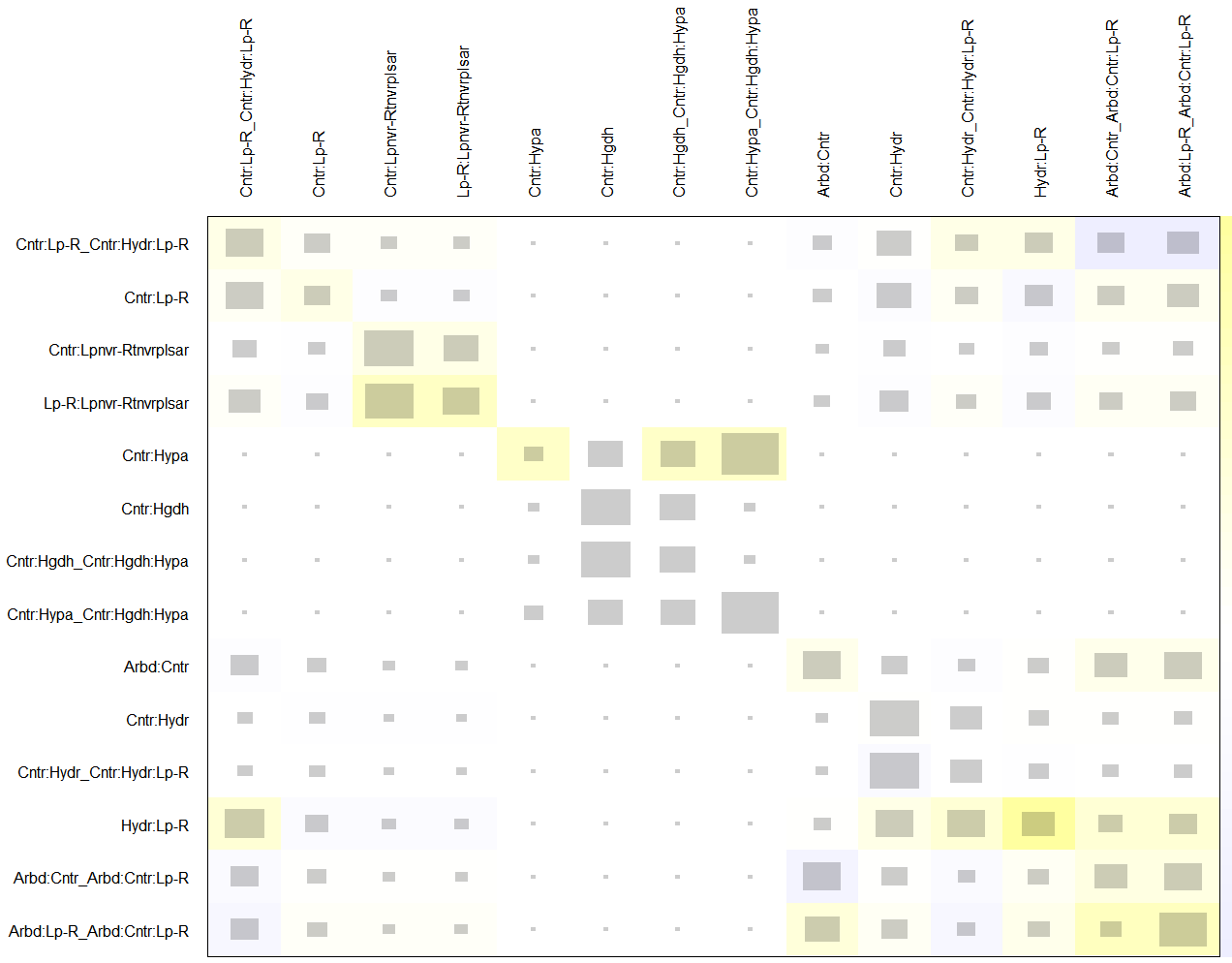


- 1. Comparison-adjusted funnel plot


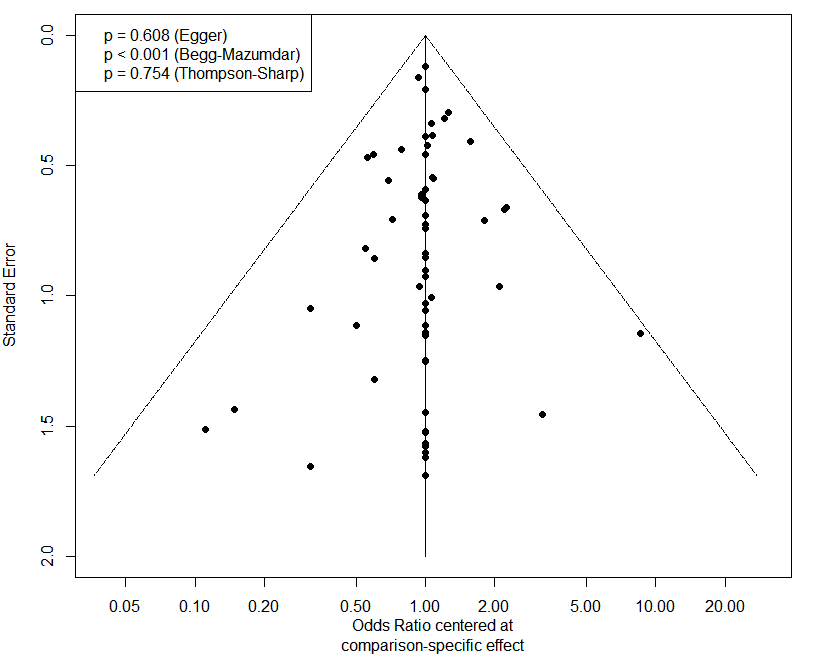


1. **Viral clearance rate (negative conversion in PCR) within 7-14 days**
   1. Network map


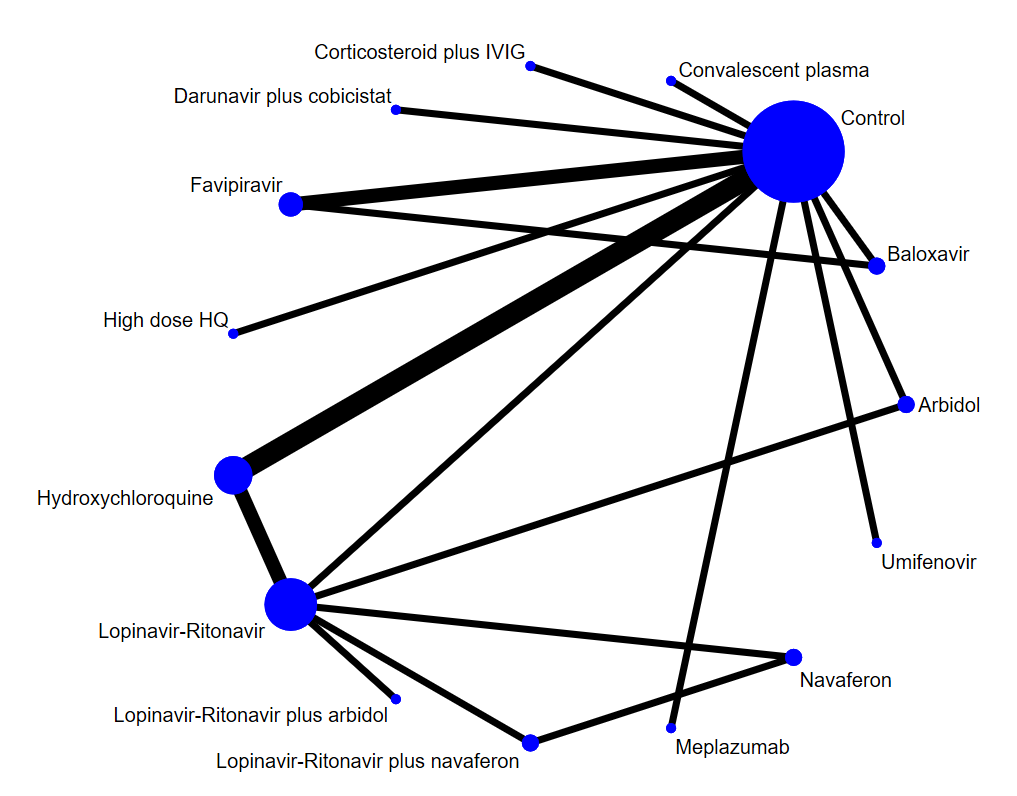


- 1. Forest plot


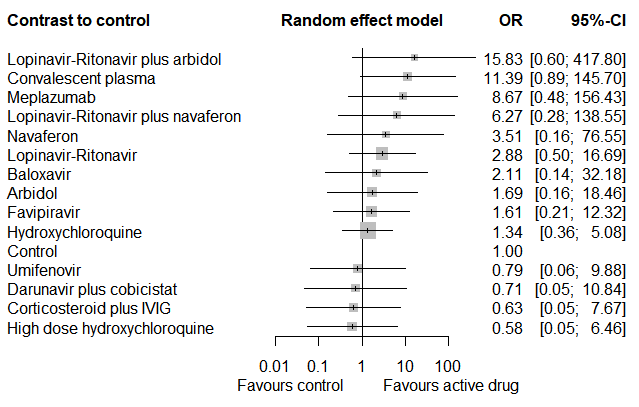


- 1. League table

| Lopinavir-Ritonavir plus arbidol | . | . | . | . | 5.50 [0.35 ; 86.99] | . | . | . | . | . | . | . | . | . |
| --- | --- | --- | --- | --- | --- | --- | --- | --- | --- | --- | --- | --- | --- | --- |
| 1.39 [0.02 ; 88.05] | Convalescent plasma | . | . | . | . | . | . | . | . | 11.39 [0.89 ;145.70] | . | . | . | . |
| 1.83 [0.02 ; 144.15] | 1.31 [0.03 ; 62.11] | Meplazumab | . | . | . | . | . | . | . | 8.67 [0.48 ;156.43] | . | . | . | . |
| 2.53 [0.06 ; 108.17] | 1.82 [0.03 ; 100.19] | 1.38 [0.02 ; 95.69] | Lopinavir-Ritonavir plus navaferon | 1.78 [0.14 ; 22.77] | 2.18 [0.17 ; 27.84] | . | . | . | . | . | . | . | . | . |
| 4.51 [0.11 ; 190.75] | 3.24 [0.06 ; 176.81] | 2.47 [0.04 ; 168.96] | 1.78 [0.14 ; 22.77] | Navaferon | 1.22 [0.10 ; 15.34] | . | . | . | . | . | . | . | . | . |
| 5.50 [0.35 ; 86.99] | 3.96 [0.18 ; 87.50] | 3.01 [0.10 ; 88.89] | 2.18 [0.17 ; 27.84] | 1.22 [0.10 ; 15.34] | Lopinavir-Ritonavir | . | 0.92 [0.07 ; 11.40] | . | 4.24 [0.65 ; 27.85] | 0.78 [0.06 ; 10.53] | . | . | . | . |
| 7.49 [0.11 ; 529.20] | 5.39 [0.13 ; 224.58] | 4.10 [0.08 ; 217.95] | 2.97 [0.05 ; 183.12] | 1.66 [0.03 ; 101.54] | 1.36 [0.05 ; 34.81] | Baloxavir | . | 1.87 [0.10 ; 35.64] | . | 1.50 [0.08 ; 27.61] | . | . | . | . |
| 9.35 [0.25 ; 350.73] | 6.73 [0.20 ; 221.51] | 5.12 [0.12 ; 218.28] | 3.70 [0.12 ; 118.42] | 2.08 [0.07 ; 65.53] | 1.70 [0.16 ; 17.79] | 1.25 [0.03 ; 46.76] | Arbidol | . | . | 0.84 [0.06 ; 11.36] | . | . | . | . |
| 9.81 [0.21 ; 462.09] | 7.05 [0.27 ; 183.78] | 5.37 [0.16 ; 184.23] | 3.88 [0.10 ; 157.57] | 2.18 [0.05 ; 87.26] | 1.78 [0.12 ; 26.19] | 1.31 [0.08 ; 20.27] | 1.05 [0.05 ; 24.15] | Favipiravir | . | 1.61 [0.21 ; 12.32] | . | . | . | . |
| 11.78 [0.49 ; 285.38] | 8.48 [0.48 ; 150.19] | 6.45 [0.27 ; 155.69] | 4.67 [0.23 ; 94.16] | 2.61 [0.13 ; 52.00] | 2.14 [0.44 ; 10.53] | 1.57 [0.08 ; 32.55] | 1.26 [0.10 ; 15.32] | 1.20 [0.11 ; 13.63] | Hydroxychloroquine | 2.03 [0.47 ; 8.72] | . | . | . | . |
| 15.83 [0.60 ; 417.80] | 11.39 [0.89 ; 145.70] | 8.67 [0.48 ; 156.43] | 6.27 [0.28 ; 138.55] | 3.51 [0.16 ; 76.55] | 2.88 [0.50 ; 16.69] | 2.11 [0.14 ; 32.18] | 1.69 [0.16 ; 18.46] | 1.61 [0.21 ; 12.32] | 1.34 [0.36 ; 5.08] | Control | 1.27 [0.10 ; 16.00] | 1.42 [0.09 ; 21.75] | 1.59 [0.13 ; 19.47] | 1.71 [0.15 ; 18.90] |
| 20.15 [0.32 ;1262.51] | 14.49 [0.40 ; 526.44] | 11.03 [0.24 ; 515.36] | 7.98 [0.15 ; 435.11] | 4.47 [0.08 ; 241.18] | 3.66 [0.17 ; 79.85] | 2.69 [0.07 ; 110.75] | 2.15 [0.07 ; 70.00] | 2.05 [0.08 ; 52.80] | 1.71 [0.10 ; 29.84] | 1.27 [0.10 ; 16.00] | Umifenovir | . | . | . |
| 22.43 [0.32 ;1592.83] | 16.14 [0.38 ; 676.47] | 12.28 [0.23 ; 656.24] | 8.88 [0.14 ; 551.28] | 4.98 [0.08 ; 305.68] | 4.08 [0.16 ; 104.96] | 2.99 [0.06 ; 141.65] | 2.40 [0.06 ; 90.34] | 2.29 [0.08 ; 68.84] | 1.90 [0.09 ; 39.70] | 1.42 [0.09 ; 21.75] | 1.11 [0.03 ; 46.11] | Darunavir plus cobicistat | . | . |
| 25.23 [0.41 ;1553.63] | 18.15 [0.51 ; 646.13] | 13.81 [0.30 ; 633.36] | 9.99 [0.19 ; 535.13] | 5.60 [0.11 ; 296.61] | 4.59 [0.22 ; 97.68] | 3.37 [0.08 ; 136.02] | 2.70 [0.08 ; 85.85] | 2.57 [0.10 ; 64.66] | 2.14 [0.13 ; 36.43] | 1.59 [0.13 ; 19.47] | 1.25 [0.04 ; 44.02] | 1.12 [0.03 ; 45.71] | Corticosteroid plus IVIG | . |
| 27.07 [0.47 ;1569.63] | 19.48 [0.59 ; 646.65] | 14.82 [0.34 ; 636.86] | 10.72 [0.21 ; 539.47] | 6.01 [0.12 ; 298.96] | 4.92 [0.25 ; 96.59] | 3.61 [0.10 ; 136.46] | 2.89 [0.10 ; 85.73] | 2.76 [0.12 ; 64.22] | 2.30 [0.15 ; 35.78] | 1.71 [0.15 ; 18.90] | 1.34 [0.04 ; 44.04] | 1.21 [0.03 ; 45.86] | 1.07 [0.03 ; 34.46] | High dose hydroxychloroquine |

Pairwise (upper right portion) and network (lower left portion) meta-analysis results are presented. Pharmacological agents are reported in order of treatment efficacy ranking according to SUCRAs. Comparison should be read from left to right. Effect estimation is presented in odds ratio (OR) with 95% CI and is located in intersection of two agents. OR greater than 1 favors the column-defining treatment (higher viral clearance rate withing 14 days). Since higher viral clearance rate reflects better outcome, increment of OR indicates better treatment. To obtain OR (95% CI) for comparison in the opposite direction, reciprocals should be taken.

- 1. Direct and indirect evidence proportion for each outcome


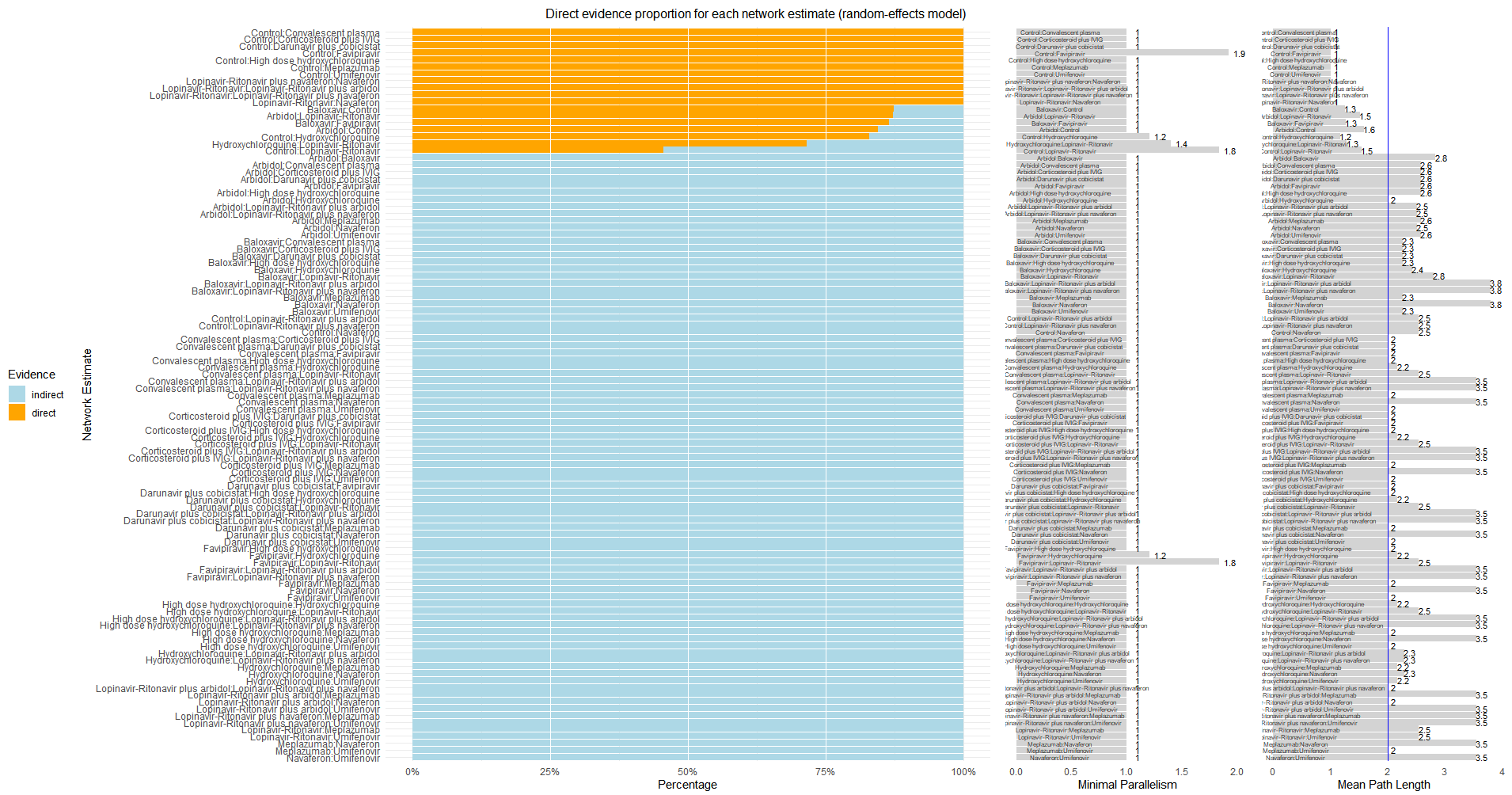


 According to König, Krahn, and Binder ([2013](https://bookdown.org/MathiasHarrer/Doing_Meta_Analysis_in_R/frequentist.html#ref-konig2013visualizing)), lower values of minimal parallelism and Mean Path Length>2 means that results for a specific comparison should be interpreted with caution.

- 1. Heterogeneity
     1. Quantifying heterogeneity: tau^2 = 1.3937; tau = 1.1805; I^2 = 83.8% [66.1%; 92.2%]
  2. Inconsistency
     1. Q statistic to assess consistency under the assumption of a full design-by-treatment interaction random effects model: Q = 2.17, p value = 0.3384
  3. Net heat plot


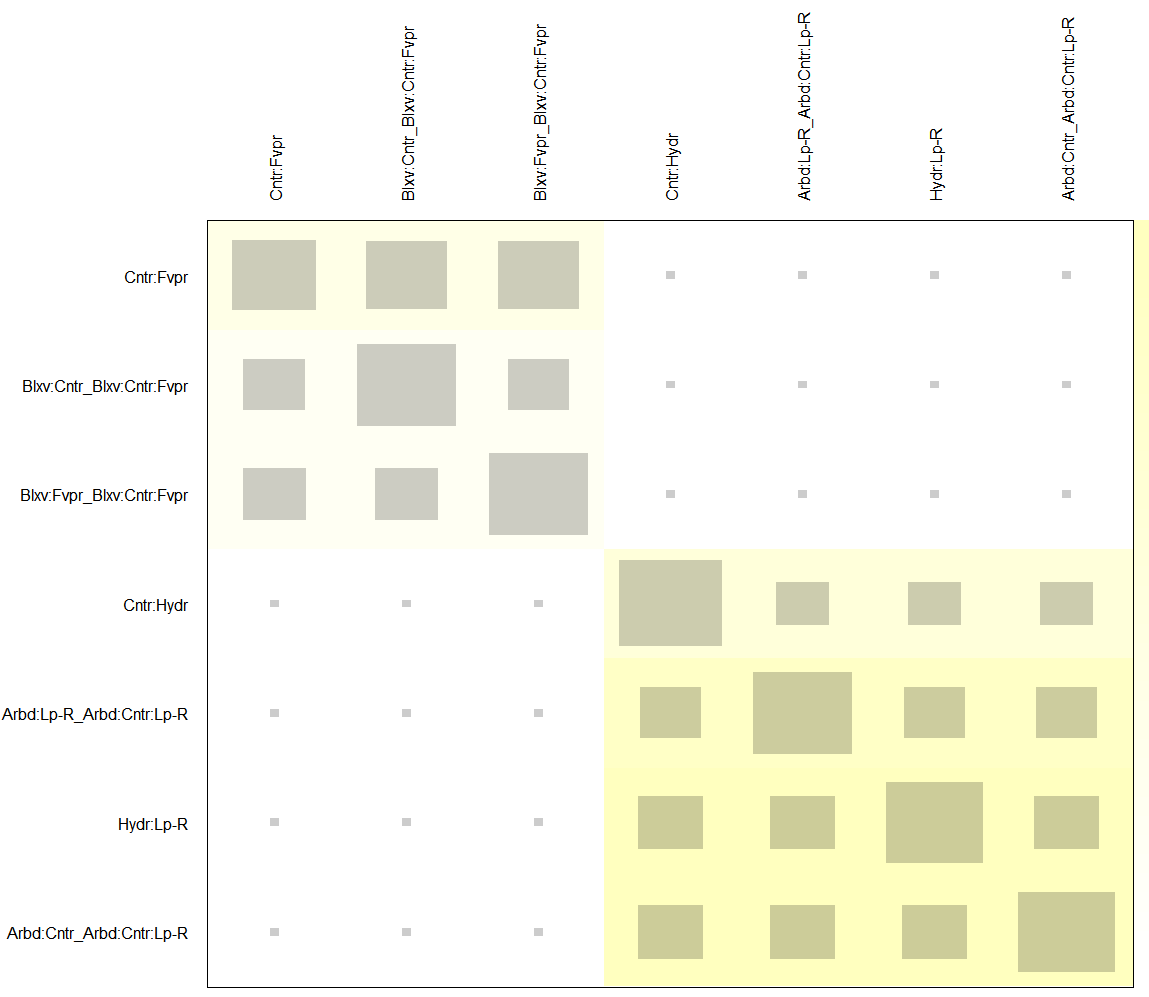


- 1. Comparison-adjusted funnel plot


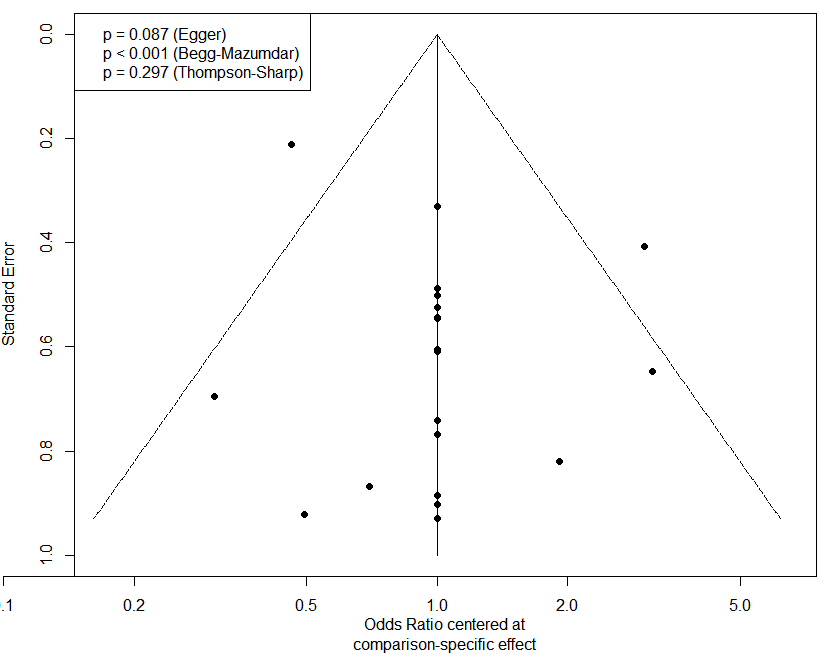


1. **Time to viral clearance (days)**
   1. Network map


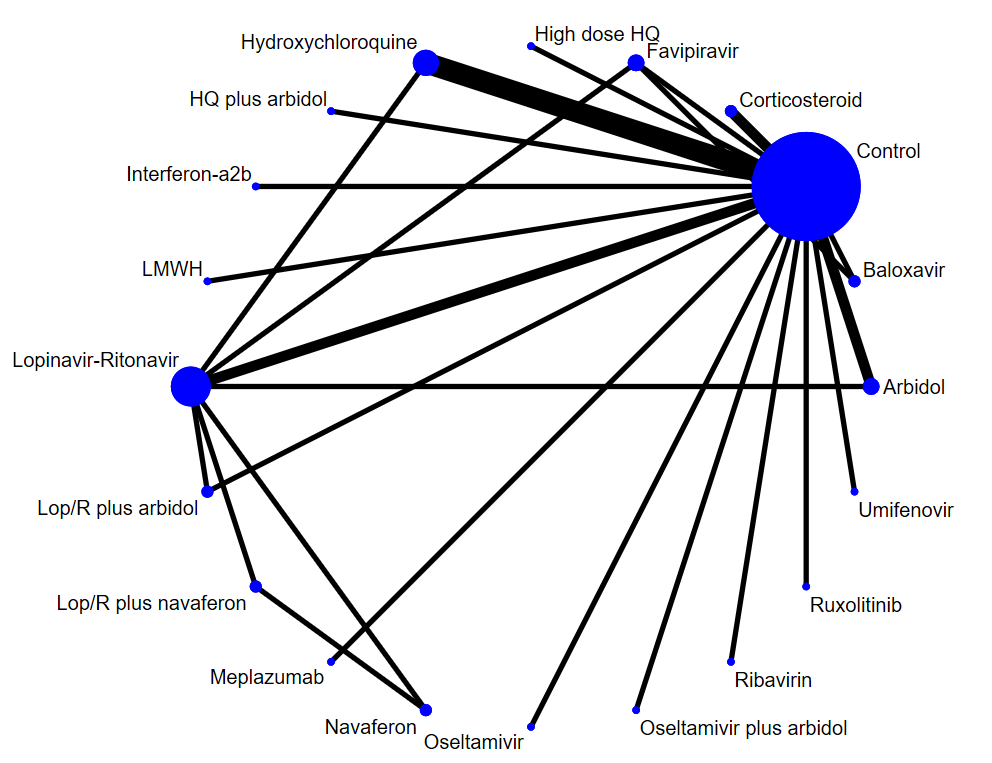


- 1. Forest plot


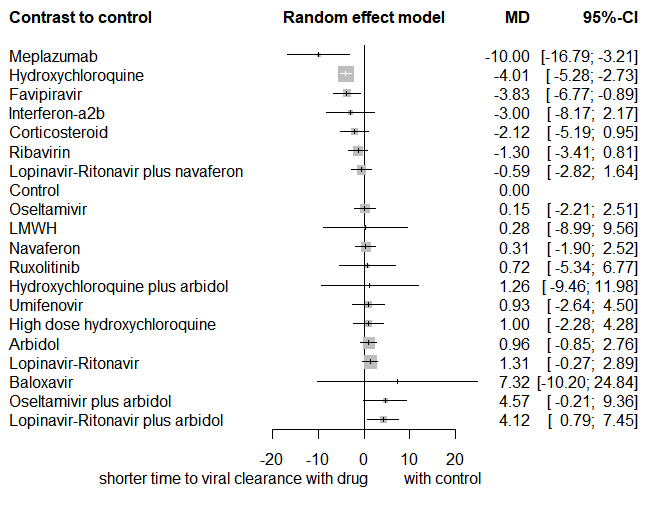


- 1. League table

| Meplazumab | . | . | . | . | . | . | -10.00 [-16.79 ;-3.21] | . | . | . | . | . | . | . | . | . | . | . | . |
| --- | --- | --- | --- | --- | --- | --- | --- | --- | --- | --- | --- | --- | --- | --- | --- | --- | --- | --- | --- |
| -5.99 [-12.90 ; 0.91] | Hydroxychloroquine | . | . | . | . | . | -4.18 [ -5.53 ;-2.84] | . | . | . | . | . | . | . | . | -3.97 [ -7.66 ;-0.28] | . | . | . |
| -6.17 [-13.56 ; 1.22] | -0.18 [ -3.29 ; 2.94] | Favipiravir | . | . | . | . | 8.17 [-11.26 ;27.60] | . | . | . | . | . | . | . | . | -5.40 [ -7.92 ;-2.88] | -3.79 [-31.92 ;24.35] | . | . |
| -7.00 [-15.53 ; 1.53] | -1.01 [ -6.34 ; 4.32] | -0.83 [ -6.78 ; 5.12] | Interferon-a2b | . | . | . | -3.00 [ -8.17 ; 2.17] | . | . | . | . | . | . | . | . | . | . | . | . |
| -7.88 [-15.33 ;-0.43] | -1.89 [ -5.21 ; 1.44] | -1.71 [ -5.96 ; 2.54] | -0.88 [ -6.90 ; 5.13] | Corticosteroid | . | . | -2.12 [ -5.19 ; 0.95] | . | . | . | . | . | . | . | . | . | . | . | . |
| -8.70 [-15.81 ;-1.59] | -2.71 [ -5.18 ;-0.23] | -2.53 [ -6.15 ; 1.09] | -1.70 [ -7.29 ; 3.89] | -0.82 [ -4.55 ; 2.91] | Ribavirin | . | -1.30 [ -3.41 ; 0.81] | . | . | . | . | . | . | . | . | . | . | . | . |
| -9.41 [-16.55 ;-2.27] | -3.42 [ -5.87 ;-0.96] | -3.24 [ -6.19 ;-0.29] | -2.41 [ -8.05 ; 3.22] | -1.53 [ -5.32 ; 2.27] | -0.71 [ -3.78 ; 2.36] | Lopinavir-Ritonavir plus navaferon | . | . | . | -0.90 [ -2.86 ; 1.06] | . | . | . | . | . | -1.90 [ -3.83 ; 0.03] | . | . | . |
| -10.00 [-16.79 ;-3.21] | -4.01 [ -5.28 ;-2.73] | -3.83 [ -6.77 ;-0.89] | -3.00 [ -8.17 ; 2.17] | -2.12 [ -5.19 ; 0.95] | -1.30 [ -3.41 ; 0.81] | -0.59 [ -2.82 ; 1.64] | Control | -0.15 [ -2.51 ; 2.21] | -0.28 [ -9.56 ; 8.99] | . | -0.72 [ -6.77 ; 5.34] | -1.26 [-11.98 ; 9.46] | -0.93 [ -4.50 ; 2.64] | -1.00 [ -4.28 ; 2.28] | -0.66 [ -2.84 ; 1.51] | -1.52 [ -3.63 ; 0.59] | -11.95 [-34.27 ;10.36] | -4.57 [ -9.36 ; 0.21] | -6.02 [-11.12 ;-0.92] |
| -10.15 [-17.33 ;-2.96] | -4.15 [ -6.84 ;-1.47] | -3.98 [ -7.74 ;-0.21] | -3.15 [ -8.83 ; 2.54] | -2.27 [ -6.14 ; 1.60] | -1.45 [ -4.61 ; 1.72] | -0.74 [ -3.98 ; 2.51] | -0.15 [ -2.51 ; 2.21] | Oseltamivir | . | . | . | . | . | . | . | . | . | . | . |
| -10.28 [-21.77 ; 1.21] | -4.29 [-13.65 ; 5.08] | -4.11 [-13.84 ; 5.62] | -3.28 [-13.90 ; 7.34] | -2.40 [-12.17 ; 7.37] | -1.58 [-11.10 ; 7.93] | -0.87 [-10.41 ; 8.67] | -0.28 [ -9.56 ; 8.99] | -0.13 [ -9.71 ; 9.44] | LMWH | . | . | . | . | . | . | . | . | . | . |
| -10.31 [-17.45 ;-3.17] | -4.32 [ -6.75 ;-1.88] | -4.14 [ -7.08 ;-1.20] | -3.31 [ -8.94 ; 2.32] | -2.43 [ -6.21 ; 1.35] | -1.61 [ -4.67 ; 1.45] | -0.90 [ -2.49 ; 0.69] | -0.31 [ -2.52 ; 1.90] | -0.16 [ -3.40 ; 3.07] | -0.03 [ -9.57 ; 9.51] | Navaferon | . | . | . | . | . | -1.00 [ -2.87 ; 0.87] | . | . | . |
| -10.72 [-19.81 ;-1.62] | -4.72 [-10.91 ; 1.47] | -4.55 [-11.28 ; 2.18] | -3.72 [-11.68 ; 4.25] | -2.83 [ -9.62 ; 3.95] | -2.02 [ -8.43 ; 4.40] | -1.31 [ -7.76 ; 5.15] | -0.72 [ -6.77 ; 5.34] | -0.57 [ -7.07 ; 5.93] | -0.43 [-11.51 ;10.64] | -0.41 [ -6.85 ; 6.04] | Ruxolitinib | . | . | . | . | . | . | . | . |
| -11.26 [-23.94 ; 1.43] | -5.26 [-16.06 ; 5.53] | -5.09 [-16.20 ; 6.03] | -4.26 [-16.16 ; 7.64] | -3.38 [-14.53 ; 7.77] | -2.56 [-13.48 ; 8.37] | -1.85 [-12.80 ; 9.10] | -1.26 [-11.98 ; 9.46] | -1.11 [-12.09 ; 9.86] | -0.98 [-15.15 ;13.20] | -0.95 [-11.89 ;10.00] | -0.54 [-12.85 ;11.77] | Hydroxychloroquine plus arbidol | . | . | . | . | . | . | . |
| -10.93 [-18.60 ;-3.26] | -4.94 [ -8.73 ;-1.14] | -4.76 [ -9.38 ;-0.14] | -3.93 [-10.22 ; 2.36] | -3.05 [ -7.76 ; 1.66] | -2.23 [ -6.38 ; 1.92] | -1.52 [ -5.73 ; 2.69] | -0.93 [ -4.50 ; 2.64] | -0.78 [ -5.06 ; 3.50] | -0.65 [-10.59 ; 9.29] | -0.62 [ -4.82 ; 3.58] | -0.21 [ -7.24 ; 6.82] | 0.33 [-10.97 ;11.63] | Umifenovir | . | . | . | . | . | . |
| -11.00 [-18.54 ;-3.46] | -5.01 [ -8.53 ;-1.48] | -4.83 [ -9.23 ;-0.42] | -4.00 [-10.13 ; 2.13] | -3.12 [ -7.61 ; 1.38] | -2.30 [ -6.21 ; 1.61] | -1.59 [ -5.56 ; 2.38] | -1.00 [ -4.28 ; 2.28] | -0.85 [ -4.90 ; 3.19] | -0.72 [-10.56 ; 9.12] | -0.69 [ -4.65 ; 3.27] | -0.28 [ -7.17 ; 6.60] | 0.26 [-10.95 ;11.47] | -0.07 [ -4.92 ; 4.78] | High dose hydroxychloroquine | . | . | . | . | . |
| -10.96 [-17.98 ;-3.94] | -4.96 [ -7.12 ;-2.80] | -4.79 [ -7.94 ;-1.63] | -3.96 [ -9.44 ; 1.52] | -3.08 [ -6.64 ; 0.49] | -2.26 [ -5.04 ; 0.52] | -1.55 [ -4.05 ; 0.96] | -0.96 [ -2.76 ; 0.85] | -0.81 [ -3.78 ; 2.16] | -0.68 [-10.13 ; 8.78] | -0.65 [ -3.13 ; 1.84] | -0.24 [ -6.56 ; 6.08] | 0.30 [-10.57 ;11.17] | -0.03 [ -4.03 ; 3.97] | 0.04 [ -3.70 ; 3.79] | Arbidol | 0.10 [ -2.60 ; 2.80] | . | . | . |
| -11.31 [-18.28 ;-4.34] | -5.32 [ -7.20 ;-3.43] | -5.14 [ -7.64 ;-2.64] | -4.31 [ -9.72 ; 1.10] | -3.43 [ -6.88 ; 0.02] | -2.61 [ -5.25 ; 0.03] | -1.90 [ -3.47 ;-0.33] | -1.31 [ -2.89 ; 0.27] | -1.16 [ -4.00 ; 1.68] | -1.03 [-10.44 ; 8.38] | -1.00 [ -2.55 ; 0.55] | -0.59 [ -6.85 ; 5.67] | -0.05 [-10.89 ;10.78] | -0.38 [ -4.29 ; 3.53] | -0.31 [ -3.96 ; 3.33] | -0.35 [ -2.30 ; 1.59] | Lopinavir-Ritonavir | . | . | -1.60 [ -5.68 ; 2.48] |
| -17.32 [-36.11 ; 1.47] | -11.33 [-28.88 ; 6.23] | -11.15 [-28.72 ; 6.42] | -10.32 [-28.59 ; 7.95] | -9.44 [-27.22 ; 8.35] | -8.62 [-26.26 ; 9.02] | -7.91 [-25.52 ; 9.70] | -7.32 [-24.84 ;10.20] | -7.17 [-24.85 ;10.50] | -7.04 [-26.86 ;12.78] | -7.01 [-24.61 ;10.59] | -6.60 [-25.14 ;11.93] | -6.06 [-26.60 ;14.48] | -6.39 [-24.27 ;11.49] | -6.32 [-24.14 ;11.50] | -6.36 [-23.95 ;11.23] | -6.01 [-23.54 ;11.53] | Baloxavir | . | . |
| -14.57 [-22.87 ;-6.27] | -8.58 [-13.53 ;-3.63] | -8.40 [-14.02 ;-2.79] | -7.57 [-14.62 ;-0.53] | -6.69 [-12.37 ;-1.01] | -5.87 [-11.10 ;-0.64] | -5.16 [-10.44 ; 0.12] | -4.57 [ -9.36 ; 0.21] | -4.43 [ -9.76 ; 0.91] | -4.29 [-14.73 ; 6.15] | -4.26 [ -9.53 ; 1.01] | -3.86 [-11.57 ; 3.86] | -3.31 [-15.05 ; 8.42] | -3.64 [ -9.61 ; 2.33] | -3.57 [ -9.37 ; 2.23] | -3.62 [ -8.73 ; 1.50] | -3.26 [ -8.30 ; 1.78] | 2.75 [-15.41 ;20.91] | Oseltamivir plus arbidol | . |
| -14.12 [-21.68 ;-6.56] | -8.13 [-11.65 ;-4.61] | -7.95 [-12.04 ;-3.86] | -7.12 [-13.28 ;-0.97] | -6.24 [-10.77 ;-1.71] | -5.42 [ -9.37 ;-1.48] | -4.71 [ -8.32 ;-1.11] | -4.12 [ -7.45 ;-0.79] | -3.98 [ -8.06 ; 0.10] | -3.84 [-13.70 ; 6.01] | -3.81 [ -7.41 ;-0.22] | -3.41 [-10.32 ; 3.50] | -2.87 [-14.09 ; 8.36] | -3.19 [ -8.08 ; 1.69] | -3.12 [ -7.80 ; 1.55] | -3.17 [ -6.79 ; 0.46] | -2.81 [ -6.06 ; 0.43] | 3.20 [-14.60 ;21.00] | 0.45 [ -5.38 ; 6.28] | Lopinavir-Ritonavir plus arbidol |

Pairwise (upper right portion) and network (lower left portion) meta-analysis results are presented. Pharmacological agents are reported in order of treatment efficacy ranking according to SUCRAs. Comparison should be read from left to right. Effect estimation is presented in mean difference (MD) with 95% CI and is located in intersection of two agents. MD less than 0 favors the column-defining treatment (shorter time to viral clearance compared to control). Since shorter time to viral clearance reflects better outcome, decrement of MD indicates better treatment. To obtain MD (95% CI) for comparison in the opposite direction, negative values should be converted into positive value and vice versa.

- 1. Direct and indirect evidence proportion for each outcome


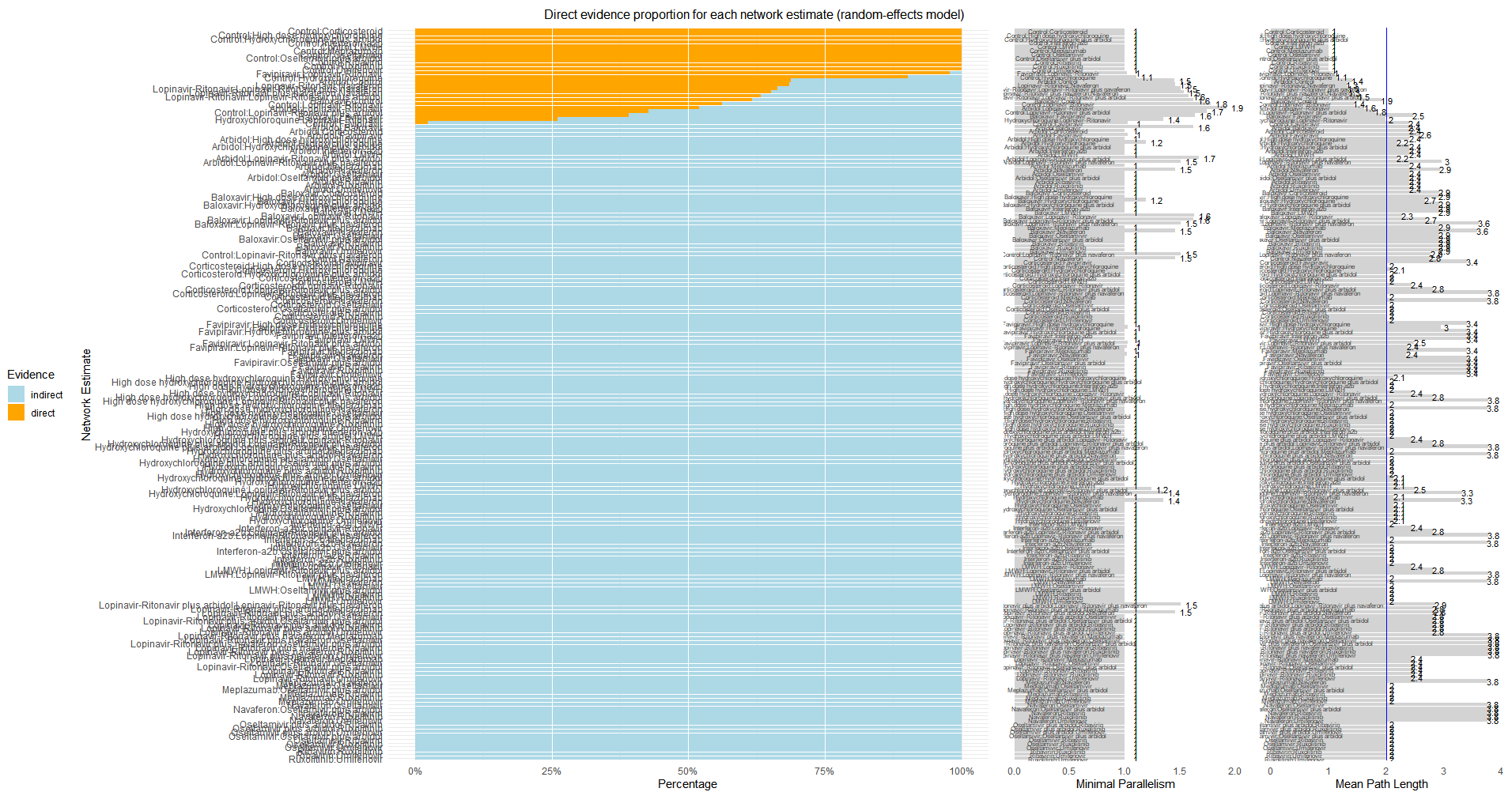


 According to König, Krahn, and Binder ([2013](https://bookdown.org/MathiasHarrer/Doing_Meta_Analysis_in_R/frequentist.html#ref-konig2013visualizing)), lower values of minimal parallelism and Mean Path Length>2 means that results for a specific comparison should be interpreted with caution.

- 1. Heterogeneity
     1. Quantifying heterogeneity: tau^2 = 0.6088; tau = 0.7803; I^2 = 27.4% [0.0%; 62.4%]
  2. Inconsistency
     1. Q statistic to assess consistency under the assumption of a full design-by-treatment interaction random effects model: Q = 2.93, p value = 0.8171
  3. Net heat plot


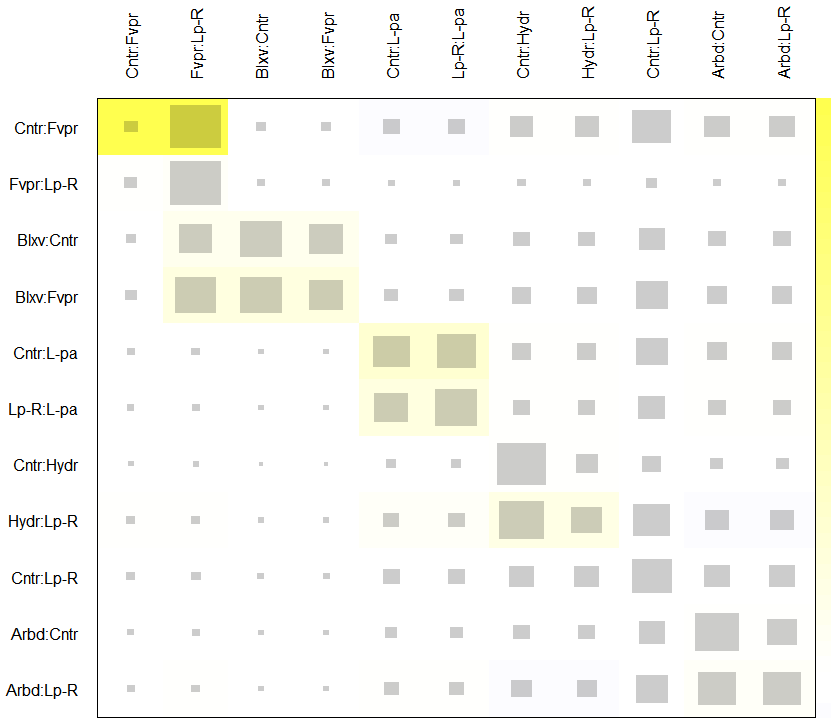


- 1. Comparison-adjusted funnel plot


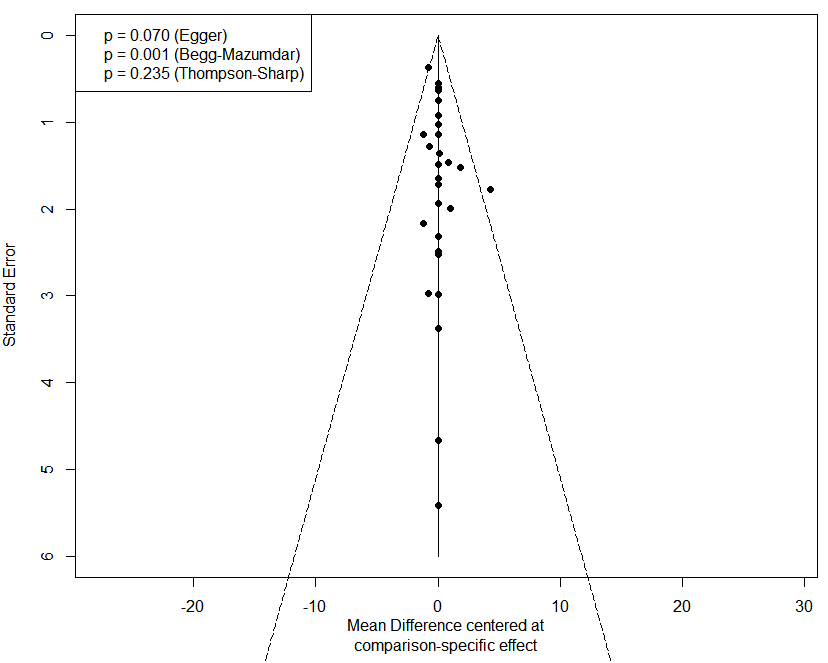


Safety outcomes: safety

1. **Δ QTc interval (msec) of HQ+AZ and azithromycin compared to ΔQTc of control**
2. **Proportion of patients experienced QTc prolongation (>500ms or delta >60ms)**
3. **Fatal cardiac complications after HQ
   (TdP, ventricular tachycardia, and severe ventricular arrhythmia)**
4. **Non-cardiac serious adverse event**
5. **Δ QTc interval (msec) of HQ+AZ and azithromycin compared to ΔQTc of control**
   1. Network map


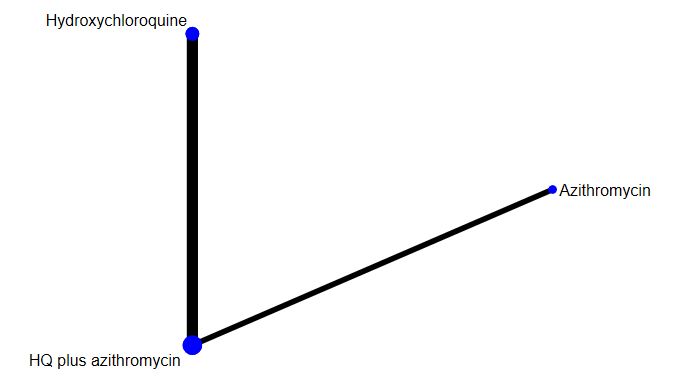


- 1. Forest plot


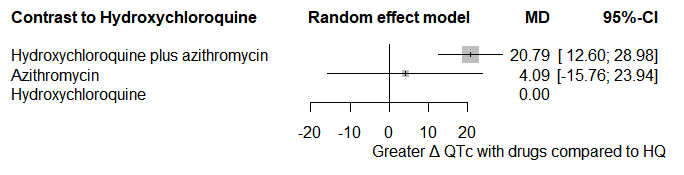


- 1. League table

| Hydroxychloroquine | . | -20.79 [-28.98 ;-12.60] |
| --- | --- | --- |
| -4.09 [-23.94 ; 15.76] | Azithromycin | -16.70 [-34.78 ; 1.38] |
| -20.79 [-28.98 ;-12.60] | -16.70 [-34.78 ; 1.38] | Hydroxychloroquine plus azithromycin |

Pairwise (upper right portion) and network (lower left portion) meta-analysis results are presented. Pharmacological agents are reported in order of treatment efficacy (ICU) ranking according to SUCRAs. Comparison should be read from left to right. Effect estimation is presented in mean difference (MD) with 95% CI and is located in intersection of two agents. MD less than 0 favors the column-defining treatment (smaller delta QTc compared to control). Since smaller delta QTc reflects better outcome, decrement of MD indicates better treatment. To obtain MD (95% CI) for comparison in the opposite direction, negative values should be converted into positive value and vice versa

- 1. Direct and indirect evidence proportion for each outcome


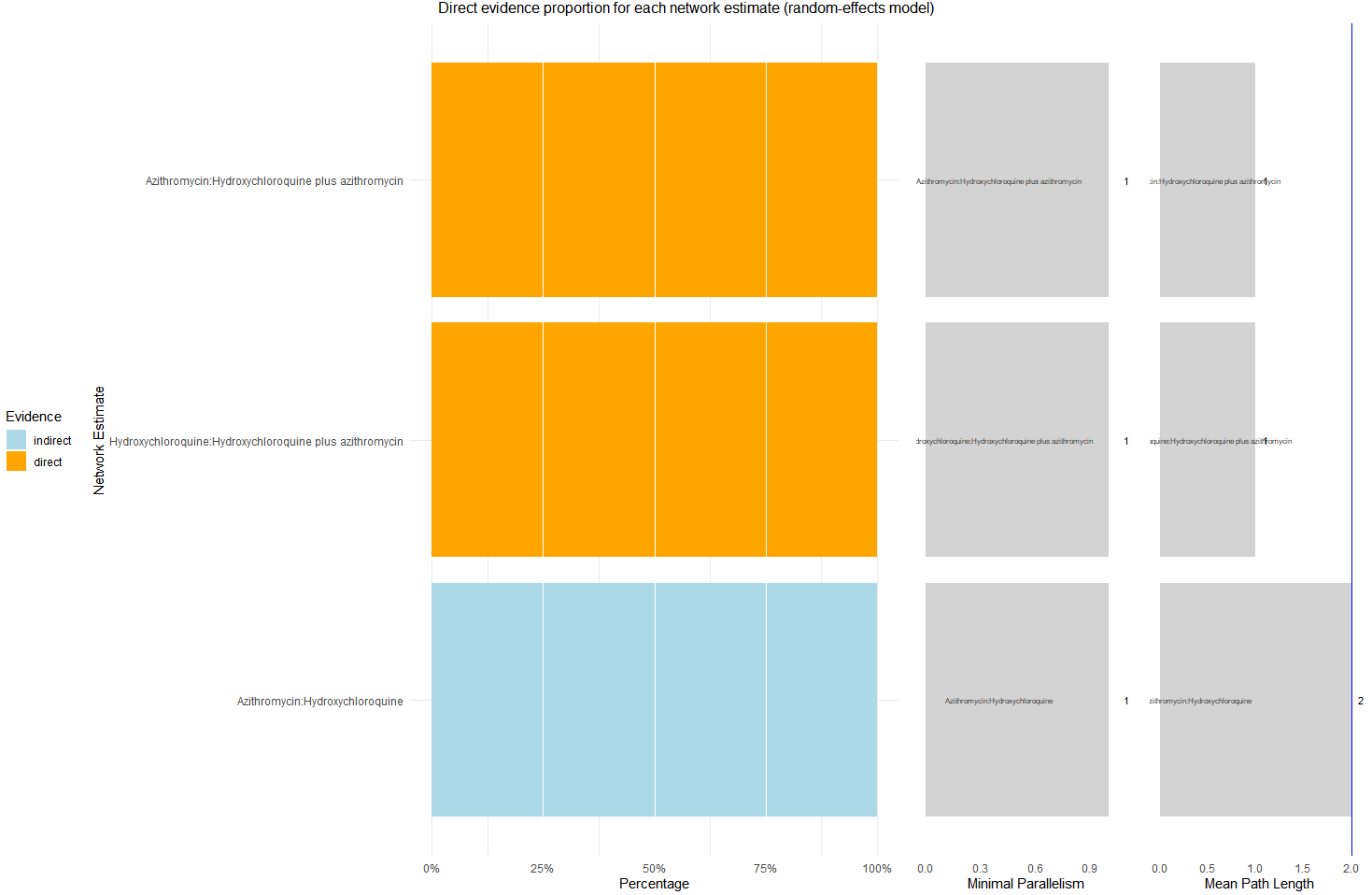


 According to König, Krahn, and Binder ([2013](https://bookdown.org/MathiasHarrer/Doing_Meta_Analysis_in_R/frequentist.html#ref-konig2013visualizing)), lower values of minimal parallelism and Mean Path Length>2 means that results for a specific comparison should be interpreted with caution.

- 1. Heterogeneity
     1. Quantifying heterogeneity: tau^2 = 0; tau = 0; I^2 = 0%
  2. Inconsistency
     1. Q statistic to assess consistency under the assumption of a full design-by-treatment interaction random effects model: p value = 1.000
  3. Net heat plot
     1. Net heat plot not available due to small number of designs
  4. Comparison-adjusted funnel plot


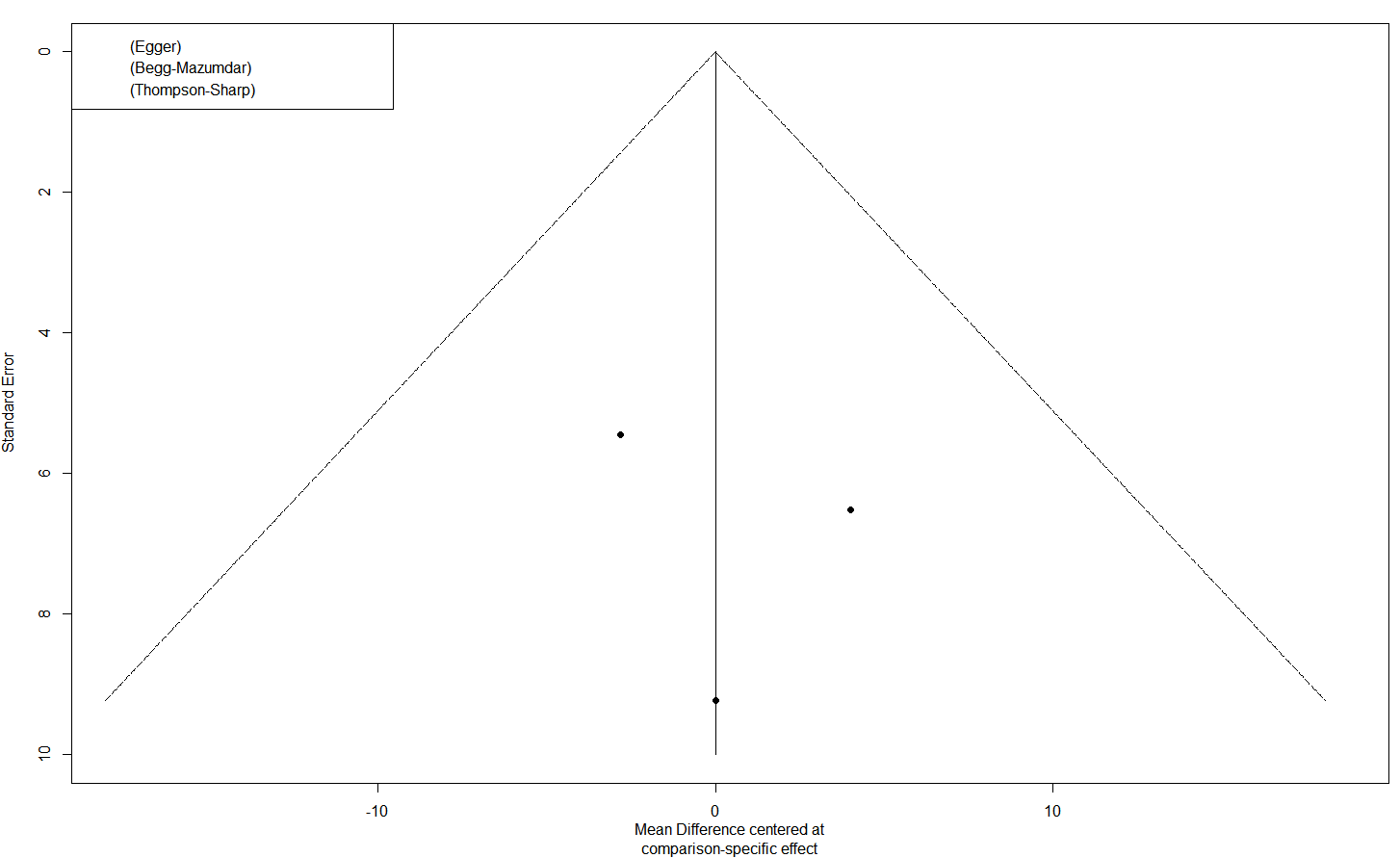


1. **Proportion of patients experienced QTc prolongation (>500ms or delta >60ms)**
   1. Network map


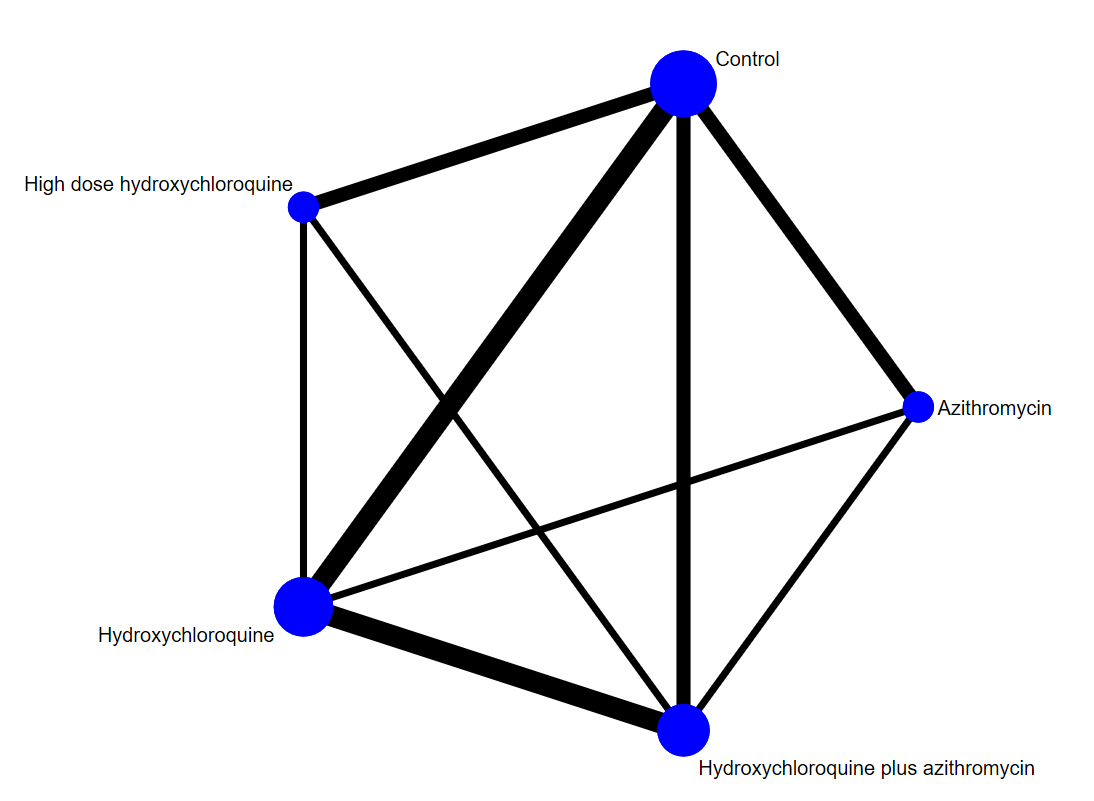


- 1. Forest plot


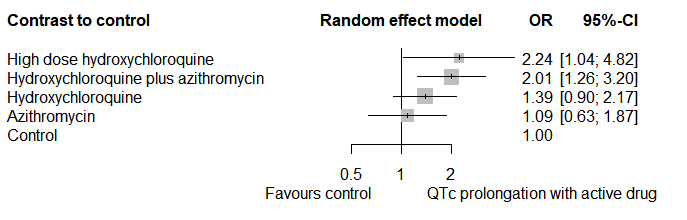


- 1. League table

| Control | 0.99 [0.48 ; 2.08] | 0.60 [0.34 ; 1.07] | 0.55 [0.30 ; 1.00] | 0.22 [0.04 ; 1.17] |
| --- | --- | --- | --- | --- |
| 0.92 [0.53 ;1.58] | Azithromycin | 0.63 [0.29 ; 1.37] | 1.34 [0.35 ; 5.18] | . |
| 0.72 [0.46 ;1.11] | 0.78 [0.45 ;1.35] | Hydroxychloroquine | 0.46 [0.22 ; 0.94] | 0.54 [0.14 ; 2.08] |
| 0.50 [0.31 ;0.79] | 0.54 [0.29 ;1.00] | 0.69 [0.43 ;1.13] | Hydroxychloroquine plus azithromycin | 1.00 [0.44 ; 2.30] |
| 0.45 [0.21 ;0.96] | 0.49 [0.20 ;1.16] | 0.62 [0.29 ;1.33] | 0.90 [0.45 ;1.80] | High dose hydroxychloroquine |

Pairwise (upper right portion) and network (lower left portion) meta-analysis results are presented. Pharmacological agents are reported in order of treatment efficacy (ICU) ranking according to SUCRAs. Comparison should be read from left to right. Effect estimation is presented in odds ratio (OR) with 95% CI and is located in intersection of two agents. OR less than 1 favors the column-defining treatment (lower QT prolongation occurrence rate). Since lower QT prolongation rate reflects better outcome, decrement of OR indicates better treatment. To obtain OR (95% CI) for comparison in the opposite direction, reciprocals should be taken

- 1. Direct and indirect evidence proportion for each outcome


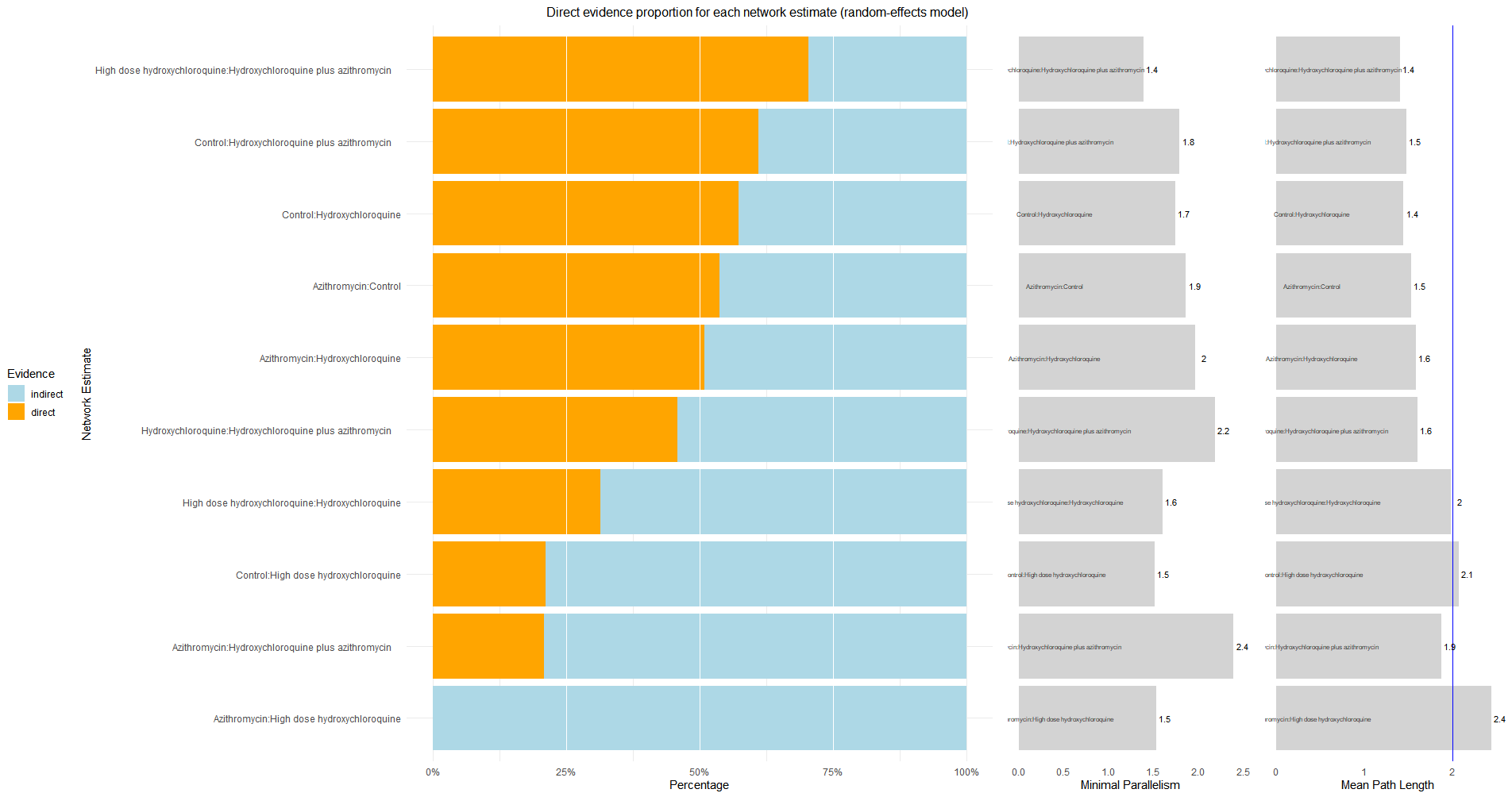


 According to König, Krahn, and Binder ([2013](https://bookdown.org/MathiasHarrer/Doing_Meta_Analysis_in_R/frequentist.html#ref-konig2013visualizing)), lower values of minimal parallelism and Mean Path Length>2 means that results for a specific comparison should be interpreted with caution.

- 1. Heterogeneity
     1. Quantifying heterogeneity: tau^2 = 0.0208; tau = 0.1442; I^2 = 5.8% [0.0%; 60.7%]
  2. Inconsistency
     1. Q statistic to assess consistency under the assumption of a full design-by-treatment interaction random effects model: Q = 7.32, p value = 0.2922
  3. Net heat plot


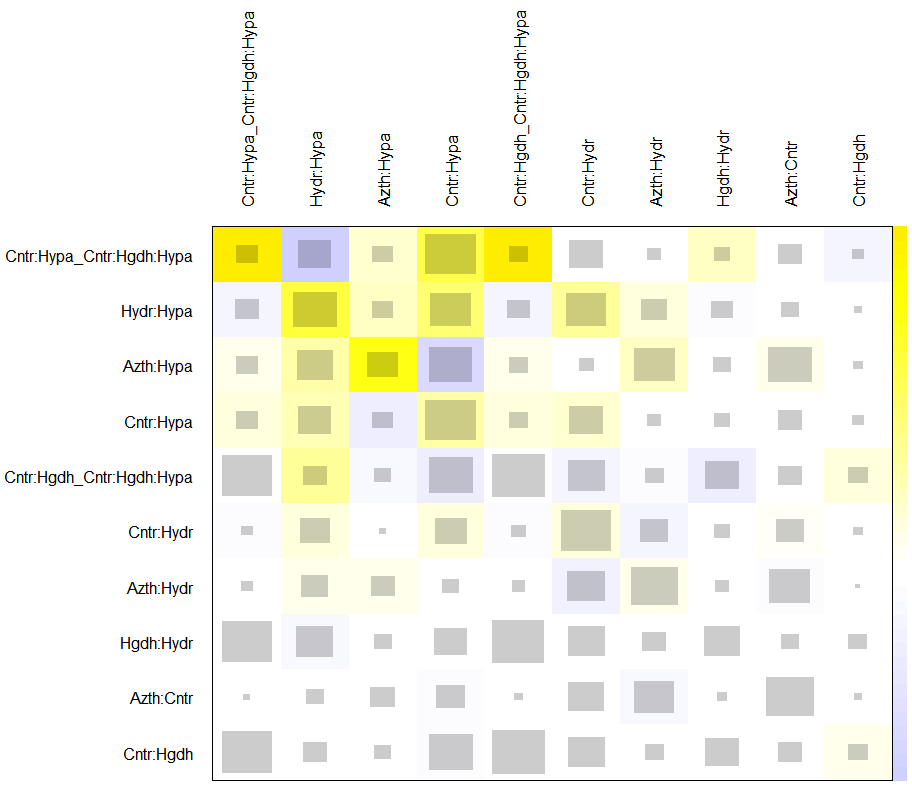


- 1. Comparison-adjusted funnel plot


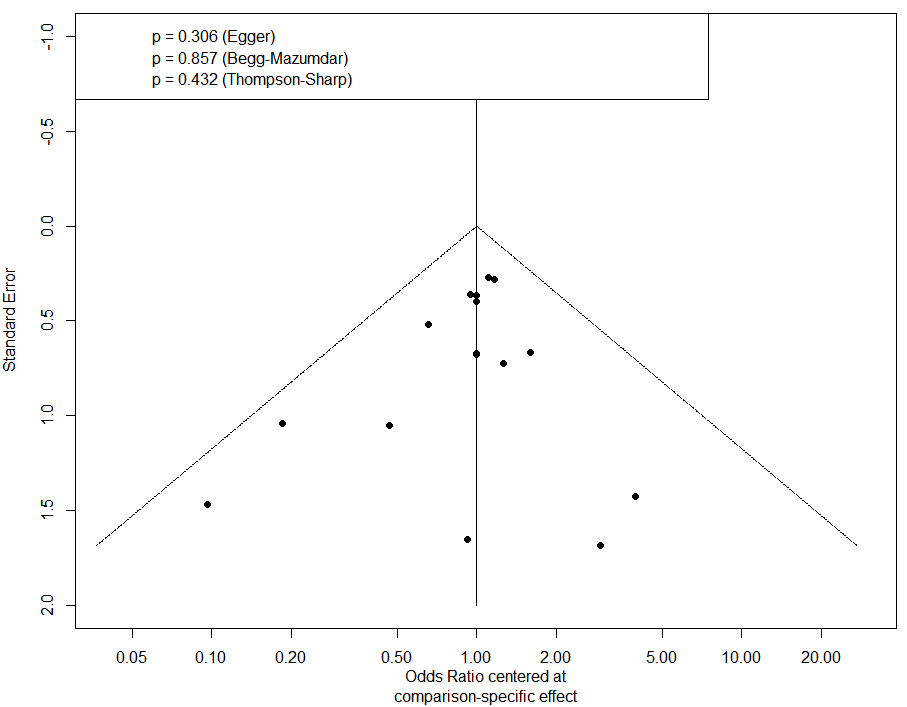


1. **Fatal cardiac complications after HQ (TdP, ventricular tachycardia, and severe ventricular arrhythmia)**
   1. Network map


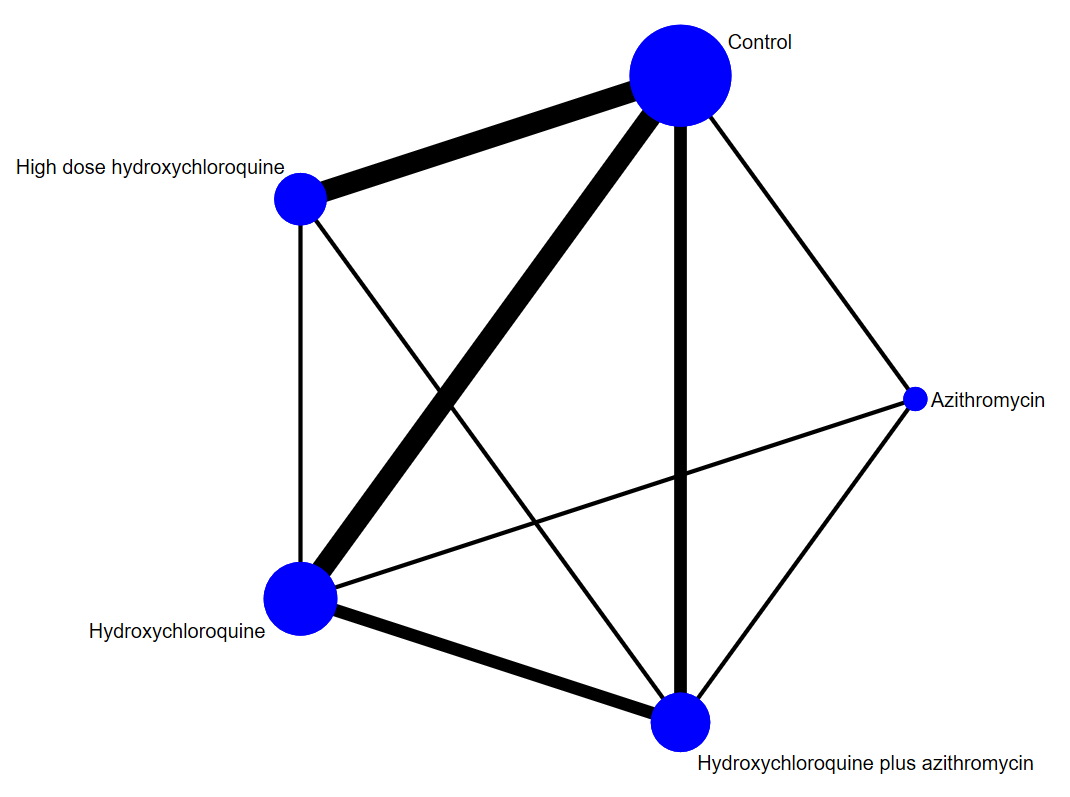


- 1. Forest plot


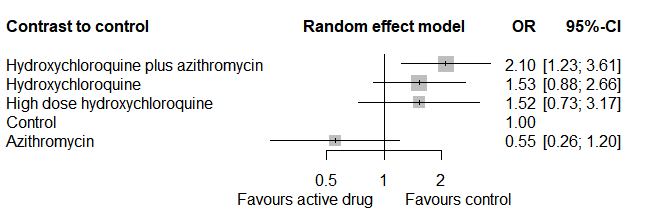


- 1. League table

| Hydroxychloroquine plus azithromycin | 1.90 [0.52 ; 6.93] | 0.83 [0.17 ; 4.16] | 2.17 [1.18 ; 3.98] | 1.36 [0.05 ; 34.55] |
| --- | --- | --- | --- | --- |
| 1.38 [0.69 ;2.75] | Hydroxychloroquine | 0.19 [0.01 ; 4.20] | 1.70 [0.92 ; 3.15] | 2.97 [1.56 ; 5.65] |
| 1.38 [0.59 ;3.23] | 1.00 [0.41 ;2.44] | High dose hydroxychloroquine | 1.26 [0.56 ; 2.81] | . |
| 2.10 [1.23 ;3.61] | 1.53 [0.88 ;2.66] | 1.52 [0.73 ;3.17] | Control | 1.56 [0.66 ; 3.70] |
| 3.81 [1.58 ;9.16] | 2.76 [1.47 ;5.19] | 2.76 [0.97 ;7.81] | 1.81 [0.84 ;3.92] | Azithromycin |

Pairwise (upper right portion) and network (lower left portion) meta-analysis results are presented. Pharmacological agents are reported in order of treatment efficacy (ICU) ranking according to SUCRAs. Comparison should be read from left to right. Effect estimation is presented in odds ratio (OR) with 95% CI and is located in intersection of two agents. OR less than 1 favors the column-defining treatment (lower fatal cardiac complication rate). Since lower fatal cardiac-related adverse rate reflects better outcome, decrement of OR indicates better treatment. To obtain OR (95% CI) for comparison in the opposite direction, reciprocals should be taken

- 1. Direct and indirect evidence proportion for each outcome


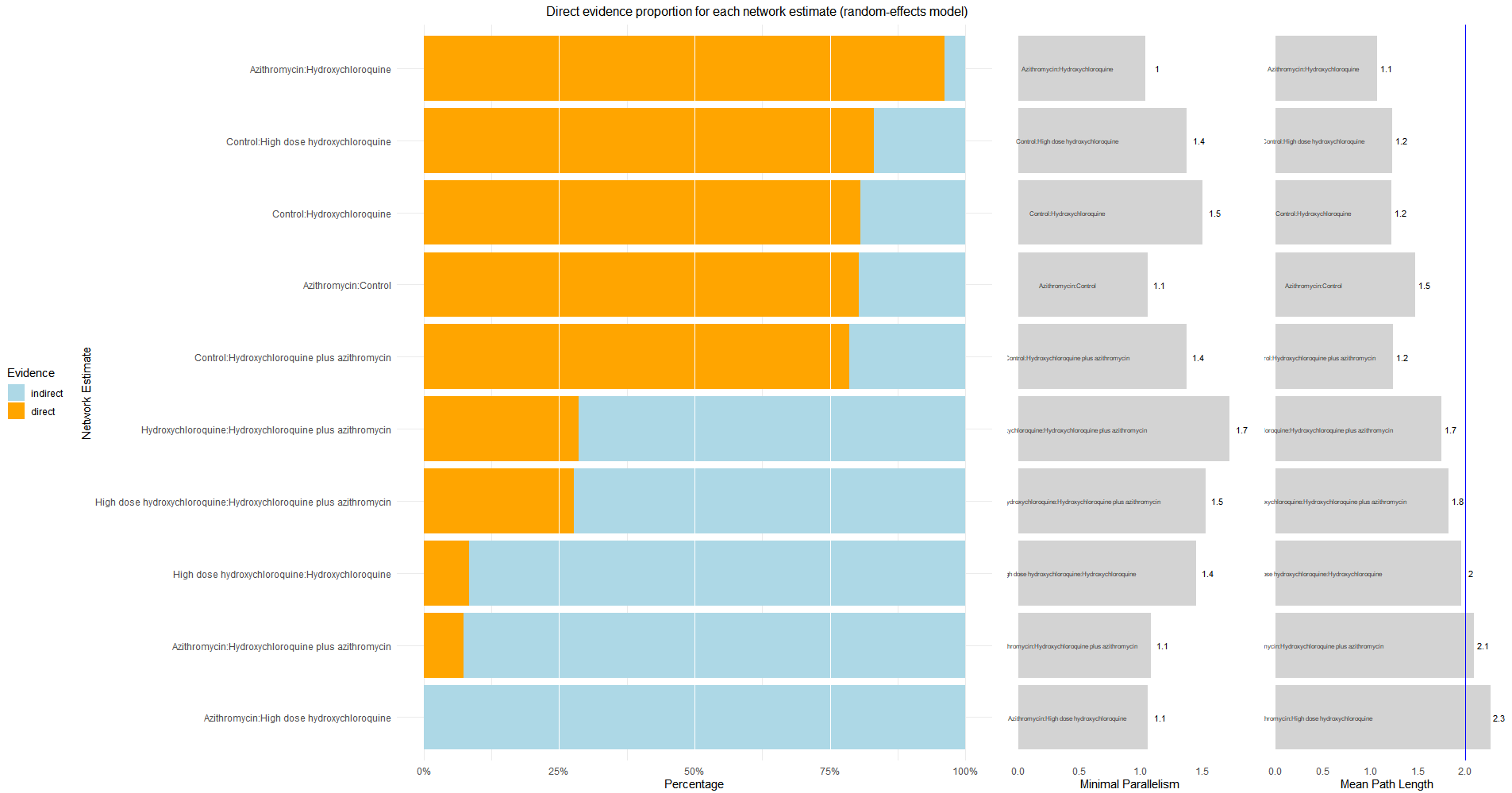


 According to König, Krahn, and Binder ([2013](https://bookdown.org/MathiasHarrer/Doing_Meta_Analysis_in_R/frequentist.html#ref-konig2013visualizing)), lower values of minimal parallelism and Mean Path Length>2 means that results for a specific comparison should be interpreted with caution.

- 1. Heterogeneity
     1. Quantifying heterogeneity: tau^2 = 0; tau = 0; I^2 = 0% [0.0%; 0.0%]
  2. Inconsistency
     1. Q statistic to assess consistency under the assumption of a full design-by-treatment interaction random effects model: Q = 3.33, p value = 0.7658
  3. Net heat plot


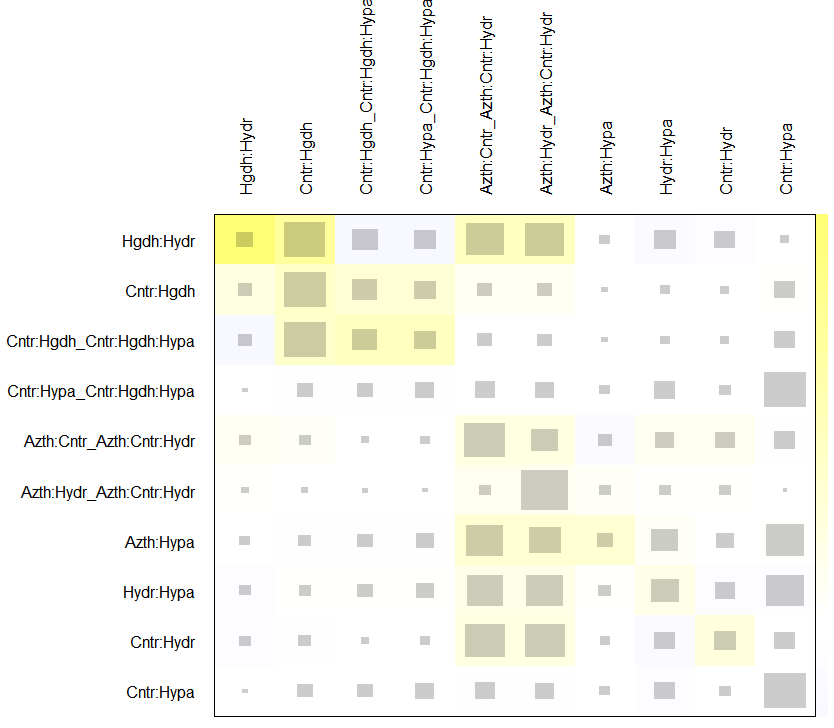


- 1. Comparison-adjusted funnel plot


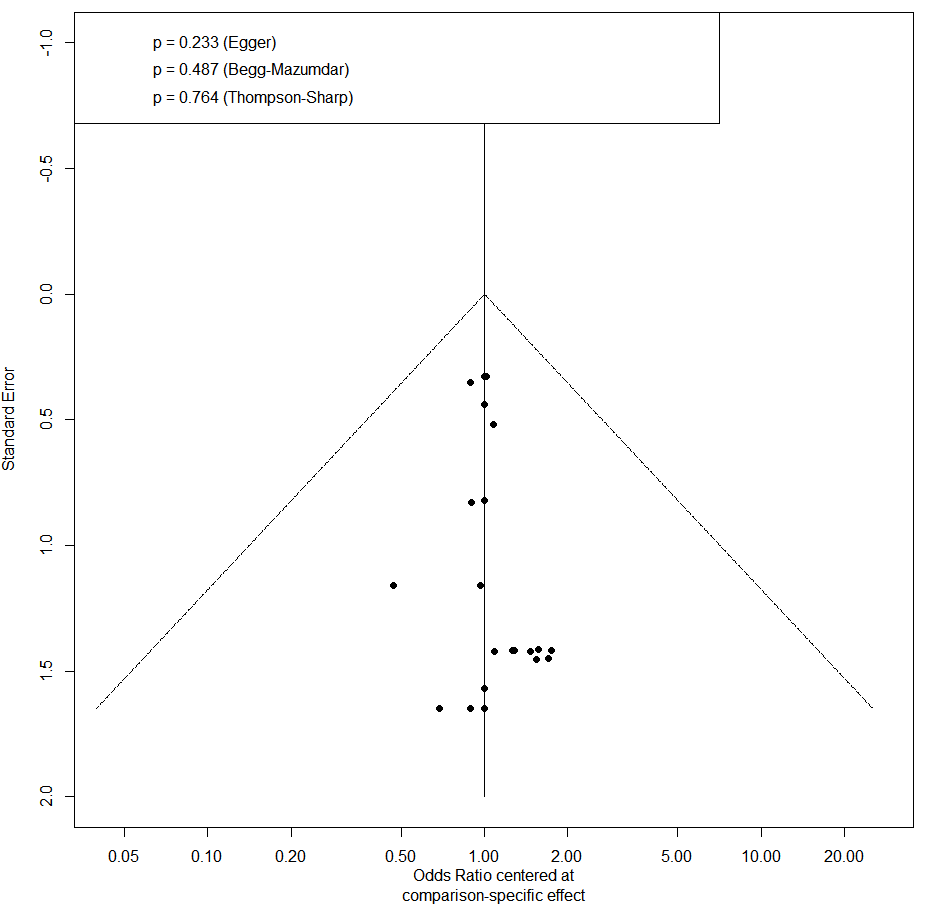


1. **Non-cardiac serious adverse events**
   1. Network map


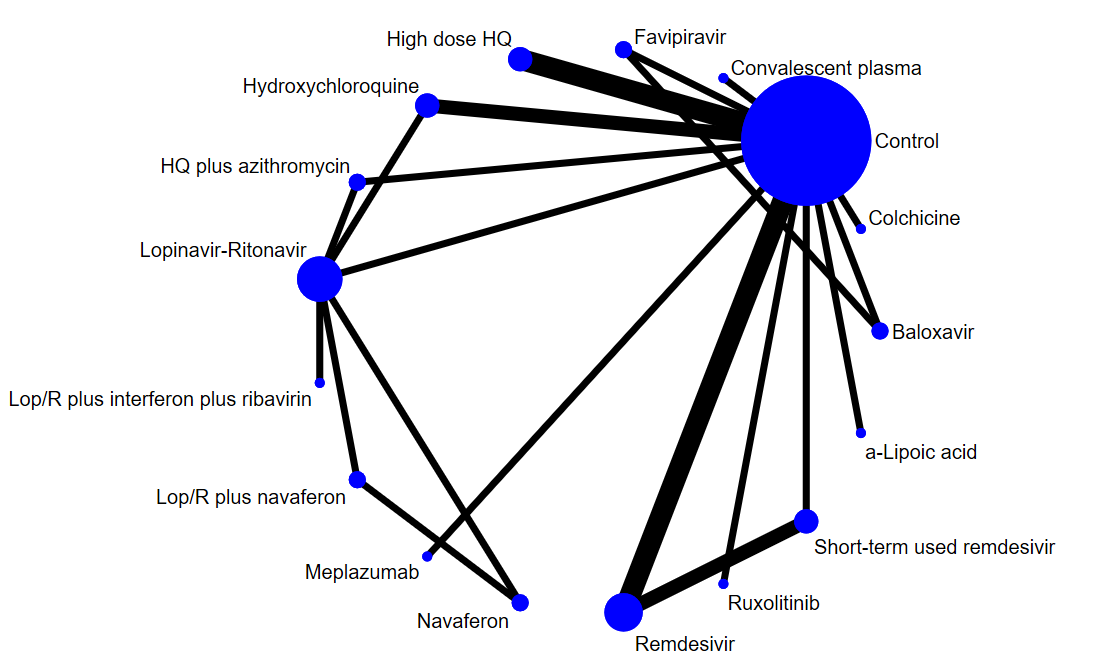


- 1. Forest plot


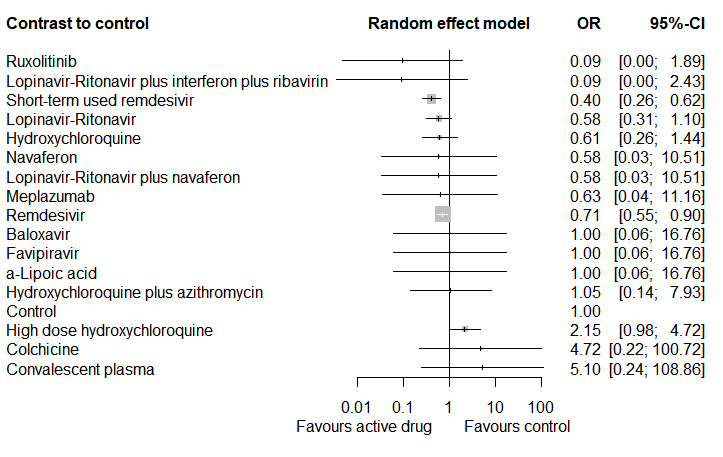


- 1. League table

| Ruxolitinib | . | . | . | . | . | . | . | . | . | . | . | . | 0.09 [0.00 ; 1.89] | . | . | . |
| --- | --- | --- | --- | --- | --- | --- | --- | --- | --- | --- | --- | --- | --- | --- | --- | --- |
| 1.04 [0.01 ; 88.32] | Lopinavir-Ritonavir plus interferon plus ribavirin | . | 0.16 [0.01 ; 3.92] | . | . | . | . | . | . | . | . | . | . | . | . | . |
| 0.24 [0.01 ; 4.91] | 0.23 [0.01 ; 6.31] | Short-term used remdesivir | . | . | . | . | . | 0.56 [0.38 ; 0.84] | . | . | . | . | 0.50 [0.22 ; 1.14] | . | . | . |
| 0.16 [0.01 ; 3.45] | 0.16 [0.01 ; 3.92] | 0.68 [0.31 ; 1.47] | Lopinavir-Ritonavir | 6.42 [0.34 ;119.62] | 1.00 [0.06 ; 16.76] | 1.00 [0.06 ; 16.76] | . | . | . | . | . | 0.73 [0.04 ; 12.22] | 0.52 [0.27 ; 1.01] | . | . | . |
| 0.16 [0.01 ; 3.51] | 0.15 [0.01 ; 4.43] | 0.65 [0.25 ; 1.72] | 0.96 [0.34 ; 2.71] | Hydroxychloroquine | . | . | . | . | . | . | . | . | 0.73 [0.30 ; 1.80] | . | . | . |
| 0.16 [0.00 ; 10.38] | 0.16 [0.00 ; 11.29] | 0.68 [0.04 ; 12.62] | 1.00 [0.06 ; 16.76] | 1.04 [0.05 ; 20.99] | Navaferon | 1.00 [0.06 ; 16.76] | . | . | . | . | . | . | . | . | . | . |
| 0.16 [0.00 ; 10.38] | 0.16 [0.00 ; 11.29] | 0.68 [0.04 ; 12.62] | 1.00 [0.06 ; 16.76] | 1.04 [0.05 ; 20.99] | 1.00 [0.06 ; 16.76] | Lopinavir-Ritonavir plus navaferon | . | . | . | . | . | . | . | . | . | . |
| 0.15 [0.00 ; 9.66] | 0.15 [0.00 ; 11.53] | 0.63 [0.03 ; 11.72] | 0.93 [0.05 ; 17.88] | 0.97 [0.05 ; 19.71] | 0.93 [0.02 ; 55.36] | 0.93 [0.02 ; 55.36] | Meplazumab | . | . | . | . | . | 0.63 [0.04 ; 11.16] | . | . | . |
| 0.13 [0.01 ; 2.70] | 0.13 [0.00 ; 3.48] | 0.56 [0.38 ; 0.83] | 0.83 [0.42 ; 1.63] | 0.86 [0.35 ; 2.11] | 0.83 [0.05 ; 15.04] | 0.83 [0.05 ; 15.04] | 0.89 [0.05 ; 15.97] | Remdesivir | . | . | . | . | 0.69 [0.54 ; 0.88] | . | . | . |
| 0.09 [0.00 ; 5.78] | 0.09 [0.00 ; 6.91] | 0.40 [0.02 ; 6.88] | 0.58 [0.03 ; 10.51] | 0.61 [0.03 ; 11.60] | 0.58 [0.01 ; 33.10] | 0.58 [0.01 ; 33.10] | 0.63 [0.01 ; 35.22] | 0.71 [0.04 ; 11.96] | Favipiravir | 1.00 [0.06 ; 16.76] | . | . | 1.00 [0.06 ; 16.76] | . | . | . |
| 0.09 [0.00 ; 5.78] | 0.09 [0.00 ; 6.91] | 0.40 [0.02 ; 6.88] | 0.58 [0.03 ; 10.51] | 0.61 [0.03 ; 11.60] | 0.58 [0.01 ; 33.10] | 0.58 [0.01 ; 33.10] | 0.63 [0.01 ; 35.22] | 0.71 [0.04 ; 11.96] | 1.00 [0.06 ; 16.76] | Baloxavir | . | . | 1.00 [0.06 ; 16.76] | . | . | . |
| 0.09 [0.00 ; 5.78] | 0.09 [0.00 ; 6.91] | 0.40 [0.02 ; 6.88] | 0.58 [0.03 ; 10.51] | 0.61 [0.03 ; 11.60] | 0.58 [0.01 ; 33.10] | 0.58 [0.01 ; 33.10] | 0.63 [0.01 ; 35.22] | 0.71 [0.04 ; 11.96] | 1.00 [0.02 ; 53.89] | 1.00 [0.02 ; 53.89] | a-Lipoic acid | . | 1.00 [0.06 ; 16.76] | . | . | . |
| 0.09 [0.00 ; 3.33] | 0.09 [0.00 ; 3.89] | 0.38 [0.05 ; 2.98] | 0.56 [0.07 ; 4.19] | 0.58 [0.06 ; 5.16] | 0.56 [0.02 ; 17.83] | 0.56 [0.02 ; 17.83] | 0.59 [0.02 ; 20.07] | 0.67 [0.09 ; 5.14] | 0.95 [0.03 ; 30.51] | 0.95 [0.03 ; 30.51] | 0.95 [0.03 ; 30.51] | Hydroxychloroquine plus azithromycin | 1.38 [0.08 ; 23.13] | . | . | . |
| 0.09 [0.00 ; 1.89] | 0.09 [0.00 ; 2.43] | 0.40 [0.26 ; 0.62] | 0.58 [0.31 ; 1.10] | 0.61 [0.26 ; 1.44] | 0.58 [0.03 ; 10.51] | 0.58 [0.03 ; 10.51] | 0.63 [0.04 ; 11.16] | 0.71 [0.55 ; 0.90] | 1.00 [0.06 ; 16.76] | 1.00 [0.06 ; 16.76] | 1.00 [0.06 ; 16.76] | 1.05 [0.14 ; 7.93] | Control | 0.47 [0.21 ; 1.02] | 0.21 [0.01 ; 4.52] | 0.20 [0.01 ; 4.19] |
| 0.04 [0.00 ; 0.97] | 0.04 [0.00 ; 1.24] | 0.18 [0.08 ; 0.45] | 0.27 [0.10 ; 0.75] | 0.28 [0.09 ; 0.91] | 0.27 [0.01 ; 5.43] | 0.27 [0.01 ; 5.43] | 0.29 [0.01 ; 5.77] | 0.33 [0.14 ; 0.75] | 0.47 [0.02 ; 8.69] | 0.47 [0.02 ; 8.69] | 0.47 [0.02 ; 8.69] | 0.49 [0.06 ; 4.28] | 0.47 [0.21 ; 1.02] | High dose hydroxychloroquine | . | . |
| 0.02 [0.00 ; 1.45] | 0.02 [0.00 ; 1.72] | 0.08 [0.00 ; 1.85] | 0.12 [0.01 ; 2.82] | 0.13 [0.01 ; 3.10] | 0.12 [0.00 ; 8.33] | 0.12 [0.00 ; 8.33] | 0.13 [0.00 ; 8.87] | 0.15 [0.01 ; 3.22] | 0.21 [0.00 ; 13.59] | 0.21 [0.00 ; 13.59] | 0.21 [0.00 ; 13.59] | 0.22 [0.01 ; 8.72] | 0.21 [0.01 ; 4.52] | 0.46 [0.02 ; 10.73] | Colchicine | . |
| 0.02 [0.00 ; 1.34] | 0.02 [0.00 ; 1.59] | 0.08 [0.00 ; 1.71] | 0.11 [0.01 ; 2.61] | 0.12 [0.00 ; 2.87] | 0.11 [0.00 ; 7.71] | 0.11 [0.00 ; 7.71] | 0.12 [0.00 ; 8.21] | 0.14 [0.01 ; 2.98] | 0.20 [0.00 ; 12.58] | 0.20 [0.00 ; 12.58] | 0.20 [0.00 ; 12.58] | 0.21 [0.01 ; 8.08] | 0.20 [0.01 ; 4.19] | 0.42 [0.02 ; 9.94] | 0.93 [0.01 ; 70.21] | Convalescent plasma |

Pairwise (upper right portion) and network (lower left portion) meta-analysis results are presented. Pharmacological agents are reported in order of treatment efficacy (ICU) ranking according to SUCRAs. Comparison should be read from left to right. Effect estimation is presented in odds ratio (OR) with 95% CI and is located in intersection of two agents. OR less than 1 favors the column-defining treatment (lower serious adverse rate). Since lower serious adverse rate reflects better outcome, decrement of OR indicates better treatment. To obtain OR (95% CI) for comparison in the opposite direction, reciprocals should be taken.

- 1. Direct and indirect evidence proportion for each outcome


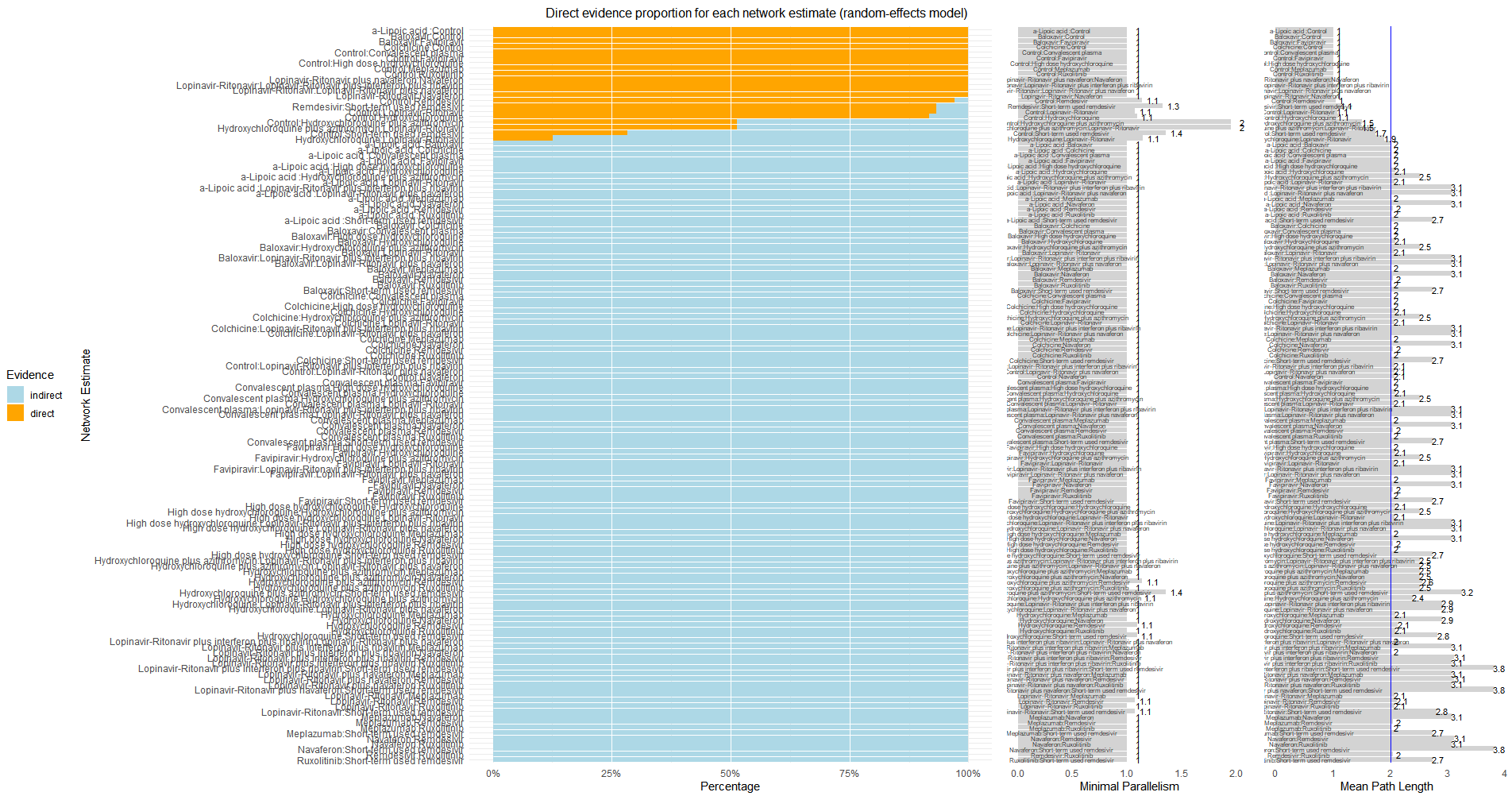


 According to König, Krahn, and Binder ([2013](https://bookdown.org/MathiasHarrer/Doing_Meta_Analysis_in_R/frequentist.html#ref-konig2013visualizing)), lower values of minimal parallelism and Mean Path Length>2 means that results for a specific comparison should be interpreted with caution.

- 1. Heterogeneity
     1. Quantifying heterogeneity: tau^2 = 0; tau = 0; I^2 = 0% [0.0%; 0.0%]
  2. Inconsistency
     1. Q statistic to assess consistency under the assumption of a full design-by-treatment interaction random effects model: Q = 3.20, p value = 0.5247
  3. Net heat plot


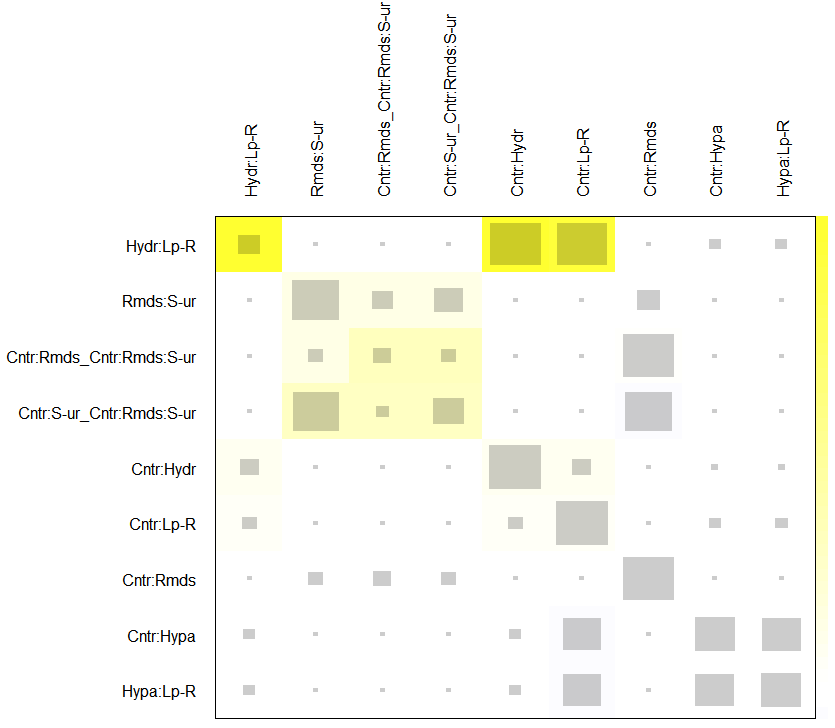


- 1. Comparison-adjusted funnel plot


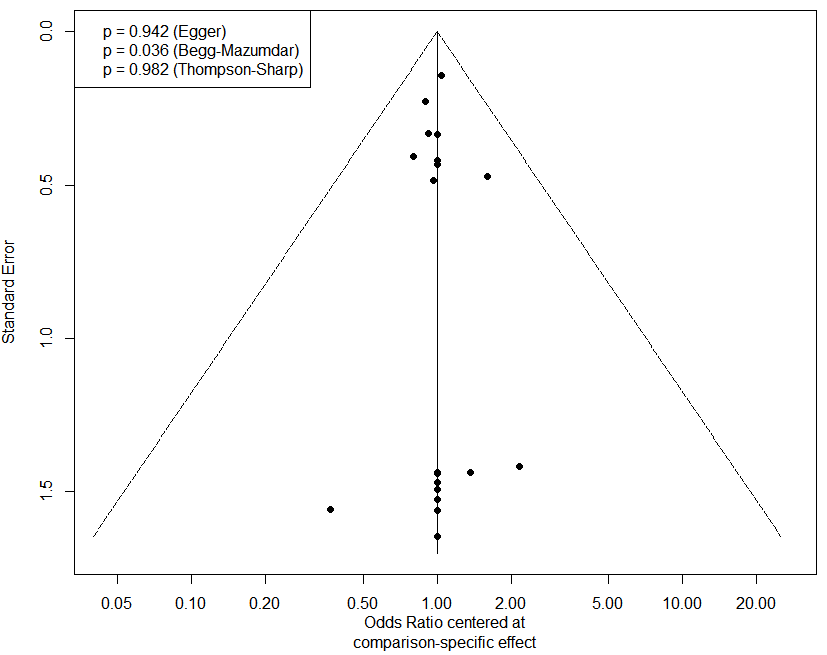

Supplement: S3 Table — (DOCX) [file pmed.1003501.s004.docx]
