# Supplementary material for: Comparative efficacy and safety of pharmacological interventions for the treatment of COVID-19: A systematic review and network meta-analysis
Source: PLoS Med. 2020 Dec 30;17(12):e1003501. doi: 10.1371/journal.pmed.1003501 (PMC7794037; doi:10.1371/journal.pmed.1003501)
Supplement: S4 Table — (DOCX) [file pmed.1003501.s005.docx]

- 1. **Subgroup analysis for moderate-severe (non-ICU) versus critically ill (ICU)**

| **Moderate and severe COVID-19 patients (non-ICU at admission) – average morality rate of 13.1%** | **Critically ill patients (ICU) – average mortality rate of 40.5%** |
| --- | --- |
| Mortality | Mortality |
| 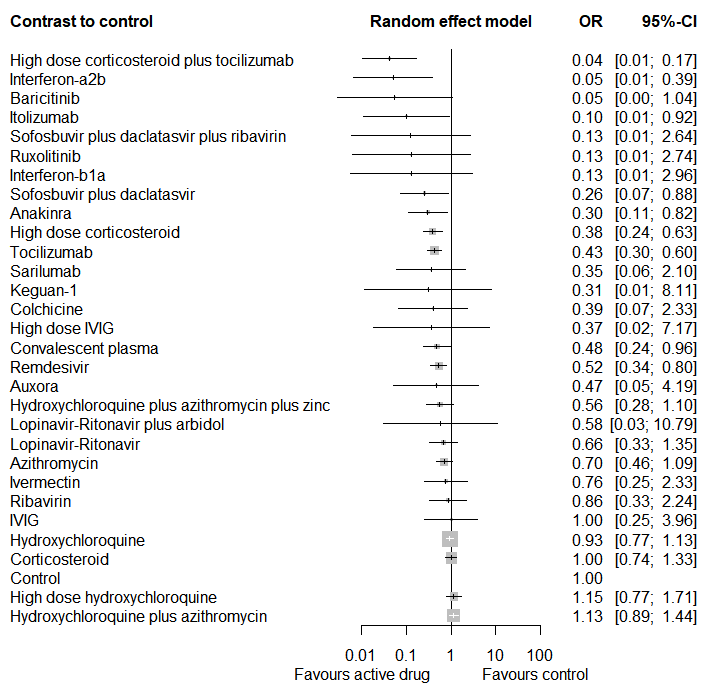 | 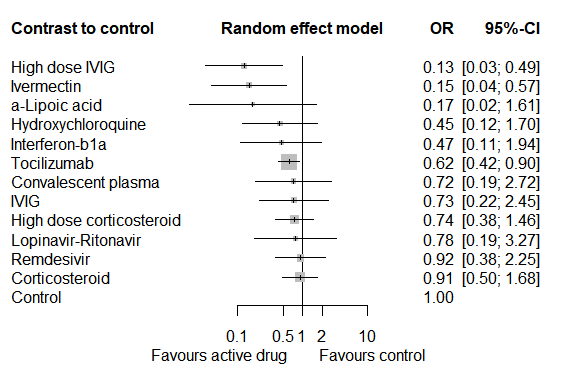 |
| Aggravation to severe course, admission to ICU, and/or mechanical ventilation | Aggravation to severe course, admission to ICU, and/or mechanical ventilation |
| 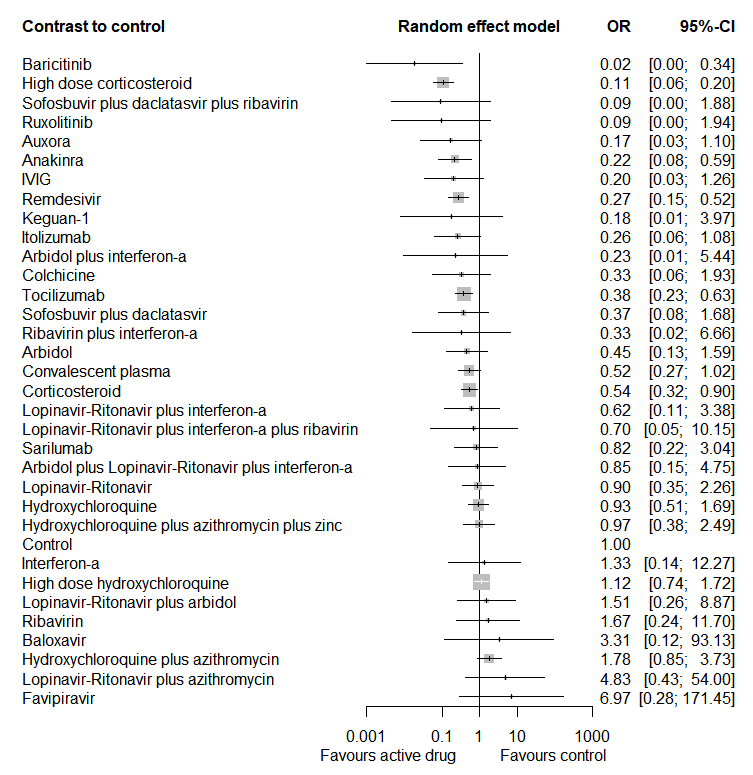 | Not applicable. Only one study. |
| Viral clearance | Viral clearance |
| 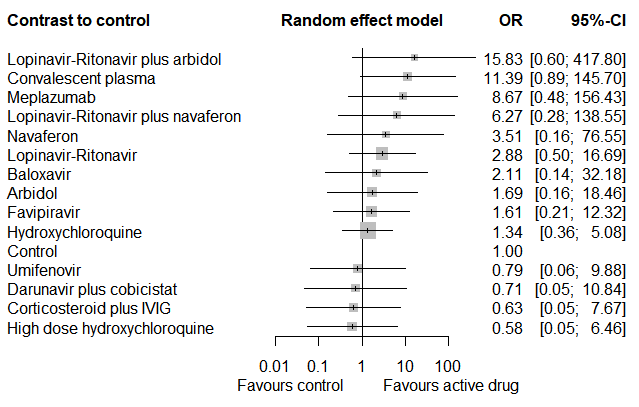 | No studies evaluated viral clearance in critically ill patients |
| Severe cardiac complications | Severe cardiac complications |
| 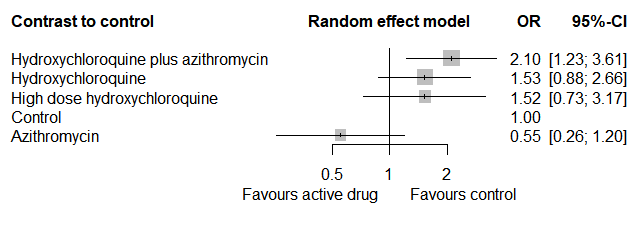 | Unable to connected as a network |
| Non-cardiac serious adverse events | Non-cardiac serious adverse events |
| 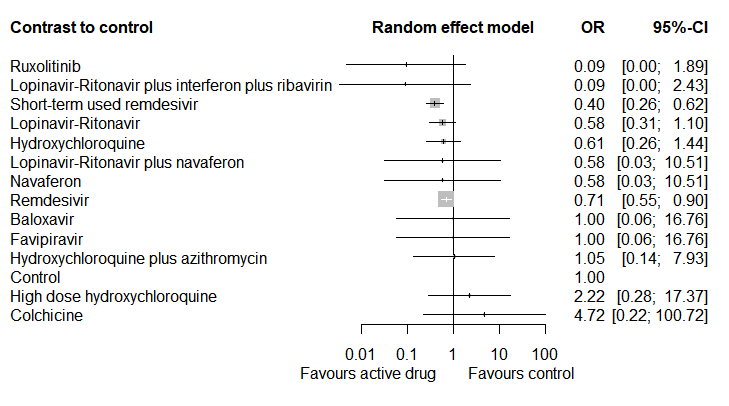 | 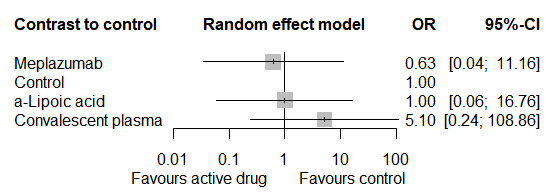 |

- 1. **Specific adverse events**

| **Diarrhea and abdominal discomfort** | **Nausea and vomiting** |
| --- | --- |
| 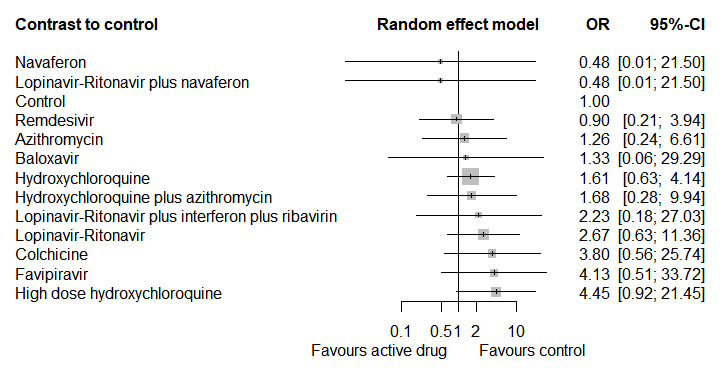 | 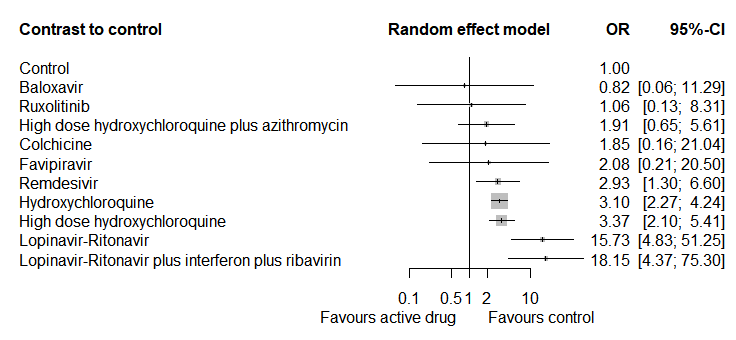 |
| **Hypoalbuminemia** | **Elevated AST/ALT** |
| **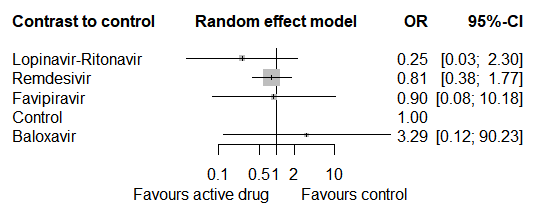** | 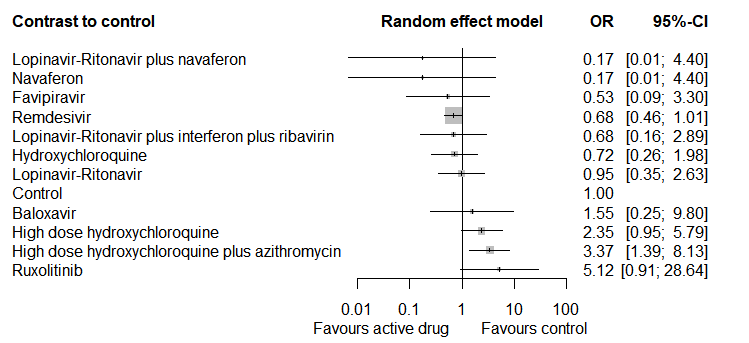 |
| **CK increased** | **Increased total bilirubin** |
| **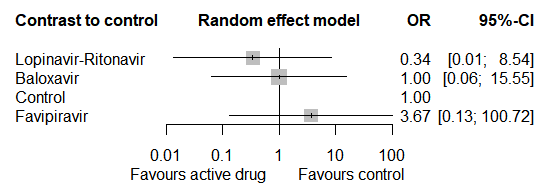** | 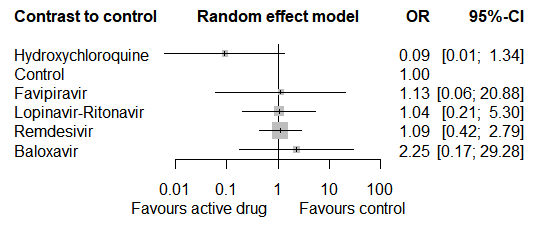 |
| **Anemia** | **Leukopenia** |
| 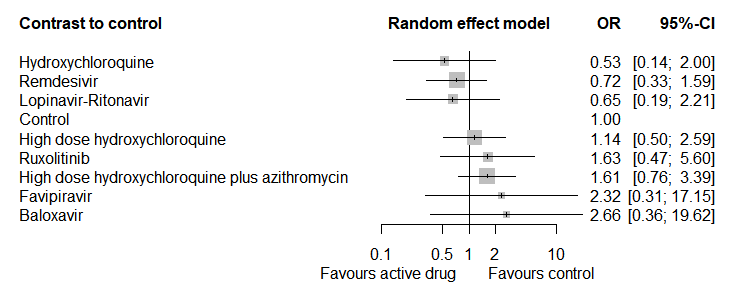 | **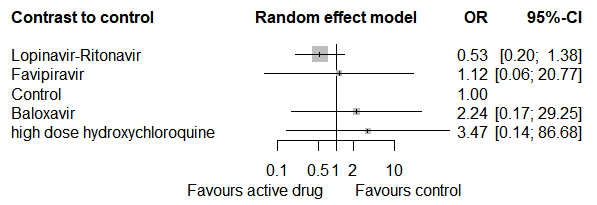** |
| **Lymphopenia** |  |
| 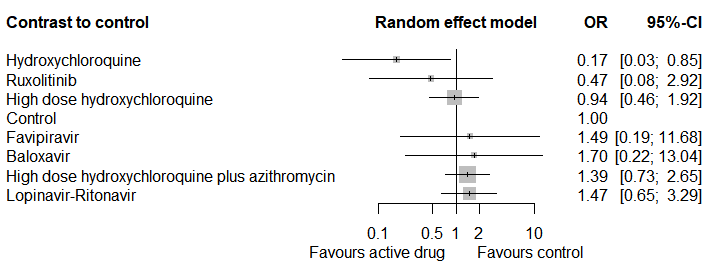 |  |

- 1. **Sensitivity analysis**

| **Sensitivity analysis – RCTs only** | |
| --- | --- |
| Mortality for moderate-severe patients (non-ICU) | Mortality for critically ill patients (ICU) |
| 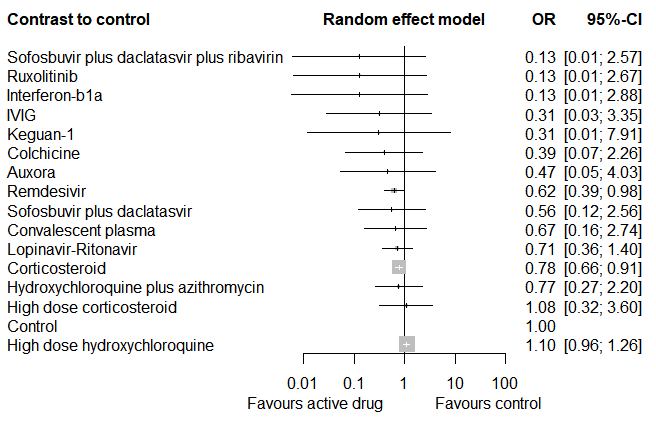 | 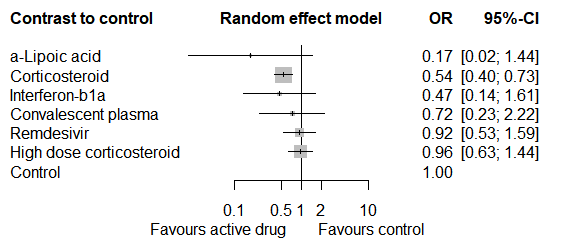 |
| Aggravation to severe course or admission to ICU | Viral clearance rate |
| 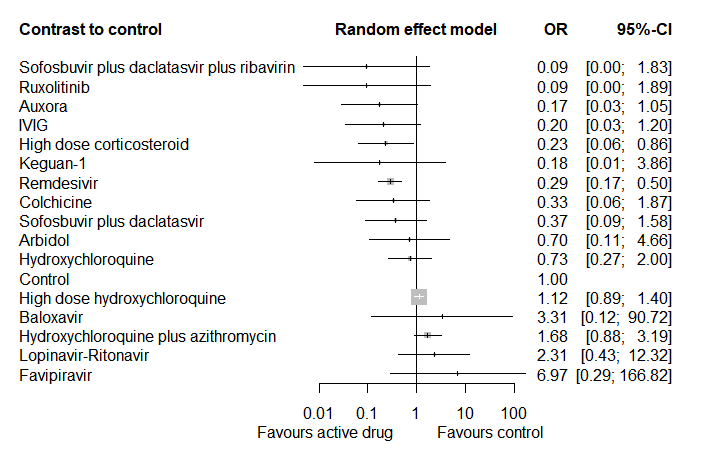 | 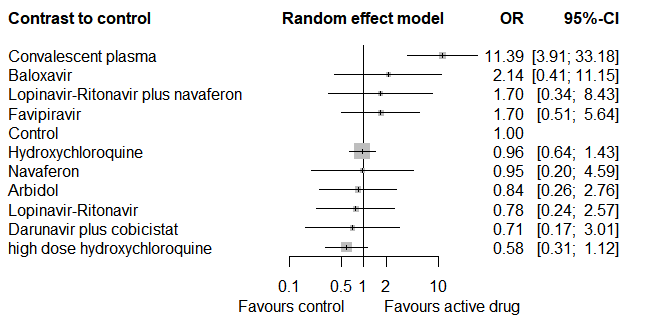 |
| Severe cardiac complications | Non-cardiac serious adverse events |
| RCTs are only relevant for adverse events (AE) with a high incidence. Observational studies are required for AEs that occur with moderate-low incidence and require longer follow-up to determine. Therefore, we found no need to conduct the sensitivity analysis of including only RCTs for the safety evaluation. | RCTs are only relevant for adverse events (AE) with a high incidence. Observational studies are required for AEs that occur with moderate-low incidence and require longer follow-up to determine. Therefore, we found no need to conduct the sensitivity analysis of including only RCTs for the safety evaluation. |

| **Sensitivity analysis – Published studies only** | |
| --- | --- |
| Mortality for moderate-severe patients (non-ICU) | Mortality for critically ill patients (ICU) |
| 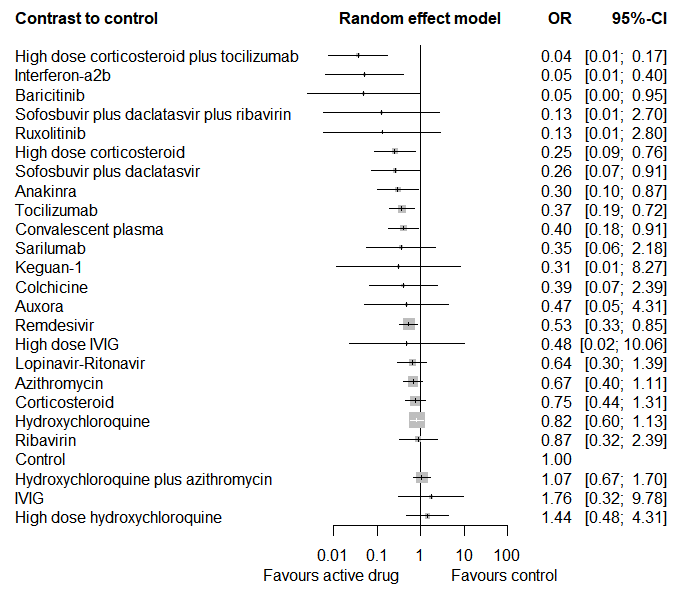 | 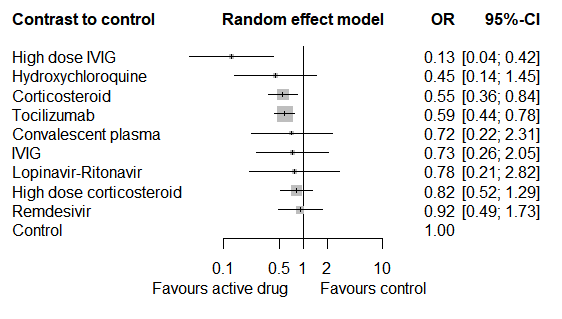 |
| Aggravation to severe course or admission to ICU | Viral clearance rate |
| 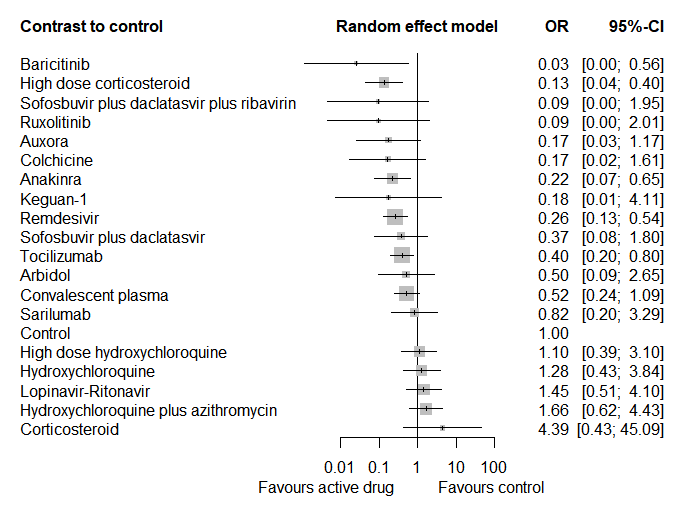 | 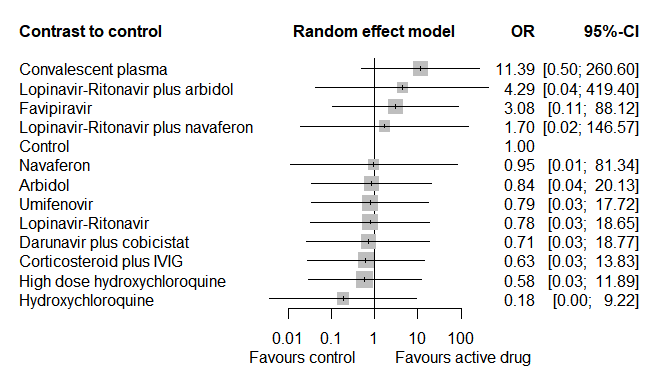 |
| Severe cardiac complications – overall studies | Non-cardiac serious adverse events |
| 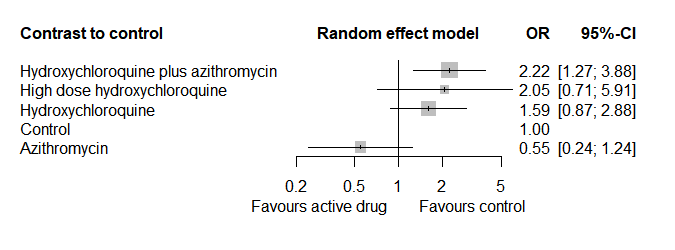 | 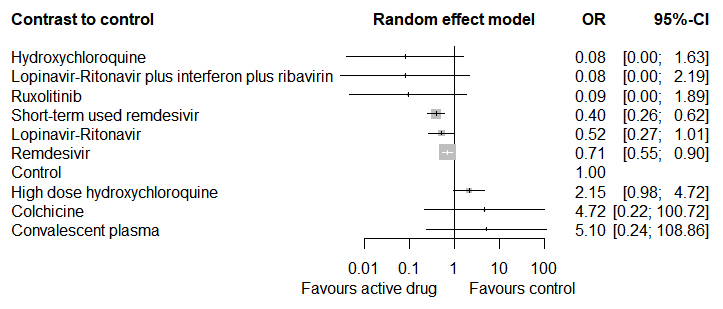 |

| **Sensitivity analysis – Studies with low to moderate risk of bias only (excluding high and serious risk of bias)** | |
| --- | --- |
| Mortality for moderate-severe patients (non-ICU) | Mortality for critically ill patients (ICU) |
| 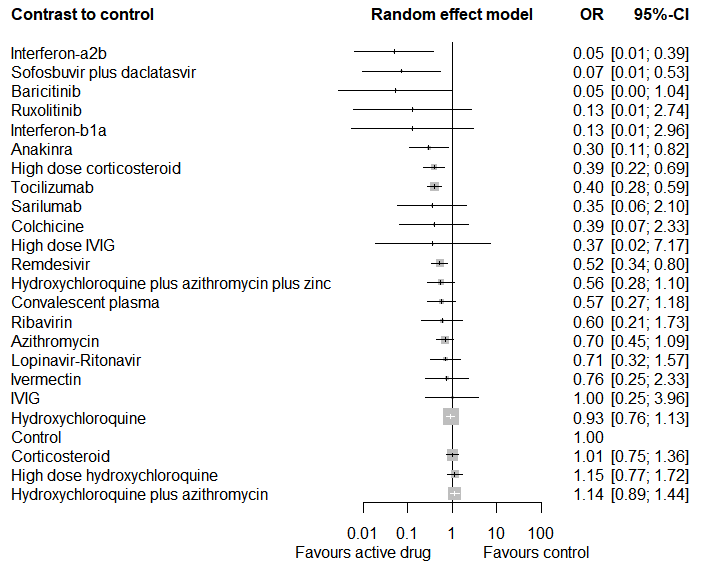 | 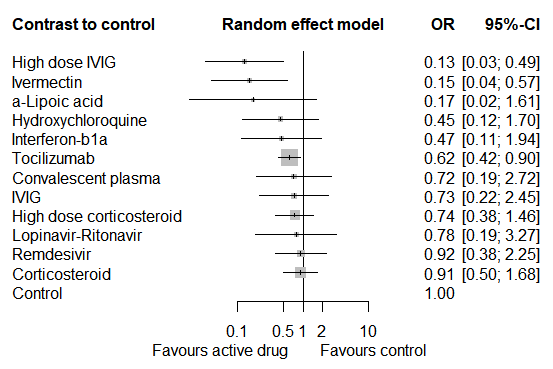 |
| Aggravation to severe course or admission to ICU | Viral clearance rate |
| 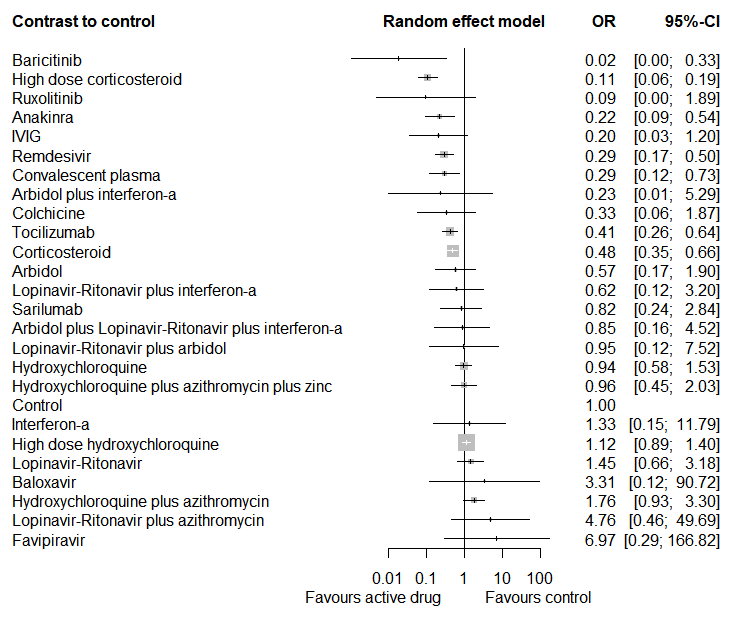 | 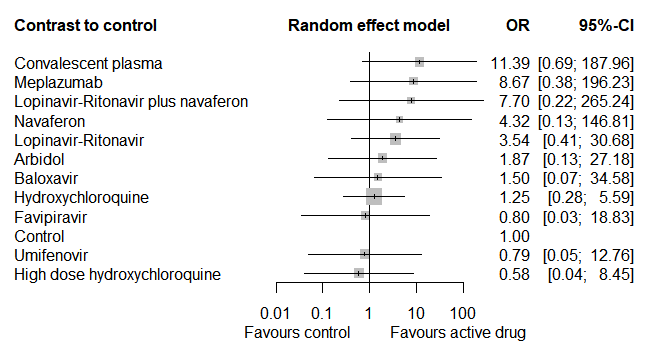 |
| Severe cardiac complications – overall studies | Non-cardiac serious adverse events |
| 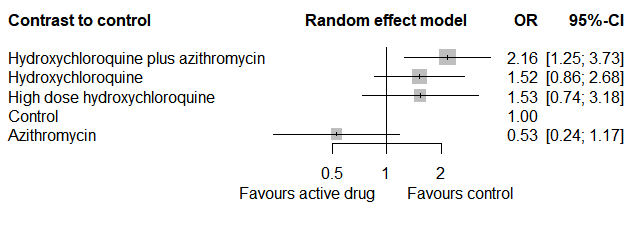 | 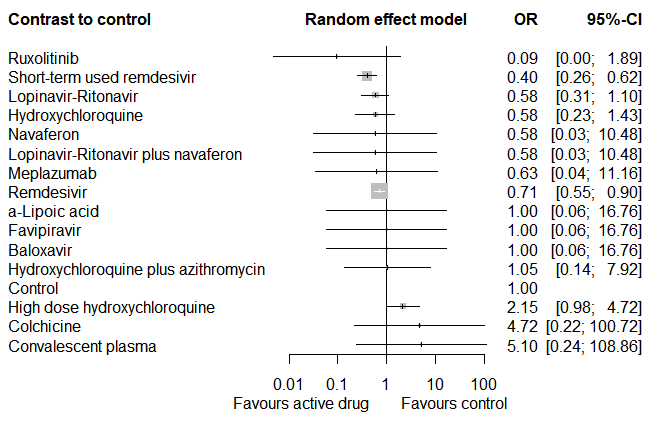 |
