# Supplementary material for: Comparative efficacy and safety of pharmacological interventions for the treatment of COVID-19: A systematic review and network meta-analysis
Source: PLoS Med. 2020 Dec 30;17(12):e1003501. doi: 10.1371/journal.pmed.1003501 (PMC7794037; doi:10.1371/journal.pmed.1003501)
Supplement: S5 Table — (DOCX) [file pmed.1003501.s006.docx]

- 1. **Excluded studies**
     1. Due to its volume, we could not describe all excluded papers, but we present representative excluded papers herein

| First author | Title | Rationale |
| --- | --- | --- |
| Yuan et al. | The correlation between viral clearance and biochemical outcomes of 94 COVID‑19 infected discharged patients | No sufficient information on control and adjustment. |
| Li et al. | (pre-print) Clinical efficacy of Arbidol in patients with 2019 novel coronavirus infected pneumonia: a retrospective cohort study | Empirical treatment group composes of mixture of numerous kinds of medications. |
| Duan et al. | Effectiveness of convalescent plasma therapy in severe COVID-19 patients | Before-after treatment study. No independent control group. |
| Yu et al. | Low dose of hydroxychloroquine reduces fatality of critically ill patients with COVID-19 | Did not provide sufficient statistical information for the outcomes of interest. |
| Deng et al. | Arbidol combined with LPV/r versus LPV/r alone against Corona Virus Disease 2019: A retrospective cohort study | Significant high proportion of corticosteroid use in monotherapy group. No adjustment conducted. |
| Wu et al. | Risk Factors Associated With Acute Respiratory Distress Syndrome and Death in Patients With Coronavirus Disease 2019 Pneumonia in Wuhan, China | Significantly different severity indices between no prednisolone group vs prednisolone group with no further adjustment |
| Cavalli et al. | Interleukin-1 blockade with high-dose anakinra in patients with COVID-19, acute respiratory distress syndrome, and hyperinflammation: a retrospective cohort study | Baseline characteristics (demography, severity indices, laboratory findings, etc) was significantly different between treatment groups and was not adjusted. |
| Quartuccio et al. | Profiling COVID-19 pneumonia progressing into the cytokine storm syndrome: results from a single Italian Centre study on tocilizumab versus standard of care | Baseline characteristics (demography, severity indices, laboratory findings, etc) was significantly different between treatment groups and was not adjusted. |
| Gautret et al. | Hydroxychloroquine and azithromycin as a treatment of COVID-19: results of an open-label non-randomized clinical trial | Study design is not sophisticated and method part does not describe sufficiently for its study. Difficult to judge whether treatment groups are well controlled or not. |
| Reynolds et al. | Renin–Angiotensin–Aldosterone System Inhibitors and Risk of Covid-19 | Initiation of medication prior to diagnosis of COVID-19 (met one of our predefined exclusion criteria) |
| Mehta et al. | Association of Use of Angiotensin-Converting Enzyme Inhibitors and Angiotensin II Receptor Blockers With Testing Positive for Coronavirus Disease 2019 (COVID-19) | Initiation of medication prior to diagnosis of COVID-19 (met one of our predefined exclusion criteria) |
| Bajo et al. | Use of renin–angiotensin–aldosterone system inhibitors and risk of COVID-19 requiring admission to hospital: a case-population study | Initiation of medication prior to diagnosis of COVID-19 (met one of our predefined exclusion criteria) |
| Zhou et al. | Interferon-a2b Treatment for COVID-19 | Significantly different initiation of treatment after symptom onset between treatment groups (not adjusted) |
| Mehra et al. | Hydroxychloroquine or chloroquine with or without a macrolide for treatment of COVID-19: a multinational registry analysis | Retracted |
| Membrillo et al. | (pre-print) Early hydroxychloroquine is associated with an increase of survival in COVID-19 patients: an observational study. | Baseline characteristics (demography, severity indices, laboratory findings, etc) was significantly different between treatment groups and was not adjusted. |
| Fadel et al. | Early Short Course Corticosteroids in Hospitalized Patients with COVID-19 | Both treatment group received corticosteroid |
| Sanchez-Montalva et al. | (pre-print) Early outcomes of tocilizumab in adults hospitalized 1 with severe COVID-19: An initial report from the Vall d Hebron COVID-19 prospective cohort study. | No independent control group |
| Michael et al. | (pre-print) Early Safety Indicators of COVID-19 Convalescent Plasma in 5,000 Patients | No independent control group |
| Chen at al. | (pre-print) Favipiravir versus Arbidol for COVID-19: A Randomized Clinical Trial | Inclusion criteria for COVID-19 was loose. They can be diagnosed without PCR. |
| Shabrawishi et al. | (pre-print) Negative nasopharyngeal SARS-CoV-2 PCR conversion in Response different therapeutic interventions. | Treatment drugs are not controlled. Mixed up. |
| Hong et al. | (pre-print) Celebrex adjuvant therapy on COVID-19: An experimental study | Lack of outcomes of interest. |
| Mallat et al. | (pre-print) Hydroxychloroquine is associated with slower viral clearance in clinical COVID-19 patients with mild to moderate disease: A retrospective study | More pneumonia in one group. Baseline characteristics (demography, severity indices, laboratory findings, etc) was significantly different between treatment groups and was not adjusted. |
| Xu et al. | (pre-print) Effective Treatment of Severe COVID-19 Patients with Tocilizumab | No independent control group |
| Salazar et al. | (pre-print) Treatment of COVID-19 Patients with Convalescent Plasma in  Houston, Texas | No independent control group |
| Patterson et al. | (pre-print) Disruption of the CCL5/RANTES-CCR5 Pathway Restores Immune Homeostasis and Reduces Plasma Viral Load in Critical COVID-19 | Not a clinical pharmacological trial/study. |
| Watson et al. | (pre-print) Concentration-dependent mortality of chloroquine in overdose | No sufficient statistical information for outcome of interest. |
| Yan et al. | (pre-print) Factors associated with prolonged viral shedding and impact of Lopinavir/Ritonavir treatment in patients with SARS-CoV-2 infection | No sufficient statistical information for outcome of interest. |
| Lazzeri et al. | (pre-print) Impact of anti-androgenic therapies on COVID-19: an observational study in male population from a COVID-19 regional centre of Lombardy (Italy) | Only male. No sufficient statistical information for outcome of interest. |
| Izoulet et al. | (pre-print) National Consumption of Antimalarial Drugs and COVID-19 Deaths Dynamics: An Ecological Study | Ecological study. No sufficient statistical information for outcome of interest. |
| Chroboczek et al. | (pre-print) Beneficial effect of corticosteroids in severe COVID-19 pneumonia: a propensity score matching analysis. | No sufficient information on baseline |
| Wang et al. | (pre-print) No Clear Benefit to the Use of Corticosteroid as Treatment in Adult Patients with Coronavirus Disease 2019 : A Retrospective Cohort Study | Not adjusted for primary outcomes |
| Ashraf et al. | (pre-print) COVID-19 in Iran, a comprehensive investigation from exposure to treatment outcomes | Baseline characteristics (demography, severity indices, laboratory findings, etc) was significantly different between treatment groups and was not adjusted. |
| Castro et al. | (pre-print) Brief Report: Identifying common pharmacotherapies associated with reduced COVID-19 morbidity using electronic health records | Baseline characteristics (demography, severity indices, laboratory findings, etc) was significantly different between treatment groups and was not adjusted. |
| Dauchet et al. | (pre-print) ACE inhibitors, AT1 receptor blockers and COVID-19 : clinical epidemiology evidences for a continuation of treatments. The ACER-COVID study | Initiation of medication prior to diagnosis of COVID-19 (met one of our predefined exclusion criteria) |
| Wang et al. | (pre-print) Efficacy and Safety of Leflunomide for Refractory COVID-19: An Open-label Controlled Study | Non-generalizable study population (refractory COVID-19 patients) |
| Lother et al. | (pre-print) Post-exposure Prophylaxis or Preemptive Therapy for SARS-Coronavirus-2: Study Protocol for a Pragmatic Randomized Controlled Trial | Study protocol. |
| Davies et al. | (pre-print) Remdesivir in treatment of COVID-19: A systematic benefit-risk assessment | Systematic review |
| Davido et al. | (pre-print) Hydroxychloroquine plus azithromycin: a potential interest in reducing in hospital morbidity due to COVID-19 pneumonia (HI-ZY-COVID) | Uncontrolled baseline and incomplete death record. |
| Cawling et al. | Impact assessment of non-pharmaceutical interventions against coronavirus disease 2019 and influenza in Hong Kong: an observational study | Non-pharmacological intervention |
| Meng et al. | (pre-print) An experimental trial of recombinant human interferon alpha nasal drops to prevent COVID-19 in medical staff in an epidemic area | Preventive intervention which is not within the scope of our interest. |
| Liu et al. | (pre-print) Anti-hypertensive Angiotensin II receptor blockers associated to mitigation of disease severity in elderly COVID-19 patients | Initiation of medication prior to diagnosis of COVID-19 (met one of our predefined exclusion criteria) |
| Rimland et al. | (pre-print) Clinical characteristics and early outcomes in patients with COVID-19 treated with tocilizumab at a United States academic center | No independent control group |
| Liu et al. | (pre-print) Clinical features and progression of acute respiratory distress syndrome in coronavirus disease 2019 | Not a pharmacological study. |
| Jurado et al. | (pre-print) COVID-19 in Spain: age, Interleukin-6, C Reactive Protein and lymphocytes as key clues from a multicenter retrospective study. | Laboratory-based study. |
| Yan et al. | (pre-print) Factors associated with prolonged viral shedding and impact of Lopinavir/Ritonavir treatment in patients with SARS-CoV-2 infection | Severity indices are significantly different between groups.Not adjusted. |
| Chen et al. | (pre-print) First Clinical Study Using HCV Protease Inhibitor Danoprevir to Treat Naïve and Experienced COVID-19 Patients | Small sample size. |
| Negril et al. | (pre-print) Heparin therapy improving hypoxia in COVID-19 patients - a case series | No sufficient information on baseline. |
| Fan et al. | (pre-print) treatment of 55 patients with COVID-19 from seven cities in northeast China who fully recovered: a single-center, retrospective, observational study | No sufficient information on baseline of each medication group. |
| Amherst et al. | (pre-print) Quantifying Treatment Effects of hydroxychloroquine and azithromycin for COVID-19: a secondary analysis of an open label non-randomized clinical trial | No sufficient information on baseline. |
| Lane et al. | (pre-print) Safety of hydroxychloroquine, alone and in combination with azithromycin, in light of rapid wide-spread use for COVID-19: a multinational, network cohort and self-controlled case series study | Not sure about credibility of data sources |
| Gremese et al. | (pre-print) Sarilumab use in severe SARS-CoV-2 pneumonia | No sufficient information on baseline of each medication group. |
| Liu et al. | (pre-print) The effect of Arbidol Hydrochloride on reducing mortality of Covid-19 patients: a retrospective study of real-world data from three hospitals in Wuhan | Medication mixed up. Cannot rule out the effect of various medication to one another. No sufficient information on baseline (only age, comorbidieis, lesion size, and SpO2 are present). |
| Duan et al | (pre-print)The feasibility of convalescent plasma therapy in severe COVID-19 patients: a pilot study | Pilot study with small sample size. |
| Liu et al. | (pre-print) Therapeutic effects of dipyridamole on COVID-19 patients with coagulation dysfunction | Non-generalizable study population (patients with coagulation dysfuntion) |
| Gritti et al. | (pre-print) Use of siltuximab in patients with COVID-19 pneumonia requiring ventilatory support | No independent control group |
| Rodriguez-Nava et al. | Atorvastatin associated with decreased hazard for death in COVID-19 patients  admitted to an ICU: a retrospective cohort study | Medication initiated prior to COVID-19 diagnosis. Editorial. |
| Yamamura et al. | Effect of favipiravir and an anti-inflammatory strategy for COVID-19 | Single arm study. Not a comparative study. |
| Gucyetmez et al. | Therapeutic plasma exchange in patients with COVID-19 pneumonia in intensive care unit: a retrospective study | Not a pharmacological study. |
| Nichols et al. | The Role of Remdesivir in South Africa: Preventing COVID-19 Deaths Through Increasing Intensive Care Unit Capacity | Modelling study |
| Keske et al. | Appropriate use of Tocilizumab in COVID-19 Infection | Not a comparative study |
| Lopez et al. | Effects of Hydroxychloroquine on Covid-19 in Intensive Care Unit Patients: Preliminary Results | Patients in both arm took HQ and differentiate groups by the concentration of serum HQ level. |
| Davido et al. | Impact of medical care including anti-infective agents use on the prognosis of COVID-19 hospitalized patients over time | Does not present the outcomes of interest (e.g., morality, aggravation, etc) |
| Shi et al. | Successful treatment with plasma exchange followed by intravenous immunoglobulin in a critically ill patient with COVID-19 | Case report |
| Veerdonk et al. | Outcomes Associated With Use of a Kinin B2 Receptor Antagonist Among Patients With COVID-19 | Does not present the outcomes of interest (e.g., morality, aggravation, etc) |
| Mustafa et al. | Extracorporeal Membrane Oxygenation for Patients With COVID-19 in Severe Respiratory Failure | Not a pharmacological study. |
| Schmidt et al. | Extracorporeal membrane oxygenation for severe acute respiratory distress syndrome associated with COVID-19: a retrospective cohort study | Not a pharmacological study. |
| Khin et al. | A potential therapeutic combination for treatment of COVID-19: synergistic  effect of DPP4 and RAAS suppression | Does not present the outcomes of interest (e.g., morality, aggravation, etc) |
| Satlin et al. | Safety, tolerability, and clinical outcomes of hydroxychloroquine for hospitalized patients with coronavirus 2019 disease | A single arm study |
| Strohbehn et al. | COVIDOSE: Low-dose tocilizumab in the treatment of Covid-19 | Does not present the outcomes of interest (e.g., morality, aggravation, etc) |
| Giacomelli et al. | EARLY ADMINISTRATION OF LOPINAVIR/RITONAVIR PLUS HYDROXYCHLOROQUINE DOES NOT ALTER THE CLINICAL COURSE OF SARSCOV-2 INFECTION: A RETROSPECTIVE COHORT STUDY. | Early versus delay treatment. |
| Tan et al. | A cohort study to evaluate the effect of combination Vitamin D, Magnesium and Vitamin B12 (DMB) on progression to severe outcome in older COVID-19 patients. | Investigated mineral supplements rather than pharmacological drug. |
| Pang et al. | Efficacy and tolerability of bevacizumab in patients with severe Covid -19 | Lack of outcomes of interest |
| Mfeukeu-Kuate et al. | Electrocardiographic safety of daily Hydroxychloroquine 400mg plus Azithromycin 250mg as an ambulatory treatment for COVID-19 patients in Cameroon | A single arm study. Not comparative study. |
| Temesgen et al. | First Clinical Use of Lenzilumab to Neutralize GM-CSF in Patients with Severe and Critical COVID-19 Pneumonia | Not a comparative study |
| Hartman et al. | Hospitalized COVID-19 Patients treated with Convalescent Plasma in a Mid-size City in the Midwest | Not a comparative study |
| Hashimoto et al. | A retrospective study evaluating efficacy and safety of compassionate  use of tocilizumab in 13 patients with severe-to-critically ill COVID-19:  analysis of well-responding cases and rapidly-worsening cases after  tocilizumab administration | Compassionate use of tocilizumab (single arm study) |
| Oteo et al. | A short therapeutic regimen based on hydroxychloroquine plus  azithromycin for the treatment of COVID-19 in patients with moderate disease. A strategy associated with a reduction in hospital admissions and complications | Single arm study. No sufficient information on baseline |
| Hoertel et al. | Association between SSRI Antidepressant Use and Reduced Risk of Intubation or Death in Hospitalized Patients with Coronavirus Disease 2019: a Multicenter Retrospective Observational Study | Uncertain whether antidepressants were administrated prior to or after diagnosis of COVID-19 |
| Isaksen et al. | Chloroquine, but not hydroxychlorquine, prolongs the QT interval in a primary care population | Absent of dosage information which is crucial for proper analysis |
| Donato et al. | Clinical and laboratory evaluation of patients with SARS-CoV-2 pneumonia treated with high-titer convalescent plasma: a prospective study | Compassionate use |
| Rahman et al. | Corticosteroid Use in Severely Hypoxemic COVID-19 Patients: An Observational Cohort Analysis of Dosing Patterns and Outcomes in the Early Phase of the Pandemic | Not adjusted for severity (SOFA scores were significantly different between groups) |
| Hu et al. | A Small-Scale Medication of Leflunomide as a Treatment of COVID-19 in an Open-Label Blank-Controlled Clinical Trial | Small scale pilot study |
| Lee et al. | An observational cohort study of hydroxychloroquine and azithromycin for COVID-19: (Can't Get No) Satisfaction | Editorial that discusses another study |
| Berenguer et al. | Characteristics and predictors of death among 4,035 consecutively hospitalized patients with COVID-19 in Spain | No comparisons made between pharmacologic agents |
| Lee et al. | Clinical Experience with Use of Remdesivir in the Treatment of Severe Acute Respiratory Syndrome Coronavirus 2: a Case Series | Case series |
| Grein et al. | Compassionate Use of Remdesivir for Patients with Severe Covid-19 | No comparisons made between pharmacologic agents |
| Jordan et al. | Compassionate Use of Tocilizumab for Treatment of SARS-CoV-2 Pneumonia | No comparisons made between pharmacologic agents |
| Gendelman et al. | Continuous hydroxychloroquine or colchicine therapy does not prevent infection with SARS-CoV-2: Insights from a large healthcare database analysis | Focus on prophylaxis |
| Khan et al. | COVID-19: Clinical aspects and therapeutics responses | No comparisons made between pharmacologic agents |
| Li et al. | Duration of SARS-CoV-2 RNA shedding and factors associated with prolonged viral shedding in patients with COVID-19 | No comparisons made between pharmacologic agents |
| Hong et al. | Early Hydroxychloroquine Administration for Rapid Severe Acute Respiratory Syndrome Coronavirus 2 Eradication | No comparisons made between pharmacologic agents |
| Million et al. | Early treatment of COVID-19 patients with hydroxychloroquine and azithromycin: A retrospective analysis of 1061 cases in Marseille, France | No comparisons made between pharmacologic agents |
| Antony et al. | Early use of tocilizumab in the prevention of adult respiratory failure in SARS-CoV-2 infections and the utilization of interleukin-6 levels in the management | No comparisons made between pharmacologic agents |
| Zeng et al. | Effect of Convalescent Plasma Therapy on Viral Shedding and Survival in Patients With Coronavirus Disease 2019 | No comparisons made between pharmacologic agents |
| Marzolini et al. | Effect of Systemic Inflammatory Response to SARS-CoV-2 on Lopinavir and Hydroxychloroquine Plasma Concentrations | No comparisons made between pharmacologic agents |
| ElSeirafi et al. | Efficacy and safety of tocilizumab in critically ill adults with COVID-19 infection in Bahrain: A report of 5 cases | No comparisons made between pharmacologic agents |
| Dimopoulos et al. | Favorable Anakinra Responses in Severe Covid-19 Patients with Secondary Hemophagocytic Lymphohistiocytosis | No comparisons made between pharmacologic agents |
| Temesgen et al. | First Clinical Use of Lenzilumab to Neutralize GM-CSF in Patients with Severe COVID-19 Pneumonia | No comparisons made between pharmacologic agents |
| Hong et al. | Five severe COVID-19 pneumonia patients treated with triple combination therapy with lopinavir/ritonavir, hydroxychloroquine, and interferon β-1b | No comparisons made between pharmacologic agents |
| Xiang et al. | Glucocorticoids improve severe or critical COVID-19 by activating ACE2 and reducing IL-6 levels | No comparisons made between pharmacologic agents |
| So et al. | High-dose, short-term corticosteroids for ARDS caused by COVID-19: a case series | No comparisons made between pharmacologic agents |
| Quartuccio et al. | Higher levels of IL-6 early after tocilizumab distinguish survivors from nonsurvivors in COVID-19 pneumonia: A possible indication for deeper targeting of IL-6 | No comparisons made between pharmacologic agents |
| Hartman et al. | Hospitalized COVID-19 Patients Treated With Convalescent Plasma in a Mid-size City in The Mid West | No comparisons made between pharmacologic agents |
| Hartman et al. | Hospitalized COVID-19 Patients treated with Convalescent Plasma in a Mid-size City in the Midwest | Duplicate |
| Hartman et al. | Hospitalized COVID-19 patients treated with Convalescent Plasma in a mid-size city in the midwest | Duplicate |
| Xue et al. | Hydroxychloroquine treatment in COVID-19: a descriptive observational analysis of 30 cases from a single center in Wuhan, China | No comparisons made between pharmacologic agents |
| Olivares-Gazca et al. | INFUSION OF CONVALESCENT PLASMA IS ASSOCIATED WITH CLINICAL IMPROVEMENT IN CRITICALLY ILL PATIENTS WITH COVID-19: A PILOT STUDY | No comparisons made between pharmacologic agents |
| Liu et al. | Lessons learned from early compassionate use of convalescent plasma on critically ill patients with Covid-19 | No comparisons made between pharmacologic agents |
| Zuo et al. | Lopinavir/ritonavir and interferon combination therapy may help shorten the duration of viral shedding in patients with COVID-19: A retrospective study in two designated hospitals in Anhui, China | No comparisons made between pharmacologic agents |
| Perotti et al. | Mortality reduction in 46 severe Covid-19 patients treated with hyperimmune plasma. A proof of concept single arm multicenter trial | No comparisons made between pharmacologic agents |
| Morena et al. | Off-label use of tocilizumab for the treatment of SARS-CoV-2 pneumonia in Milan, Italy | No comparisons made between pharmacologic agents |
| Liu et al. | Patients of COVID-19 may benefit from sustained Lopinavir-combined regimen and the increase of Eosinophil may predict the outcome of COVID-19 progression | No comparisons made between pharmacologic agents |
| Lê et al. | Pharmacokinetics of lopinavir/ritonavir oral solution to treat COVID-19 in mechanically ventilated ICU patients | Pharmacokinetics study |
| Sciascia et al. | Pilot prospective open, single-arm multicentre study on off-label use of tocilizumab in patients with severe COVID-19 | Single arm study |
| Morath et al. | Plasma exchange in critically ill COVID-19 patients | Plasma exchange |
| Zhang et al. | Potential of Arbidol for Post-exposure Prophylaxis of COVID-19 Transmission: A Preliminary Report of a Retrospective Cohort Study | Focus on prophylaxis |
| Lohse et al. | Predictive factors of mortality in patients treated with tocilizumab for acute respiratory distress syndrome related to coronavirus disease 2019 (COVID-19) | No comparisons made between pharmacologic agents |
| Jung et al. | Re: Low-dose corticosteroid therapy does not delay viral clearance in patients with COVID-19 | Reply |
| Selvaraj et al. | Short-Term Dexamethasone in Sars-CoV-2 Patients | No comparisons made between pharmacologic agents |
| Dastan et al. | Subcutaneous administration of interferon beta-1a for COVID-19: A non-controlled prospective trial | No comparisons made between pharmacologic agents |
| Mastroianni et al. | Subcutaneous tocilizumab treatment in patients with severe COVID-19-related cytokine release syndrome: An observational cohort study | No comparisons made between pharmacologic agents |
| Petrulewicz et al. | The clinical course and short-term outcome of COVID-19 in a cohort of hemodialysis patients | Full text not retrievable |
| Hazbun et al. | The Combination of Tocilizumab and Methylprednisolone Along With Initial Lung Recruitment Strategy in Coronavirus Disease 2019 Patients Requiring Mechanical Ventilation: A Series of 21 Consecutive Cases | No comparisons made between pharmacologic agents |
| Borku Uysal et al. | Tocilizumab challenge: A series of cytokine storm therapy experiences in hospitalized COVID-19 pneumonia patients | No comparisons made between pharmacologic agents |
| Qian et al. | Tocilizumab exerts anti-inflammatory activity in six critically ill COVID-19 patients: a retrospective analysis | No comparisons made between pharmacologic agents |
| Antinori et al. | Tocilizumab for cytokine storm syndrome in COVID-19 pneumonia: an increased risk for candidemia? | No comparisons made between pharmacologic agents |
| Tomasiewicz et al. | Tocilizumab for patients with severe COVID-19: a retrospective, multi-center study | No comparisons made between pharmacologic agents |
| Fernández-Ruiz et al. | Tocilizumab for the treatment of adult patients with severe COVID-19 pneumonia: A single-center cohort study | No comparisons made between pharmacologic agents |
| Knorr et al. | Tocilizumab in patients with severe COVID-19: A single-center observational analysis | No comparisons made between pharmacologic agents |
| Luo et al. | Tocilizumab treatment in COVID-19: A single center experience | No comparisons made between pharmacologic agents |
| Zhang et al. | Treatment With Convalescent Plasma for Critically Ill Patients With Severe Acute Respiratory Syndrome Coronavirus 2 Infection | No comparisons made between pharmacologic agents |
| Titanji et al. | Use of Baricitinib in Patients with Moderate and Severe COVID-19 | No comparisons made between pharmacologic agents |
| Hegerova et al. | Use of convalescent plasma in hospitalized patients with COVID-19: case series | Case series |
| Hassoun et al. | Utilizing tocilizumab for the treatment of cytokine release syndrome in COVID-19 | Case series |
| Zhang et al. | A comparative study on the time to achieve negative nucleic acid testing and hospital stays between Danoprevir and Lopinavir/Ritonavir in the treatment of patients with COVID-19 | Not providing sufficient statistical data for analysis |
| Moschini et al. | Effects on QT interval of hydroxychloroquine associated with ritonavir/  darunavir or azithromycin in patients with SARS‑CoV‑2 infection | No isolated HQ groups. HQ/RD combination may induce confounding in QT interval analysis. |
| Yang et al. | Effectiveness of Arbidol for COVID-19 Prevention in Health Professionals | Included uninfected patients (prophylactic use of arbidol) |
| Langer-Gould et al. | Early Identification of COVID-19 Cytokine Storm and Treatment with  Anakinra or Tocilizumab | Difference in baseline severities between Anakinra and Tocilizumab groups are too large to be sufficiently adjusted by statistical approaches (intubated patients account for 56.1% for anakinra group while 96.2% in tocilizumab group). |
| Yu et al. | COVID-19 patients benefit from early antiviral treatment：a comparative, retrospective study | Early versus delayed intervention |
| Hu et al. | Corticosteroid, oseltamivir and delayed admission are independent risk factors for prolonged viral shedding in patients with Coronavirus Disease 2019 | Presented data are not in usable format. |
| Gao et al. | A retrospective evaluation on the efficacy of Lopinavir/ritonavir and chloroquine to treat non-severe COVID-19 patients | No sufficient information on baseline of each medication group (too rough) |
| Chopra et al. | Corticosteroid Administration Is Associated With Improved Outcome in Patients With Severe Acute Respiratory Syndrome Coronavirus 2-Related Acute Respiratory Distress Syndrome | Does not present the outcomes of interest (e.g., morality, aggravation, etc) |
| Kelly et al. | Clinical outcomes and adverse events in patients hospitalized with COVID-19, treated with off-label hydroxychloroquine and azithromycin | Significant differences in baseline characteristics between treatment and non-treatment groups while not adjusted by any means. |
| Xu et al. | Arbidol/IFN-a2b therapy for patients with corona virus disease 2019: a  retrospective multicenter cohort study | Uncontrolled baseline |
| Scarsi et al. | Association between treatment with colchicine and improved survival in a single-centre cohort of adult hospitalised patients with COVID-19 pneumonia and acute respiratory distress syndrome | Uncontrolled baselines between groups and lack of evidence for multivariable adjustment. |
| Mazzanti et al. | Association of Hydroxychloroquine With QTc Interval in Patients With COVID-19 | Required data for meta-analysis is absent |
| Bhandari et al. | Characteristics, Treatment Outcomes and Role of Hydroxychloroquine among 522 COVID-19 hospitalized patients in Jaipur City: An Epidemio-Clinical Study | Limited data for baseline characteristics; unable to judge whether groups are sufficiently controlled or not. |
| Choi et al. | Clinical Characteristics and Disease Progression in Early-Stage COVID-19 Patients in South Korea | Absence of outcomes of interest. |
| Moreno-Perez et al. | Experience with tocilizumab in severe COVID-19 pneumonia after 80 days of  follow-up: A retrospective cohort study | Uncontrolled baselines between groups and lack of multivariable adjustment for tocilizumab versus control. |
| Yan et al. | Factors associated with prolonged viral shedding and impact of lopinavir/ ritonavir treatment in hospitalized non-critically ill patients with SARS-CoV-2 infection | Required data for meta-analysis is absent |
| Klopfenstein et al. | Tocilizumab therapy reduced intensive care unit admissions and/or mortality in COVID-19 patients | Uncontrolled baseline |
| Kewan et al. | Tocilizumab for treatment of patients with severe COVID_19:A retrospective cohort study | Uncontrolled baseline |
| NA | The use of hydroxychloroquine plus azithromycin and early hospital admission are beneficial in Covid-19 patients: Turkey experience with real-life data | No sufficient information on baseline of each medication group |
| Sinkeler et al. | The risk of QTc-interval prolongation in COVID-19 patients treated with chloroquine | A single arm study |
| Vahedi et al. | The clinical value of two combination regimens in the Management of Patients Suffering from Covid-19 pneumonia: a single centered, retrospective, observational study | No sufficient information on baseline of each medication group |
| Erkurt et al. | Life-saving effect of convalescent plasma treatment in covid-19 disease:  Clinical trial from eastern Anatolia | A single arm study; no control group |
| Pettit et al. | Late Onset Infectious Complications and Safety of Tocilizumab in the Management of COVID-19 | Lack of evidence to judge whether experimental group and control group are sufficiently controlled at their baselines. |
| Zhou et al. | Interferon-a2b Treatment for COVID-19 | Absence of outcomes of interest. |
| Lofgren et al. | Safety of Hydroxychloroquine among Outpatient Clinical Trial Participants for COVID-19 | Not hospitalized patients |
| Ong et al. | Safety and potential efficacy of cyclooxygenase-2 inhibitors in coronavirus disease 2019 | Uncontrolled baselines |
| Potere et al. | Interleukin-6 receptor blockade with subcutaneous tocilizumab in severe COVID-19 pneumonia and hyperinflammation: a case–control study | No sufficient information on baseline of each medication group |
| Panagopoulos et al. | Lopinavir/ritonavir as a third agent in the antiviral regimen for SARS-CoV-2 infection | Uncontrolled baselines |
| Potere et al. | Low-Dose Subcutaneous Tocilizumab to Prevent Disease Progression in  Patients with Moderate COVID-19 Pneumonia and Hyperinflammation | Uncontrolled baselines (especially, severity profiles between two groups are different) |
| Coenen et al. | Oseltamivir for coronavirus illness: post-hoc exploratory analysis of an open-label, pragmatic, randomized controlled trial in European primary care from 2016 to 2018 | Exploratory analysis of data from an open-label, pragmatic, randomised controlled trial during three influenza seasons, from 2016 to 2018 (not including SARS-CoV2) |
| Rojas-Marte et al. | Outcomes in patients with severe COVID-19 disease treated with tocilizumab: a case–controlled study | Not matched |
| Lagier et al. | Outcomes of 3,737 COVID-19 patients treated with hydroxychloroquine/  azithromycin and other regimens in Marseille, France: A retrospective analysis | Heterogeneous treatment and control groups; control group also includes HQ/AZ administrated patients. |
| Tuncer et al. | QT interval evaluation associated with the use of hydroxychloroquine with combined use of azithromycin among hospitalised children positive for coronavirus disease 2019 | Children |
| Hsia et al. | QT prolongation in a diverse, urban population of COVID-19 patients treated with hydroxychloroquine, chloroquine, or azithromycin | A single arm study |
| Esquivel-Moynelo et al. | Effect of combination of interferon alpha-2b and interferon-gamma or interferon alpha-2b alone for elimination of SARS-CoV-2 viral RNA**.** Preliminary results of a randomized controlled clinical trial. | Absence of outcomes of interest. |
| Chen et al. | Effectiveness of Convalescent Plasma for Treatment of COVID-19 Patients | Not a comparative study |
| Carvalho et al. | Effects of Tocilizumab in Critically Ill Patients With COVID-19: A Quasi-Experimental Study | Absence of outcomes of interest. |
| Wang et al. | Efficacy and Safety of Leflunomide for Refractory COVID-19: An Open-label Controlled Study | Investigated on refractory COVID-19 patients (SARS-CoV-2 positive for more than 28 days despite standard care), which were not a target population of our research. |
| Hoertel et al. | Observational Study of Haloperidol in Hospitalized Patients with Covid-19 | No isolated data for intubation and mortality |
| Jee et al. | Oncologic Immunomodulatory Agents in Patients with Cancer and COVID-19 | Cancer patients |
| Vasylyeva et al. | Remdesivir for COVID-19: match-population analysis with compassionate use of Remdesivir for severe COVID-19 | Uncontrolled baseline |
| Pereda et al. | Therapeutic effectiveness of interferon-alpha 2b against COVID-19: the Cuban experience | No sufficient summary statistics for meta-analysis |
| Mikulska et al. | Tocilizumab and steroid treatment in patients with COVID-19 pneumonia. | Mixed drug regimen (unable to identify isolated effect of tocilizumab and steroid) |
| Elharrar et al. | Use of Prone Positioning in Nonintubated Patients With COVID-19 and Hypoxemic Acute Respiratory Failure | Not a pharmacological intervention study |
| Keller et al. | Effect of Systemic Glucocorticoids on Mortality or Mechanical Ventilation in Patients With COVID-19 | Lack of evidence to judge whether experimental group and control group are sufficiently controlled at their baselines |
| Coppo et al. | Feasibility and physiological effects of prone positioning in non-intubated patients with acute respiratory failure due to COVID-19 (PRON-COVID): a prospective cohort study | Not a pharmacological intervention study |
| Xiao et al. | Efficacy of Huoxiang Zhengqi dropping pills and Lianhua Qingwen granules in treatment of COVID-19: A randomized controlled trial | Unclear drug mix in Western Medicine (WM)group potentially prevent clarifying the isolated effect of each drug. |
