# Supplementary material for: Comparative efficacy and safety of pharmacological interventions for the treatment of COVID-19: A systematic review and network meta-analysis
Source: PLoS Med. 2020 Dec 30;17(12):e1003501. doi: 10.1371/journal.pmed.1003501 (PMC7794037; doi:10.1371/journal.pmed.1003501)
Supplement: S1 Text — (DOCX) [file pmed.1003501.s007.docx]

**Search Strategy**

**PubMed**

(COVID-19 [mh] OR SARS-CoV-2[mh] OR coronavirus[mh])

**AND**

(treat*[ti] OR EIDD[tiab] OR antiviral[tiab] OR flu[ti] OR favipiravir[tiab] OR avigan[tiab] OR umifenovir[tiab] OR arbidol[tiab] OR chloroquine[tiab] OR hydroxychloroquine[tiab] OR azithromycin[tiab] OR antibiotics[tiab] OR ebola[ti] OR remdesivir[tiab] OR HIV[ti] OR kaletra[tiab] OR lopinavir[tiab] OR ritonavir[tiab] OR actemra[tiab] OR tocilizumab[tiab] OR interleukin[tiab] OR sarilumab[tiab] OR kevzara[tiab] OR corticosteroid[tiab] OR prednisolone[tiab] OR ACEi[tiab] OR angiotensin[tiab] OR malari*[tiab])

**AND**

(randomized [tw] OR randomized controlled trial [tiab] OR RCT [tw] OR label*[tiab] OR retrospective[tiab] OR observational[tiab] OR intensive care unit [tiab] OR critically ill [tiab] OR severe [tiab] OR moderate [tiab] OR inpatient [tiab] OR ARDS [tw] OR acute respiratory distress syndrome [tw])

**NOT**

(non-pharmacological intervnetion[tw] or mirror[tiab] or pilot[tw] or autobiography[pt] or bibliography[pt] or biography[pt] or case reports[pt] or congresses[pt] or editorial[pt] or government publications[pt] or interview[pt] or lectures[pt] or legal cases[pt] or legislation[pt] or comment or ("Animals"[Mesh] NOT "Humans"[Mesh]) OR rats[tw] or cow[tw] or cows[tw] or chicken*[tw] or horse[tw] or horses[tw] or mice[tw] or mouse[tw])

Search strategies used for other databases are fairly identical or slightly modified on the circumstance of each database.
