# Supplementary material for: Comparative efficacy and safety of pharmacological interventions for the treatment of COVID-19: A systematic review and network meta-analysis
Source: PLoS Med. 2020 Dec 30;17(12):e1003501. doi: 10.1371/journal.pmed.1003501 (PMC7794037; doi:10.1371/journal.pmed.1003501)
Supplement: S2 Text — (DOCX) [file pmed.1003501.s008.docx]

**Appendix for method**

**Definitions and outcomes**

The primary outcomes were mortality rate, progression rate to severe pneumonia or intensive care unit (ICU) admission, time to viral clearance, QTc prolongation, fatal cardiac adverse event rate, and non-cardiac serious adverse event rate. Fatal cardiac complications include torsades de pointes (TdP), cardiac arrest, and severe ventricular arrhythmia, while non-cardiac serious complications include septic shock, acute gastritis, hemorrhage of lower digestive track, unconsciousness, decreased glomerular filtration rate (acute kidney injury), respiratory distress, pulmonary embolism, hypotension, as specified per study. Non-lethal complications such as nausea, vomiting and diarrhea that required discontinuation of treatment were also included. QTc prolongation was defined as QTc interval >500ms or change in QTc interval (ΔQTc) of >60ms after treatment.

Some observational studies were deemed at potentially serious risk of confounding bias; however, they were included in our primary analysis because 1) the confounding present in these studies were deemed unlikely to impact our analysis and 2) exclusion of the study may introduce selection bias in our analysis as explained in detail in Appendix.

We used hydroxychloroquine and chloroquine interchangeably in our analysis as they share common mechanism. However, we preferentially used HCQ when study provided data on both HCQ and CQ. Most of results derived from HCQ, and CQ composes very minor.

For some HQ studies, only the adverse event-related data from this study was used in this network meta-analysis, as this study investigates the effect of hydroxychloroquine as a prophylactic measure which is not the focus of our meta-analysis. We allowed to include non-hospitalized patient exceptionally in analyses for adverse events, as low incident adverse events warrant longer observation.
